# Comparison of Glucose-Lowering Drugs as Second-Line Treatment for Type 2 Diabetes: A Systematic Review and Meta-Analysis

**DOI:** 10.3390/jcm11185435

**Published:** 2022-09-16

**Authors:** Shuyan Gu, Xiaoqian Hu, Xuemei Zhen, Lizheng Shi, Hui Shao, Xueshan Sun, Yuxuan Gu, Minzhuo Huang, Hengjin Dong

**Affiliations:** 1 Center for Health Policy and Management Studies, School of Government, Nanjing University, Nanjing 210023, China; 2 College of Politics and Public Administration, Qingdao University, Qingdao 266071, China; 3 Centre for Health Management and Policy Research, School of Public Health, Cheeloo College of Medicine (NHC Key Laboratory of Health Economics and Policy Research), Shandong University, Jinan 250012, China; 4 Department of Global Health Management and Policy, School of Public Health and Tropical Medicine, Tulane University, New Orleans, LA 70112, USA; 5 Department of Pharmaceutical Outcomes and Policy, College of Pharmacy, University of Florida, Gainesville, FL 32611, USA; 6 Center for Health Policy Studies, School of Public Health, Zhejiang University School of Medicine, Hangzhou 310058, China

**Keywords:** type 2 diabetes, second-line treatment, sulfonylureas, glinides, thiazolidinediones, α-glucosidase inhibitors, dipeptidyl peptidase-4 inhibitors, sodium-glucose cotransporter-2 inhibitors, glucagon-like peptide-1 receptor agonists, insulins

## Abstract

Background: Multiple glucose-lowering drugs are available as add-ons to metformin for a second-line treatment for type 2 diabetes. However, no systematic and comparative data are available for them in China. We aimed to compare the effects of glucose-lowering drugs added to metformin in China. Methods: PubMed, Embase, Web of Science, CNKI, WanFang Data, and Chongqing VIP from 1 January 2000 until 31 December 2020 were systematically searched for randomized controlled trials comparing a glucose-lowering drug added to metformin with metformin in Chinese type 2 diabetes patients. Drug classes included sulfonylureas (SUs), glinides (NIDEs), thiazolidinediones (TZDs), α-glucosidase inhibitors (AGIs), dipeptidyl peptidase-4 inhibitors (DPP-4is), sodium-glucose cotransporter-2 inhibitors (SGLT2is), glucagon-like peptide-1 receptor agonists (GLP-1RAs), and insulins (INSs). Two reviewers independently screened studies, extracted data, and appraised the risk of bias. Results: 315 trials were included. In patients receiving metformin alone, the addition of NIDEs produced the greatest additional HbA1c reductions (1.29%; 95% CI 0.97, 1.60); while INSs yielded both the largest additional FPG reductions (1.58 mmol/L; 95% CI 1.22, 1.94) and 2 hPG reductions (2.52 mmol/L; 95% CI 1.83, 3.20). INS add-ons also conferred the largest additional HDL-C increases (0.40 mmol/L; 95% CI 0.16, 0.64), whereas AGI add-ons generated the greatest TC reductions (1.08 mmol/L; 95% CI 0.78, 1.37). The greatest incremental SBP reductions (6.65 mmHg; 95% CI 4.13, 9.18) were evident with SGLT2i add-ons. GLP-1RA add-ons had the greatest BMI reductions (1.96 kg/m^2^, 95% CI 1.57, 2.36), meanwhile with the lowest (0.54 time) hypoglycemia risk. Overall, only the GLP-1RA add-ons demonstrated a comprehensive beneficial effect on all outcomes. Furthermore, our results corroborated intraclass differences among therapies. Given the limited evidence, we could not reach a conclusion about the optimal therapies regarding mortality and vascular outcomes. Conclusion: The results suggested a potential treatment hierarchy for clinicians and patients, with the GLP-1RA add-ons being most preferred based on their favorable efficacy and safety profiles; and provided a unified hierarchy of evidence for conducting country-specific cost-effectiveness analyses.

## 1. Introduction

Type 2 diabetes imposes serious threats to national health and economy [1]. China had the world’s largest diabetes population of 116.4 million in 2019, with a related health expenditure of $109.0 billion [1]. Type 2 diabetes as a progressive disease requires stepwise intensified treatments to maintain glycemic targets [2,3,4]. Clinical guidelines recommend metformin (MET) as the first-line therapy, and once the glycemic target is not achieved, a dual therapy of a glucose-lowering drug added to MET is proceeded to as a second-line treatment [2,3]. Eight classes of add-on drugs are available, including sulfonylureas (SUs), glinides (NIDEs), α-glucosidase inhibitors (AGIs), thiazolidinediones (TZDs), dipeptidyl peptidase 4 inhibitors (DPP-4is), sodium-glucose cotransporter 2 inhibitors (SGLT2is), glucagon-like peptide-1 receptor agonists (GLP-1RAs), and insulins (INSs) [2]. These many optional drugs have increased the complexity of therapy choice in clinical practice, and increased uncertainty regarding the optimal MET-based dual therapy for patients. The most commonly-used add-on drugs in China were SUs (42.7%), followed by AGIs (35.9%) and NIDEs (27.5%) [5]. However, 32% of the patients altered their therapies in one year, citing poor effectiveness or intolerant adverse events [5]. The suboptimal medication adherence in turn impedes treatment effects, further increasing the risks of diabetes-related vascular events and medical costs [6,7,8].

It is of value to choose rational drugs for individualized patients as add-ons to MET. The choice of which drug to add is based on drug-specific effects and patient factors [2,3,4]. The evidence demonstrates that focusing only on strict glycemic control has a limited effect on reducing the risks of diabetes-related vascular events for type 2 diabetes patients, especially those with longer diabetes duration, older age, or multiple cardiovascular risk factors (e.g., dyslipidemia, obesity/overweight, and hypertension); while a comprehensive control of these risk factors can obtain greater clinical benefits and play a better role in preventing vascular events [2,9,10]. Clinical guidelines recommend a patient-centered approach when choosing therapies, with the primary goal of achieving glycemic targets, whilst actively managing other cardiovascular risk factors, minimizing adverse events, and improving vascular outcomes based on patient factors [2,3,11].

Evidence-based therapeutic decision making requires comparisons of all relevant competing treatments [12]. Although there are numerous trials comparing MET-based dual therapies with MET alone, limited evidence is available to support one combination over another across all the MET-based dual therapies [3]. Notably, no systematic review has compared all available MET-based dual therapies with regard to a full range of intermediate outcomes (e.g., blood glucose, body weight, lipids, and blood pressure) and other clinically important outcomes (e.g., adverse events and cardiovascular risks) in China. Therefore, this study systematically summarized and compared the treatment effects of all available glucose-lowering drugs added to MET as second-line treatment for type 2 diabetes in China, to help clinicians and patients choose rational therapies in clinical practice.

## 2. Methods

This study followed the statement for Preferred Reporting Items for Systematic Reviews and Meta-Analysis [13] and was registered at PROSPERO (CRD42021223933). Trials comparing MET-based dual therapy with MET alone are common in China. We approached the comparisons across MET-based dual therapies by using MET as a common comparator.

### 2.1. Data Sources and Searches

PubMed, Embase, Web of Science, China National Knowledge Infrastructure (CNKI), WanFang Data, and Chongqing VIP were systematically searched for randomized controlled trials (RCTs) published between 1 January 2000 and 31 December 2020, which compared a glucose-lowering drug added to MET with MET in Chinese type 2 diabetes patients. Search strategies are detailed in Supplementary Materials Table S1. The references of retrieved RCTs and relevant reviews were manually examined to identify additional trials.

### 2.2. Study Selection

Studies were eligible if the (*1*) participants were Chinese adults with type 2 diabetes; (*2*) intervention was a glucose-lowering drug added to MET, whose usage and dosage met the recommendation of Chinese clinical guideline [14], and MET was administered at a constant dose; (*3*) comparison was a MET monotherapy or placebo added to MET, whose usage and dosage were the same as that in intervention; (*4*) background therapy was limited to lifestyle intervention; (*5*) primary outcome was hemoglobin Alc (HbA1c), and secondary outcomes were fasting plasma glucose (FPG), 2 h postprandial plasma glucose (2 hPG), body mass index (BMI), total cholesterol (TC), high-density lipoprotein-cholesterol (HDL-C), systolic blood pressure (SBP), hypoglycemia, renal function, lactic acidosis, mortality, or vascular outcomes; (*6*) study design was RCT; (*7*) study duration was ≥12 weeks; and (*8*) study was published in Chinese or English. Eligibility criteria are detailed in Supplementary Materials Table S2.

A total of 26 drugs from eight classes approved for type 2 diabetes in China were targeted [2]:
SUsGlyburide, glimepiride, gliclazide, glipizide, gliquidone NIDEsRepaglinide, nateglinide, mitiglinide TZDsRosiglitazone, pioglitazone AGIsAcarbose, voglibose, miglitol DPP-4isSitagliptin, saxagliptin, vildagliptin, linagliptin, alogliptin SGLT2isDapagliflozin, empagliflozin, canagliflozin GLP-1RAsExenatide, liraglutide, lixisenatide, beinaglutide INSsInsulin and insulin analogs 

After deduplication, two reviewers (S.G. and X.H.) independently screened the titles and abstracts of retrieved records, and examined the full texts of potentially eligible records. The results were cross-checked by two reviewers (Y.G. and M.H.) with any discrepancies resolved by consensus.

### 2.3. Data Extraction and Quality Assessment

Two reviewers (S.G. and X.S.) independently extracted data using a structured form, and appraised the risk of bias of the trials using the Cochrane Collaboration’s risk of bias assessment tool [15]. The extracted data comprised the study design, study and participant characteristics, intervention and comparison, outcomes, and other data. The publication bias was assessed using Egger’s test or Harbord’s modified test. The results were cross-checked by two reviewers (Y.G. and M.H.) with any discrepancies resolved by a consensus.

### 2.4. Data Synthesis and Analysis

Initially, a series of meta-analyses were conducted to assess the treatment effects of MET-based dual therapies vs. MET on each outcome. Then, using MET as a common comparator, the adjusted indirect treatment comparisons based on the Bucher method were used to compare the treatment effects of MET-based dual therapies with each other [16,17]. We calculated the weighted mean differences (WMD) with 95% confidence intervals (CIs) for continuous outcomes, and risk ratios (RR) with 95% CIs for dichotomous outcomes. We conservatively used a random-effects model assuming a substantial variability in effect size across therapies and studies [18,19]. We used the *I*^2^ statistic to assess heterogeneity and considered it important when it was greater than 50% [20]. The subgroup analyses were conducted based on individual drugs within the same class. Sensitivity analyses were run by using fixed-effects meta-analyses. All analyses were conducted in Stata/SE 15.1 (StataCorp LLC, College Station, TX, USA).

## 3. Results

### 3.1. Overview of Trials

**Figure 1:**
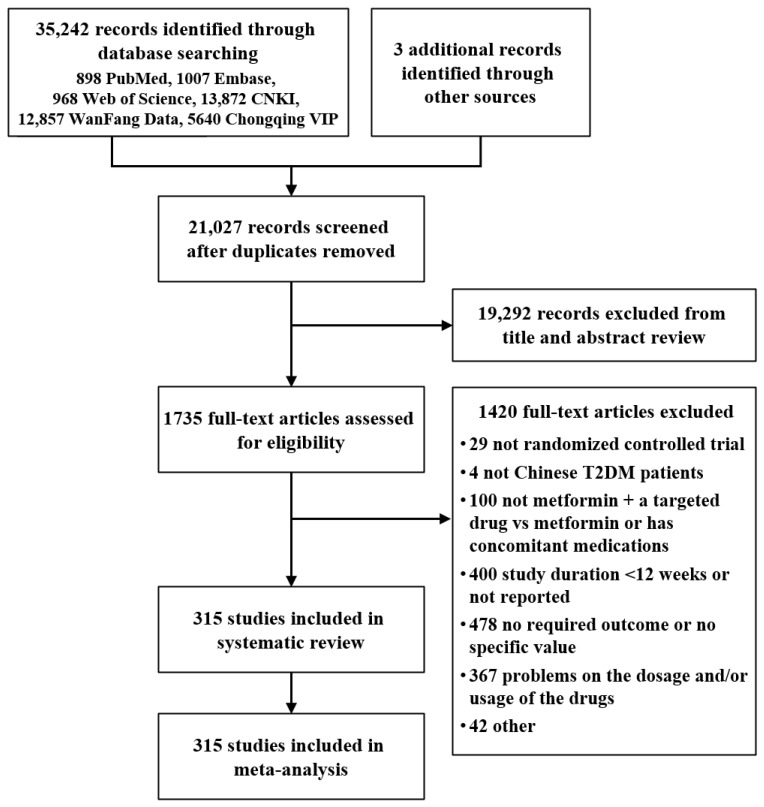
Flow diagram of study selection. *CNKI*, *China National Knowledge Infrastructure*.

The initial searches retrieved 35,245 records, of which 1735 full texts were assessed for eligibility. Finally, 315 trials were included [21,22,23,24,25,26,27,28,29,30,31,32,33,34,35,36,37,38,39,40,41,42,43,44,45,46,47,48,49,50,51,52,53,54,55,56,57,58,59,60,61,62,63,64,65,66,67,68,69,70,71,72,73,74,75,76,77,78,79,80,81,82,83,84,85,86,87,88,89,90,91,92,93,94,95,96,97,98,99,100,101,102,103,104,105,106,107,108,109,110,111,112,113,114,115,116,117,118,119,120,121,122,123,124,125,126,127,128,129,130,131,132,133,134,135,136,137,138,139,140,141,142,143,144,145,146,147,148,149,150,151,152,153,154,155,156,157,158,159,160,161,162,163,164,165,166,167,168,169,170,171,172,173,174,175,176,177,178,179,180,181,182,183,184,185,186,187,188,189,190,191,192,193,194,195,196,197,198,199,200,201,202,203,204,205,206,207,208,209,210,211,212,213,214,215,216,217,218,219,220,221,222,223,224,225,226,227,228,229,230,231,232,233,234,235,236,237,238,239,240,241,242,243,244,245,246,247,248,249,250,251,252,253,254,255,256,257,258,259,260,261,262,263,264,265,266,267,268,269,270,271,272,273,274,275,276,277,278,279,280,281,282,283,284,285,286,287,288,289,290,291,292,293,294,295,296,297,298,299,300,301,302,303,304,305,306,307,308,309,310,311,312,313,314,315,316,317,318,319,320,321,322,323,324,325,326,327,328,329,330,331,332,333,334,335], with a total of 35,022 patients identified, which were randomly assigned to 20 drugs plus MET or MET alone (Figure 1). The sample size of the trials varied from 38 to 800, with a study duration ranging from 12 to 52 weeks. Sitagliptin+MET were studied in 66 trials, with liraglutide+MET (38 trials), pioglitazone+MET (32 trials), and acarbose+MET (32 trials) being the three next most studied therapies. No eligible trial for glyburide, nateglinide, mitiglinide, canagliflozin, lixisenatide, or beinaglutide was found. All trials focused on reporting intermediate outcomes, with only nine trials mentioning mortality or vascular outcomes. Mean age of the participants ranged from 28 to 79.02 years, with a diabetes duration of 0.02 to 18.50 years. The characteristics and risk of bias of the trials are shown in Supplementary Materials Table S3. The publication bias across studies is shown in Supplementary Materials Figure S1.

### 3.2. Intermediate Outcomes

#### 3.2.1. Hemoglobin Alc

A total of 315 trials (35,022 patients) provided data on HbA1c. In patients receiving MET alone, the addition of a glucose-lowering drug generated significant additional HbA1c reductions. The greatest reductions were observed with NIDEs (1.29%; 95% CI 0.97, 1.60), followed by SUs (1.16%; 95% CI 0.90, 1.43) and GLP-1RAs (1.14%; 95% CI 1.01, 1.28); while TZDs (0.79%; 95% CI 0.60, 0.99) conferred the smallest reductions. There were large differences in HbA1c reductions found within SUs, ranging from 0.86% (95% CI 0.59, 1.13) with glipizide to 1.70% (95% CI 0.68, 2.72) with gliclazide, and within DPP-4is, ranging from 0.76% (95% CI 0.63, 0.89) with vildagliptin to 1.35% (95% CI 0.80, 1.90) with alogliptin (Table 1; Supplementary Materials Figures S2–S9).

Across all dual therapies, NIDEs+MET, SUs+MET, and GLP-1RAs+MET had significantly greater HbA1c reductions than TZDs+MET, ranging from 0.35% (95% CI 0.11, 0.59) with GLP-1RAs+MET to 0.49% (95% CI 0.12, 0.86) with NIDEs+MET. No significant difference was noted between other comparisons (Table 2).

#### 3.2.2. Fasting Plasma Glucose

A total of 311 trials (33,848 patients) provided data on FPG. When used as add-ons to MET, all drug classes produced significantly incremental FPG reductions, among which INSs (1.58 mmol/L; 95% CI 1.22, 1.94) yielded the largest reductions, followed by NIDEs (1.43 mmol/L; 95% CI 1.03, 1.83) and GLP-1RAs (1.33 mmol/L; 95% CI 1.14, 1.53). As a class, SGLT2is (0.87 mmol/L; 95% CI 0.62, 1.11) conferred the smallest reductions, which were inferior to AGIs (0.97 mmol/L; 95% CI 0.82, 1.12); but there were large differences within AGIs, with a 1.33 mmol/L (95% CI 0.62, 2.04) reduction with voglibose vs. no significant reduction with miglitol. Considerable differences were also found within SUs, ranging from 1.01 mmol/L (95% CI 0.64, 1.38) with glipizide to 2.20 mmol/L (95% CI 1.78, 2.62) with gliquidone (Table 1; Supplementary Materials Figures S10–S17).

Amongst all dual therapies, INSs+MET and GLP-1RAs+MET demonstrated significantly greater FPG reductions than DPP-4is+MET, TZDs+MET, AGIs+MET, and SGLT2is+MET, with reductions ranging from 0.23 mmol/L (95% CI 0.02, 0.45) with GLP-1RAs+MET vs. DPP-4is+MET to 0.71 mmol/L (95% CI 0.28, 1.15) with INSs+MET vs. SGLT2is+MET. In addition, NIDEs+MET were superior to AGIs+MET and SGLT2is+MET, whilst SUs+MET were superior to SGLT2is+MET (Table 2).

#### 3.2.3. The 2 h Postprandial Plasma Glucose

A total of 282 trials (30,651 patients) provided data on 2 hPG. The addition of a glucose-lowering drug to MET reported significant additional 2 hPG reductions. The largest reductions were 2.52 mmol/L (95% CI 1.83, 3.20) evident with INSs, followed by 1.90 mmol/L (95% CI 1.34, 2.47) with NIDEs and 1.90 mmol/L (95% CI 1.48, 2.33) with SUs; while the smallest reductions were 1.44 mmol/L (95% CI 1.07, 1.81) induced by SGLT2is. There were considerable differences in 2 hPG reductions within AGIs, ranging from 1.27 mmol/L (95% CI 0.40, 2.13) with miglitol to 3.29 mmol/L (95% CI 2.19, 4.39) with voglibose; within SUs, ranging from 1.70 mmol/L (95% CI 0.65, 2.76) with gliclazide to 2.97 mmol/L (95% CI 2.30, 3.64) with gliquidone; and within DPP-4is, ranging from 1.20 mmol/L (95% CI 0.73, 1.66) with vildagliptin to 2.03 mmol/L (95% CI 1.83, 2.22) with sitagliptin (Table 1; Supplementary Materials Figures S18–S25).

Across all dual therapies, the only significant differences noted were between INSs+MET and DPP-4is+MET, TZDs+MET, GLP-1RAs+MET, AGIs+MET, or SGLT2is+MET. INSs+MET were favored over these therapies, with 2 hPG reductions ranging from 0.76 mmol/L (95% CI 0.06, 1.46) vs. DPP-4is+MET to 1.08 mmol/L (95% CI 0.30, 1.86) vs. SGLT2is+MET (Table 2).

#### 3.2.4. Body Mass Index

A total of 104 trials (10,650 patients) provided data on BMI. As add-ons to MET, GLP-1RAs, AGIs, SGLT2is, and DPP-4is generated significantly additional BMI reductions, by a range of 1.19 kg/m^2^ (95% CI 0.83, 1.55) with DPP-4is to 1.96 kg/m^2^ (95% CI 1.57, 2.36) with GLP-1RAs; while no significant changes were observed with other add-ons. Across individual drugs, all GLP-1RAs and SGLT2is significantly reduced BMI, with reductions ranging from 1.39 kg/m^2^ (95% CI 0.73, 2.05) with dapagliflozin to 2.11 kg/m^2^ (95% CI 0.44, 3.78) with exenatide. By contrast, large differences existed within five DPP-4is, as only sitagliptin and vildagliptin yielded significant BMI decreases, in the order of 1.66 kg/m^2^ (95% CI 1.33, 1.99) and 0.82 kg/m^2^ (95% CI 0.26, 1.38) (Table 1; Supplementary Materials Figures S26–S32).

Amongst all dual therapies, GLP-1RAs+MET, AGIs+MET, and SGLT2is+MET reported significantly greater BMI reductions than INSs+MET, SUs+MET, and TZDs+MET, ranging from 1.08 kg/m^2^ (95% CI 0.27, 1.89) with SGLT2is+MET vs. INSs+MET to 1.90 kg/m^2^ (95% CI 0.66, 3.13) with GLP-1RAs+MET vs. TZDs+MET. Moreover, GLP-1RAs+MET were also favored over DPP-4is+MET, while DPP-4is+MET were favored over INSs+MET and SUs+MET (Table 2).

#### 3.2.5. Total Cholesterol

A total of 69 trials (7241 patients) provided data on TC. With the exception of NIDEs and SUs (no data available), the addition of other drug classes to MET all yielded significantly incremental TC reductions. The greatest reductions were detected with AGIs (1.08 mmol/L; 95% CI 0.78, 1.37), followed by GLP-1RAs (0.94 mmol/L; 95% CI 0.72, 1.15) and DPP-4is (0.71 mmol/L; 95% CI 0.57, 0.85). TZDs (0.38 mmol/L; 95% CI 0.13, 0.63) conferred the smallest reductions, although there were big intraclass differences, with a 0.61 mmol/L (95% CI 0.36, 0.87) reduction with pioglitazone vs. no significant reduction with rosiglitazone. Large differences were also found within SGLT2is, with a 0.54 mmol/L (95% CI 0.15, 0.93) reduction with dapagliflozin vs. no significant reduction with empagliflozin. Notably, all GLP-1RAs significantly reduced TC with relatively small intraclass difference, in the order of 0.90 mmol/L (95% CI 0.66, 1.15) with liraglutide and 1.11 mmol/L (95% CI 0.62, 1.59) with exenatide (Table 1; Supplementary Materials Figures S33–S38).

Among all dual therapies, AGIs+MET and GLP-1RAs+MET lowered TC to a significantly greater extent than SGLT2is+MET, INSs+MET, and TZDs+MET, with reductions ranging from 0.44 mmol/L (95% CI 0.01, 0.86) with GLP-1RAs+MET vs. SGLT2is+MET to 0.70 mmol/L (95% CI 0.31, 1.09) with AGIs+MET vs. TZDs+MET. Additionally, AGIs+MET were more efficacious than DPP-4is+MET, while DPP-4is+MET were more efficacious than TZDs+MET (Table 2).

#### 3.2.6. High-Density Lipoprotein-Cholesterol

A total of 54 trials (5272 patients) provided data on HDL-C. The addition of INSs, AGIs, DPP-4is, or GLP-1RAs to MET generated significantly incremental HDL-C increases, while other add-ons demonstrated no significant changes. The greatest increases were observed with INSs (0.40 mmol/L; 95% CI 0.16, 0.64), followed by AGIs (0.37 mmol/L; 95% CI 0.26, 0.47) and DPP-4is (0.32 mmol/L; 95% CI 0.21, 0.43). The smallest increases were evident with GLP-1RAs (0.11 mmol/L; 95% CI 0.01, 0.21), although big intraclass differences existed, with a 0.12 mmol/L (95% CI 0.01, 0.24) increase with liraglutide vs. no significant increase with exenatide. Within DPP-4is, there were also great differences, as only sitagliptin (0.41 mmol/L; 95% CI 0.26, 0.56) and alogliptin (0.40 mmol/L; 95% CI 0.28, 0.52) demonstrated significant HDL-C increases (Table 1; Supplementary Materials Figures S39–S45).

Across all dual therapies, INSs+MET, AGIs+MET, and DPP-4is+MET had significantly greater HDL-C increases than GLP-1RAs+MET and SUs+MET, ranging from 0.21 mmol/L (95% CI 0.06, 0.35) with DPP-4is+MET vs. GLP-1RAs+MET to 0.55 mmol/L (95% CI 0.23, 0.86) with INSs+MET vs. SUs+MET. Besides, GLP-1RAs+MET and SGLT2is+MET worked better than SUs+MET (Table 2).

#### 3.2.7. Systolic Blood Pressure

A total of 19 trials (1522 patients) provided data on SBP. As add-ons to MET, only SGLT2is (6.65 mmHg; 95% CI 4.13, 9.18) and GLP-1RAs (5.25 mmHg; 95% CI 2.33, 8.18) produced significantly additional SBP reductions, while no significant changes were observed with other add-ons. SGLT2is produced relatively larger reductions, in the order of 6.46 mmHg (95% CI 2.50, 10.42) with dapagliflozin and 6.61 mmHg (95% CI 2.06, 11.16) with empagliflozin. GLP-1RAs yielded smaller reductions, with a 5.77 mmHg (95% CI 2.69, 8.85) reduction with liraglutide vs. no significant reduction with exenatide. Of note, as a DPP-4i, sitagliptin also conferred a large SBP reduction of 12.12 mmHg (95% CI 6.15, 18.10) (Table 1; Supplementary Materials Figures S46–S50).

Across all dual therapies, no significant difference on SBP effect between all comparisons was detected (Table 2).

### 3.3. Adverse Events

A total of 204 trials (23,006 patients) provided data on hypoglycemia. GLP-1RAs (0.54; 95% CI 0.34, 0.85) and DPP-4is (0.77; 95% CI 0.61, 0.98) as add-ons to MET reported significantly lower hypoglycemia risks than MET alone, while no significant changes were noted in other add-ons (Table 1; Supplementary Materials Figures S51–S58). Amongst all dual therapies, only GLP-1RAs+MET were found to have significantly lower hypoglycemia risks than SUs+MET, with a RR of 0.41 (95% CI 0.18, 0.93) (Table 2).

A total of 27 trials (2621 patients) provided the data of DPP-4is (11 trials), TZDs (5 trials), AGIs (3 trials), SUs (3 trials), SGLT2is (3 trials), INSs (2 trials), and GLP-1RAs (1 trial) as add-ons to MET on renal function. A total of 26 of them reported that no renal function impairment occurred during the trials, with the remaining one reporting that glipizide added to MET did not have a significant impact on renal function compared with MET alone. In addition, three trials provided data of sitagliptin, vildagliptin, and liraglutide added to MET on lactic acidosis, all of which reported that no lactic acidosis occurred during the trials.

### 3.4. Mortality and Vascular Outcomes

Only two trials provided the mortality data of sitagliptin, liraglutide, and insulin glargine as add-ons to MET, reporting that no death occurred during the trials. Seven trials reported vascular outcomes, but without subdividing and detailing the events. One trial on miglitol and one trial on gliclazide reported that no cardiovascular events occurred during the trials. By contrast, one trial found that the addition of acarbose to MET demonstrated a 0.18 (95% CI 0.04, 0.77) times lower risk of vascular events than MET alone. Two trials reported that vildagliptin added to MET conferred a 0.16 (95% CI 0.04, 0.60) times lower risk of vascular events than MET alone. Whereas, two trials demonstrated that the addition of rosiglitazone to MET did not have significant impact on vascular events (Supplementary Materials Figures S59 and S60).

### 3.5. Sensitivity Analysis

Sensitivity analyses did not significantly change the overall results (Supplementary Materials Figures S61–S68).

## 4. Discussion

Ideally, the choice of a drug to add to MET requires a multi-factorial consideration that goes beyond glucose control, and encompasses a control of other risk factors and a beneficial effect on long-term outcomes. However, despite the wide use of MET-based dual therapies in China, conclusive evidence on mortality and vascular benefits is lacking. Given the limited data on long-term outcomes, we provided detailed comparative evidence on intermediate outcomes and common adverse events, which are cardiovascular risk factors and routinely monitored throughout diabetes care. Moreover, there are eight classes of add-on drugs with varying treatment effects, not only between drug classes but also between drugs of the same class. Thus, we reported efficacy and safety results across drug classes and individual drugs, and compared them against each other, to aid decision-makers in selecting therapy.

Evidences suggest that intensive glycemic control can reduce the risks of diabetes-related vascular events among patients in the early stage of diabetes [336,337]. This study demonstrated that in patients receiving MET alone, the addition of NIDEs produced the greatest additional HbA1c reductions, followed by SUs and GLP-1RA. The results generally supported the expert consensus on management algorithm for Chinese patients with type 2 diabetes [338], and the observed reductions were around the recommendation of Chinese clinical guideline [14]. However, the results were not fully consistent with the Sherifali (2010) study, which compared the HbA1c effect across six drug classes by synthesizing 61 English-language RCTs identified through May 2008, and found the max doses of SUs as having the greatest HbA1c effect, followed by TZDs when used as a monotherapy or added to other drugs [339]; and the Tsapas (2020) study, which compared the HbA1c effect across eight drug classes by synthesizing 296 English-language RCTs identified through December 2019, and found INSs and specific GLP-1 RAs added to MET-based therapy as having the greatest HbA1c reductions [340]. However, our results were not directly comparable to both studies, due to differences in the eligibility criteria used, such as the participants, the studied drugs, the comparisons, and the background therapies. In terms of plasma glucose, INS add-ons yielded both the largest additional FPG reductions and 2 hPG reductions, followed by NIDEs. However, across individual drugs, gliclazide, gliquidone, and voglibose generated the largest reductions on HbA1c, FPG, and 2 hPG, respectively. Moreover, HbA1c reduction with glipizide (0.86%) was more similar to TZDs (0.79%) than other SUs, such as gliclazide (1.70%). Large intraclass differences also existed regarding plasma glucose, such as FPG reductions of 1.01 mmol/L with glipizide vs. 2.20 mmol/L with gliquidone within SUs, and 2 hPG reductions of 1.27 mmol/L with miglitol vs. 3.29 mmol/L with voglibose within AGIs. These findings verified that drugs within the same class also demonstrated important differences. As such, optimal therapeutic decisions should also be guided by evidence on the comparative effects between individual drugs, especially when choosing from drugs belong to the same classes.

Obesity or overweight is common in type 2 diabetes patients, and weight is an important treatment-related outcome to patients [341,342]. Reducing body weight in obese or overweight type 2 diabetes patients can improve their quality of life and cardiovascular risk factors [343,344]. This study demonstrated that GLP-1RAs conferred the greatest BMI reductions, followed by AGIs and SGLT2is, which were all favored over INSs, SUs, and TZDs, when added to MET. The results roughly correspond to the findings of the Maruthur (2016) study, which compared the weight effect across five drug classes by synthesizing 48 English-language RCTs identified through March 2015, and found GLP-1RAs and SGLT2is added to MET conferred weight loss; however, the Maruthur study did not include AGIs, NIDEs, and INSs, and did not target Chinese patients [345]. The results also supported current clinical guidelines that favor either GLP-1RAs or SGLT2is for treating patients who need to promote weight loss, notably, the Chinese guideline, which suggests GLP-1RAs, SGLT2is, and AGIs can reduce weight for Chinese patients [3,14,346].

Dyslipidemia and hypertension are also common conditions coexisting with type 2 diabetes, which are routinely monitored and frequently addressed during treatment. A total of 72% of Chinese type 2 diabetes patients reported either dyslipidemia, hypertension, or both, and were 6 times more likely to develop cardiovascular diseases than those with diabetes alone [347]. It is of value to have an integrated management of them. This study demonstrated that the addition of AGIs, GLP-1RAs, DPP-4is, or INSs to MET had beneficial effects on both TC and HDL-C. AGI add-ons conferred the greatest TC reductions, followed by GLP-1RAs, which were favored over SGLT2is, INSs, and TZDs. INS add-ons had the biggest HDL-C increases, followed by AGIs, which were favored over SUs. Considerable intraclass differences in TC and HDL-C effect also existed, such as only pioglitazone demonstrated significant TC reduction within two TZDs, whilst only sitagliptin and alogliptin demonstrated significant HDL-C increases within five DPP-4is. The previous study reported that even a small SBP reduction of 2.4 mmHg lowered the risk of cardiovascular events in type 2 diabetes patients [348]. Since this study demonstrated that the addition of SGLT2is or GLP-1RAs to MET conferred additional SBP reductions by 6.65 and 5.25 mmHg, both classes might have beneficial effects on cardiovascular events. The results somewhat corroborated the findings of the Maruthur (2016) study, which compared the SBP effect of four drug classes by synthesizing 23 English-language RCTs, and found that SGLT2is and GLP-1RAs added to MET reduced SBP more than MET alone [345]; and the Tsapas (2021) study, which compared the SBP effect of 21 antidiabetic drugs by synthesizing 204 English-language RCTs, and found SGLT2is and GLP-1RAs were the most efficacious in reducing SBP compared with a placebo [349].

A patient-centered treatment approach should not only optimize efficacy, but also minimize adverse events and have beneficial effect on long-term outcomes [11]. Hypoglycemia is a commonly-observed adverse event in glucose-lowering drugs, which may impede treatment effect by suboptimal medication adherence, and can be life-threatening [2,11,350]. This study demonstrated that GLP-1RAs added to MET had the lowest hypoglycemia risk, and were favored over SUs, which were partly in accordance with previous findings [345,351]. Overall, amongst all add-on drug classes, only GLP-1RA add-ons produced a comprehensive beneficial effect on HbA1c, FPG, 2 hPG, BMI, TC, HDL-C, and SBP, while conferred a lower hypoglycemia risk. However, given the limited evidence, we could not reach a conclusion about the optimal therapies that have favorable profiles in terms of renal function, lactic acidosis, mortality, and vascular outcomes.

To our knowledge, only a few systematic reviews were found to compare several drug classes, but they were not targeted at Chinese patients, did not cover all available drugs, and only included English-language studies [339,340,345,349,351]. There is no comparative evidence on all available drugs, especially for Chinese patients. Therefore, our review had obvious strengths, in that it entailed a comprehensive search for all approved and used drugs for second-line treatment in China, by searching the English- and Chinese-language databases. It is the first attempt to systematically compare the advantages and disadvantages of these drugs as add-ons to MET for Chinese type 2 diabetes patients, by combining multiple clinical outcomes important to clinicians and patients. Although our review was not directly comparable to previous reviews because of differences in the study objective, study populations, and eligibility criteria used, our observations expanded the knowledge by supplementing with evidence from China.

Certain limitations should be acknowledged. First, systematic review and meta-analysis inevitably have inherent limitations, such as risk of the bias of trials, potential publication bias and clinical heterogeneity. Although methodological controversies of indirect comparison remained, the adjusted indirect treatment comparison based on Bucher method can partially take into account the baseline risk and other prognostic factors of participants in different trials, and maintain certain strengths of randomized allocation in the original trials when estimating effect size [16,352]. Second, for reasons of practicality and to facilitate the interpretation and applicability of the results in clinical practice, we combined all approved doses of each drug into a single treatment node; thus, our results do not allow one to draw conclusions regarding the varying effects amongst different doses of the same drug. Third, most RCTs had a short study duration, with limited ability to assess long-term outcomes; thus, the generalizability of our results may be limited due to the absence of long-term evidence. Fourth, for several drug classes, not all targeted drugs were covered (e.g., for three targeted NIDEs, only studies for repaglinide were found), which may not represent the whole class, and not all RCTs reported all targeted outcomes, thereby affecting the reliability of their respective quantitative estimates. Fifth, this review focused on adults with type 2 diabetes, while MET in patients aged 10 to 16 years was proven by several studies an efficacy and tolerability similar to that in adults; thus, future study may pay attention to this population. Lastly, newer studies, such as those published in 2021, were not analyzed in this review, which may introduce a possible bias in the analysis.

## 5. Conclusions

The choice of a glucose-lowering drug added to MET should be tailored, depend on drug-specific effects and individualized patient characteristics and preferences. Our results provided a unified hierarchy of evidence for caring for type 2 diabetes and suggested a potential treatment hierarchy for clinicians and patients in China, with GLP-1RA add-ons being the most preferred based on their favorable combined efficacy and safety profiles; NIDEs or SUs suggested for patients whose HbA1c is far from the target; INSs or NIDEs suggested for patients whose FPG or 2 hPG is out of control; GLP-1RAs or AGIs suggested for patients for whom BMI or TC management is a priority; INSs or AGIs suggested for patients for whom HDL-C control is an emphasis; SGLT2is or GLP-1RAs suggested for patients having a need to lower SBP; and GLP-1RAs or DPP-4is suggested for patients wishing to minimize hypoglycemia. Additionally, our results corroborated the intraclass differences among therapies. Of course, these results may incorporate recent findings in cardiovascular outcomes in foreign populations.

From an overall point of view, long-term treatment costs across therapies should be considered alongside their clinical outcomes, by means of country-specific cost-effectiveness analyses. This study also provided systematic and comparative clinical data for conducting cost-effectiveness studies, which will add cost evidence for the rational choice of therapies and improving the allocation of healthcare resources. In a future study, we intend to use these data to conduct a cost-effectiveness study of these therapies.

## Figures and Tables

**Table 1:** Treatment effects of glucose-lowering drugs added to metformin compared with metformin monotherapy.

Therapy	HbA1c, %	FPG, mmol/L	2 hPG, mmol/L	BMI, kg/m^2^	TC, mmol/L	HDL-C, mmol/L	SBP, mmhg	Hypoglycemia
TZD	−0.79 [−0.99, −0.60] *	−1.04 [−1.24, −0.84] *	−1.71 [−2.00, −1.42] *	−0.07 [−1.24, 1.10]	−0.38 [−0.63, −0.13] *	0.18 [−0.39, 0.75]	—	1.16 [0.60, 2.22]
	*I*^2^ = *90.9*%	*I*^2^ = *86.5*%	*I*^2^ = *84.5*%	*I*^2^ = *93.4*%	*I*^2^ = *81.7*%	*I*^2^ = *98.3*%		*I*^2^ = *0.0*%
Rosiglitazone	−0.73 [−1.03, −0.42] *	−1.11 [−1.39, −0.83] *	−1.75 [−2.40, −1.10] *	−1.23 [−2.51, 0.06]	−0.03 [−0.21, 0.15]	—	—	1.46 [0.51, 4.18]
Pioglitazone	−0.83 [−1.07, −0.58] *	−1.03 [−1.27, −0.78] *	−1.66 [−1.96, −1.36] *	0.51 [−0.49, 1.51]	−0.61 [−0.87, −0.36] *	0.18 [−0.39, 0.75]	—	1.00 [0.43, 2.30]
DPP-4i	−0.99 [−1.09, −0.89] *	−1.10 [−1.20, −1.00] *	−1.76 [−1.91, −1.61] *	−1.19 [−1.55, −0.83] *	−0.71 [−0.85, −0.57] *	0.32 [0.21, 0.43] *	−5.40 [−10.98, 0.18]	0.77 [0.61, 0.98] *
	*I*^2^ = *80.5*%	*I*^2^ = *73.1*%	*I*^2^ = *70.9*%	*I*^2^ = *92.3*%	*I*^2^ = *79.9*%	*I*^2^ = *93.2*%	*I*^2^ = *89.2*%	*I*^2^ = *0.0*%
Sitagliptin	−1.08 [−1.24, −0.93] *	−1.26 [−1.41, −1.11] *	−2.03 [−2.22, −1.83] *	−1.66 [−1.99, −1.33] *	−0.90 [−1.12, −0.67] *	0.41 [0.26, 0.56] *	−12.12 [−18.10, −6.15] *	0.74 [0.55, 1.01]
Saxagliptin	−0.80 [−0.96, −0.63] *	−0.91 [−1.12, −0.69] *	−1.44 [−1.70, −1.17] *	−0.41 [−0.87, 0.05]	−0.48 [−0.89, −0.07] *	0.01 [−0.11, 0.13]	−1.20 [−7.65, 5.25]	0.98 [0.56, 1.72]
Vildagliptin	−0.76 [−0.89, −0.63] *	−0.85 [−1.05, −0.66] *	−1.20 [−1.66, −0.73] *	−0.82 [−1.38, −0.26] *	−0.33 [−0.49, −0.16] *	0.18 [−0.14, 0.49]	0.14 [−10.11, 10.39]	0.65 [0.34, 1.27]
Linagliptin	−0.90 [−1.17, −0.63] *	−1.15 [−1.47, −0.83] *	−1.97 [−2.37, −1.58] *	−2.11 [−4.60, 0.39]	−0.87 [−1.86, 0.13]	0.25 [−0.25, 0.75]	−4.00 [−8.56, 0.56]	0.72 [0.28, 1.87]
Alogliptin	−1.35 [−1.90, −0.80] *	−1.08 [−1.33, −0.82] *	−1.31 [−1.86, −0.77] *	−0.43 [−2.68, 1.82]	−0.63 [−0.72, −0.53] *	0.40 [0.28, 0.52] *	−0.29 [−4.24, 3.66]	1.00 [0.06, 15.73]
AGI	−1.03 [−1.25, −0.80] *	−0.97 [−1.12, −0.82] *	−1.65 [−1.92, −1.39] *	−1.75 [−2.48, −1.02] *	−1.08 [−1.37, −0.78] *	0.37 [0.26, 0.47] *	−3.82 [−8.12, 0.47]	0.76 [0.46, 1.26]
	*I*^2^ = *90.1*%	*I*^2^ = *66.6*%	*I*^2^ = *76.0*%	*I*^2^ = *0.0*%	*I*^2^ = *87.3*%	*I*^2^ = *78.8*%	*I*^2^ = *41.1*%	*I*^2^ = *0.0*%
Acarbose	−1.03 [−1.26, −0.79] *	−0.99 [−1.15, −0.83] *	−1.63 [−1.90, −1.36] *	−1.75 [−2.48, −1.02] *	−1.08 [−1.37, −0.78] *	0.37 [0.26, 0.47] *	−3.82 [−8.12, 0.47]	0.75 [0.44, 1.26]
Voglibose	−1.15 [−1.96, −0.34] *	−1.33 [−2.04, −0.62] *	−3.29 [−4.39, −2.19] *	—	—	—	—	1.00 [0.02, 49.17]
Miglitol	−1.04 [−1.97, −0.11] *	−0.48 [−0.98, 0.02]	−1.27 [−2.13, −0.40] *	—	—	—	—	1.00 [0.11, 9.47]
SGLT2i	−1.07 [−1.43, −0.71] *	−0.87 [−1.11, −0.62] *	−1.44 [−1.81, −1.07] *	−1.52 [−2.06, −0.98] *	−0.50 [−0.87, −0.13] *	0.21 [−0.03, 0.46]	−6.65 [−9.18, −4.13] *	1.00 [0.38, 2.64]
	*I*^2^ = *86.6*%	*I*^2^ = *34.1*%	*I*^2^ = *39.7*%	*I*^2^ = *39.1*%	*I*^2^ = *79.0*%	*I*^2^ = *87.2*%	*I*^2^ = *0.0*%	*I*^2^ = *0.0*%
Dapagliflozin	−1.02 [−1.45, −0.59] *	−0.84 [−1.11, −0.57] *	−1.38 [−1.78, −0.98] *	−1.39 [−2.05, −0.73] *	−0.54 [−0.93, −0.15] *	0.23 [−0.04, 0.51]	−6.46 [−10.42, −2.50] *	1.00 [0.34, 2.94]
Empagliflozin	−1.28 [−1.83, −0.73] *	−1.22 [−1.97, −0.47] *	−1.88 [−2.97, −0.79] *	−1.90 [−2.55, −1.25] *	−0.10 [−1.08, 0.88]	0.10 [−0.36, 0.56]	−6.61 [−11.16, −2.06] *	1.00 [0.11, 9.47]
INS	−1.12 [−1.42, −0.82] *	−1.58 [−1.94, −1.22] *	−2.52 [−3.20, −1.83] *	−0.45 [−1.05, 0.16]	−0.48 [−0.70, −0.27] *	0.40 [0.16, 0.64] *	—	0.58 [0.30, 1.10]
	*I*^2^ = *83.9*%	*I*^2^ = *77.9*%	*I*^2^ = *88.5*%	*I*^2^ = *0.0*%	*I*^2^ = *0.0*%	*I*^2^ = *0.0*%		*I*^2^ = *20.7*%
Insulin	−1.12 [−1.42, −0.82] *	−1.58 [−1.94, −1.22] *	−2.52 [−3.20, −1.83] *	−0.45 [−1.05, 0.16]	−0.48 [−0.70, −0.27] *	0.40 [0.16, 0.64] *	—	0.58 [0.30, 1.10]
GLP-1RA	−1.14 [−1.28, −1.01] *	−1.33 [−1.53, −1.14] *	−1.68 [−1.94, −1.42] *	−1.96 [−2.36, −1.57] *	−0.94 [−1.15, −0.72] *	0.11 [0.01, 0.21] *	−5.25 [−8.18, −2.33] *	0.54 [0.34, 0.85] *
	*I*^2^ = *71.0*%	*I*^2^ = *82.0*%	*I*^2^ = *49.7*%	*I*^2^ = *80.9*%	*I*^2^ = *62.7*%	*I*^2^ = *87.0*%	*I*^2^ = *0.0*%	*I*^2^ = *0.0*%
Exenatide	−0.74 [−0.94, −0.55] *	−1.26 [−1.66, −0.87] *	−1.59 [−2.45, −0.73] *	−2.11 [−3.78, −0.44] *	−1.11 [−1.59, −0.62] *	0.04 [−0.06, 0.15]	−0.40 [−9.83, 9.03]	1.00 [0.11, 9.48]
Liraglutide	−1.21 [−1.36, −1.06] *	−1.35 [−1.57, −1.14] *	−1.70 [−1.98, −1.42] *	−1.97 [−2.36, −1.58] *	−0.90 [−1.15, −0.66] *	0.12 [0.01, 0.24] *	−5.77 [−8.85, −2.69] *	0.53 [0.33, 0.84] *
SU	−1.16 [−1.43, −0.90] *	−1.32 [−1.66, −0.98] *	−1.90 [−2.33, −1.48] *	−0.24 [−0.99, 0.51]	—	−0.14 [−0.34, 0.05]	−3.04 [−6.98, 0.89]	1.31 [0.67, 2.58]
	*I*^2^ = *88.3*%	*I*^2^ = *91.0*%	*I*^2^ = *83.4*%	*I*^2^ = *46.2*%		*I*^2^ = *0.0*%	*I*^2^ = *0.0*%	*I*^2^ = *0.0*%
Glimepiride	−1.14 [−1.55, −0.74] *	−1.24 [−1.72, −0.76] *	−1.81 [−2.45, −1.16] *	−0.15 [−2.08, 1.79]	—	—	—	1.49 [0.54, 4.10]
Gliclazide	−1.70 [−2.72, −0.68] *	−1.88 [−3.03, −0.72] *	−1.70 [−2.76, −0.65] *	−0.10 [−0.67, 0.47]	—	−0.16 [−0.37, 0.05]	−1.90 [−7.19, 3.39]	0.74 [0.14, 3.83]
Glipizide	−0.86 [−1.13, −0.59] *	−1.01 [−1.38, −0.64] *	−2.07 [−2.48, −1.66] *	−0.80 [−2.65, 1.05]	—	—	—	1.51 [0.34, 6.77]
Gliquidone	−1.52 [−1.84, −1.20] *	−2.20 [−2.62, −1.78] *	−2.97 [−3.64, −2.30] *	—	—	0.00 [−0.64, 0.64]	−4.45 [−10.33, 1.43]	0.74 [0.02, 35.42]
NIDE	−1.29 [−1.60, −0.97] *	−1.43 [−1.83, −1.03] *	−1.90 [−2.47, −1.34] *	—	—	—	—	0.85 [0.28, 2.59]
	*I*^2^ = *80.0*%	*I*^2^ = *75.1*%	*I*^2^ = *81.6*%					*I*^2^ = *0.0*%
Repaglinide	−1.29 [−1.60, −0.97] *	−1.43 [−1.83, −1.03] *	−1.90 [−2.47, −1.34] *	—	—	—	—	0.85 [0.28, 2.59]

For HbA1c, FPG, 2 hPG, BMI, TC, HDL-C, and SBP, data are WMDs and 95% CIs; for hypoglycemia, data are RRs and 95% CIs. * Statistically significant differences. Table 1 were summary results from Supplementary Materials Figures S2–S58. A 2 hPG, 2 h postprandial plasma glucose. AGI, α-glucosidase inhibitor. BMI, body mass index. CI, confidence interval. DPP-4i, dipeptidyl peptidase 4 inhibitor. FPG, fasting plasma glucose. GLP-1RA, glucagon-like peptide-1 receptor agonist. HbA1c, hemoglobin Alc. HDL-C, high density lipoprotein-cholesterol. INS, insulin. MET, metformin. NIDE, glinide. RR, risk ratio. SBP, systolic blood pressure. SGLT2i, sodium-glucose cotransporter 2 inhibitor. SU, sulfonylurea. TC, total cholesterol. TZD, thiazolidinedione. WMD, weighted mean difference.

**Table 2:** Treatment effects of glucose-lowering drugs added to metformin compared with each other.

**HbA1c, %** (**Left Lower Half**)						**FPG, mmol/L** (**Right Upper Half**)	
TZD+MET	0.06 [−0.16, 0.28]	−0.07 [−0.32, 0.18]	−0.17 [−0.49, 0.14]	0.54 [0.13, 0.95] *	0.30 [0.02, 0.57] *	0.28 [−0.11, 0.67]	0.39 [−0.05, 0.84]
0.19 [−0.03, 0.41]	DPP-4i+MET	−0.13 [−0.31, 0.05]	−0.24 [−0.50, 0.03]	0.48 [0.10, 0.85] *	0.23 [0.02, 0.45] *	0.22 [−0.13, 0.57]	0.33 [−0.08, 0.74]
0.23 [−0.07, 0.53]	0.04 [−0.21, 0.28]	AGI+MET	−0.10 [−0.39, 0.19]	0.61 [0.22, 1.00] *	0.37 [0.12, 0.62] *	0.36 [−0.02, 0.73]	0.46 [0.04, 0.89] *
0.27 [−0.14, 0.68]	0.08 [−0.30, 0.45]	0.04 [−0.39, 0.47]	SGLT2i+MET	0.71 [0.28, 1.15] *	0.47 [0.15, 0.78] *	0.46 [0.04, 0.88] *	0.56 [0.10, 1.03] *
0.33 [−0.03, 0.69]	0.13 [−0.18, 0.45]	0.10 [−0.28, 0.47]	0.06 [−0.41, 0.53]	INS+MET	−0.24 [−0.65, 0.17]	−0.25 [−0.75, 0.24]	−0.15 [−0.68, 0.39]
0.35 [0.11, 0.59] *	0.16 [−0.01, 0.32]	0.12 [−0.15, 0.38]	0.08 [−0.31, 0.46]	0.02 [−0.31, 0.35]	GLP-1RA+MET	−0.01 [−0.40, 0.38]	0.10 [−0.35, 0.54]
0.37 [0.04, 0.70] *	0.18 [−0.11, 0.46]	0.14 [−0.21, 0.49]	0.10 [−0.35, 0.55]	0.04 [−0.36, 0.44]	0.02 [−0.28, 0.32]	SU+MET	0.11 [−0.42, 0.63]
0.49 [0.12, 0.86] *	0.30 [−0.03, 0.63]	0.26 [−0.13, 0.65]	0.22 [−0.26, 0.70]	0.16 [−0.27, 0.60]	0.14 [−0.20, 0.48]	0.12 [−0.29, 0.54]	NIDE+MET
**2 hPG, mmol/L** (**Left Lower Half**)						**BMI, kg/m^2^** (**Right Upper Half**)	
TZD+MET	1.13 [−0.10, 2.35]	1.68 [0.31, 3.06] *	1.46 [0.17, 2.74] *	0.38 [-0.93, 1.70]	1.90 [0.66, 3.13] *	0.17 [−1.22, 1.56]	—
0.05 [−0.28, 0.38]	DPP-4i+MET	0.56 [−0.26, 1.37]	0.33 [−0.32, 0.98]	−0.75 [−1.45, −0.04] *	0.77 [0.24, 1.30] *	−0.95 [−1.79, −0.12] *	—
−0.06 [−0.45, 0.34]	−0.10 [−0.41, 0.20]	AGI+MET	−0.23 [−1.13, 0.68]	−1.30 [−2.25, −0.35] *	0.21 [−0.62, 1.04]	−1.51 [−2.56, −0.46] *	—
−0.27 [−0.74, 0.20]	−0.32 [−0.72, 0.08]	−0.22 [−0.67, 0.24]	SGLT2i+MET	−1.08 [−1.89, −0.27] *	0.44 [−0.23, 1.11]	−1.28 [−2.21, −0.36] *	—
0.81 [0.07, 1.55] *	0.76 [0.06, 1.46] *	0.86 [0.13, 1.59] *	1.08 [0.30, 1.86] *	INS+MET	1.51 [0.79, 2.24] *	−0.21 [−1.17, 0.76]	—
−0.03 [−0.42, 0.37]	−0.08 [−0.38, 0.23]	0.03 [−0.34, 0.40]	0.25 [−0.21, 0.70]	−0.83 [−1.56, −0.10] *	GLP-1RA+MET	−1.72 [−2.57, −0.87] *	—
0.19 [−0.32, 0.71]	0.14 [−0.31, 0.59]	0.25 [−0.25, 0.75]	0.46 [−0.10, 1.03]	−0.62 [−1.42, 0.19]	0.22 [−0.28, 0.72]	SU+MET	—
0.20 [−0.44, 0.83]	0.15 [−0.44, 0.73]	0.25 [−0.37, 0.87]	0.47 [−0.21, 1.14]	−0.61 [−1.50, 0.27]	0.22 [−0.40, 0.84]	0.00 [−0.70, 0.71]	NIDE+MET
**TC, mmol/L** (**Left Lower Half**)						**HDL**-**C, mmol/L** (**Right Upper Half**)	
TZD+MET	−0.14 [−0.72, 0.44]	−0.19 [−0.77, 0.39]	−0.03 [−0.66, 0.59]	−0.22 [−0.84, 0.40]	0.07 [−0.51, 0.65]	0.33 [−0.28, 0.93]	—
0.33 [0.04, 0.62] *	DPP-4i+MET	−0.05 [−0.20, 0.10]	0.11 [−0.17, 0.38]	−0.08 [−0.34, 0.18]	0.21 [0.06, 0.35] *	0.46 [0.24, 0.69] *	—
0.70 [0.31, 1.09] *	0.37 [0.04, 0.70] *	AGI+MET	0.16 [−0.11, 0.42]	−0.03 [−0.29, 0.23]	0.26 [0.11, 0.40] *	0.51 [0.29, 0.74] *	—
0.12 [−0.32, 0.57]	−0.21 [−0.60, 0.19]	−0.58 [−1.05, −0.11] *	SGLT2i+MET	−0.19 [−0.53, 0.16]	0.10 [−0.16, 0.37]	0.36 [0.04, 0.68] *	—
0.11 [−0.23, 0.44]	−0.22 [−0.48, 0.03]	−0.59 [−0.96, −0.23] *	−0.02 [−0.44, 0.41]	INS+MET	0.29 [0.03, 0.55] *	0.55 [0.23, 0.86] *	—
0.56 [0.23, 0.89] *	0.23 [−0.03, 0.49]	−0.14 [−0.51, 0.23]	0.44 [0.01, 0.86] *	0.46 [0.15, 0.76] *	GLP-1RA+MET	0.26 [0.04, 0.48] *	—
—	—	—	—	—	—	SU+MET	—
—	—	—	—	—	—	—	NIDE+MET
**SBP, mmHg** (**Left Lower Half**)						**Hypoglycemia** (**Right Upper Half**)	
TZD+MET	1.50 [0.75, 3.01]	1.52 [0.67, 3.46]	1.16 [0.36, 3.72]	2.01 [0.80, 5.01]	2.13 [0.96, 4.72]	0.88 [0.35, 2.26]	1.36 [0.38, 4.95]
—	DPP-4i+MET	1.01 [0.58, 1.76]	0.77 [0.28, 2.09]	1.34 [0.67, 2.65]	1.42 [0.85, 2.38]	0.59 [0.29, 1.21]	0.91 [0.29, 2.83]
—	−1.57 [−8.62, 5.47]	AGI+MET	0.76 [0.26, 2.27]	1.32 [0.58, 2.99]	1.41 [0.71, 2.77]	0.58 [0.25, 1.35]	0.90 [0.26, 3.04]
—	1.26 [−4.87, 7.38]	2.83 [−2.16, 7.82]	SGLT2i+MET	1.74 [0.54, 5.57]	1.85 [0.63, 5.40]	0.76 [0.23, 2.50]	1.18 [0.27, 5.16]
—	—	—	—	INS+MET	1.06 [0.48, 2.34]	0.44 [0.17, 1.12]	0.68 [0.19, 2.46]
—	−0.14 [−6.45, 6.16]	1.43 [−3.77, 6.63]	−1.40 [−5.27, 2.47]	—	GLP-1RA+MET	0.41 [0.18, 0.93] *	0.64 [0.19, 2.12]
—	−2.36 [−9.19, 4.47]	−0.78 [−6.61, 5.04]	−3.61 [−8.29, 1.06]	—	−2.21 [−7.12, 2.69]	SU+MET	1.54 [0.42, 5.68]
—	—	—	—	—	—	—	NIDE+MET

Treatment estimates are WMDs and 95% CIs of the column-defining treatment compared with the row-defining treatment for HbA1c, 2 hPG, TC, and SBP (left lower half), and WMDs > 0 favor the row-defining treatment. Treatment estimates are WMDs and 95% CIs (or RRs and 95% CIs) of the row-defining treatment compared with the column-defining treatment for FPG, BMI, and HDL-C (or hypoglycemia) (right upper half), and WMDs > 0 (or RRs > 1) favor the column-defining treatment. * Statistically significant differences. A 2 hPG, 2 h postprandial plasma glucose. AGI, α-glucosidase inhibitor. BMI, body mass index. CI, confidence interval. DPP-4i, dipeptidyl peptidase 4 inhibitor. FPG, fasting plasma glucose. GLP-1RA, glucagon-like peptide-1 receptor agonist. HbA1c, hemoglobin Alc. HDL-C, high density lipoprotein-cholesterol. INS, insulin. MET, metformin. NIDE, glinide. RR, risk ratio. SBP, systolic blood pressure. SGLT2i, sodium-glucose cotransporter 2 inhibitor. SU, sulfonylurea. TC, total cholesterol. TZD, thiazolidinedione. WMD, weighted mean difference.

## Data Availability

The datasets used and/or analyzed during the current study are available from the corresponding authors on reasonable request.

## Supplementary Materials

The following supporting information can be downloaded at: https://www.mdpi.com/article/10.3390/jcm11185435/s1. Table S1. Search terms and search strategies; Table S2. Eligibility criteria; Table S3. Characteristics and risk of bias of the included trials; Figure S1. Risk of publication bias across studies; Figure S2. Meta-analysis results for change in hemoglobin Alc (%) of thiazolidinediones added to metformin compared with metformin monotherapy; Figure S3. Meta-analysis results for change in hemoglobin Alc (%) of dipeptidyl peptidase 4 inhibitors added to metformin compared with metformin monotherapy; Figure S4. Meta-analysis results for change in hemoglobin Alc (%) of α-glucosidase inhibitors added to metformin compared with metformin monotherapy; Figure S5. Meta-analysis results for change in hemoglobin Alc (%) of sodium-glucose cotransporter 2 inhibitors added to metformin compared with metformin monotherapy; Figure S6. Meta-analysis results for change in hemoglobin Alc (%) of insulins added to metformin compared with metformin monotherapy; Figure S7. Meta-analysis results for change in hemoglobin Alc (%) of glucagon-like peptide-1 receptor agonists added to metformin compared with metformin monotherapy; Figure S8. Meta-analysis results for change in hemoglobin Alc (%) of sulfonylureas added to metformin compared with metformin monotherapy; Figure S9. Meta-analysis results for change in hemoglobin Alc (%) of glinides added to metformin compared with metformin monotherapy; Figure S10. Meta-analysis results for change in fasting plasma glucose (mmol/L) of thiazolidinediones added to metformin compared with metformin monotherapy; Figure S11. Meta-analysis results for change in fasting plasma glucose (mmol/L) of dipeptidyl peptidase 4 inhibitors added to metformin compared with metformin monotherapy; Figure S12. Meta-analysis results for change in fasting plasma glucose (mmol/L) of α-glucosidase inhibitors added to metformin compared with metformin monotherapy; Figure S13. Meta-analysis results for change in fasting plasma glucose (mmol/L) of sodium-glucose cotransporter 2 inhibitors added to metformin compared with metformin monotherapy; Figure S14. Meta-analysis results for change in fasting plasma glucose (mmol/L) of insulins added to metformin compared with metformin monotherapy; Figure S15. Meta-analysis results for change in fasting plasma glucose (mmol/L) of glucagon-like peptide-1 receptor agonists added to metformin compared with metformin monotherapy; Figure S16. Meta-analysis results for change in fasting plasma glucose (mmol/L) of sulfonylureas added to metformin compared with metformin monotherapy; Figure S17. Meta-analysis results for change in fasting plasma glucose (mmol/L) of glinides added to metformin compared with metformin monotherapy; Figure S18. Meta-analysis results for change in 2 h postprandial plasma glucose (mmol/L) of thiazolidinediones added to metformin compared with metformin monotherapy; Figure S19. Meta-analysis results for change in 2 h postprandial plasma glucose (mmol/L) of dipeptidyl peptidase 4 inhibitors added to metformin compared with metformin monotherapy; Figure S20. Meta-analysis results for change in 2 h postprandial plasma glucose (mmol/L) of α-glucosidase inhibitors added to metformin compared with metformin monotherapy; Figure S21. Meta-analysis results for change in 2 h postprandial plasma glucose (mmol/L) of sodium-glucose cotransporter 2 inhibitors added to metformin compared with metformin monotherapy; Figure S22. Meta-analysis results for change in 2 h postprandial plasma glucose (mmol/L) of insulins added to metformin compared with metformin monotherapy; Figure S23. Meta-analysis results for change in 2 h postprandial plasma glucose (mmol/L) of glucagon-like peptide-1 receptor agonists added to metformin compared with metformin monotherapy; Figure S24. Meta-analysis results for change in 2 h postprandial plasma glucose (mmol/L) of sulfonylureas added to metformin compared with metformin monotherapy; Figure S25. Meta-analysis results for change in 2 h postprandial plasma glucose (mmol/L) of glinides added to metformin compared with metformin monotherapy; Figure S26. Meta-analysis results for change in body mass index (kg/m^2^) of thiazolidinediones added to metformin compared with metformin monotherapy; Figure S27. Meta-analysis results for change in body mass index (kg/m^2^) of dipeptidyl peptidase 4 inhibitors added to metformin compared with metformin monotherapy; Figure S28. Meta-analysis results for change in body mass index (kg/m^2^) of α-glucosidase inhibitors added to metformin compared with metformin monotherapy; Figure S29. Meta-analysis results for change in body mass index (kg/m^2^) of sodium-glucose cotransporter 2 inhibitors added to metformin compared with metformin monotherapy; Figure S30. Meta-analysis results for change in body mass index (kg/m^2^) of insulins added to metformin compared with metformin monotherapy; Figure S31. Meta-analysis results for change in body mass index (kg/m^2^) of glucagon-like peptide-1 receptor agonists added to metformin compared with metformin monotherapy; Figure S32. Meta-analysis results for change in body mass index (kg/m^2^) of sulfonylureas added to metformin compared with metformin monotherapy; Figure S33. Meta-analysis results for change in total cholesterol (mmol/L) of thiazolidinediones added to metformin compared with metformin monotherapy; Figure S34. Meta-analysis results for change in total cholesterol (mmol/L) of dipeptidyl peptidase 4 inhibitors added to metformin compared with metformin monotherapy; Figure S35. Meta-analysis results for change in total cholesterol (mmol/L) of α-glucosidase inhibitors added to metformin compared with metformin monotherapy; Figure S36. Meta-analysis results for change in total cholesterol (mmol/L) of sodium-glucose cotransporter 2 inhibitors added to metformin compared with metformin monotherapy; Figure S37. Meta-analysis results for change in total cholesterol (mmol/L) of insulins added to metformin compared with metformin monotherapy; Figure S38. Meta-analysis results for change in total cholesterol (mmol/L) of glucagon-like peptide-1 receptor agonists added to metformin compared with metformin monotherapy; Figure S39. Meta-analysis results for change in high density lipoprotein-cholesterol (mmol/L) of thiazolidinediones added to metformin compared with metformin monotherapy; Figure S40. Meta-analysis results for change in high density lipoprotein-cholesterol (mmol/L) of dipeptidyl peptidase 4 inhibitors added to metformin compared with metformin monotherapy; Figure S41. Meta-analysis results for change in high density lipoprotein-cholesterol (mmol/L) of α-glucosidase inhibitors added to metformin compared with metformin monotherapy; Figure S42. Meta-analysis results for change in high density lipoprotein-cholesterol (mmol/L) of sodium-glucose cotransporter 2 inhibitors added to metformin compared with metformin monotherapy; Figure S43. Meta-analysis results for change in high density lipoprotein-cholesterol (mmol/L) of insulins added to metformin compared with metformin monotherapy; Figure S44. Meta-analysis results for change in high density lipoprotein-cholesterol (mmol/L) of glucagon-like peptide-1 receptor agonists added to metformin compared with metformin monotherapy; Figure S45. Meta-analysis results for change in high density lipoprotein-cholesterol (mmol/L) of sulfonylureas added to metformin compared with metformin monotherapy; Figure S46. Meta-analysis results for change in systolic blood pressure (mmHg) of dipeptidyl peptidase 4 inhibitors added to metformin compared with metformin monotherapy; Figure S47. Meta-analysis results for change in systolic blood pressure (mmHg) of α-glucosidase inhibitors added to metformin compared with metformin monotherapy; Figure S48. Meta-analysis results for change in systolic blood pressure (mmHg) of sodium-glucose cotransporter 2 inhibitors added to metformin compared with metformin monotherapy; Figure S49. Meta-analysis results for change in systolic blood pressure (mmHg) of glucagon-like peptide-1 receptor agonists added to metformin compared with metformin monotherapy; Figure S50. Meta-analysis results for change in systolic blood pressure (mmHg) of sulfonylureas added to metformin compared with metformin monotherapy; Figure S51. Meta-analysis results for incidence of hypoglycemia of thiazolidinediones added to metformin compared with metformin monotherapy; Figure S52. Meta-analysis results for incidence of hypoglycemia of dipeptidyl peptidase 4 inhibitors added to metformin compared with metformin monotherapy; Figure S53. Meta-analysis results for incidence of hypoglycemia of α-glucosidase inhibitors added to metformin compared with metformin monotherapy; Figure S54. Meta-analysis results for incidence of hypoglycemia of sodium-glucose cotransporter 2 inhibitors added to metformin compared with metformin monotherapy; Figure S55. Meta-analysis results for incidence of hypoglycemia of insulins added to metformin compared with metformin monotherapy; Figure S56. Meta-analysis results for incidence of hypoglycemia of glucagon-like peptide-1 receptor agonists added to metformin compared with metformin monotherapy; Figure S57. Meta-analysis results for incidence of hypoglycemia of sulfonylureas added to metformin compared with metformin monotherapy; Figure S58. Meta-analysis results for incidence of hypoglycemia of glinides added to metformin compared with metformin monotherapy; Figure S59. Meta-analysis results for incidence of complications of vildagliptin added to metformin compared with metformin monotherapy; Figure S60. Meta-analysis results for incidence of complications of rosiglitazone added to metformin compared with metformin monotherapy; Figure S61. Sensitivity analysis results for change in hemoglobin Alc (%) of glucose-lowering drugs added to metformin compared with metformin monotherapy; Figure S62. Sensitivity analysis results for change in fasting plasma glucose (mmol/L) of glucose-lowering drugs added to metformin compared with metformin monotherapy; Figure S63. Sensitivity analysis results for change in 2 h postprandial plasma glucose (mmol/L) of glucose-lowering drugs added to metformin compared with metformin monotherapy; Figure S64. Sensitivity analysis results for change in body mass index (kg/m^2^) of glucose-lowering drugs added to metformin compared with metformin monotherapy; Figure S65. Sensitivity analysis results for change in total cholesterol (mmol/L) of glucose-lowering drugs added to metformin compared with metformin monotherapy; Figure S66. Sensitivity analysis results for change in high density lipoprotein-cholesterol (mmol/L) of glucose-lowering drugs added to metformin compared with metformin monotherapy; Figure S67. Sensitivity analysis results for change in systolic blood pressure (mmHg) of glucose-lowering drugs added to metformin compared with metformin monotherapy; Figure S68. Sensitivity analysis results for incidence of hypoglycemia of glucose-lowering drugs added to metformin compared with metformin monotherapy.

## Abbreviations

2 hPG: 2 h postprandial plasma glucose; AGI: α-glucosidase inhibitor; BMI: body mass index; CI: confidence interval; CNKI: China National Knowledge Infrastructure; DPP-4i: dipeptidyl peptidase 4 inhibitor; FPG: fasting plasma glucose; GLP-1RA: glucagon-like peptide-1 receptor agonist; HbA1c: hemoglobin Alc; HDL-C: high density lipoprotein-cholesterol; INS: insulin; MET: metformin; NIDE: glinide; RCT: randomized controlled trial; RR: risk ratio; SBP: systolic blood pressure; SGLT2i: sodium-glucose cotransporter 2 inhibitor; SU: sulfonylurea; TC: total cholesterol; TZD: thiazolidinedione; WMD: weighted mean difference.
