# Supplementary material for: Comparison of Glucose-Lowering Drugs as Second-Line Treatment for Type 2 Diabetes: A Systematic Review and Meta-Analysis
Source: J Clin Med. 2022 Sep 16;11(18):5435. doi: 10.3390/jcm11185435 (PMC9504435; doi:10.3390/jcm11185435)
Supplement: Supplementary file 1 [file jcm-11-05435-s001.zip › jcm-1852568-supplementary.pdf]

Tables

Table S1. Search terms and search strategies

**Database 1: PubMed**		
Advanced Search	Query	Results
Search strategy	(((((Type 2 diabetes) OR (Non insulin dependent diabetes mellitus)) AND (((((((((((((((((((((((((((((((((((Metformin) OR (Sulfonylurea)) OR (Glyburide)) OR (Glibenclamide)) OR (Glimepiride)) OR (Gliclazide)) OR (Glipizide)) OR (Gliquidone)) OR (Thiazolidinedione)) OR (Rosiglitazone)) OR (Pioglitazone)) OR (Glinide)) OR (Repaglinide)) OR (Nateglinide)) OR (Mitiglinide)) OR (α-Glucosidase inhibitor)) OR (Acarbose)) OR (Voglibose)) OR (Miglitol)) OR (Dipeptidyl peptidase 4 inhibitor)) OR (Sitagliptin)) OR (Saxagliptin)) OR (Vildagliptin)) OR (Linagliptin)) OR (Alogliptin)) OR (Sodium-glucose cotransporter 2 inhibitor)) OR (Dapagliflozin)) OR (Empagliflozin)) OR (Canagliflozin)) OR (Glucagon-like peptide-1 receptor agonist)) OR (Exenatide)) OR (Liraglutide)) OR (Lixisenatide)) OR (Beinaglutide)) OR (Insulin))) AND (Metformin)) AND ((((China) OR (Chinese)) OR (Taiwan)) OR (Hong Kong))) AND (("2000/01/01"[Date - Publication] : "2020/12/31"[Date - Publication])) Filters: Humans	898
**Database 2: Embase**		
Advanced Search	Query	Results
Search strategy	('type 2 diabetes' OR 'non insulin dependent diabetes mellitus') AND (metformin OR sulfonylurea OR glyburide OR glibenclamide OR glimepiride OR gliclazide OR glipizide OR gliquidone OR thiazolidinedione OR rosiglitazone OR pioglitazone OR glinide OR repaglinide OR nateglinide OR mitiglinide OR 'α-glucosidase inhibitor' OR acarbose OR voglibose OR miglitol OR 'dipeptidyl peptidase 4 inhibitor' OR sitagliptin OR saxagliptin OR vildagliptin OR linagliptin OR alogliptin OR 'sodium-glucose cotransporter 2 inhibitor' OR dapagliflozin OR empagliflozin OR canagliflozin OR 'glucagon-like peptide-1 receptor agonist' OR exenatide OR liraglutide OR lixisenatide OR beinaglutide OR insulin) AND metformin AND (china OR chinese OR taiwan OR 'hong kong') AND ([article]/lim OR [article in press]/lim) AND ([chinese]/lim OR [english]/lim) AND ([adult]/lim OR [young adult]/lim OR [middle aged]/lim OR [aged]/lim OR [very elderly]/lim) AND [humans]/lim AND [clinical study]/lim AND [embase]/lim AND [2000-2020]/py	1007
**Database 3: Web of Science**		
Advanced Search	Query	Results

Search strategy	TS=('Type 2 diabetes' OR 'Non insulin dependent diabetes mellitus') AND TS=(Metformin OR Sulfonylurea OR Glyburide OR Glibenclamide OR Glimepiride OR Gliclazide OR Glipizide OR Gliquidone OR Thiazolidinedione OR Rosiglitazone OR Pioglitazone OR Glinide OR Repaglinide OR Nateglinide OR Mitiglinide OR 'α-Glucosidase inhibitor' OR Acarbose OR Voglibose OR Miglitol OR 'Dipeptidyl peptidase 4 inhibitor' OR Sitagliptin OR Saxagliptin OR Vildagliptin OR Linagliptin OR Alogliptin OR 'Sodium-glucose cotransporter 2 inhibitor' OR Dapagliflozin OR Empagliflozin OR Canagliflozin OR 'Glucagon-like peptide-1 receptor agonist' OR Exenatide OR Liraglutide OR Lixisenatide OR Beinaglutide OR Insulin) AND TS=(Metformin) AND TS=(China OR Chinese OR Taiwan OR 'Hong Kong')	968
Publication date	2000 to 2020	
Article type	Article or Clinical Trial	
Language	English or Chinese	
**Database 4: China National Knowledge Infrastructure (CNKI)**		
Expert Search	Query	Results
Search strategy	(SU=2 型糖尿病 OR SU=II型糖尿病 OR SU=非胰岛素依赖型糖尿病) AND (SU=二甲双胍 OR SU=磺脲类药物 OR SU=格列本脲 OR SU=格列美脲 OR SU=格列齐特 OR SU=格列吡嗪 OR SU=格列喹酮 OR SU=噻唑烷二酮类药物 OR SU=TZDs OR SU=罗格列酮 OR SU=吡格列酮 OR SU=格列奈类药物 OR SU=瑞格列奈 OR SU=那格列奈 OR SU=米格列奈 OR SU=α-糖苷酶抑制剂 OR SU=阿卡波糖 OR SU=伏格列波糖 OR SU=米格列醇 OR SU=DPP-4 抑制剂 OR SU=西格列汀 OR SU=沙格列汀 OR SU=维格列汀 OR SU=利格列汀 OR SU=阿格列汀 OR SU=SGLT2 抑制剂 OR SU=达格列净 OR SU=恩格列净 OR SU=卡格列净 OR SU=GLP-1 受体激动剂 OR SU=艾塞那肽 OR SU=利拉鲁肽 OR SU=利司那肽 OR SU=贝那鲁肽 OR SU=胰岛素) AND (SU=二甲双胍)	13872
Publication date	2000-01-01 to 2020-12-31	
Article type	Academic Journal; Theses & Dissertations; Monographic Serials	
**Database 5: WanFang Data**		
Expert Search	Query	Results

Search strategy	(主题:(2 型糖尿病) or 主题:(II型糖尿病) or 主题:(非胰岛素依赖型糖尿病)) and (主题:(二甲双胍) or 主题:(磺脲类药物) or 主题:(格列本脲) or 主题:(格列美脲) or 主题:(格列齐特) or 主题:(格列吡嗪) or 主题:(格列喹酮) or 主题:(噻唑烷二酮类药物) or 主题:(TZDs) or 主题:(罗格列酮) or 主题:(吡格列酮) or 主题:(格列奈类药物) or 主题:(瑞格列奈) or 主题:(那格列奈) or 主题:(米格列奈) or 主题:(α-糖苷酶抑制剂) or 主题:(阿卡波糖) or 主题:(伏格列波糖) or 主题:(米格列醇) or 主题:(DPP-4 抑制剂) or 主题:(西格列汀) or 主题:(沙格列汀) or 主题:(维格列汀) or 主题:(利格列汀) or 主题:(阿格列汀) or 主题:(SGLT2 抑制剂) or 主题:(达格列净) or 主题:(恩格列净) or 主题:(卡格列净) or 主题:(GLP-1 受体激动剂) or 主题:(艾塞那肽) or 主题:(利拉鲁肽) or 主题:(利司那肽) or 主题:(贝那鲁肽) or 主题:(胰岛素)) and (主题:(二甲双胍))	12857
Publication date	2000 to 2020	
Article type	Academic Journal; Theses & Dissertations	
**Database 6: Chongqing VIP (CQVIP)**		
Expert Search	Query	Results
Search strategy	(M=2 型糖尿病 OR M=II型糖尿病 OR M=非胰岛素依赖型糖尿病) AND (M=二甲双胍 OR M=磺脲类药物 OR M=格列本脲 OR M=格列美脲 OR M=格列齐特 OR M=格列吡嗪 OR M=格列喹酮 OR M=噻唑烷二酮类药物 OR M=TZDs OR M=罗格列酮 OR M=吡格列酮 OR M=格列奈类药物 OR M=瑞格列奈 OR M=那格列奈 OR M=米格列奈 OR M=α-糖苷酶抑制剂 OR M=阿卡波糖 OR M=伏格列波糖 OR M=米格列醇 OR M=DPP-4 抑制剂 OR M=西格列汀 OR M=沙格列汀 OR M=维格列汀 OR M=利格列汀 OR M=阿格列汀 OR M=SGLT2 抑制剂 OR M=达格列净 OR M=恩格列净 OR M=卡格列净 OR M=GLP-1 受体激动剂 OR M=艾塞那肽 OR M=利拉鲁肽 OR M=利司那肽 OR M=贝那鲁肽 OR M=胰岛素) AND (M=二甲双胍)	5640
Publication date	2000 to 2020	

Table S2. Eligibility criteria

Inclusion criteria
**(1)** Participants were Chinese patients with type 2 diabetes (≥18 years).<br>**(2)** Intervention was a dual therapy of a glucose-lowering drug added to metformin. The usage and dosage of the drugs met the recommendation of Chinese clinical guideline, and metformin was administered at a constant dose (500~2000 mg/d).<br>26 drugs from eight drug classes which have been approved for type 2 diabetes in China were targeted:<br>*SUs*: glyburide (2.5~20 mg/d), glimepiride (1~8 mg/d), gliclazide (80~320; **-**MR 30~120 mg/d), glipizide (2.5~30 mg/d), gliquidone (30~180 mg/d)<br>*NIDEs*: repaglinide (1~16 mg/d), nateglinide (120~360 mg/d), mitiglinide (30~60 mg/d)<br>*TZDs*: rosiglitazone (4~8 mg/d), pioglitazone (15~45 mg/d)<br>*AGIs*: acarbose (100~300 mg/d), voglibose (0.2~0.9 mg/d), miglitol (100~300 mg/d)<br>*DPP-4is*: sitagliptin (100 mg/d), saxagliptin (5 mg/d), vildagliptin (100 mg/d), linagliptin (5 mg/d), alogliptin (25 mg/d)<br>*SGLT2is*: dapagliflozin (10 mg/d), empagliflozin (10~25 mg/d), canagliflozin (100~300 mg/d)<br>*GLP-1RAs*: exenatide (0.01~0.02 mg/d), liraglutide (0.6~1.8 mg/d), lixisenatide (0.01~0.02 mg/d), beinaglutide (0.3~0.6 mg/d)<br>*INSs*: insulin and insulin analogs<br>**(3)** Comparison was metformin monotherapy or placebo added to metformin. The usage and dosage of metformin were the same as that in Intervention.<br>**(4)** Background therapy was limited to lifestyle intervention.<br>**(5)** Primary outcome was hemoglobin Alc (HbA1c), secondary outcomes were fasting plasma glucose (FPG), 2h postprandial plasma glucose (2hPG), body mass index (BMI), total cholesterol (TC), high-density lipoprotein-cholesterol (HDL-C), systolic blood pressure (SBP), hypoglycemia, renal function, lactic acidosis, mortality, macrovascular outcomes, or microvascular outcomes. The change-from-baseline value of continuous outcomes with related standard deviation or standard error or 95% confidence interval (or the mean values of the outcomes in the baseline and in the endpoint with related standard deviation or standard error or 95% confidence interval) should be reported.<br>**(6)** Study design was randomized controlled trial.<br>**(7)** Study duration was ≥12 weeks.<br>**(8)** Study was published in Chinese or English.
Exclusion criteria

(1) Participants were not patients with type 2 diabetes, such as those with impaired glucose tolerance, impaired fasting glucose, type 1 diabetes, gestational diabetes, or latent autoimmune diabetes in adults.

(2) Participants were not Chinese patients.

(3) Participants were <18 years.

(4) Participants were comorbid with pulmonary tuberculosis, tumor, cancer, diabetic ketoacidosis, or mental diseases; or participants with severe heart, liver or kidney dysfunction; or participants during pregnancy or lactation; or participants in the perioperative period.

(5) Intervention was not a targeted glucose-lowering drug plus metformin; or in addition to the targeted drug, there were concomitant medications that affect the outcomes, such as other glucose-lowering drugs, antihypertensive drugs and antihyperlipidemic drugs; or the dose of the drugs were not targeted or not reported; or the dose of metformin changed during the treatment.

(6) Comparison was not metformin monotherapy or placebo added to metformin; or there were concomitant medications that affect the outcomes, such as other glucose-lowering drugs, antihypertensive drugs and antihyperlipidemic drugs; or the usage and dosage of metformin were not the same as that in Intervention.

(7) Study duration was <12 weeks or not reported.

(8) There was no required clinical outcome, or has required outcome but no required specific value.

(9) Study was not randomized controlled trial, such as observational study, economic evaluation, review, abstract, commentary or letter.

(10) Duplicate.

(11) Other, such as no full text, no abstract, data or content problems in article, sample size not reported.

Table S3. Characteristics and risk of bias of the included trials

Study	Characteristics of study																Risk of bias of study					
	Study duration	Background therapy	Treatment		Sample size, n		Female, n		Age, y		Diabetes duration, y		Baseline HbA1c, %		Drug dose, mg/d		Random sequence generation	Allocation concealment	Blinding of participants & personnel	Blinding of outcome assessment	Incomplete outcome data	Selective reporting
			I	C	I	C	I	C	I	C	I	C	I	C	Add-on drug	C						
Gao H 2020 [1]	3 m	nr	Acarbose+ Metformin	Metformin	50	50	23	26	50.56(2.01)	51.02(2.13)	2.87(0.84)	2.92(0.73)	9.01(1.1)	9.06(1.13)	300	750	Unclear	Unclear	High	Low	Low	Unclear
Chen BZ 2020 [2]	3 m	Diet	Acarbose+ Metformin	Metformin	43	43	20	19	67.9(7.4)	68.3(7.2)	7.3(3.2)	7.2(3.1)	10.13(1.22)	10.09(1.36)	Initial:150; Max:300	1500	Low	Unclear	High	Low	Low	Unclear
Li SJ 2020 [3]	12 w	nr	Acarbose+ Metformin	Metformin	42	42	18	20	56.58(7.83)	56.42(7.6)	7.88(3.16)	7.81(2.95)	7.85(0.99)	7.9(1.15)	150	1500	Low	Unclear	High	Low	Low	Unclear
Xing CL 2020 [4]	3 m	nr	Acarbose+ Metformin	Metformin	40	40	22	21	41.47(15.28)	43.59(12.76)	6.49(1.06)	6.64(1.45)	8.79(1.23)	8.89(0.85)	150	1500	Unclear	Unclear	High	Low	Low	Unclear
Xing LY 2020a [5]	3 m	Diet, exercise	Acarbose+ Metformin	Metformin	30	30	10	11	50.14(7.28)	50.09(7.23)	4.41(1.49)	4.38(1.52)	9.7(1.4)	9.8(1.5)	150	1000	Low	Unclear	High	Low	Low	Unclear
Zhu XF 2019 [6]	3 m	nr	Acarbose+ Metformin	Metformin	68	68	29	29	64.57(9.87)	63.3(9.4)	6.3(1.6)	6.54(1.33)	9.1(2.01)	9.02(1.98)	150	500	Low	Unclear	High	Low	Low	Unclear
Tang WQ 2019 [7]	12 w	Diet, exercise	Acarbose+ Metformin	Metformin	60	60	29	26	46.02(5.73)	45.74(5.19)	3.43(1.71)	3.27(1.32)	8.6(1.24)	8.72(0.95)	150	1500	Unclear	Unclear	High	Low	Low	Unclear
Si J 2019 [8]	12 w	nr	Acarbose+ Metformin	Metformin	57	57	25	27	56.7(5.4)	56.2(5.2)	4.6(2.5)	4.6(2)	8.6(1.1)	8.7(1.2)	150	1500	Unclear	Unclear	High	Low	Low	Unclear
Huo HW 2019 [9]	3 m	Diet, exercise	Acarbose+ Metformin	Metformin	45	45	18	19	54.7(6)	54.8(6.2)	newly diagnosed	newly diagnosed	7.3(1.57)	7.29(1.55)	150	1500	Unclear	Unclear	High	Low	Low	Unclear
Chen ZH 2019 [10]	12 w	Diet, exercise	Acarbose+ Metformin	Metformin	43	43	19	21	55.23(4.31)	55.28(4.36)	6.05(0.24)	6.11(0.27)	8.39(1.31)	8.42(1.39)	150	1500	Low	Unclear	High	Low	Low	Unclear
Li SH 2019A [11]	90 d	Diet, exercise	Acarbose+ Metformin	Metformin	38	38	14	16	66.31(4.15)	66.27(4.12)	6.43(1.57)	6.47(1.6)	8.94(1.32)	8.96(1.36)	150	1500	Low	Unclear	High	Low	Low	Unclear

Li SH 2019B [12]	12 w	nr	Acarbose+ Metformin	Metformin	32	33	14	14	54.21(2.78)	54.56(2.45)	12.11(0.21)	12.2(0.27)	11.35(2.23)	11.25(2.01)	150	1000	Unclear	Unclear	High	Low	Low	Unclear
Huang WJ 2019 [13]	90 d	nr	Acarbose+ Metformin	Metformin	30	30	10	11	51.87(5.36)	51.35(5.28)	nr	nr	8.47(0.87)	8.43(0.86)	150	750	Low	Unclear	High	Low	Low	Unclear
Yao WL 2018 [14]	3 m	Diet, exercise	Acarbose+ Metformin	Metformin	70	70	27	31	58.3(2.5)	58(2.7)	4(1.5)	3.9(1.6)	8.65(1.32)	8.68(1.2)	150	1500	Unclear	Unclear	High	Low	Low	Unclear
Yang H 2018 [15]	12 w	Diet, exercise	Acarbose+ Metformin	Metformin	63	63	30	28	49.01(8.27)	48.55(8.61)	5.58(1.62)	5.31(1.77)	8.9(0.59)	8.62(0.57)	150	1500	Low	Unclear	High	Low	Low	Unclear
Wang YL 2018 [16]	12 w	Diet, exercise	Acarbose+ Metformin	Metformin	45	45	22	21	56.39(4.67)	56.17(4.84)	4.19(1.26)	4.23(1.3)	9.37(1.58)	9.32(1.56)	150	1500	Unclear	Unclear	High	Low	Low	Unclear
Wu YY 2018 [17]	12 w	Diet, exercise	Acarbose+ Metformin	Metformin	44	43	21	23	52.6(0.4)	53.4(0.6)	nr	nr	9.17(1.57)	9.18(1.54)	Initial:150; Max:300	1500	Unclear	Unclear	High	Low	Low	Unclear
Tang J 2018 [18]	3 m	Diet, exercise	Acarbose+ Metformin	Metformin	42	42	18	17	47.2(6.8)	47.5(6.4)	0.19(0.04)	0.21(0.05)	8.65(1.27)	8.94(1.03)	150	1500	Low	Unclear	High	Low	Low	Unclear
Fang Y 2018 [19]	3 m	Diet, exercise	Acarbose+ Metformin	Metformin	41	41	16	17	53.97(4.92)	53.75(4.83)	7.35(0.29)	7.28(0.13)	9.23(1.57)	9.2(1.68)	150	1000	Unclear	Unclear	High	Low	Low	Unclear
Jiang KR 2017 [20]	12 w	nr	Acarbose+ Metformin	Metformin	182	182	87	89	48.12(6.29)	49.54(6.76)	nr	nr	8.98(1.67)	8.93(1.45)	150	1500	Unclear	Unclear	High	Low	Low	Unclear
Jiao YF 2017 [21]	3 m	nr	Acarbose+ Metformin	Metformin	68	68	56	53	58.8(9.6)	58.3(9.4)	4~9	4~9	9.83(0.43)	9.76(0.45)	150	750	Unclear	Unclear	High	Low	Low	Unclear
Ren HB 2017 [22]	12 w	Diet, exercise	Acarbose+ Metformin	Metformin	65	65	31	29	51.6(4.5)	52.3(5.1)	3.1(0.7)	3.4(0.9)	9.23(1.27)	9.19(1.41)	Initial:150; Max:300	1500	Unclear	Unclear	High	Low	Low	Unclear
Yang X 2017 [23]	3 m	Diet, exercise	Acarbose+ Metformin	Metformin	42	42	17	16	56.12(15.24)	56.48(14.46)	0.64(0.32)	0.54(0.39)	9.12(1.01)	9.24(1.18)	Initial:150; Max:300	1500	Unclear	Unclear	High	Low	Low	Unclear
Guo CX 2017 [24]	3 m	nr	Acarbose+ Metformin	Metformin	36	36	24	25	61.76(7.75)	61.94(7.41)	nr	nr	7.21(1.82)	7.32(1.62)	150	1500	Low	Unclear	High	Low	Low	Unclear
Han JK 2016 [25]	12 w	Diet, exercise	Acarbose+ Metformin	Metformin	59	59	26	26	51.03(9.48)	51.03(9.48)	5.89(1.26)	5.89(1.26)	9.38(1.56)	9.44(1.63)	150	1500	Unclear	Unclear	High	Low	Low	Unclear

Huang LY 2015 [26]	3 m	Diet, exercise	Acarbose+ Metformin	Metformin	57	57	24	25	52.15(1.25)	56.36(2.36)	5.74(1.36)	5.6(1.32)	8.74(0.41)	8.36(0.58)	150	1500	Unclear	Unclear	High	Low	Low	Unclear
Wang XQ 2015 [27]	3 m	Diet, exercise	Acarbose+ Metformin	Metformin	40	40	40	40	52.8(4.9)	52.8(4.9)	2.3(1.4)	2.3(1.4)	8.96(1.24)	8.79(1.36)	150	1500	Low	Unclear	High	Low	Low	Unclear
Liu J 2015 [28]	3 m	Diet, exercise	Acarbose+ Metformin	Metformin	33	33	11	14	50.2(10.5)	49.3(11.8)	5.7(1.5)	5.2(2.3)	9.22(1.59)	9.18(1.81)	150	1000	Unclear	Unclear	High	Low	Low	Unclear
Zu SQ 2015 [29]	3 m	Diet, exercise	Acarbose+ Metformin	Metformin	33	32	18	18	68.4(4.7)	68.4(4.7)	4.6(1.5)	4.6(1.5)	8.1(1.4)	8.3(1.3)	150	1500	Low	Unclear	High	Low	Low	Unclear
Tan YL 2014 [30]	3 m	nr	Acarbose+ Metformin	Metformin	262	262	101	130	68.1(10.1)	69.2(9.2)	5.8(2.3)	6.4(1.9)	9.43(1.41)	9.41(1.57)	150	1500	Unclear	Unclear	High	Low	Low	Unclear
Zeng XY 2014 [31]	3 m	Diet, exercise	Acarbose+ Metformin	Metformin	44	44	13	14	69.21(3.16)	69.93(3.21)	4.12(1.35)	4.32(1.41)	8.27(1.02)	8.33(1.11)	150	1500	Unclear	Unclear	High	Low	Low	Unclear
Zhu ZL 2011 [32]	6 m	Diet, exercise	Acarbose+ Metformin	Metformin	31	33	13	14	52(10)	52(10)	newly diagnosed	newly diagnosed	8.1(1.5)	8.2(1.1)	150	1500	Unclear	Unclear	High	Low	Low	Unclear
Tan RF 2017 [33]	12 w	Diet	Voglibose+ Metformin	Metformin	39	39	12	16	43.2(6.5)	42.8(6.7)	newly diagnosed	newly diagnosed	8.64(1.95)	8.76(1.73)	0.6	1500	Low	Unclear	High	Low	Low	Unclear
Xing LY 2020b [5]	3 m	Diet, exercise	Miglitol+ Metformin	Metformin	30	30	12	11	50.12(7.27)	50.09(7.23)	4.42(1.51)	4.38(1.52)	9.6(1.6)	9.8(1.5)	150	1000	Low	Unclear	High	Low	Low	Unclear
Huang Y 2015 [34]	12 w	Diet, exercise	Miglitol+ Metformin	Metformin	36	36	14	15	60.31(11.92)	59.24(10.75)	nr	nr	9.3(2.65)	9.15(2.89)	150	1000	Unclear	Unclear	High	Low	Low	Unclear
He K 2014 [35]	12 w	Diet, exercise	Miglitol+ Metformin	Metformin	50	50	21	23	53.1(9.3)	52.4(9.6)	nr	nr	7.4(1.2)	7.2(1.3)	150	1500	Unclear	Unclear	High	Low	Low	Unclear
Wang XH 2020 [36]	3 m	nr	Glimepiride+ Metformin	Metformin	41	41	7	8	74.3(3.29)	73.99(2.17)	nr	nr	8.43(0.22)	8.4(0.23)	2~6	1500	Unclear	Unclear	High	Low	Low	Unclear
Lin ML 2019 [37]	12 w	nr	Glimepiride+ Metformin	Metformin	45	45	16	19	53.8(6.4)	55.2(6.2)	newly diagnosed	newly diagnosed	8.9(1.2)	8.7(1.8)	Initial:1; Max:6	1500	Unclear	Unclear	High	Low	Low	Unclear
Chen YF 2019 [38]	3 m	nr	Glimepiride+ Metformin	Metformin	41	41	15	16	55(3.55)	55.8(3)	5.05(0.35)	5(0.32)	8.82(2.3)	8.8(2.21)	2	1500	Unclear	Unclear	High	Low	Low	Unclear

Leng B 2018 [39]	24 w	nr	Glimepiride+ Metformin	Metformin	30	30	13	12	51.5(3.5)	53.5(4.5)	6.5(2.5)	7(2.5)	10.6(1.9)	10.3(1.8)	Initial:1; Max:6	1500	Unclear	Unclear	High	Low	Low	Unclear
Wang XS 2017 [40]	3 m	Diet, exercise	Glimepiride+ Metformin	Metformin	83	83	nr	nr	71.4(5.3)	71.4(5.3)	5.2(4.3)	5.2(4.3)	9.53(1.34)	9.5(1.41)	Initial:1; Max:6	1500	Unclear	Unclear	High	Low	Low	Unclear
Chen SP 2017 [41]	12 w	Diet	Glimepiride+ Metformin	Metformin	40	40	16	18	73.24(3.55)	72.11(3.02)	nr	nr	8.8(0.6)	8.7(0.5)	Initial:1; Max:6	1000	Low	Unclear	High	Low	Low	Unclear
Yang QF 2016a [42]	15 w	nr	Glimepiride+ Metformin	Metformin	140	70	68	34	58.05(10.93)	57.39(12.24)	nr	nr	9.18(2.94)	9.37(2.65)	2~4	2000	Unclear	Unclear	High	Low	Low	Unclear
Mao ZH 2016 [43]	12 w	nr	Glimepiride+ Metformin	Metformin	50	50	18	22	46.2(10.5)	48.8(8.3)	6.3(1.2)	6.2(1.3)	9.23(1.13)	9.18(1.28)	Initial:1; Max:6	1500	Low	Unclear	High	Low	Low	Unclear
Ma CH 2016a [44]	3 m	Diet, exercise	Glimepiride+ Metformin	Metformin	30	30	12	14	45(9)	44(10)	6(3)	5(3)	8.2(0.3)	8.1(0.4)	2	1500	Unclear	Unclear	High	Low	Low	Unclear
Xu YJ 2015 [45]	3 m	nr	Glimepiride+ Metformin	Metformin	39	39	20	21	50.3(8.4)	51.2(8.2)	nr	nr	10.5(1.9)	10.3(1.7)	Initial:1; Max:6	1500	Low	Unclear	High	Low	Low	Unclear
Li X 2012 [46]	3 m	Diet, exercise	Glimepiride+ Metformin	Metformin	42	42	nr	nr	57.6(7.3)	57.6(7.3)	4.9(1.2)	4.9(1.2)	9.2(1.7)	9(1.9)	Initial:1; Max:6	1500	Unclear	Unclear	High	Low	Low	Unclear
Yi YD 2019 [47]	3 m	nr	Gliclazide+ Metformin	Metformin	25	25	11	10	51.71(2.13)	51.35(2.24)	14.52(0.13)	14.1(0.11)	11.35(1.91)	11.25(1.44)	30~60	750	Low	Unclear	High	Low	Low	Unclear
Chen YF 2013 [48]	3 m	Diet, exercise	Gliclazide+ Metformin	Metformin	42	42	18	17	50.07(8.38)	49.67(8.42)	nr	nr	12.58(4.07)	11.86(4.12)	30~90	1500	Unclear	Unclear	High	Low	Low	Unclear
He F 2013 [49]	6 m	nr	Gliclazide+ Metformin	Metformin	43	43	nr	nr	50.2(7.5)	50.2(7.5)	4.6(1.5)	4.6(1.5)	11.7(1.9)	12.1(2.3)	Initial:30; Max:120	1500	Unclear	Unclear	High	Low	Low	Unclear
Luo ZZ 2012 [50]	16 w	Diet, exercise	Gliclazide+ Metformin	Metformin	50	50	27	25	45.1(7.8)	43.2(8.1)	0.17~10	0.17~10	8.1(0.6)	8.2(0.5)	30	1500	Unclear	Unclear	High	Low	Low	Unclear
Guo XT 2020 [51]	12 w	nr	Glipizide+ Metformin	Metformin	51	51	26	24	60.13(10.68)	59.52(10.61)	nr	nr	7.47(1.24)	7.36(1.12)	10	1700	Unclear	Unclear	High	Low	Low	Unclear
Wang Q 2020 [52]	3 m	nr	Glipizide+ Metformin	Metformin	35	35	17	14	68.5(3.2)	69.3(3)	nr	nr	8.1(1.3)	8(1.2)	7.5~30	1000	Low	Unclear	High	Low	Low	Unclear

Sun HQ 2019 [53]	90 day	Diet, exercise	Glipizide+ Metformin	Metformin	55	55	25	26	61~79	60~80	5.92(1.74)	5.92(1.74)	8.01(1.28)	8.02(1.3)	15	1500	Low	Unclear	High	Low	Low	Unclear
Bao YM 2017 [54]	3 m	Diet, exercise	Glipizide+ Metformin	Metformin	80	80	28	26	68.47(5.83)	68.4(5.81)	nr	nr	11.35(3.72)	11.45(3.79)	10	500	Low	Unclear	High	Low	Low	Unclear
Wang SQ 2016a [55]	12 w	nr	Glipizide+ Metformin	Metformin	20	20	8	8	53.24(8.71)	53.24(8.71)	2.1(0.6)	2.1(0.6)	7.74(1.96)	7.68(1.82)	5	1000	Unclear	Unclear	Low	Low	Low	Unclear
Guo SB 2012 [56]	12 w	nr	Glipizide+ Metformin	Metformin	61	61	25	25	33~62	33~62	nr	nr	9.4(1.8)	9.1(2.1)	15	1500	Unclear	Unclear	High	Low	Low	Unclear
Liu GL 2004 [57]	16 w	Diet, exercise	Gliquidone+ Metformin	Metformin	22	16	12	7	56.9(3.8)	56.1(3.4)	8.7(1.4)	8.7(1.4)	10.01(0.56)	9.21(0.43)	90~180	1500	Unclear	Unclear	High	Low	Low	Unclear
Pi HC 2020 [58]	12 w	Diet, exercise	Repaglinide+ Metformin	Metformin	62	62	26	24	52.41(9.52)	52.46(9.48)	nr	nr	8.06(1.37)	8.04(1.39)	1	1500	Unclear	Unclear	High	Low	Low	Unclear
Ye Q 2020 [59]	3 m	Diet, exercise	Repaglinide+ Metformin	Metformin	50	50	21	24	71.83(3.52)	72.54(3.69)	6.97(1.43)	7.43(1.32)	9.33(1.36)	9.42(1.27)	3	750	Unclear	Unclear	High	Low	Low	Unclear
Zhou C 2019 [60]	4 m	nr	Repaglinide+ Metformin	Metformin	65	65	33	35	51.36(9.45)	51.11(9.13)	7.45(1.52)	7.26(1.36)	9.61(2.12)	9.54(2.04)	Initial:1.5; Max:6	1500	Low	Unclear	High	Low	Low	Unclear
Yan HP 2019 [61]	12 w	nr	Repaglinide+ Metformin	Metformin	50	50	nr	nr	63.8(5.7)	63.8(5.7)	5.8(2.7)	5.8(2.7)	10.48(1.69)	10.51(1.62)	Initial:1.5; Max:3	1500	Low	Unclear	High	Low	Low	Unclear
Zhu FL 2018 [62]	3 m	nr	Repaglinide+ Metformin	Metformin	77	77	38	40	53.96(6.26)	55.19(6.53)	8.43(3.19)	8.05(3.37)	9.28(1.33)	9.15(1.64)	1.5	1500	Low	Unclear	High	Low	Low	Unclear
Li JP 2018 [63]	4 m	nr	Repaglinide+ Metformin	Metformin	55	55	24	26	51.8(7.9)	52.5(8.4)	newly diagnosed	newly diagnosed	9.49(1.28)	9.37(1.34)	1.5	1500	Unclear	Unclear	High	Low	Low	Unclear
Gao S 2017 [64]	4 m	Diet, exercise	Repaglinide+ Metformin	Metformin	67	67	31	29	51.13(9.15)	51.78(8.64)	nr	nr	9.63(2.14)	9.56(2.06)	Initial:1.5; Max:6	1500	Low	Unclear	High	Low	Low	Unclear
Huang YY 2017 [65]	4 m	Diet, exercise	Repaglinide+ Metformin	Metformin	60	60	23	22	53.04(7.13)	53.18(7.24)	newly diagnosed	newly diagnosed	9.48(1.27)	9.38(1.26)	1.5	1500	Low	Unclear	High	Low	Low	Unclear
Han J 2017 [66]	4 m	Diet, exercise	Repaglinide+ Metformin	Metformin	43	43	17	18	51.96(6.78)	52.73(6.15)	10.64(3.25)	10.09(3.67)	9.39(1.58)	9.15(1.24)	1.5	1500	Low	Unclear	High	Low	Low	Unclear

Hu ZY 2016 [67]	4 m	Diet, exercise	Repaglinide+ Metformin	Metformin	72	72	28	32	53.12(2.04)	52.29(2.11)	newly diagnosed	newly diagnosed	9.47(1.26)	9.39(1.25)	1.5	1500	Unclear	Unclear	High	Low	Low	Unclear
Yu YY 2015 [68]	12 w	Diet, exercise	Repaglinide+ Metformin	Metformin	60	60	24	26	52.5(10.2)	53.7(9.5)	nr	nr	10.1(1.6)	10.4(1.4)	1.5	1500	Unclear	Unclear	High	Low	Low	Unclear
Sun ZC 2015 [69]	3 m	Diet, exercise	Repaglinide+ Metformin	Metformin	40	40	17	18	59.1(5.1)	58.3(4.9)	nr	nr	11.47(2.31)	11.23(2.01)	Initial:1.5; Max:12	1500	Low	Unclear	High	Low	Low	Unclear
Xu SL 2013 [70]	3 m	nr	Repaglinide+ Metformin	Metformin	280	280	93	93	38.7	38.7	newly diagnosed	newly diagnosed	8.5(1.3)	8.4(1.3)	1.5	1500	Unclear	Unclear	High	Low	Low	Unclear
He M 2013A [71]	3 m	Diet, exercise	Repaglinide+ Metformin	Metformin	74	74	36	36	47.27(8.72)	47.27(8.72)	nr	nr	9.38(1.36)	9.4(1.94)	Initial:1.5; Max:16	1500	Unclear	Unclear	High	Low	Low	Unclear
He M 2013B [72]	3 m	Diet, exercise	Repaglinide+ Metformin	Metformin	61	61	25	24	46.05(10.26)	45.68(9.18)	newly diagnosed	newly diagnosed	8.92(1.8)	8.83(1.62)	Initial:1.5; Max:16	1500	Unclear	Unclear	High	Low	Low	Unclear
Lei YT 2020 [73]	4 m	nr	Sitagliptin+ Metformin	Metformin	400	400	150	150	62.9(3.1)	60.3(4.1)	7.3(2.6)	6.5(2.3)	9.62(1.54)	9.59(1.26)	100	1500	Unclear	Unclear	High	Low	Low	Unclear
Hu D 2020 [74]	3 m	nr	Sitagliptin+ Metformin	Metformin	92	92	41	44	54.8(8.1)	54.4(7.2)	newly diagnosed	newly diagnosed	8.45(1.94)	8.37(1.89)	100	1500	Unclear	Unclear	High	Low	Low	Unclear
Ding XY 2020 [75]	3 m	nr	Sitagliptin+ Metformin	Metformin	74	74	33	32	57.1(4)	56.7(4.3)	3.1(0.2)	3.1(0.2)	7.91(1.19)	7.86(1.21)	100	1500	Low	Unclear	High	Low	Low	Unclear
Luo HQ 2020[76]	12 m	Diet, exercise	Sitagliptin+ Metformin	Metformin	57	56	28	26	40.27(5.81)	39.84(4.96)	newly diagnosed	newly diagnosed	10.57(2.04)	10.48(2.31)	100	1500	Low	Unclear	High	Low	Low	Unclear
Zhang XL 2020 [77]	12 w	nr	Sitagliptin+ Metformin	Metformin	55	55	26	26	56.47(3.56)	56.47(3.56)	3.17(0.52)	3.17(0.52)	9.6(1.72)	9.63(1.67)	100	1500	Low	Unclear	High	Low	Low	Unclear
Liang YF 2020 [78]	12 w	Diet, exercise	Sitagliptin+ Metformin	Metformin	55	55	22	23	55(12)	55.4(12.3)	5.7(2.2)	5.7(2.3)	8.9(1.4)	8.7(1.3)	100	500	Low	Unclear	High	Low	Low	Unclear
Li Y 2020 [79]	3 m	Diet, exercise	Sitagliptin+ Metformin	Metformin	50	50	23	24	55.14(6.03)	54.99(5.73)	4.12(1.02)	4.09(0.92)	10.52(3.47)	10.64(3.5)	100	1500	Low	Unclear	High	Low	Low	Unclear
Sun XO 2020 [80]	12 w	Diet, exercise	Sitagliptin+ Metformin	Metformin	50	50	24	23	31.25(13.18)	32.82(13.36)	2.32(1.68)	2.74(1.79)	8.52(1.42)	8.82(1.58)	100	1500	Unclear	Unclear	High	Low	Low	Unclear

He C 2020 [81]	3 m	Diet, exercise	Sitagliptin+ Metformin	Metformin	49	49	28	31	71.2(6.59)	72.6(6.04)	nr	nr	8.95(1.63)	8.97(1.65)	100	1500	Low	Unclear	High	Low	Low	Unclear
Xiao BM 2020 [82]	3 m	nr	Sitagliptin+ Metformin	Metformin	44	43	25	23	39.79(5.32)	39.23(5.16)	nr	nr	11.12(1.46)	10.23(2.15)	100	1000	Unclear	Unclear	High	Low	Low	Unclear
Wu SL 2020 [83]	3 m	Diet, exercise	Sitagliptin+ Metformin	Metformin	43	43	18	20	54.15(12.92)	53.92(15.06)	0.67(0.11)	0.66(0.13)	8.57(1.28)	8.29(1.4)	100	1500	Low	Unclear	High	Low	Low	Unclear
Chen J 2020 [84]	12 w	Diet, exercise	Sitagliptin+ Metformin	Metformin	40	40	17	18	62.3	62.2	4.5	4.6	8.2(1.1)	8.3(1.3)	100	1500	Low	Unclear	High	Low	Low	Unclear
Niu XP 2020 [85]	3 m	Diet, exercise	Sitagliptin+ Metformin	Metformin	37	37	15	16	56.38(5.24)	56.31(5.21)	3.21(0.56)	3.14(0.58)	8.17(0.74)	8.22(0.76)	100	1500	Unclear	Unclear	High	Low	Low	Unclear
Yang XQ 2020 [86]	12 w	Diet, exercise	Sitagliptin+ Metformin	Metformin	35	35	14	15	49.54(2.45)	49.24(2.92)	9.83(2.07)	10.14(2.12)	9.68(1.47)	9.69(1.38)	100	1500	Unclear	Unclear	High	Low	Low	Unclear
Hu B 2020 [87]	3 m	Diet, exercise	Sitagliptin+ Metformin	Metformin	28	28	11	12	52.8(3.28)	51.7(3.01)	nr	nr	7.4(0.8)	7.3(0.6)	100	1500	Unclear	Unclear	High	Low	Low	Unclear
Liu F 2019 [88]	12 w	Diet, exercise	Sitagliptin+ Metformin	Metformin	100	100	46	48	54.2(2.43)	54.4(2.38)	nr	nr	8.4(1.7)	8.5(1.8)	100	1500	Low	Unclear	High	Low	Low	Unclear
Liu L 2019 [89]	3 m	Diet, exercise	Sitagliptin+ Metformin	Metformin	78	78	34	36	44.55(2.17)	44.39(2.02)	nr	nr	8.49(0.7)	8.44(0.74)	100	1000	Unclear	Unclear	High	Low	Low	Unclear
Wang J 2019 [90]	12 w	nr	Sitagliptin+ Metformin	Metformin	69	69	39	40	59.5(3.4)	58.6(3.4)	5.29(1.02)	5.41(1.08)	7.62(2.33)	7.58(2.38)	100	1500	Low	Unclear	High	Low	Low	Unclear
Li J 2019 [91]	12 w	nr	Sitagliptin+ Metformin	Metformin	60	60	25	27	55.6(4.4)	55.52(4.37)	5.43(1.2)	5.32(1.14)	8.86(1.54)	8.9(1.51)	100	1500	Low	Unclear	High	Low	Low	Unclear
Xie D 2019 [92]	12 w	Diet, exercise	Sitagliptin+ Metformin	Metformin	55	55	nr	nr	48.5(3.6)	48.5(3.6)	< 5	< 5	9.7(1.3)	9.7(1.4)	100	1500	Unclear	Unclear	High	Low	Low	Unclear
Li BA 2019 [93]	12 w	nr	Sitagliptin+ Metformin	Metformin	55	55	27	26	52.17(2.43)	53.11(2.54)	2.52(1.34)	2.61(1.38)	8.59(1.35)	8.65(1.28)	100	1500	Low	Unclear	High	Low	Low	Unclear
Wu P 2019 [94]	13 w	nr	Sitagliptin+ Metformin	Metformin	41	41	21	19	53.01(6.81)	52.38(6.94)	newly diagnosed	newly diagnosed	8.58(1.26)	8.62(1.21)	100	1500	Low	Unclear	High	Low	Low	Unclear

Yang WD 2018 [95]	6 m	Diet, exercise	Sitagliptin+ Metformin	Metformin	68	60	36	24	46.31(12.03)	45.64(11.35)	0.30(0.10)	0.31(0.1)	8.96(1.4)	8.94(1.39)	100	1500	Unclear	Unclear	High	Low	Low	Unclear
Li HX 2018 [96]	12 w	nr	Sitagliptin+ Metformin	Metformin	60	60	24	23	46.43(5.62)	60.25(6.25)	3.15(1.36)	3.07(1.26)	11.04(1.54)	11.07(1.35)	100	1000	High	Unclear	High	Low	Low	Unclear
Jing HW 2018 [97]	12 w	Diet, exercise	Sitagliptin+ Metformin	Metformin	60	60	32	32	58.86(4.81)	58.23(5.01)	nr	nr	9.46(1.12)	9.42(1.13)	100	1500	Low	Unclear	High	Low	Low	Unclear
Chen L 2018 [98]	12 w	nr	Sitagliptin+ Metformin	Metformin	50	50	21	23	52.19(3.64)	55.46(2.73)	3.16(1.77)	4.22(2.15)	8.43(2.01)	8.54(1.98)	100	1500	Low	Unclear	High	Low	Low	Low
Chen Z 2018 [99]	12 w	Diet, exercise	Sitagliptin+ Metformin	Metformin	49	49	17	25	46(12)	48(12)	1.3(1)	1.1(1.3)	9.7(1.4)	9.6(1.2)	100	1500	Low	Unclear	High	Low	Low	Low
Li F 2018 [100]	4 m	Diet, exercise	Sitagliptin+ Metformin	Metformin	47	47	19	17	49.83(4.9)	48.04(5.82)	1.99(0.43)	1.94(0.41)	7.69(1.05)	7.55(0.97)	100	1000	Low	Unclear	High	Low	Low	Unclear
Zhou QM 2018 [101]	12 w	nr	Sitagliptin+ Metformin	Metformin	46	46	22	22	54.82(7.69)	54.82(7.69)	3.28(1.16)	3.28(1.16)	10.59(3.32)	10.25(3.27)	100	1500	Unclear	Unclear	High	Low	Low	Unclear
Zhu HQ 2018 [102]	3 m	nr	Sitagliptin+ Metformin	Metformin	34	34	11	14	68.6(2.8)	70.1(2.9)	newly diagnosed	newly diagnosed	8.82(0.83)	8.79(0.81)	100	2000	Low	Unclear	High	Low	Low	Unclear
Zhang CJ 2018 [103]	12 w	nr	Sitagliptin+ Metformin	Metformin	30	30	14	15	56.35(10.27)	58.72(8.51)	10.31(3.13)	12.08(4.63)	8.82(1.18)	8.65(1.22)	100	1500	Low	Unclear	High	Low	Low	Unclear
Tang Y 2017 [104]	24 w	nr	Sitagliptin+ Metformin	Metformin	146	143	78	73	67(3.4)	69(4.6)	6.4(2.2)	6(3.4)	8.8(2.1)	8.6(3.2)	100	1500	Unclear	Unclear	High	Low	Low	Unclear
Guan XP 2017 [105]	12 w	nr	Sitagliptin+ Metformin	Metformin	60	60	28	27	60.11(8.72)	60.2(8.69)	12.85(2.93)	12.9(2.87)	8.13(1.46)	8.18(1.5)	100	1500	Low	Unclear	High	Low	Low	Unclear
Gao HM 2017 [106]	12 w	Diet, exercise	Sitagliptin+ Metformin	Metformin	48	48	20	22	46.62(4.55)	47.51(4.32)	0.23(0.14)	0.24(0.13)	8.04(1.44)	8.17(1.39)	100	1500	Unclear	Unclear	High	Low	Low	Unclear
Yang N 2017 [107]	3 m	nr	Sitagliptin+ Metformin	Metformin	47	47	22	24	69.87(5.14)	68.18(5.06)	1.87(0.67)	2.01(0.51)	8.55(1.79)	8.46(1.82)	100	1500	Low	Unclear	High	Low	Low	Unclear
Zhou Y 2017 [108]	12 w	Diet, exercise	Sitagliptin+ Metformin	Metformin	45	45	22	24	45.6(7.3)	47.2(7.8)	newly diagnosed	newly diagnosed	10.46(3.25)	10.36(3.42)	100	1500	Low	Unclear	High	Low	Low	Unclear

Li JJ 2017 [109]	6 m	Diet, exercise	Sitagliptin+ Metformin	Metformin	43	43	19	18	44.6(12.3)	44.7(12.4)	5.3(2.3)	5.3(2.4)	9.2(1.6)	9.3(1.6)	100	1500	Low	Unclear	High	Low	Low	Unclear
Ji LN 2016a [110]	24 w	Diet, exercise	Sitagliptin+ Metformin	Metformin	125	124	58	49	52.4(9.3)	53(10.3)	1.1(0.3)	1.1(0.2)	8.6(0.9)	8.7(1.1)	100	1700	Low	Low	Low	Low	Low	Low
Ji LN 2016b [110]	24 w	Diet, exercise	Sitagliptin+ Metformin	Metformin	122	126	37	57	52.6(11.3)	52.6(9.5)	1.1(0.3)	1(0.2)	8.5(1)	8.7(1)	100	1000	Low	Low	Low	Low	Low	Low
Yang QM 2016 [111]	12 w	Diet, exercise	Sitagliptin+ Metformin	Metformin	49	49	23	23	20~65	20~65	newly diagnosed	newly diagnosed	8(0.5)	8.3(0.2)	100	1500	Unclear	Unclear	High	Low	Low	Unclear
Hu GH 2016 [112]	3 m	Diet, exercise	Sitagliptin+ Metformin	Metformin	48	48	nr	nr	53.2(5.1)	53.2(5.1)	newly diagnosed	newly diagnosed	8.7(1.3)	8.6(1.4)	100	500	Unclear	Unclear	High	Low	Low	Unclear
Wu XH 2016 [113]	12 w	Diet, exercise	Sitagliptin+ Metformin	Metformin	45	45	nr	nr	45.5	45.5	3.5	3.5	8.53(2.51)	8.23(2.44)	100	1500	Unclear	Unclear	High	Low	Low	Unclear
Yang F 2016 [114]	6 m	Diet, exercise	Sitagliptin+ Metformin	Metformin	45	45	20	19	56.58(8.31)	56.62(8.28)	5.68(1.35)	5.71(1.37)	10.22(1.4)	10.15(1.38)	100	1500	Unclear	Unclear	High	Low	Low	Unclear
Li LQ 2016 [115]	12 w	Diet, exercise	Sitagliptin+ Metformin	Metformin	42	41	15	14	45.34(3.13)	45.12(3.43)	newly diagnosed	newly diagnosed	11.35(1.92)	11.25(1.41)	100	1500	Low	Unclear	High	Low	Low	Unclear
Zhang TT 2016 [116]	90 d	Diet, exercise	Sitagliptin+ Metformin	Metformin	41	41	20	21	60.98(3.14)	61.13(2.92)	6.14(0.52)	5.85(0.12)	7.86(1.18)	7.67(1.15)	100	500	Low	Unclear	High	Low	Low	Unclear
Han M 2016 [117]	12 w	Diet, exercise	Sitagliptin+ Metformin	Metformin	34	34	14	13	50.62(4.14)	50.43(4.21)	6.15(1.23)	6.21(1.29)	8.76(1.37)	8.83(1.31)	100	1500	Low	Unclear	High	Low	Low	Unclear
Pang XX 2016 [118]	3 m	Diet, exercise	Sitagliptin+ Metformin	Metformin	33	32	12	10	52.81(9.54)	53.51(5.87)	≤0.75	≤0.75	9.42(2.64)	9.57(1.72)	100	1500	Unclear	Unclear	High	Low	Low	Unclear
Wang XL 2016 [119]	12 w	nr	Sitagliptin+ Metformin	Metformin	32	32	14	15	44.5(5.3)	44.8(5.7)	nr	nr	8.1(1.8)	8.2(1.7)	100	1500	Unclear	Unclear	High	Low	Low	Unclear
Qiao YC 2016 [120]	3 m	Diet, exercise	Sitagliptin+ Metformin	Metformin	32	32	14	14	47.6(2.4)	47.6(2.4)	4.3(1.2)	4.3(1.2)	8(1.5)	8.2(1.7)	100	1500	Unclear	Unclear	High	Low	Low	Unclear
Qi BB 2016 [121]	24 w	Diet, exercise	Sitagliptin+ Metformin	Metformin	30	30	12	16	40(12)	42(10)	newly diagnosed	newly diagnosed	7.5(0.6)	7.3(0.5)	100	1500	Low	Unclear	High	Low	Low	Unclear

Jiang X 2016 [122]	12 m	Diet, exercise	Sitagliptin+ Metformin	Metformin	30	30	14	11	43.7(6.5)	44.6(8.7)	6.5(0.8)	6.2(0.7)	8.75(1.19)	8.69(1.42)	100	1500	Unclear	Unclear	High	Low	Low	Unclear
Yao L 2016 [123]	16 w	Diet, exercise	Sitagliptin+ Metformin	Metformin	30	30	11	11	49.5(9.2)	49.5(9.2)	1~12	1~12	7.97(1.24)	8.14(1.24)	100	1000	Low	Unclear	High	Low	Low	Unclear
Zhang N 2015 [124]	3 m	Diet, exercise	Sitagliptin+ Metformin	Metformin	62	62	21	22	54.2(5.2)	54.6(5.6)	5.5(0.9)	5.3(0.7)	9(1.4)	8.8(1.3)	100	1500	Low	Unclear	High	Low	Low	Unclear
Yuan XJ 2015 [125]	3 m	Diet, exercise	Sitagliptin+ Metformin	Metformin	50	50	22	24	56.5	54.5	newly diagnosed	newly diagnosed	11.24(3.32)	11.43(3.27)	100	1500	Unclear	Unclear	High	Low	Low	Unclear
Zhang HT 2015 [126]	24 w	nr	Sitagliptin+ Metformin	Metformin	35	35	15	15	48.5(10.8)	49.2(11.2)	0.6(0.13)	0.59(0.11)	8.31(1.25)	8.29(1.33)	100	1500	Unclear	Unclear	High	Low	Low	Unclear
Zhuo FT 2015 [127]	12 w	Diet, exercise	Sitagliptin+ Metformin	Metformin	34	35	17	17	62.71(12.93)	61.65(12.55)	newly diagnosed	newly diagnosed	9.34(2.42)	9.37(2.38)	100	1500	Unclear	Unclear	High	Low	Low	Unclear
Fan SH 2015 [128]	12 w	Diet, exercise	Sitagliptin+ Metformin	Metformin	32	32	14	13	55.3(10.8)	52.5(9.6)	newly diagnosed	newly diagnosed	8.5(2.6)	8.2(2.5)	100	1500	Low	Unclear	High	Low	Low	Unclear
Li Z 2015 [129]	12 w	Diet, exercise	Sitagliptin+ Metformin	Metformin	31	31	12	12	47.9(4.2)	47.9(4.2)	nr	nr	9(1.3)	9.3(1.4)	100	1500	Low	Unclear	High	Low	Low	Unclear
Chen P 2015a [130]	12 w	Diet, exercise	Sitagliptin+ Metformin	Metformin	30	30	nr	nr	nr	nr	nr	nr	8.2(1.3)	8.1(1.4)	100	1500	Unclear	Unclear	High	Low	Low	Unclear
Guo YF 2014 [131]	3 m	Diet, exercise	Sitagliptin+ Metformin	Metformin	45	45	0	0	78(5.5)	78(5.5)	18.5(2.8)	18.5(2.8)	8.1(1.4)	8(1.3)	100	1500	Unclear	Unclear	High	Low	Low	Unclear
Zhang XF 2014 [132]	12 w	Diet, exercise	Sitagliptin+ Metformin	Metformin	40	40	14	12	45(16)	44(17)	newly diagnosed	newly diagnosed	10.01(1.28)	10.11(1.1)	100	1500	Low	Unclear	High	Low	Low	Unclear
Qiu W 2014 [133]	3 m	Diet, exercise	Sitagliptin+ Metformin	Metformin	40	40	17	15	52.34(3.47)	52.34(3.47)	6.23(1.69)	6.23(1.69)	11.52(3.91)	11.36(3.74)	100	1500	Unclear	Unclear	High	Low	Low	Unclear
Nie FR 2013 [134]	4 m	nr	Sitagliptin+ Metformin	Metformin	80	80	37	36	58.7(4.6)	58.3(4.2)	0.44(0.13)	5.1(1.4)	7.63(1.92)	7.56(1.97)	100	1500	Unclear	Unclear	High	Low	Low	Unclear
Li WM 2013 [135]	3 m	nr	Sitagliptin+ Metformin	Metformin	70	70	33	35	51.3(8.38)	52.1(7.72)	6.9	7	8.7(1.1)	9.5(1.4)	100	1500	Unclear	Unclear	High	Low	Low	Unclear

Zhang LL 2013 [136]	12 w	Diet, exercise	Sitagliptin+ Metformin	Metformin	57	56	20	23	54.9(10.8)	55.8(10.7)	6.17(1.34)	6.19(1.35)	9.13(1.39)	9.51(1.46)	100	1500	Unclear	Unclear	High	Low	Low	Unclear
Zhang HY 2013 [137]	12 w	Diet, exercise	Sitagliptin+ Metformin	Metformin	48	48	23	21	52(4.9)	51.8(5.2)	newly diagnosed	newly diagnosed	8.3(1.4)	8.3(1.7)	100	750	Unclear	Unclear	High	Low	Low	Unclear
Jiao XM 2013 [138]	12 w	Diet, exercise	Sitagliptin+ Metformin	Metformin	30	30	13	15	46.4(8.2)	43.3(7.1)	newly diagnosed	newly diagnosed	8(1.5)	8.2(1.7)	100	1500	Unclear	Unclear	High	Low	Low	Unclear
Li J 2020 [139]	3 m	nr	Saxagliptin+ Metformin	Metformin	100	100	42	40	63.28(9.12)	63.28(8.25)	14.9(10.22)	14.88(8.56)	7.55(1.05)	7.55(1.22)	5	2000	Low	Unclear	High	Low	Low	Unclear
Deng YY 2020 [140]	3 m	nr	Saxagliptin+ Metformin	Metformin	51	51	24	26	60.14(10.73)	59.56(9.82)	0.76(0.19)	0.73(0.19)	7.92(1.55)	7.86(1.63)	5	1500	Low	Unclear	High	Low	Low	Unclear
Zheng HY 2020 [140]	3 m	nr	Saxagliptin+ Metformin	Metformin	39	39	18	17	55.71(6.31)	55.89(6.27)	1.12(0.56)	1.18(0.61)	8.68(1.63)	8.67(1.62)	5	1500	Low	Unclear	High	Low	Low	Unclear
Zhao PY 2019 [142]	12 w	Diet, exercise	Saxagliptin+ Metformin	Metformin	38	38	nr	nr	55.3(2.5)	55.2(2.2)	6(1.2)	6(1.2)	8.5(0.9)	8.1(1.1)	5	1500	Unclear	Unclear	High	Low	Low	Unclear
Mei YJ 2018 [143]	12 w	Diet, exercise	Saxagliptin+ Metformin	Metformin	50	50	16	20	52.5(2.3)	53.2(2.1)	newly diagnosed	newly diagnosed	8.5(2.1)	8.6(2.3)	5	1500	Low	Unclear	High	Low	Low	Unclear
Shi FM 2018 [144]	28 w	nr	Saxagliptin+ Metformin	Metformin	40	40	16	15	48.9(9.1)	48.6(8.9)	nr	nr	7.49(0.54)	7.41(0.47)	5	2000	Unclear	Unclear	High	Low	Low	Unclear
Shan L 2018 [145]	24 w	Diet	Saxagliptin+ Metformin	Metformin	35	35	16	15	63.8(3.2)	64.5(2.5)	8.3(2.3)	8.5(2.5)	8.55(1.15)	8.56(1.2)	5	2000	Low	Unclear	High	Low	Low	Unclear
Tao T 2018 [146]	24 w	Diet, exercise	Saxagliptin+ Metformin	Metformin	21	21	nr	nr	29(5)	28(3)	newly diagnosed	newly diagnosed	7.4(0.3)	7.3(0.2)	5	2000	Low	Unclear	High	Low	Low	Low
Li GH 2017 [147]	12 w	Diet, exercise	Saxagliptin+ Metformin	Metformin	96	96	nr	nr	52.70(10.71)	53.09(11.59)	newly diagnosed	newly diagnosed	8.21(0.76)	8.22(0.67)	5	1500	Low	Unclear	High	Low	Low	Unclear
Dong H 2017 [148]	6 m	nr	Saxagliptin+ Metformin	Metformin	58	58	31	30	58(7.8)	58.4(7.2)	5.4(2.1)	5(2.9)	8.36(0.77)	8.19(0.94)	5	1500	Low	Unclear	High	Low	Low	Unclear
Zhang W 2017 [149]	12 w	Diet, exercise	Saxagliptin+ Metformin	Metformin	42	42	19	18	46.21(5.26)	47.05(5.12)	newly diagnosed	newly diagnosed	9.37(1.44)	9.36(1.45)	5	1500	Unclear	Unclear	High	Low	Low	Unclear

Li HW 2017 [150]	12 w	nr	Saxagliptin+ Metformin	Metformin	32	32	nr	nr	nr	nr	nr	nr	9.68(1.25)	9.36(1.29)	5	1500	Low	Unclear	High	Low	Low	Unclear
Zhang GP 2017 [151]	6 m	nr	Saxagliptin+ Metformin	Metformin	30	30	13	12	58.51(2.49)	58.57(2.53)	newly diagnosed	newly diagnosed	9.32(1.45)	9.38(1.49)	5	1500	Low	Unclear	High	Low	Low	Unclear
Ding R 2016 [152]	3 m	Diet, exercise	Saxagliptin+ Metformin	Metformin	30	30	10	12	48.73(8.7)	50.2(7.74)	5.11(1.29)	4.58(1.3)	10.3(1.12)	9.9(1.01)	5	1500	Unclear	Unclear	High	Low	Low	Unclear
Wang SQ 2016b [55]	12 w	nr	Saxagliptin+ Metformin	Metformin	20	20	8	8	53.24(8.71)	53.24(8.71)	2.1(0.6)	2.1(0.6)	7.56(2.11)	7.68(1.82)	5	1000	Unclear	Unclear	Low	Low	Low	Unclear
Chen QL 2015 [153]	16 w	Diet, exercise	Saxagliptin+ Metformin	Metformin	85	85	40	42	46.5(5.2)	47.4(4.8)	newly diagnosed	newly diagnosed	8.4(1.7)	8.2(1.7)	5	1500	Unclear	Unclear	High	Low	Low	Unclear
Liang M 2015 [153]	12 w	Diet, exercise	Saxagliptin+ Metformin	Metformin	48	48	22	23	54.2(1.7)	53.2(1.5)	newly diagnosed	newly diagnosed	9.23(1.56)	9.36(1.45)	5	1500	Unclear	Unclear	High	Low	Low	Unclear
Feng J 2015 [155]	12 w	nr	Saxagliptin+ Metformin	Metformin	48	48	10	12	79.02(3.26)	78.02(4.56)	17(4.23)	18(4.06)	8.2(1.2)	8.3(1.1)	5	1500	Low	Unclear	High	Low	Low	Unclear
Diao YL 2015 [156]	24 w	nr	Saxagliptin+ Metformin	Metformin	47	48	19	20	55.9(10.8)	52.3(10.1)	6.2(2.1)	5.6(2.4)	7.5(0.65)	7.52(0.58)	5	1500	Low	Unclear	High	Low	Low	Unclear
Wan J 2015 [157]	24 w	nr	Saxagliptin+ Metformin	Metformin	35	33	19	18	55.5(16.38)	55.5(16.38)	newly diagnosed	newly diagnosed	8.24(1.71)	8.49(1.67)	5	1000	Unclear	Unclear	Low	Low	Low	Unclear
Sun Y 2014A [158]	12 w	Diet, exercise	Saxagliptin+ Metformin	Metformin	44	40	20	19	56(8)	52(7)	newly diagnosed	newly diagnosed	8.26(2.15)	8.19(2.51)	5	1500	Unclear	Unclear	High	Low	Low	Unclear
Lu JE 2014 [159]	16 w	Diet	Saxagliptin+ Metformin	Metformin	30	30	16	12	46(12)	45(13)	0.02(0.00)	0.02 (0.00)	8.4(1.6)	8.2(1.8)	5	1500	Low	Unclear	High	Low	Low	Unclear
Sun Y 2014B [160]	12 w	Diet, exercise	Saxagliptin+ Metformin	Metformin	30	30	13	12	56.1(8.4)	52.4(7.2)	newly diagnosed	newly diagnosed	8.27(1.53)	8.37(2.01)	5	1500	Unclear	Unclear	High	Low	Low	Unclear
Li JJ 2013 [161]	24 w	nr	Saxagliptin+ Metformin	Metformin	33	33	17	14	56.48(9.13)	52.79(11.72)	newly diagnosed	newly diagnosed	7.55(0.74)	7.36(0.74)	5	1000	Low	Unclear	High	Low	Low	Unclear
Chu YN 2020 [162]	24 w	nr	Vildagliptin+ Metformin	Metformin	80	80	32	35	47.92(4.45)	48.34(4.71)	4.23(1.22)	4.4(1.3)	9.23(1.8)	9.32(1.42)	100	2000	Low	Unclear	High	Low	Low	Unclear

Wang TM 2020 [163]	12 w	Diet, exercise	Vildagliptin+ Metformin	Metformin	48	49	19	21	51.4(4.5)	51(4.4)	nr	nr	8.24(1.49)	8.21(1.47)	100	1500	Unclear	Unclear	High	Low	Low	Unclear
Wang QL 2020 [164]	16 w	nr	Vildagliptin+ Metformin	Metformin	48	47	24	21	62.8(8.9)	63.8(9.1)	nr	nr	8.64(1.11)	8.51(1.04)	100	1000	Unclear	Unclear	High	Low	Low	Unclear
Feng Y 2019 [165]	12 w	nr	Vildagliptin+ Metformin	Metformin	40	40	17	18	54.5(7.6)	54.3(7.3)	newly diagnosed	newly diagnosed	8.23(0.93)	8.22(0.96)	100	1500	Low	Unclear	High	Low	Low	Unclear
Liao QH 2018 [166]	12 w	Diet, exercise	Vildagliptin+ Metformin	Metformin	75	75	33	35	46.25(7.36)	46.32(7.1)	4.28(2)	4.3(1.96)	8.22(1.54)	8.25(1.47)	100	2000	Low	Unclear	High	Low	Low	Unclear
Fu CM 2018 [167]	12 w	Diet, exercise	Vildagliptin+ Metformin	Metformin	65	65	31	32	59.6(13.2)	57.9(12.8)	0.5~8	0.5~6	8.21(1.45)	8.19(1.53)	100	1500	Unclear	Unclear	High	Low	Low	Unclear
Peng XH 2018 [168]	12 w	Diet, exercise	Vildagliptin+ Metformin	Metformin	43	43	17	18	58.6(2.3)	58.3(2.1)	nr	nr	8.25(1.19)	8.23(1.24)	100	2000	Unclear	Unclear	High	Low	Low	Unclear
Li SJ 2018 [169]	24 w	Diet, exercise	Vildagliptin+ Metformin	Metformin	38	38	11	13	49.29(2.67)	48.61(2.18)	nr	nr	11.51(1.92)	11.56(1.46)	100	1000	Low	Unclear	High	Low	Low	Unclear
Su JF 2018A [170]	12 w	Diet, exercise	Vildagliptin+ Metformin	Metformin	36	36	14	19	41.5(4.9)	42.3(5.1)	newly diagnosed	newly diagnosed	7.6(1.2)	7.7(1)	100	1500	Low	Unclear	High	Low	Low	Unclear
Zhao YH 2017 [171]	24 w	Diet, exercise	Vildagliptin+ Metformin	Metformin	60	60	30	31	46.5(6.2)	46.2(4.5)	4.5(1.5)	4.3(1.9)	9.31(1.53)	9(1.76)	100	2000	Low	Unclear	High	Low	Low	Unclear
Qu JC 2017A [172]	12 w	Diet, exercise	Vildagliptin+ Metformin	Metformin	40	40	19	22	61.5(9.2)	60.9(7.7)	newly diagnosed	newly diagnosed	8.53(1.11)	8.35(1.09)	100	1500	Unclear	Unclear	High	Low	Low	Unclear
Shi CL 2017 [173]	3 m	nr	Vildagliptin+ Metformin	Metformin	38	38	16	14	56.7(7.33)	56.68(7.31)	nr	nr	8.89(1.15)	8.9(1.16)	100	1500	Unclear	Unclear	High	Low	Low	Unclear
Li R 2016 [174]	12 w	Diet, exercise	Vildagliptin+ Metformin	Metformin	50	50	21	22	46.03(8.87)	45.73(9.23)	newly diagnosed	newly diagnosed	8.2(1.45)	8.23(1.56)	100	1500	Low	Unclear	High	Low	Low	Unclear
Zhao XW 2016A [175]	24 w	Diet, exercise	Vildagliptin+ Metformin	Metformin	50	50	23	24	48.8(8.6)	49.1(10.2)	nr	nr	8.91(1.16)	8.86(1.04)	100	2000	Low	Unclear	High	Low	Low	Unclear
Jia XW 2015 [176]	90 d	Diet	Vildagliptin+ Metformin	Metformin	87	85	44	44	48.8(8.3)	46.2(8)	0.57(0.39)	0.56(0.29)	7.94(0.91)	7.72(0.73)	100	1000	Unclear	Unclear	Low	Low	Low	Unclear

Zheng ZP 2015 [177]	3 m	Diet, exercise	Vildagliptin+ Metformin	Metformin	50	50	20	23	59.27(10.75)	56.55(10.04)	newly diagnosed	newly diagnosed	8.28(1.79)	8.21(1.93)	100	1500	Low	Unclear	High	Low	Low	Unclear
Liang CY 2015 [178]	36 w	nr	Vildagliptin+ Metformin	Metformin	42	42	12	22	52.2(6.4)	53.7(7.5)	nr	nr	9(1.6)	9(1.5)	100	1000	Low	Unclear	High	Low	Low	Unclear
Cheng LY 2014 [179]	12 w	Diet, exercise	Vildagliptin+ Metformin	Metformin	75	75	45	48	55.82(6.21)	55.76(6.2)	7.33(1.69)	7.24(1.66)	8.77(1.93)	8.8(1.92)	100	1000	Low	Unclear	High	Low	Low	Unclear
Chen XS 2014 [180]	6 m	nr	Vildagliptin+ Metformin	Metformin	60	60	nr	nr	64.38(2.09)	64.38(2.09)	7.29(1.59)	7.29(1.59)	9.77(1.67)	9.92(1.42)	100	1500	Low	Unclear	Low	Low	Low	Unclear
Yan ZD 2014 [181]	24 w	nr	Vildagliptin+ Metformin	Metformin	60	60	21	23	53.8(7.1)	52.4(6.7)	nr	nr	8.09(1.67)	8.21(1.87)	100	1500	Unclear	Unclear	High	Low	Low	Unclear
Lai KB 2014 [182]	12 w	Diet, exercise	Vildagliptin+ Metformin	Metformin	52	52	22	21	45.3(3.6)	45.5(3.2)	5.3(1.6)	5.4(1.3)	8.2(1.5)	8.21(1.42)	100	1500	Low	Unclear	High	Low	Low	Unclear
Li RH 2020 [183]	3 m	nr	Linagliptin+ Metformin	Metformin	48	48	23	21	50.18(6.52)	50.34(6.82)	5.35(3.24)	5.12(3.47)	8.63(1.74)	8.69(1.56)	5	1500	Low	Unclear	High	Low	Low	Unclear
Wang XP 2020 [184]	12 w	Diet, exercise	Linagliptin+ Metformin	Metformin	48	48	20	19	68.51(4.24)	68.53(4.23)	10.18(2.11)	10.15(2.08)	9.26(1.84)	9.18(1.92)	5	1500	Low	Unclear	High	Low	Low	Unclear
Zhou DQ 2020 [185]	12 w	Diet, exercise	Linagliptin+ Metformin	Metformin	30	30	13	12	nr	nr	< 0.25	< 0.25	9.78(2.56)	9.83(2.18)	5	1500	Unclear	Unclear	High	Low	Low	Unclear
Sun TT 2019 [186]	3 m	Diet, exercise	Linagliptin+ Metformin	Metformin	83	83	37	35	50.04(7.11)	49.38(6.92)	newly diagnosed	newly diagnosed	9.62(1.53)	9.54(1.37)	5	1500	Unclear	Unclear	High	Low	Low	Unclear
Chen KF 2019 [187]	3 m	nr	Linagliptin+ Metformin	Metformin	62	62	21	22	58.36(2.68)	59.71(2.81)	3.17(1.36)	3.06(1.29)	9.43(2.24)	9.45(2.17)	5	1500	Unclear	Unclear	High	Low	Low	Unclear
Guo CM 2019 [188]	12 w	nr	Linagliptin+ Metformin	Metformin	56	56	21	23	54.7(7.5)	54.4(7.2)	3.8(2.3)	3.9(2)	9.5(1.1)	9.3(1.3)	5	1500	Low	Unclear	High	Low	Low	Unclear
Bai FW 2019 [189]	12 w	nr	Linagliptin+ Metformin	Metformin	50	50	21	20	70.12(1.34)	70.99(1.65)	newly diagnosed	newly diagnosed	7.22(1.31)	7.26(1.67)	5	1500	Low	Unclear	High	Low	Low	Unclear
Niu S 2019 [190]	12 w	Diet, exercise	Linagliptin+ Metformin	Metformin	50	50	21	21	50.32(3.14)	50.32(3.14)	5.32(1.02)	5.32(1.02)	6.9(0.9)	6.8(1.2)	5	1500	Unclear	Unclear	High	Low	Low	Unclear

Jin J 2018 [191]	12 w	Diet, exercise	Linagliptin+ Metformin	Metformin	65	65	24	28	47.6(9.8)	45.2(8.4)	newly diagnosed	newly diagnosed	7.54(1.12)	7.61(0.85)	5	1500	Unclear	Unclear	Low	Low	Low	Unclear
Wang XW 2018 [192]	3 m	Diet, exercise	Linagliptin+ Metformin	Metformin	60	60	26	27	45~59	44~58	3.9(1.3)	3.8(1.2)	8.68(1.26)	8.62(1.45)	5	1950	Unclear	Unclear	Low	Low	Low	Unclear
Gao FD 2017 [193]	6 m	Diet, exercise	Linagliptin+ Metformin	Metformin	50	50	19	21	60.32(6.32)	59.65(6.79)	6.78(1.58)	6.32(1.38)	7.85(2.13)	7.89(2.05)	5	1500	Low	Unclear	High	Low	Low	Unclear
Zhao XW 2016B [194]	24 w	Diet, exercise	Linagliptin+ Metformin	Metformin	50	50	24	23	49.1(10.2)	48.8(8.6)	nr	nr	8.91(1.16)	8.86(1.04)	5	2000	Low	Unclear	High	Low	Low	Unclear
Zheng FP 2016a [195]	12 w	Diet, exercise	Linagliptin+ Metformin	Metformin	33	33	17	15	47(3.1)	48.5(2.9)	newly diagnosed	newly diagnosed	8.3(2.1)	8.1(2.6)	5	1000	Low	Unclear	High	Low	Low	Unclear
Chen P 2015b [130]	12 w	Diet, exercise	Linagliptin+ Metformin	Metformin	30	30	nr	nr	nr	nr	nr	nr	8.2(1.4)	8.1(1.4)	5	1500	Unclear	Unclear	High	Low	Low	Unclear
Wu SY 2020 [196]	24 w	nr	Alogliptin+ Metformin	Metformin	84	96	36	40	51.73(1.52)	52.73(1.42)	newly diagnosed	newly diagnosed	8.61(1.02)	8.46(1.35)	25	1500	Unclear	Unclear	High	Low	Low	Unclear
Cai YH 2020 [197]	6 m	nr	Alogliptin+ Metformin	Metformin	64	64	28	30	52.4(2.4)	52.6(2.3)	newly diagnosed	newly diagnosed	10.35(1.47)	10.22(1.52)	25	2000	Unclear	Unclear	High	Low	Low	Unclear
Zhang XT 2020 [198]	24 w	nr	Alogliptin+ Metformin	Metformin	40	40	17	18	58.64(6.83)	58.71(6.95)	newly diagnosed	newly diagnosed	8.27(0.85)	8.22(0.81)	25	2000	Low	Unclear	High	Low	Low	Unclear
Wei CW 2020 [199]	4 m	nr	Alogliptin+ Metformin	Metformin	35	35	17	16	62.48(3.76)	61.57(3.26)	6.8(1.6)	6.7(1.9)	8.05(0.42)	7.94(0.56)	25	1500	Low	Unclear	High	Low	Low	Unclear
Zheng ZG 2019 [200]	6 m	Diet, exercise	Alogliptin+ Metformin	Metformin	62	62	26	27	51.01(7.93)	50.24(7.65)	newly diagnosed	newly diagnosed	8.1(0.89)	8.23(0.91)	25	2000	Low	Unclear	High	Low	Low	Unclear
Qiao CF 2019 [201]	24 w	Diet, exercise	Alogliptin+ Metformin	Metformin	47	47	21	22	59.24(7.13)	59.61(7.28)	1.65(0.51)	1.69(0.53)	8.79(1.12)	8.4(1.2)	25	1500	Low	Unclear	High	Low	Low	Unclear
Weng Y 2016 [202]	20 w	Diet, exercise	Alogliptin+ Metformin	Metformin	50	50	nr	nr	nr	nr	nr	nr	8.9(3.3)	8.8(3.8)	25	1500	Unclear	Unclear	High	Low	Low	Unclear
Zheng FP 2016b [195]	12 w	Diet, exercise	Alogliptin+ Metformin	Metformin	33	33	14	15	49.3(2.6)	48.5(2.9)	newly diagnosed	newly diagnosed	8.4(2.5)	8.1(2.6)	25	1000	Low	Unclear	High	Low	Low	Unclear

Xie LH 2016 [203]	24 w	Diet, exercise	Rosiglitazone+ Metformin	Metformin	80	80	27	30	56.8(7.2)	55.3(6.8)	newly diagnosed	newly diagnosed	9.07(1.32)	8.93(1.23)	4	1500	Low	Unclear	High	Low	Low	Unclear
Li JY 2014 [204]	4 m	Diet, exercise	Rosiglitazone+ Metformin	Metformin	140	140	48	52	39~72	37~71	nr	nr	10.07(0.94)	11.08(0.75)	8	750	Unclear	Unclear	High	Low	Low	Unclear
Lei CY 2014 [205]	12 w	Diet, exercise	Rosiglitazone+ Metformin	Metformin	110	110	50	51	52.23(5.34)	52.18(5.33)	5.05(0.41)	5.02(0.36)	9.89(1.15)	9.93(1.05)	8	1500	Unclear	Unclear	High	Low	Low	Unclear
Yang XD 2013 [206]	16 w	nr	Rosiglitazone+ Metformin	Metformin	75	75	34	35	53.46(1.78)	53.12(1.75)	nr	nr	8.65(2.24)	8.73(2.18)	4	1500	Unclear	Unclear	High	Low	Low	Unclear
Yu CY 2013 [207]	16 w	Diet, exercise	Rosiglitazone+ Metformin	Metformin	60	60	31	30	53.46(1.58)	54.41(1.67)	<2	<2	8.65(2.24)	8.73(2.18)	4	1500	Unclear	Unclear	High	Low	Low	Unclear
Gao Y 2013 [208]	16 w	Diet, exercise	Rosiglitazone+ Metformin	Metformin	45	45	24	25	53.4(1.8)	54.1(1.7)	nr	nr	8.6(2.2)	8.7(2.2)	4	1500	Unclear	Unclear	High	Low	Low	Unclear
Liu M 2012 [209]	12 w	Diet, exercise	Rosiglitazone+ Metformin	Metformin	40	40	9	6	63.99(2.78)	63.77(3.23)	nr	nr	7.82(1.9)	7.57(1.67)	4	1500	Unclear	Unclear	High	Low	Low	Unclear
Li YG 2011 [210]	3 m	Diet, exercise	Rosiglitazone+ Metformin	Metformin	30	30	nr	nr	32~65	32~65	0.5~1	0.5~1	7.9(0.6)	7.1(0.8)	4	500	Unclear	Unclear	High	Low	Low	Unclear
Li GH 2010 [211]	24 w	Diet, exercise	Rosiglitazone+ Metformin	Metformin	60	60	nr	nr	43(6)	43(6)	7(4)	7(4)	8.8(1.5)	8.7(1.1)	4	750	Unclear	Unclear	High	Low	Low	Unclear
Xia ZX 2009 [212]	24 w	Diet, exercise	Rosiglitazone+ Metformin	Metformin	60	60	29	33	58.5	59.6	9.4	10.2	11.2(1.4)	11.8(1)	4	1000	Unclear	Unclear	High	Low	Low	Unclear
Zhong FD 2009 [213]	12 w	Diet, exercise	Rosiglitazone+ Metformin	Metformin	36	36	18	19	44.67(9.73)	46.39(10.13)	newly diagnosed	newly diagnosed	8.86(0.98)	9.1(1.08)	4	500	Unclear	Unclear	High	Low	Low	Unclear
Xie XM 2020 [214]	6 m	Diet, exercise	Pioglitazone+ Metformin	Metformin	75	75	36	35	56.31(3.82)	57.25(3.56)	4.7(1.1)	4.8(1.2)	8.5(1.2)	8.6(1.4)	30	750	Low	Unclear	High	Low	Low	Unclear
Di JN 2020 [215]	16 w	Diet	Pioglitazone+ Metformin	Metformin	57	57	nr	nr	49(6.3)	49(6.3)	5.2(3.5)	5.2(3.5)	8.93(1.31)	8.83(1.21)	15	1500	Low	Unclear	High	Low	Low	Unclear
Cao WF 2020 [216]	3 m	nr	Pioglitazone+ Metformin	Metformin	50	50	14	16	51.56(2.34)	51.21(2.17)	8.51(0.45)	8.52(0.61)	9.24(0.45)	9.28(0.48)	15	750	Unclear	Unclear	High	Low	Low	Unclear

Shi YQ 2020 [217]	6 m	Diet, exercise	Pioglitazone+ Metformin	Metformin	31	31	13	12	61.55(4.94)	60.36(4.86)	6.45(0.48)	6.33(0.49)	9.41(2.62)	9.52(2.68)	20	1000	Low	Unclear	High	Low	Low	Unclear
Zhang H 2019 [218]	6 m	nr	Pioglitazone+ Metformin	Metformin	100	100	49	47	56.99(3.19)	57.83(3.21)	1.28(0.51)	1.32(0.53)	8.61(2.05)	8.73(1.93)	15	1500	Unclear	Unclear	High	Low	Low	Unclear
Hu KY 2019 [219]	6 m	Diet, exercise	Pioglitazone+ Metformin	Metformin	68	68	28	31	55.8(8.1)	54.6(8.7)	>0.5	>0.5	8.1(0.9)	8.8(0.7)	30	750	Unclear	Unclear	High	Low	Low	Unclear
Wang HJ 2019 [220]	3 m	Diet, exercise	Pioglitazone+ Metformin	Metformin	46	46	21	22	48.69(6.72)	49.38(6.21)	5.76(3.58)	5.69(3.75)	9.28(1.32)	9.31(1.27)	30	1000	Unclear	Unclear	High	Low	Low	Unclear
Gao SW 2019 [221]	6 m	Diet, exercise	Pioglitazone+ Metformin	Metformin	40	40	15	16	41.5(3.5)	42.5(2.5)	4.2(1.2)	3.5(1.5)	8.2(1)	8.1(1.1)	30	1500	Unclear	Unclear	High	Low	Low	Unclear
Guo H 2018 [222]	6 m	nr	Pioglitazone+ Metformin	Metformin	45	45	22	20	55.09(6.56)	54.36(6.32)	7.56(2.74)	7.14(2.21)	8.72(0.78)	8.69(0.72)	20	1500	Low	Unclear	High	Low	Low	Unclear
Chen GM 2018 [223]	6 m	nr	Pioglitazone+ Metformin	Metformin	39	39	nr	nr	57(5)	58(6)	2.25(0.42)	2.33(0.33)	8.69(2.03)	8.73(1.95)	15	1500	Unclear	Unclear	High	Low	Low	Unclear
Ren BX 2018 [224]	6 m	nr	Pioglitazone+ Metformin	Metformin	37	37	14	16	58.29(4.21)	58.36(4.17)	2.15(0.5)	2.1(0.53)	8.69(1.12)	8.74(1.07)	15	1500	Low	Unclear	Low	Low	Low	Unclear
Li SQ 2016 [225]	3 m	nr	Pioglitazone+ Metformin	Metformin	56	56	28	29	54.3(13.8)	53.7(13.1)	7.7(3.5)	8.1(3)	9.1(2.3)	9.3(2.1)	15	1000	Low	Unclear	High	Low	Low	Unclear
Zhang CR 2016 [226]	3 m	Diet, exercise	Pioglitazone+ Metformin	Metformin	55	55	24	23	63.6(5.9)	63.7(5.8)	8.3(5.7)	8.1(5.6)	7.5(2.3)	7.4(2.2)	30	1500	Unclear	Unclear	High	Low	Low	Unclear
Zhu XX 2015 [227]	6 m	nr	Pioglitazone+ Metformin	Metformin	80	80	37	35	57.9(3.5)	58.3(3.3)	nr	nr	8.5(1.9)	8.5(1.8)	20	1500	Unclear	Unclear	High	Low	Low	Unclear
Wang T 2015 [228]	6 m	Diet, exercise	Pioglitazone+ Metformin	Metformin	77	75	37	36	55.3(6.5)	55.8(7.1)	nr	nr	8.5(1.2)	8.6(1.5)	30	750	Unclear	Unclear	High	Low	Low	Unclear
Ma ZX 2015 [229]	6 m	Diet, exercise	Pioglitazone+ Metformin	Metformin	70	70	23	20	55.6(7.2)	54.9(6.6)	9.04(1.52)	8.96(1.47)	8.14(0.94)	8.08(0.77)	30	750	Low	Unclear	High	Low	Low	Unclear
Zhou YQ 2015 [230]	3 m	Diet, exercise	Pioglitazone+ Metformin	Metformin	65	65	nr	nr	56.4(5.2)	56.4(5.2)	8.9(3.1)	8.9(3.1)	8.7(0.9)	8.5(1.1)	15	1500	Low	Unclear	High	Low	Low	Unclear

Zhang CH 2015 [231]	3 m	nr	Pioglitazone+ Metformin	Metformin	40	40	16	17	46.4(1.6)	46.3(1.7)	7.4(1.5)	7.2(1.3)	9.87(1.25)	9.89(1.24)	20	1500	Low	Unclear	High	Low	Low	Unclear
Ren YL 2015 [232]	16 w	nr	Pioglitazone+ Metformin	Metformin	35	35	13	14	54.7(5.5)	54.3(5.8)	4.6(1.4)	4.7(1.2)	8.25(2.11)	8.19(2.07)	30	1000	Low	Unclear	High	Low	Low	Unclear
Yang HM 2015 [233]	6 m	Diet, exercise	Pioglitazone+ Metformin	Metformin	30	30	nr	nr	35~65	35~65	newly diagnosed	newly diagnosed	8.7(2.1)	8.5(1.9)	30	750	Unclear	Unclear	High	Low	Low	Unclear
Shen CM 2014 [234]	6 m	nr	Pioglitazone+ Metformin	Metformin	102	102	39	47	56.3(9.9)	58.2(10.4)	5.5(1.9)	5.3(1.9)	9.01(1.32)	9.19(1.51)	20	1500	Unclear	Unclear	High	Low	Low	Unclear
Du YJ 2014 [235]	6 m	Diet, exercise	Pioglitazone+ Metformin	Metformin	72	72	29	31	54.32(4.71)	54.92(4.81)	nr	nr	8.78(0.61)	8.89(0.59)	30	750	Unclear	Unclear	High	Low	Low	Unclear
Zhang Y 2013 [236]	3 m	nr	Pioglitazone+ Metformin	Metformin	75	75	33	33	71.23(10.32)	71.23(10.32)	7.31(1.12)	7.31(1.12)	8.59(1.56)	8.64(1.64)	30	1000	Unclear	Unclear	High	Low	Low	Unclear
Bi XS 2013 [237]	16 w	nr	Pioglitazone+ Metformin	Metformin	60	60	nr	nr	nr	nr	nr	nr	8.8(2)	9.1(2.3)	25	1500	Low	Unclear	High	Low	Low	Unclear
Zhong J 2013 [238]	12 w	nr	Pioglitazone+ Metformin	Metformin	30	30	16	13	61.2(7.1)	61.2(7.1)	12.1(3.2)	12.1(3.2)	6.84(0.26)	6.79(0.17)	30	750	Unclear	Unclear	High	Low	Low	Unclear
Jiang H 2012 [239]	6 m	nr	Pioglitazone+ Metformin	Metformin	113	114	nr	nr	nr	nr	nr	nr	8.7(2)	8.8(1.9)	20	1500	Unclear	Unclear	High	Low	Low	Unclear
Zhu YF 2012 [240]	12 w	nr	Pioglitazone+ Metformin	Metformin	67	67	31	30	55.7(5.5)	56.5(4.8)	nr	nr	8.7(2)	8.8(1.9)	30	1500	Unclear	Unclear	High	Low	Low	Unclear
Teng YP 2012 [241]	6 m	Diet, exercise	Pioglitazone+ Metformin	Metformin	50	50	20	24	44(6.4)	45(7.8)	nr	nr	8.47(1.42)	8.59(1.2)	15	1000	Unclear	Unclear	High	Low	Low	Unclear
Yang YB 2012B [242]	3 m	Diet, exercise	Pioglitazone+ Metformin	Metformin	42	42	nr	nr	50.2(3.4)	50.2(3.4)	0.5~11	0.5~11	8.7(0.8)	8.4(0.9)	30	1500	Unclear	Unclear	High	Low	Low	Unclear
Guo HF 2011 [243]	6 m	Diet, exercise	Pioglitazone+ Metformin	Metformin	120	120	58	59	54.2	55.3	nr	nr	8.92(1.54)	8.58(1.21)	30	1500	Unclear	Unclear	High	Low	Low	Unclear
Hong YJ 2010 [244]	6 m	nr	Pioglitazone+ Metformin	Metformin	51	51	25	22	52.1(13.6)	53.7(14.3)	nr	nr	8.73(1.72)	8.71(1.71)	30	1500	Unclear	Unclear	High	Low	Low	Unclear

Deng JH 2009 [245]	3 m	Diet, exercise	Pioglitazone+ Metformin	Metformin	40	40	nr	nr	nr	nr	0.5~1	0.5~1	9.7(1.3)	9.4(1.2)	30	1500	Unclear	Unclear	High	Low	Low	Unclear
Li L 2020 [246]	12 w	nr	Dapagliflozin+ Metformin	Metformin	85	82	39	37	63.26(5.18)	62.35(4.17)	6.23(1.52)	5.16(1.27)	9.43(0.94)	9.57(0.87)	10	1500	Low	Unclear	High	Low	Low	Unclear
Song QQ 2020 [247]	16 w	Diet, exercise	Dapagliflozin+ Metformin	Metformin	50	50	26	28	35.5(4.3)	34.4(4.9)	1.5(0.5)	1.5(0.5)	8.5(1)	8.4(1.2)	10	2000	Unclear	Unclear	High	Low	Low	Unclear
Yang Y 2020 [248]	3 m	Diet, exercise	Dapagliflozin+ Metformin	Metformin	50	50	23	26	55.98(6.77)	54.42(8.37)	7.28(5.1)	6.7(4.92)	8.99(0.64)	9.1(0.62)	10	1500	Low	Unclear	High	Low	Low	Unclear
Li JS 2020A [249]	3 m	nr	Dapagliflozin+ Metformin	Metformin	44	44	19	17	40.81(3.85)	40.36(3.94)	2.23(1.04)	2.12(1.15)	9.43(1.29)	9.42(1.28)	10	1500	Unclear	Unclear	High	Low	Low	Unclear
Ren HX 2020 [250]	12 w	nr	Dapagliflozin+ Metformin	Metformin	35	35	16	17	61.6(1.2)	61.5(1.3)	7.8(0.3)	7.7(0.4)	8.53(1.18)	8.52(1.19)	10	1500	Low	Unclear	High	Low	Low	Unclear
Wang Y 2020A [251]	3 m	Diet	Dapagliflozin+ Metformin	Metformin	35	35	16	14	46.94(6.42)	46.52(6.31)	5.34(0.61)	5.11(0.72)	8.62(1.21)	8.57(1.34)	10	1500	Unclear	Unclear	High	Low	Low	Unclear
Liu XJ 2019 [252]	3 m	Diet, exercise	Dapagliflozin+ Metformin	Metformin	44	44	15	19	55(8.5)	55(8.2)	10.02(5.25)	10.05(5.22)	8.6(1.4)	8.5(1.24)	10	1000	Low	Unclear	High	Low	Low	Unclear
Xu J 2019 [253]	16 w	Diet, exercise	Dapagliflozin+ Metformin	Metformin	40	40	18	22	42.8(11.2)	43.2(10.5)	newly diagnosed	newly diagnosed	8.89(1.61)	8.88(1.27)	10	1500	Low	Unclear	High	Low	Low	Low
Liu XY 2020 [254]	16 w	Diet, exercise	Empagliflozin+ Metformin	Metformin	40	40	11	12	47.9(11.6)	48.1(12.2)	newly diagnosed	newly diagnosed	8.8(1.7)	8.9(2.1)	10	1500	Low	Unclear	High	Low	Low	Low
Liu SX 2020 [255]	12 w	nr	Empagliflozin+ Metformin	Metformin	40	40	21	22	50.14(8.64)	49.19(12.19)	nr	nr	7.9(0.7)	8(0.7)	10	2000	Unclear	Unclear	Low	Low	Low	Unclear
He ML 2020 [256]	12 w	Diet, exercise	Empagliflozin+ Metformin	Metformin	31	31	15	14	52.6(3.5)	52.5(3.6)	5.5(1.5)	5.6(1.3)	8.6(1.5)	8.4(1.4)	10	1700	Unclear	Unclear	High	Low	Low	Unclear
Wei ZX 2018 [257]	12 w	Diet, exercise	Exenatide+ Metformin	Metformin	60	60	28	26	47.79(3.74)	47.96(3.56)	nr	nr	8.15(0.69)	8.23(0.74)	Initial:0.01; 1 w later:0.02	1500	Low	Unclear	High	Low	Low	Unclear
Hou XL 2018 [258]	24 w	nr	Exenatide+ Metformin	Metformin	40	40	13	15	48(6.2)	49(6.8)	7(1.2)	6.8(1)	11.2(1.1)	11.6(1.2)	Initial:0.01; 4 w later:0.02	1700	Low	Unclear	High	Low	Low	Unclear

Zhou RF 2017 [259]	12 w	nr	Exenatide+ Metformin	Metformin	30	30	16	17	48.4(12.4)	49.1(12)	newly diagnosed	newly diagnosed	7.6(0.6)	7.6(0.6)	0.01	1000	Unclear	Unclear	High	Low	Low	Low
Li BW 2015 [260]	16 w	nr	Exenatide+ Metformin	Metformin	128	128	60	57	52.14(5.23)	53.07(5.46)	newly diagnosed	newly diagnosed	8.36(1.15)	8.27(1.34)	Initial:0.01; 8 w later:0.02	1000	Unclear	Unclear	High	Low	Low	Unclear
Pan NN 2014 [261]	3 m	Diet, exercise	Exenatide+ Metformin	Metformin	30	30	nr	nr	60~85	60~85	0.25	0.25	7.5(0.9)	7.5(1.2)	Initial:0.01; 1 m later:0.02	1500	Unclear	Unclear	High	Low	Low	Unclear
Li JS 2020B [262]	12 w	Diet, exercise	Liraglutide+ Metformin	Metformin	80	80	31	29	44.51(5.01)	43.62(4.98)	nr	nr	9.36(1.09)	9.26(1.11)	0.6	1500	Low	Unclear	High	Low	Low	Unclear
Liu YD 2020 [263]	4 m	nr	Liraglutide+ Metformin	Metformin	79	78	36	37	51.61(5.21)	51.23(5.74)	nr	nr	9.95(1.56)	9.97(1.52)	0.6	1500	Unclear	Unclear	High	Low	Low	Unclear
Lin Y 2020 [264]	3 m	nr	Liraglutide+ Metformin	Metformin	60	60	26	24	72.13(5.74)	72.69(4.35)	6.97(1.46)	7.32(1.78)	10.64(1.32)	10.89(1.25)	Initial:1.2; Max:1.8	1500	Low	Unclear	High	Low	Low	Unclear
Yang M 2020 [265]	4 m	Diet, exercise	Liraglutide+ Metformin	Metformin	53	53	32	31	54.3(2.4)	53.7(2.6)	1~15	1~14	14.51(4.11)	14.36(3.98)	1.2	1500	Unclear	Unclear	High	Low	Low	Unclear
Jia C 2020 [266]	12 w	nr	Liraglutide+ Metformin	Metformin	45	45	nr	nr	60.4(3.5)	60.4(3.5)	nr	nr	9.8(1.3)	9.9(1.1)	Initial:0.6; 4 w later:1.2	1500	Low	Unclear	High	Low	Low	Unclear
Liu WF 2020 [267]	3 m	nr	Liraglutide+ Metformin	Metformin	45	45	21	20	57.13(14.29)	58.34(15.49)	3.49(1.07)	3.52(1.04)	8.78(1.26)	8.79(1.24)	0.6	2000	Low	Unclear	High	Low	Low	Unclear
Liu HY 2020 [268]	3 m	Diet, exercise	Liraglutide+ Metformin	Metformin	43	43	20	21	52.62(5.35)	51.46(6.31)	3.23(0.62)	3.27(0.75)	8.73(0.71)	8.62(0.84)	0.6	1000	Low	Unclear	High	Low	Low	Low
Guo W 2020a [269]	26 w	Diet, exercise	Liraglutide+ Metformin	Metformin	31	30	15	10	53.1(6.3)	52.6(3.9)	nr	nr	7.5(1.3)	7.4(1)	Initial:0.6; Max:1.8	2000	Low	Low	Low	Low	Low	Low
Lai XY 2020 [270]	24 w	nr	Liraglutide+ Metformin	Metformin	30	30	14	16	41.4(3.2)	42.3(3.1)	<0.5	<0.5	8.3(1.2)	8.2(0.9)	Initial:0.6; 3 w:1.2	1500	Low	Unclear	High	Low	Low	Unclear
Wang YQ 2019 [271]	16 w	Diet, exercise	Liraglutide+ Metformin	Metformin	80	80	37	36	61.7(13.6)	62.8(14.4)	0.5~8	0.5~10	9.2(2)	9.3(2.2)	Initial:0.6; 2 w:1.2	1500	Unclear	Unclear	High	Low	Low	Unclear
Li LB 2019 [272]	3 m	Diet, exercise	Liraglutide+ Metformin	Metformin	65	65	30	32	56.86(4.23)	56.35(4.17)	8.31(1.98)	8.49(2.01)	8.8(2.18)	8.72(2.13)	Initial:0.6; 2 w:1.2	2000	Low	Unclear	High	Low	Low	Unclear

Chen CX 2019 [273]	16 w	nr	Liraglutide+ Metformin	Metformin	54	54	22	25	41.25(4.53)	41.19(4.55)	1.07(0.29)	1.07(0.29)	10.02(1.04)	9.98(1.03)	0.6	2000	Unclear	Unclear	High	Low	Low	Unclear
Wu J 2019 [274]	3 m	nr	Liraglutide+ Metformin	Metformin	52	52	21	23	61.7(5.2)	61.3(5.6)	6.2(0.5)	6.4(0.8)	9.83(1.04)	9.76(1.52)	1.8	1500	Unclear	Unclear	High	Low	Low	Unclear
Wang LY 2019 [275]	3 m	nr	Liraglutide+ Metformin	Metformin	48	48	21	22	59.4(10.5)	58.7(10.9)	nr	nr	8.96(0.48)	8.89(0.51)	Initial:0.6; 2 w:1.2; 3 w:1.8	1000	Unclear	Unclear	High	Low	Low	Unclear
Shi GL 2019 [276]	12 w	nr	Liraglutide+ Metformin	Metformin	39	39	19	17	53(10.5)	52.3(10.8)	9.5(3.2)	9.5(3)	8.12(1.23)	8.2(1.3)	Initial:0.6; 2 w:1.2; Max:1.8	2000	Low	Unclear	High	Low	Low	Unclear
Zu Q 2019 [277]	12 w	nr	Liraglutide+ Metformin	Metformin	35	35	15	13	45.26(3.17)	44.83(3.82)	nr	nr	10.15(1.26)	9.97(1.12)	0.6	2000	Unclear	Unclear	High	Low	Low	Unclear
Ye XX 2019 [278]	4 m	nr	Liraglutide+ Metformin	Metformin	31	31	9	10	47.46(6.89)	47.41(6.85)	newly diagnosed	newly diagnosed	8.79(0.86)	8.68(0.72)	0.6	2000	Low	Unclear	High	Low	Low	Unclear
Cheng XF 2019 [279]	3 m	nr	Liraglutide+ Metformin	Metformin	31	31	12	14	63.89(5.43)	63.58(5.39)	7.06(3.61)	6.89(3.71)	8.41(0.61)	8.26(0.67)	Initial:0.6; 2 w:1.2; Max:1.8	1500	Unclear	Unclear	High	Low	Low	Unclear
Jian XH 2018 [280]	3 m	nr	Liraglutide+ Metformin	Metformin	58	58	26	29	56.51(6.05)	56.33(8.63)	nr	nr	8.7(1.04)	8.88(1.24)	Initial:0.6; 2 w:1.2	2000	Unclear	Unclear	High	Low	Low	Unclear
Wang XY 2018 [281]	3 m	Diet, exercise	Liraglutide+ Metformin	Metformin	46	46	21	22	55.01(10.46)	54.51(10.32)	nr	nr	9.24(2.79)	9.36(2.64)	0.6~1.2	1500	Low	Unclear	High	Low	Low	Unclear
Yang XQ 2018 [282]	16 w	nr	Liraglutide+ Metformin	Metformin	45	45	27	28	51.71(2.19)	51.56(2.19)	12.51(0.21)	12.32(0.24)	11.13(1.11)	11.14(1.24)	0.6	2000	Low	Unclear	High	Low	Low	Unclear
Tang XY 2018 [283]	3 m	Diet, exercise	Liraglutide+ Metformin	Metformin	40	40	17	19	42.86(2.3)	44.25(1.69)	<2	<2	9.68(1.37)	10.15(1.4)	0.6	2000	Low	Unclear	High	Low	Low	Unclear
Su JF 2018B [284]	12 w	Diet, exercise	Liraglutide+ Metformin	Metformin	38	38	16	14	53(8)	52(8)	5.7(0.7)	5.8(0.7)	8.1(1.2)	8(1.1)	Initial:0.6; Max:1.8	1500	Low	Unclear	High	Low	Low	Unclear
Ma Y 2018 [285]	3 m	Diet, exercise	Liraglutide+ Metformin	Metformin	37	37	15	16	52.75(8.62)	51.46(9.12)	nr	nr	9.25(1.18)	8.79(1.21)	Initial:0.6; 2 w:1.2	1500	Low	Unclear	High	Low	Low	Unclear
Zhao PY 2018 [286]	3 m	nr	Liraglutide+ Metformin	Metformin	35	35	15	16	55.6(1.9)	55.3(1.8)	nr	nr	9.25(2.71)	9.28(2.78)	0.6~1.8	1500	Unclear	Unclear	High	Low	Low	Unclear

He XY 2018 [287]	3 m	nr	Liraglutide+ Metformin	Metformin	34	34	13	11	57.84(6.41)	58.29(6.13)	7.85(2.44)	8.17(2.29)	8.34(1.32)	8.16(0.84)	Initial:0.6; 2 w:1.2	1000	Low	Unclear	High	Low	Low	Unclear
Zhang L 2017 [288]	16 w	nr	Liraglutide+ Metformin	Metformin	50	50	19	18	56.25(2.7)	56.67(2.71)	4.51(2.67)	4.56(2.62)	11.15(1.92)	11.21(1.41)	Initial:0.6; 3 w: 1.8	1700	Low	Unclear	High	Low	Low	Unclear
Tu CF 2017 [289]	4 m	nr	Liraglutide+ Metformin	Metformin	49	49	19	17	69.5(3.1)	70.7(3.3)	nr	nr	8.79(0.86)	8.82(0.85)	0.6	2000	Unclear	Unclear	High	Low	Low	Unclear
Si M 2017 [290]	12 w	Diet	Liraglutide+ Metformin	Metformin	36	40	nr	nr	39.62(3.12)	39.62(3.12)	newly diagnosed	newly diagnosed	9.68(1.34)	9.55(1.18)	Initial:0.6; Max:1.8	2000	Low	Unclear	High	Low	Low	Unclear
Qu JC 2017B [291]	24 w	nr	Liraglutide+ Metformin	Metformin	30	30	nr	nr	nr	nr	<0.25	<0.25	8.3(1.3)	8.1(0.9)	Initial:0.6; 3 w:1.2	1500	Unclear	Unclear	High	Low	Low	Unclear
Yang QF 2016b [42]	15 w	nr	Liraglutide+ Metformin	Metformin	140	70	65	34	58.12(11.82)	57.39(12.24)	nr	nr	9.23(2.8)	9.37(2.65)	1.8	2000	Unclear	Unclear	High	Low	Low	Unclear
Wang CX 2016 [292]	24 w	Diet, exercise	Liraglutide+ Metformin	Metformin	81	81	nr	nr	44.52(9.37)	45.45(7.62)	nr	nr	7.72(1.95)	7.35(1.57)	Initial:0.6, 2 w:1.2	1500	Unclear	Unclear	High	Low	Low	Unclear
Song WR 2016 [293]	3 m	nr	Liraglutide+ Metformin	Metformin	53	53	19	20	38.7(4.9)	38.2(5.1)	nr	nr	10.7(2.01)	10.69(1.93)	0.6	2000	Unclear	Unclear	High	Low	Low	Unclear
Xue GF 2016 [294]	16 w	nr	Liraglutide+ Metformin	Metformin	49	49	20	22	52.4(5.2)	53.3(5.8)	5.7(1.3)	6.1(1.8)	10.2(1.7)	9.6(1.5)	0.6	2000	Low	Unclear	High	Low	Low	Unclear
Ma CH 2016b [44]	12 w	Diet, exercise	Liraglutide+ Metformin	Metformin	30	30	13	14	45(10)	44(10)	5(3)	5(3)	8(0.4)	8.1(0.4)	Initial:0.6~1.2; Max:1.8	1500	Unclear	Unclear	High	Low	Low	Unclear
Yao YN 2015 [295]	24 w	Diet, exercise	Liraglutide+ Metformin	Metformin	66	66	11	13	45.31(10.35)	43.88(12.66)	nr	nr	7.72(1.95)	7.61(1.97)	Initial:0.6; 2 w:1.2	1500	Low	Unclear	High	Low	Low	Unclear
Liu CL 2015 [296]	3 m	Diet, exercise	Liraglutide+ Metformin	Metformin	60	60	31	35	55.34(9.58)	53.33(10.31)	nr	nr	10.21(1.35)	10.18(1.13)	Initial:0.6; 2 w:1.2	1500	Low	Unclear	High	Low	Low	Unclear
Zhao FL 2015 [297]	16 w	nr	Liraglutide+ Metformin	Metformin	44	44	20	21	55.08(8.21)	53.76(6.76)	nr	nr	8.78(0.87)	8.67(0.73)	0.6	2000	Low	Unclear	High	Low	Low	Unclear
Wang Y 2020B [298]	3 m	nr	Insulin glargine+ Metformin	Metformin	50	50	22	24	47.82(4.77)	47.87(4.78)	nr	nr	8.19(0.45)	8.22(0.5)	Initial: 10U; later: 0.2U/kg·d; adjust based on fasting plasma glucose	1000	Low	Unclear	High	Low	Low	Unclear

Wang F 2020 [299]	12 w	Diet, exercise	Insulin glargine+ Metformin	Metformin	40	40	17	19	55.34(7.1)	56.24(6.93)	8.33(2.56)	7.12(2.68)	9.81(2.24)	9.79(2.31)	Initial:0.2U/kg; add 2U every 3 d	1500	High	Unclear	High	Low	Low	Unclear
Zeng CM 2020 [300]	3 m	nr	Insulin glargine+ Metformin	Metformin	44	36	22	16	69.85(7.15)	69.5(6.85)	nr	nr	10.44(1.86)	10.22(1.25)	Initial: 6U~8U	500	Low	Unclear	High	Low	Low	Unclear
Guo W 2020b [269]	26 w	Diet, exercise	Insulin glargine+ Metformin	Metformin	30	30	12	10	52(8.7)	52.6(3.9)	nr	nr	7.4(0.9)	7.4(1)	10U; add 1 U/d to get fasting plasma glucose <7 mmol/l	2000	Low	Low	Low	Low	Low	Low
Li ZZ 2019 [301]	3 m	nr	Insulin glargine+ Metformin	Metformin	53	53	23	25	69.87(3.74)	70.08(3.67)	4.21(1.35)	4.16(1.4)	10.31(2.62)	10.25(2.64)	10U/d	1500	Unclear	Unclear	High	Low	Low	Unclear
Chang J 2019 [302]	3 m	Diet, exercise	Insulin glargine+ Metformin	Metformin	44	44	20	19	62.25(2.78)	62.36(2.84)	2.92(0.78)	2.95(0.74)	9.36(0.91)	9.38(0.98)	10U/d	1500	Low	Unclear	High	Low	Low	Unclear
Zhang WH 2015 [303]	3 m	Diet, exercise	Insulin glargine+ Metformin	Metformin	54	54	23	21	51.92(3.44)	51.54(3.51)	newly diagnosed	newly diagnosed	8.97(1.88)	9.01(1.86)	Initial: 0.2U/kg·d; adjust 2~4U every 3d based on fasting plasma glucose	1500	Low	Unclear	High	Low	Low	Unclear
Liu SJ 2015 [304]	3 m	nr	Insulin glargine+ Metformin	Metformin	50	50	28	26	51.01(8.12)	53.12(8.11)	5.6(1.89)	5.68(2.01)	10.71(2.32)	10.31(2.53)	Initial: 0.2U/kg·d; adjust based on blood glucose	1000	Unclear	Unclear	High	Low	Low	Unclear
Ma L 2015 [305]	3 m	nr	Insulin glargine+ Metformin	Metformin	48	48	25	26	53.7(3.8)	54.3(3.4)	6.6(1.9)	6.8(2.1)	10.9(2.5)	10.7(2.3)	Initial: 0.2U/kg·d; adjust based on blood glucose	1000	Unclear	Unclear	High	Low	Low	Unclear
Li M 2015 [306]	12 w	nr	Insulin glargine+ Metformin	Metformin	47	47	20	18	48.2(8.5)	47.6(7.3)	5.4(1.2)	5.6(1.5)	10.4(1.8)	10.1(2.7)	Initial: 10U/d; adjust based on blood glucose	1500	Unclear	Unclear	High	Low	Low	Unclear
He RY 2015 [307]	12 w	Diet	Insulin glargine+ Metformin	Metformin	39	39	19	20	39(4.5)	38(4.7)	nr	nr	9.35(1.95)	9.36(1.76)	0.15U/kg·d	1500	Unclear	Unclear	High	Low	Low	Unclear
Ma YM 2015 [308]	16 w	Diet, exercise	Insulin glargine+ Metformin	Metformin	33	33	16	13	59(11)	63(10)	nr	nr	9.6(2.6)	10.1(1.8)	Initial:0.2U/kg·d ; adjust based on blood glucose	1500	Unclear	Unclear	High	Low	Low	Unclear
He YF 2014 [309]	3 m	Diet, exercise	Insulin glargine+ Metformin	Metformin	40	40	13	15	67.5(5.2)	68.4(5.5)	6.8(1.8)	7.2(2.2)	9.7(1.7)	9.7(1.3)	Initial: 10U; adjust 2U/ time based on blood glucose	1500	Unclear	Unclear	High	Low	Low	Unclear
Liu GH 2018 [310]	12 w	nr	Insulin glargine+ Metformin	Metformin	56	56	26	24	65.22(1.24)	65.23(1.23)	7.56(0.04)	7.54(0.45)	8.54(1.35)	8.54(1.36)	Initial: 8U; adjust based on blood glucose	1500	Unclear	Unclear	High	Low	Low	Unclear
Lin JB 2019 [311]	6 m	nr	Insulin+ Metformin	Metformin	91	91	45	46	58.3(2.2)	58.5(2.1)	<1	<1	10.99(0.99)	11.12(0.91)	Initial: 0.2U/kg·d	1000	Low	Unclear	High	Low	Low	Unclear

Li RL 2014 [312]	12 w	Diet, exercise	Insulin+ Metformin	Metformin	50	50	nr	nr	59.53(10.23)	59.53(10.23)	8.52(1.6)	8.52(1.6)	10.4(1.28)	10.35(1.32)	Initial: 8U; adjust based on blood glucose	1500	Low	Unclear	High	Low	Low	Unclear
Ding JP 2019 [313]	12 w	nr	Insulin+ Metformin	Metformin	38	38	17	18	60.5(0.4)	60.3(0.6)	nr	nr	10.14(1.17)	10.2(1.09)	0.2~0.3U/kg·d; adjust based on blood glucose	750	Low	Unclear	High	Low	Low	Unclear
Liu J 2019 [314]	12 w	nr	Insulin+ Metformin	Metformin	48	42	18	22	42.08(9.34)	43.12(8.1)	newly diagnosed	newly diagnosed	9.45(1.64)	9.86(1.32)	0.3~0.4U/kg	2000	Low	Unclear	High	Low	Low	Unclear
Cao YQ 2014 [315]	12 w	Diet, exercise	Insulin+ Metformin	Metformin	40	40	16	18	54.4(9.8)	56.6(10.2)	8~17	7~19	9.05(0.62)	9.11(0.74)	Initial: morning 15 U/d, evening 10 U/d; Max: morning 40 U/d, evening 30 U/d	1500	Unclear	Unclear	High	Low	Low	Unclear

C, comparison. d, day. HbA1c, hemoglobin Alc. I, intervention. m, month. nr, not reported. w, week.

Figures

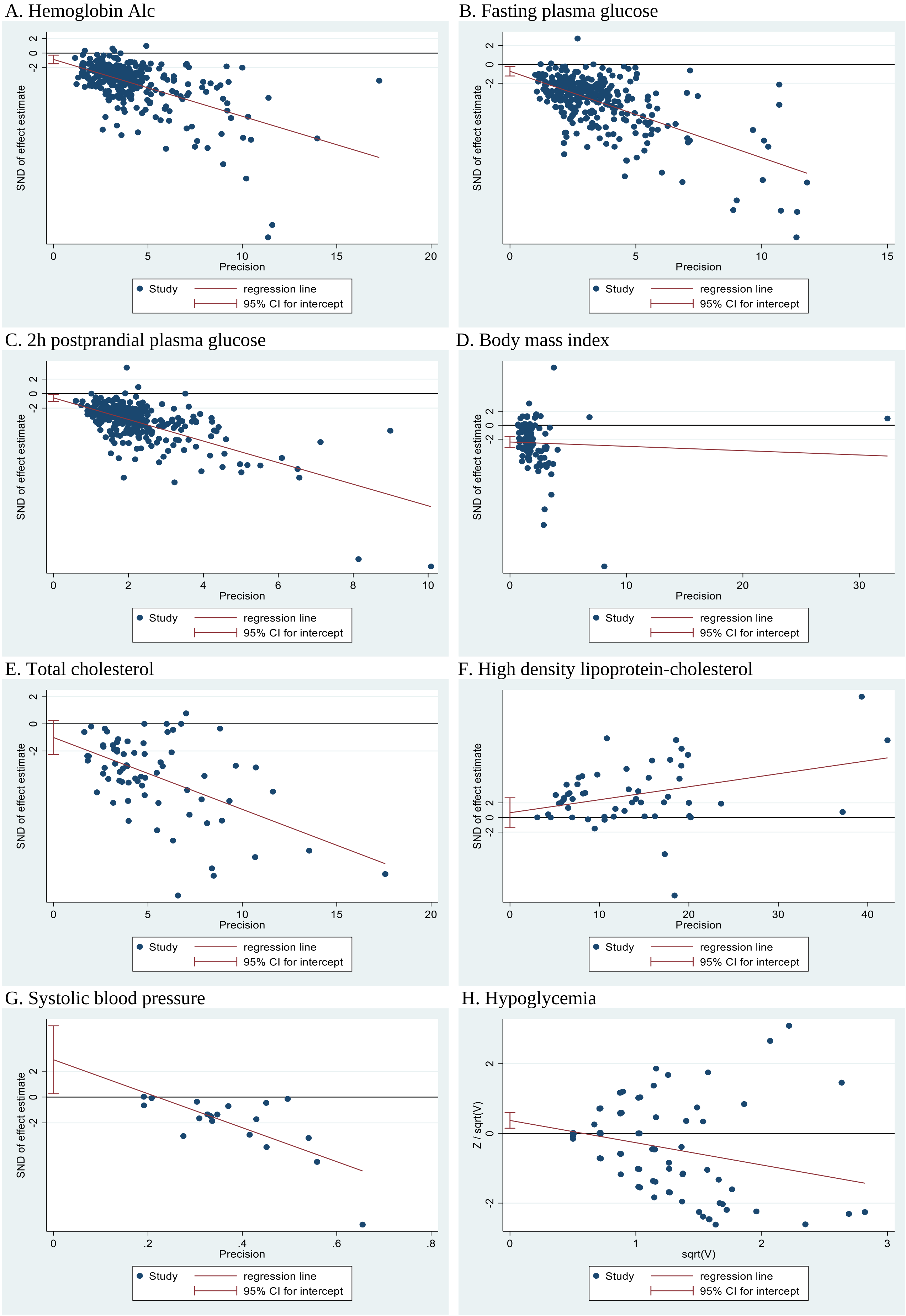

Figure S1. Risk of publication bias across studies

【Hemoglobin Alc (%)】

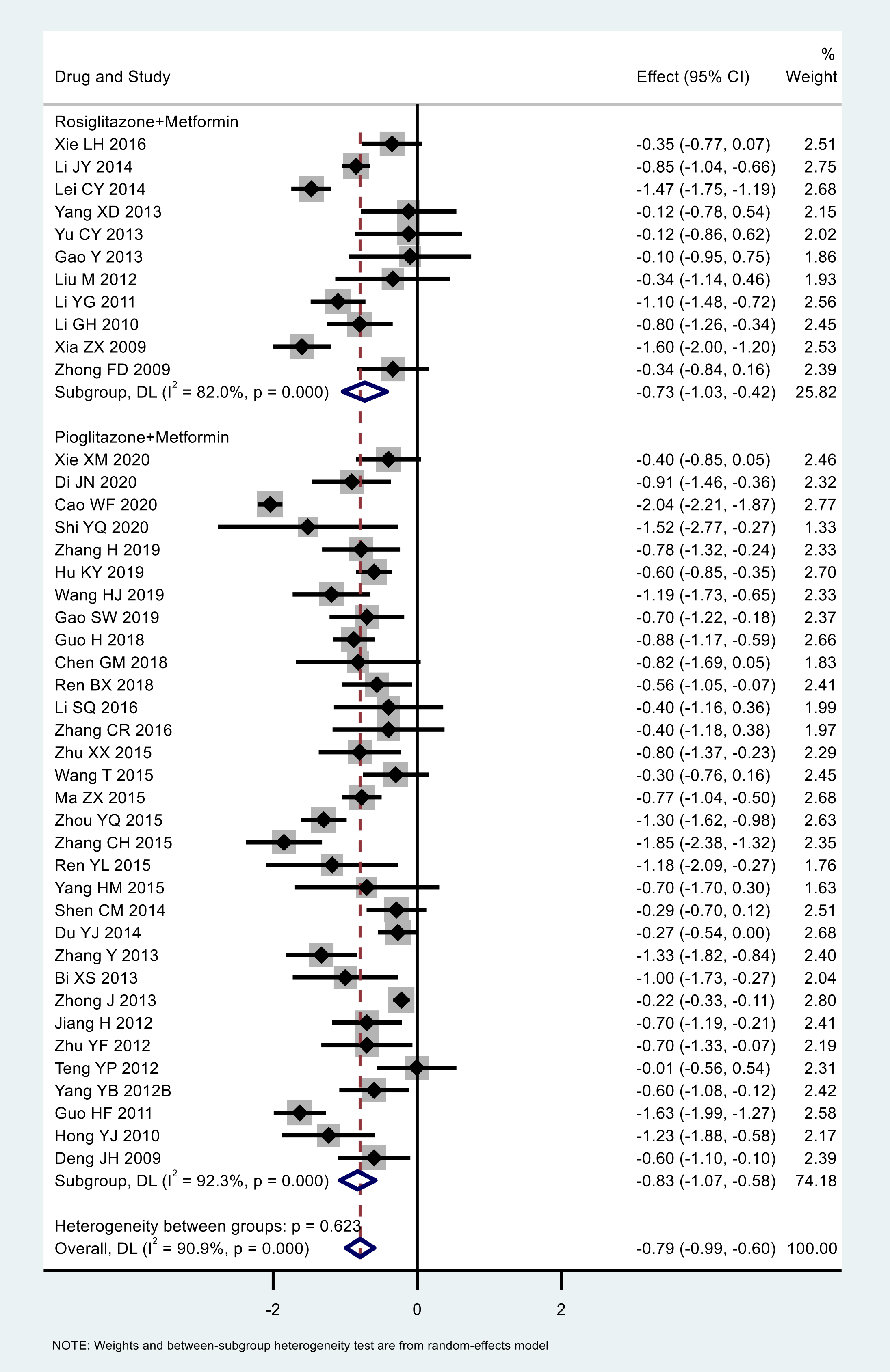

Figure S2. Meta-analysis results for change in hemoglobin Alc (%) of thiazolidinediones added to metformin compared with metformin monotherapy

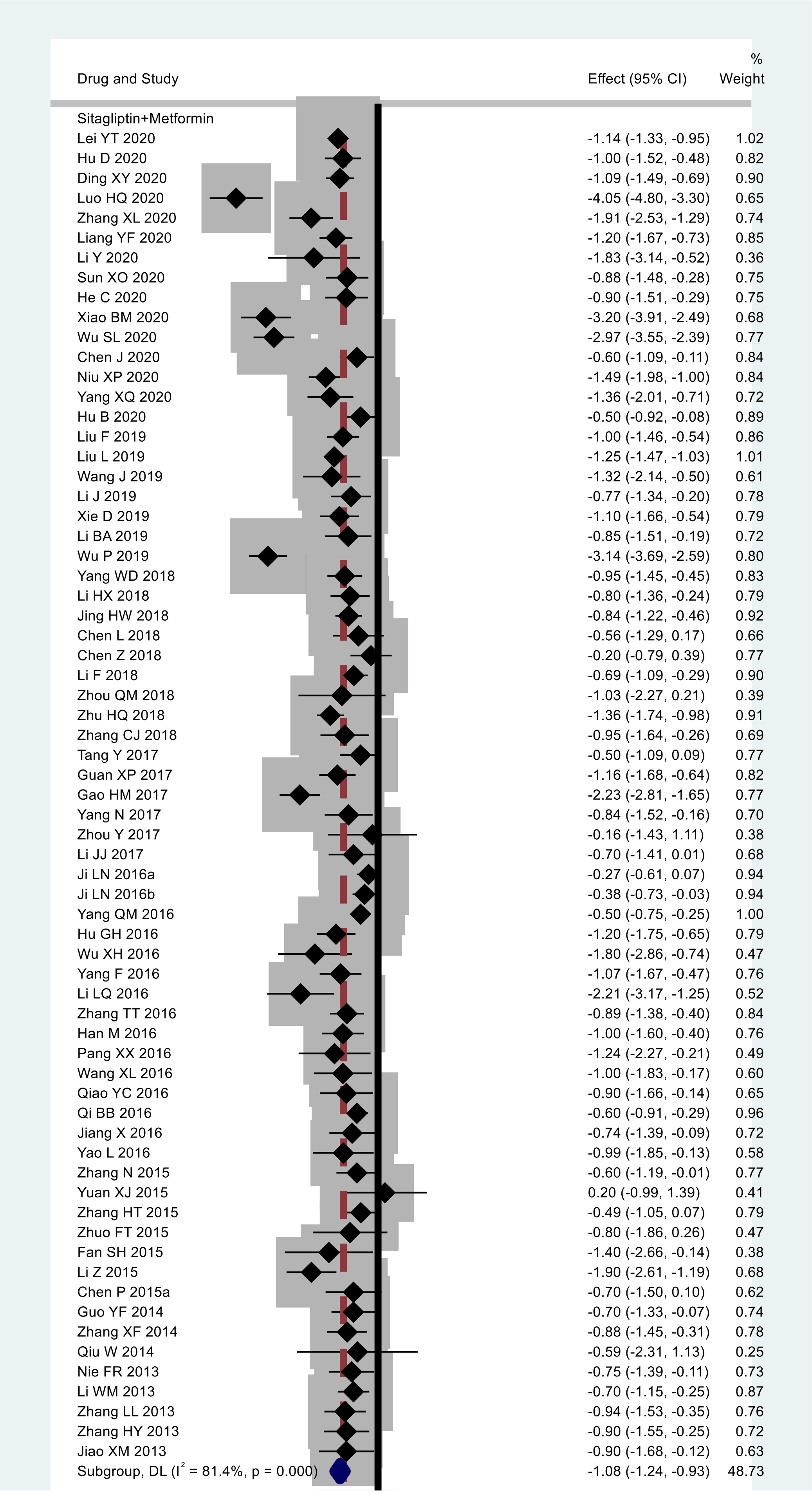
Drug and Study
Effect (95% CI)
% Weight
Sitagliptin+Metformin
Lei YT 2020 -1.14 (-1.33, -0.95) 1.02
Hu D 2020 -1.00 (-1.52, -0.48) 0.82
Ding XY 2020 -1.09 (-1.49, -0.69) 0.90
Luo HQ 2020 -4.05 (-4.80, -3.30) 0.65
Zhang XL 2020 -1.91 (-2.53, -1.29) 0.74
Liang YF 2020 -1.20 (-1.67, -0.73) 0.85
Li Y 2020 -1.83 (-3.14, -0.52) 0.36
Sun XO 2020 -0.88 (-1.48, -0.28) 0.75
He C 2020 -0.90 (-1.51, -0.29) 0.75
Xiao BM 2020 -3.20 (-3.91, -2.49) 0.68
Wu SL 2020 -2.97 (-3.55, -2.39) 0.77
Chen J 2020 -0.60 (-1.09, -0.11) 0.84
Niu XP 2020 -1.49 (-1.98, -1.00) 0.84
Yang XQ 2020 -1.36 (-2.01, -0.71) 0.72
Hu B 2020 -0.50 (-0.92, -0.08) 0.89
Liu F 2019 -1.00 (-1.46, -0.54) 0.86
Liu L 2019 -1.25 (-1.47, -1.03) 1.01
Wang J 2019 -1.32 (-2.14, -0.50) 0.61
Li J 2019 -0.77 (-1.34, -0.20) 0.78
Xie D 2019 -1.10 (-1.66, -0.54) 0.79
Li BA 2019 -0.85 (-1.51, -0.19) 0.72
Wu P 2019 -3.14 (-3.69, -2.59) 0.80
Yang WD 2018 -0.95 (-1.45, -0.45) 0.83
Li HX 2018 -0.80 (-1.36, -0.24) 0.79
Jing HW 2018 -0.84 (-1.22, -0.46) 0.92
Chen L 2018 -0.56 (-1.29, 0.17) 0.66
Chen Z 2018 -0.20 (-0.79, 0.39) 0.77
Li F 2018 -0.69 (-1.09, -0.29) 0.90
Zhou QM 2018 -1.03 (-2.27, 0.21) 0.39
Zhu HQ 2018 -1.36 (-1.74, -0.98) 0.91
Zhang CJ 2018 -0.95 (-1.64, -0.26) 0.69
Tang Y 2017 -0.50 (-1.09, 0.09) 0.77
Guan XP 2017 -1.16 (-1.68, -0.64) 0.82
Gao HM 2017 -2.23 (-2.81, -1.65) 0.77
Yang N 2017 -0.84 (-1.52, -0.16) 0.70
Zhou Y 2017 -0.16 (-1.43, 1.11) 0.38
Li JJ 2017 -0.70 (-1.41, 0.01) 0.68
Ji LN 2016a -0.27 (-0.61, 0.07) 0.94
Ji LN 2016b -0.38 (-0.73, -0.03) 0.94
Yang QM 2016 -0.50 (-0.75, -0.25) 1.00
Hu GH 2016 -1.20 (-1.75, -0.65) 0.79
Wu XH 2016 -1.80 (-2.86, -0.74) 0.47
Yang F 2016 -1.07 (-1.67, -0.47) 0.76
Li LQ 2016 -2.21 (-3.17, -1.25) 0.52
Zhang TT 2016 -0.89 (-1.38, -0.40) 0.84
Han M 2016 -1.00 (-1.60, -0.40) 0.76
Pang XX 2016 -1.24 (-2.27, -0.21) 0.49
Wang XL 2016 -1.00 (-1.83, -0.17) 0.60
Qiao YC 2016 -0.90 (-1.66, -0.14) 0.65
Qi BB 2016 -0.60 (-0.91, -0.29) 0.96
Jiang X 2016 -0.74 (-1.39, -0.09) 0.72
Yao L 2016 -0.99 (-1.85, -0.13) 0.58
Zhang N 2015 -0.60 (-1.19, -0.01) 0.77
Yuan XJ 2015 0.20 (-0.99, 1.39) 0.41
Zhang HT 2015 -0.49 (-1.05, 0.07) 0.79
Zhuo FT 2015 -0.80 (-1.86, 0.26) 0.47
Fan SH 2015 -1.40 (-2.66, -0.14) 0.38
Li Z 2015 -1.90 (-2.61, -1.19) 0.68
Chen P 2015a -0.70 (-1.50, 0.10) 0.62
Guo YF 2014 -0.70 (-1.33, -0.07) 0.74
Zhang XF 2014 -0.88 (-1.45, -0.31) 0.78
Qiu W 2014 -0.59 (-2.31, 1.13) 0.25
Nie FR 2013 -0.75 (-1.39, -0.11) 0.73
Li WM 2013 -0.70 (-1.15, -0.25) 0.87
Zhang LL 2013 -0.94 (-1.53, -0.35) 0.76
Zhang HY 2013 -0.90 (-1.55, -0.25) 0.72
Jiao XM 2013 -0.90 (-1.68, -0.12) 0.63
Subgroup, DL (I² = 81.4%, p = 0.000) -1.08 (-1.24, -0.93) 48.73

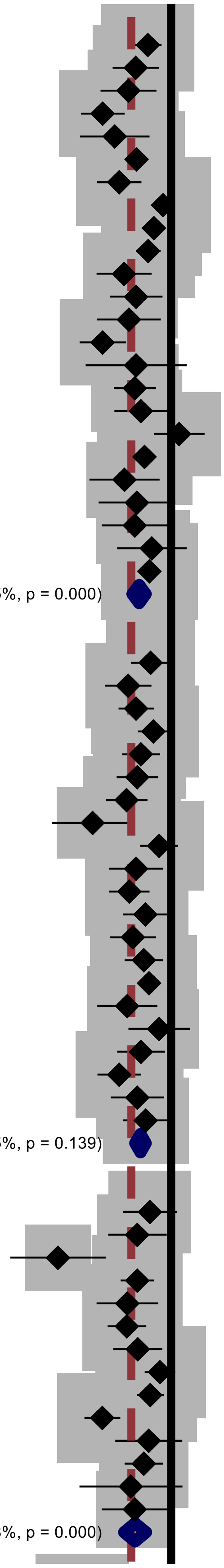

Study	ES (95% CI)	Weight
**Saxagliptin+Metformin**		
Li J 2020	-0.58 (-0.92, -0.24)	0.94
Deng YY 2020	-0.88 (-1.45, -0.31)	0.78
Zheng HY 2020	-1.06 (-1.75, -0.37)	0.69
Zhao PY 2019	-1.70 (-2.24, -1.16)	0.80
Mei YJ 2018	-1.40 (-2.26, -0.54)	0.58
Shi FM 2018	-0.86 (-1.07, -0.65)	1.01
Shan L 2018	-1.29 (-1.84, -0.74)	0.79
Tao T 2018	-0.20 (-0.41, 0.01)	1.01
Li GH 2017	-0.43 (-0.64, -0.22)	1.01
Dong H 2017	-0.57 (-0.88, -0.26)	0.96
Zhang W 2017	-1.17 (-1.85, -0.49)	0.70
Li HW 2017	-0.87 (-1.52, -0.22)	0.72
Zhang GP 2017	-1.05 (-1.84, -0.26)	0.62
Ding R 2016	-1.70 (-2.27, -1.13)	0.78
Wang SQ 2016b	-0.87 (-2.12, 0.38)	0.38
Chen QL 2015	-0.90 (-1.42, -0.38)	0.82
Liang M 2015	-0.75 (-1.41, -0.09)	0.71
Feng J 2015	0.20 (-0.43, 0.83)	0.74
Diao YL 2015	-0.66 (-0.90, -0.42)	1.00
Wan J 2015	-1.16 (-2.03, -0.29)	0.58
Sun Y 2014A	-0.85 (-1.80, 0.10)	0.53
Lu JE 2014	-0.90 (-1.72, -0.08)	0.60
Sun Y 2014B	-0.48 (-1.35, 0.39)	0.58
Li JJ 2013	-0.54 (-0.71, -0.37)	1.03
Subgroup, DL (I^2 = 70.5%, p = 0.000)	-0.80 (-0.96, -0.63)	18.37
**Vildagliptin+Metformin**		
Chu YN 2020	-0.51 (-1.00, -0.02)	0.84
Wang TM 2020	-1.07 (-1.65, -0.49)	0.77
Wang QL 2020	-0.87 (-1.31, -0.43)	0.88
Feng Y 2019	-0.44 (-0.83, -0.05)	0.91
Liao QH 2018	-0.75 (-1.22, -0.28)	0.85
Fu CM 2018	-0.84 (-1.35, -0.33)	0.82
Peng XH 2018	-1.11 (-1.63, -0.59)	0.82
Li SJ 2018	-1.95 (-2.96, -0.94)	0.50
Su JF 2018A	-0.30 (-0.77, 0.17)	0.85
Zhao YH 2017	-0.87 (-1.54, -0.20)	0.71
Qu JC 2017A	-1.04 (-1.54, -0.54)	0.83
Shi CL 2017	-0.64 (-1.20, -0.08)	0.79
Li R 2016	-0.95 (-1.53, -0.37)	0.78
Zhao XW 2016A	-0.68 (-1.16, -0.20)	0.85
Jia XW 2015	-0.55 (-0.78, -0.32)	1.01
Zheng ZP 2015	-1.09 (-1.84, -0.34)	0.65
Liang CY 2015	-0.30 (-1.06, 0.46)	0.64
Cheng LY 2014	-0.75 (-1.34, -0.16)	0.76
Chen XS 2014	-1.29 (-1.83, -0.75)	0.80
Yan ZD 2014	-0.84 (-1.50, -0.18)	0.71
Lai KB 2014	-0.63 (-1.20, -0.06)	0.78
Subgroup, DL (I^2 = 25.5%, p = 0.139)	-0.76 (-0.89, -0.63)	16.53
**Linagliptin+Metformin**		
Li RH 2020	-0.53 (-1.20, 0.14)	0.71
Wang XP 2020	-0.84 (-1.57, -0.11)	0.67
Zhou DQ 2020	-2.81 (-3.99, -1.63)	0.41
Sun TT 2019	-0.84 (-1.26, -0.42)	0.89
Chen KF 2019	-1.09 (-1.85, -0.33)	0.64
Guo CM 2019	-1.10 (-1.58, -0.62)	0.85
Bai FW 2019	-0.83 (-1.44, -0.22)	0.75
Niu S 2019	-0.28 (-0.66, 0.10)	0.92
Jin J 2018	-0.52 (-0.85, -0.19)	0.95
Wang XW 2018	-1.71 (-2.16, -1.26)	0.87
Gao FD 2017	-0.56 (-1.39, 0.27)	0.60
Zhao XW 2016B	-0.68 (-1.16, -0.20)	0.85
Zheng FP 2016a	-1.00 (-2.28, 0.28)	0.37
Chen P 2015b	-0.90 (-1.72, -0.08)	0.60
Subgroup, DL (I^2 = 67.8%, p = 0.000)	-0.90 (-1.17, -0.63)	10.07

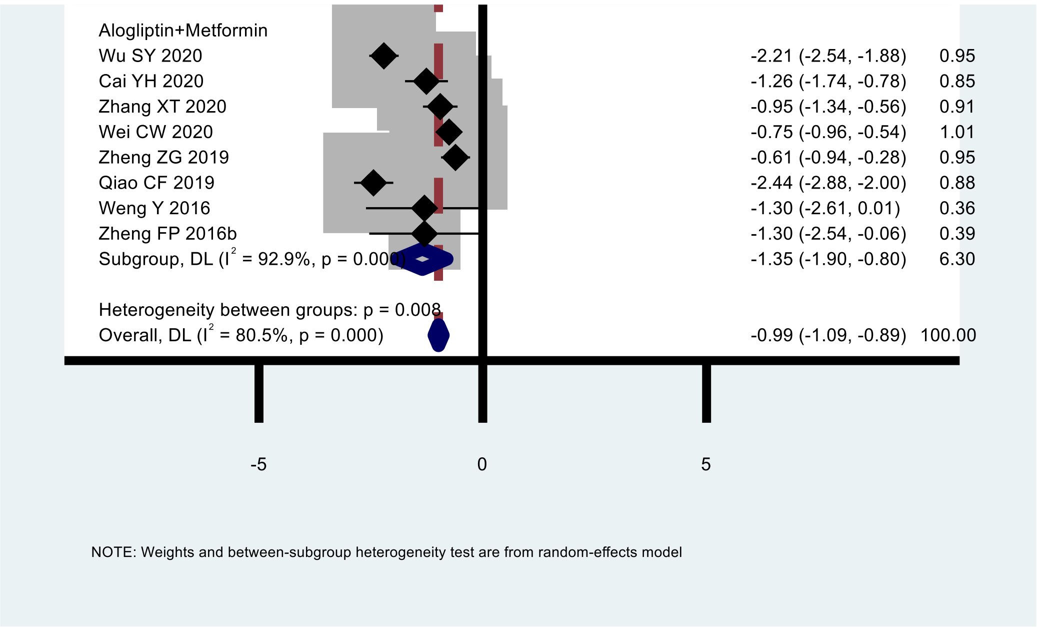

Figure S3. Meta-analysis results for change in hemoglobin Alc (%) of dipeptidyl peptidase 4 inhibitors added to metformin compared with metformin monotherapy

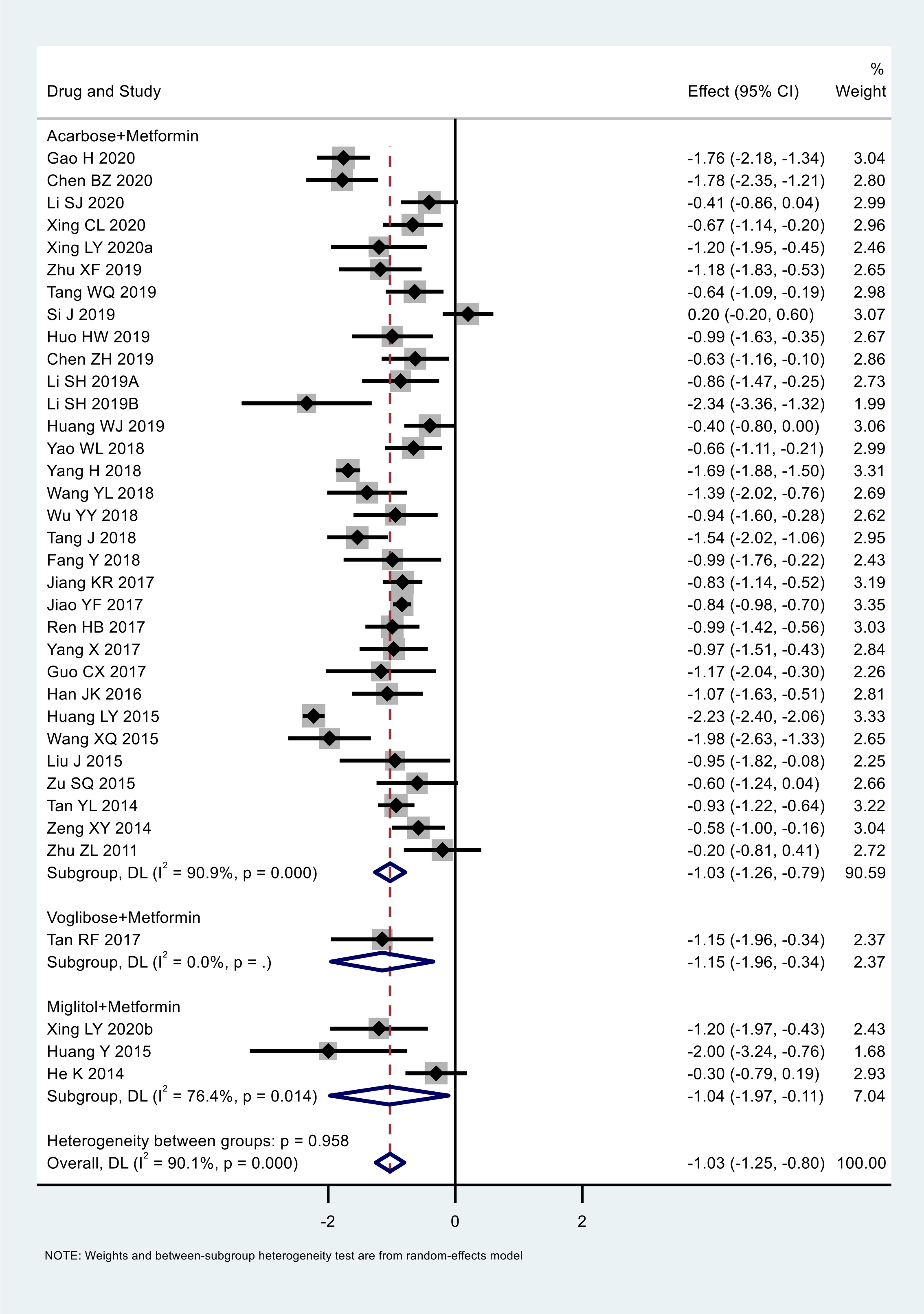

Figure S4. Meta-analysis results for change in hemoglobin Alc (%) of α-glucosidase inhibitors added to metformin compared with metformin monotherapy

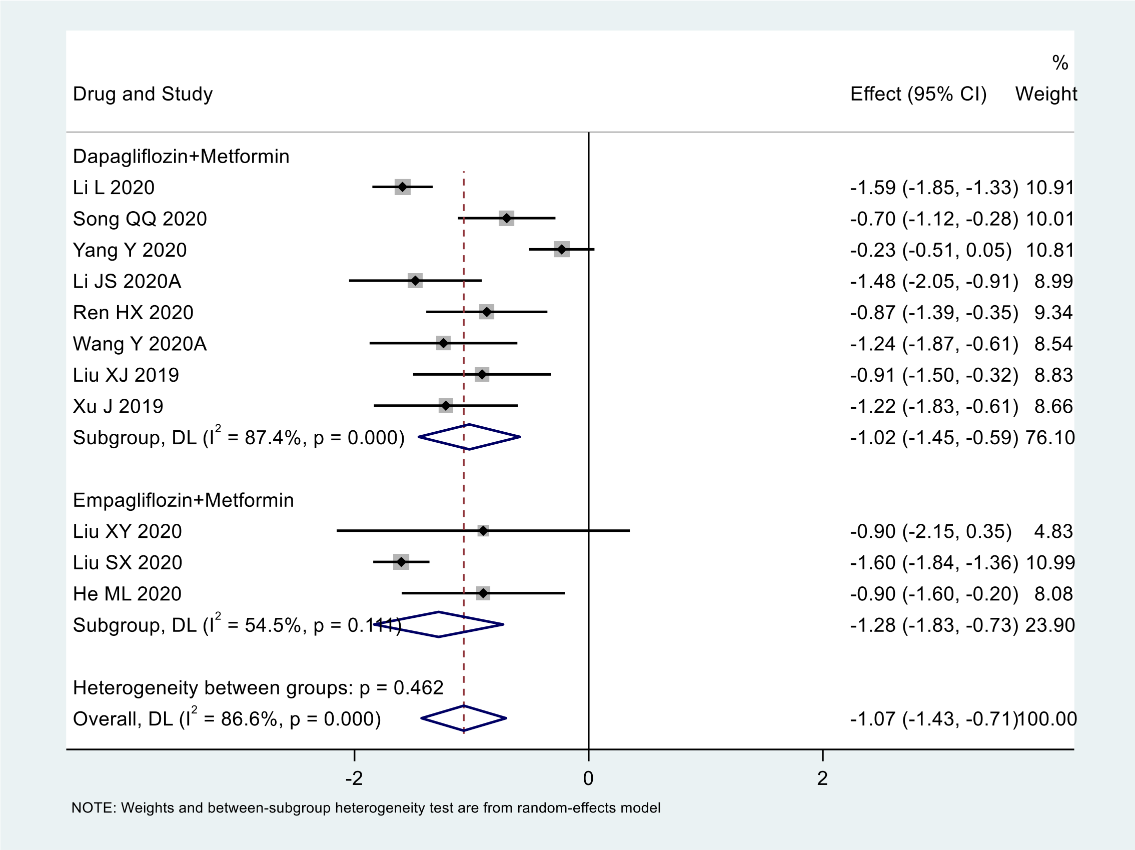

Figure S5. Meta-analysis results for change in hemoglobin Alc (%) of sodium-glucose cotransporter 2 inhibitors added to metformin compared with metformin monotherapy

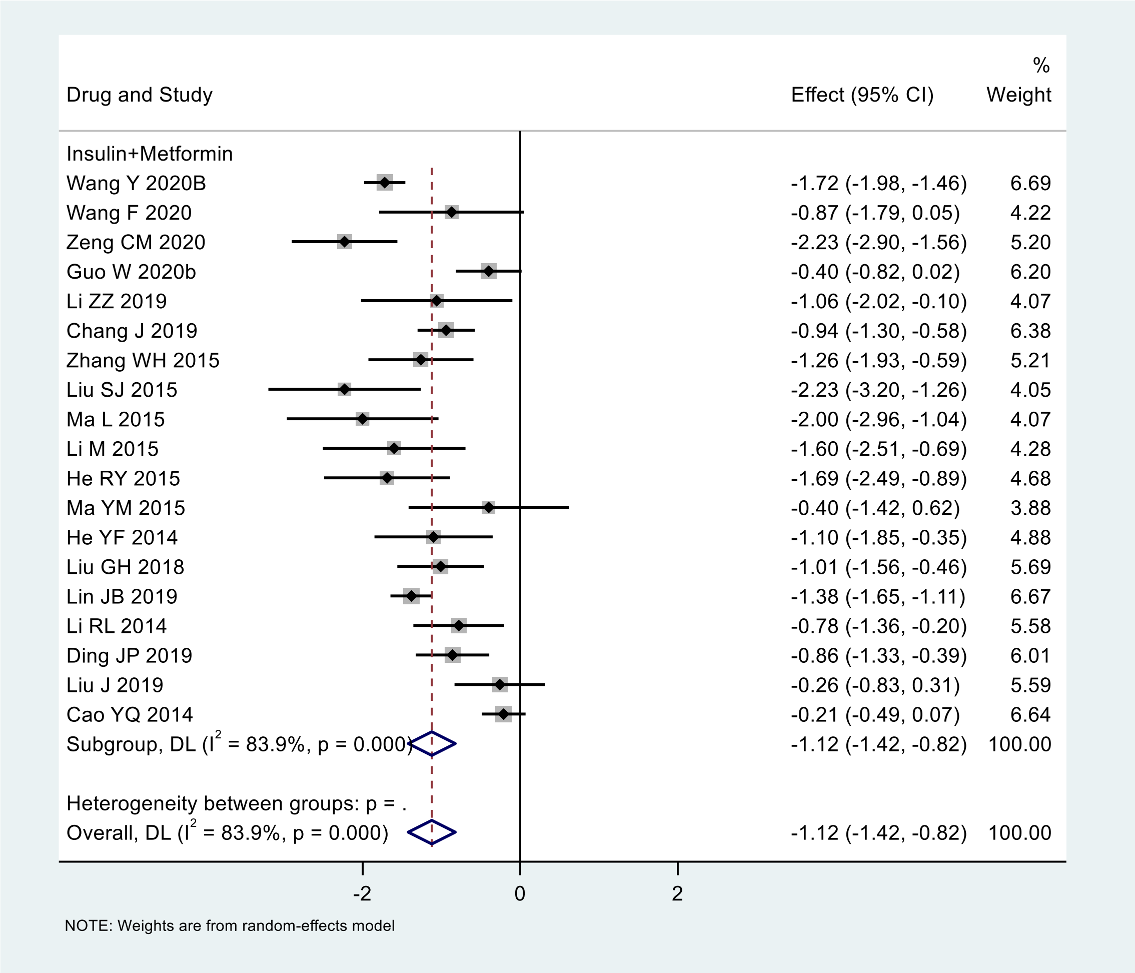

Figure S6. Meta-analysis results for change in hemoglobin Alc (%) of insulins added to metformin compared with metformin monotherapy

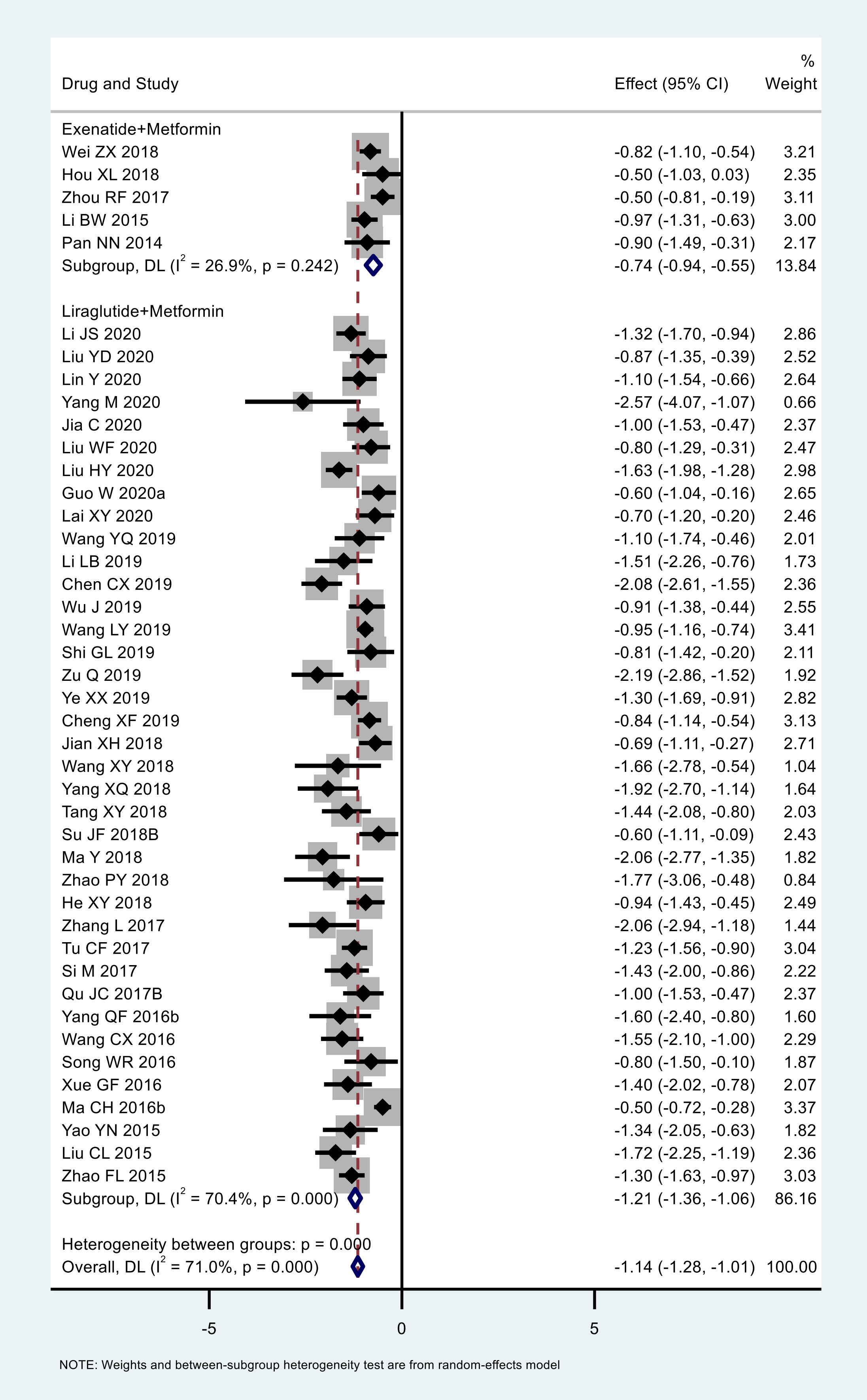

Figure S7. Meta-analysis results for change in hemoglobin Alc (%) of glucagon-like peptide-1 receptor agonists added to metformin compared with metformin monotherapy

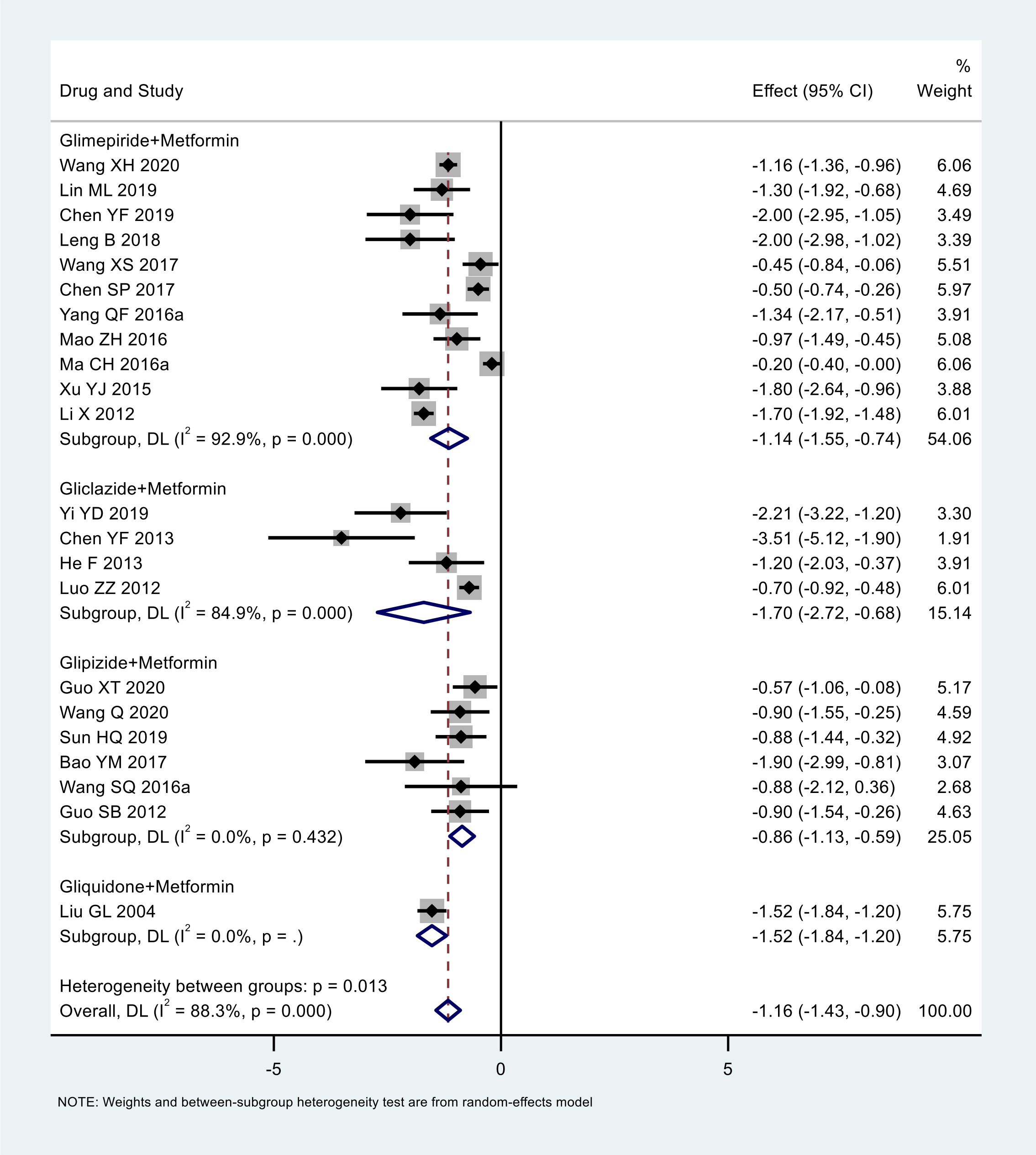

Figure S8. Meta-analysis results for change in hemoglobin Alc (%) of sulfonylureas added to metformin compared with metformin monotherapy

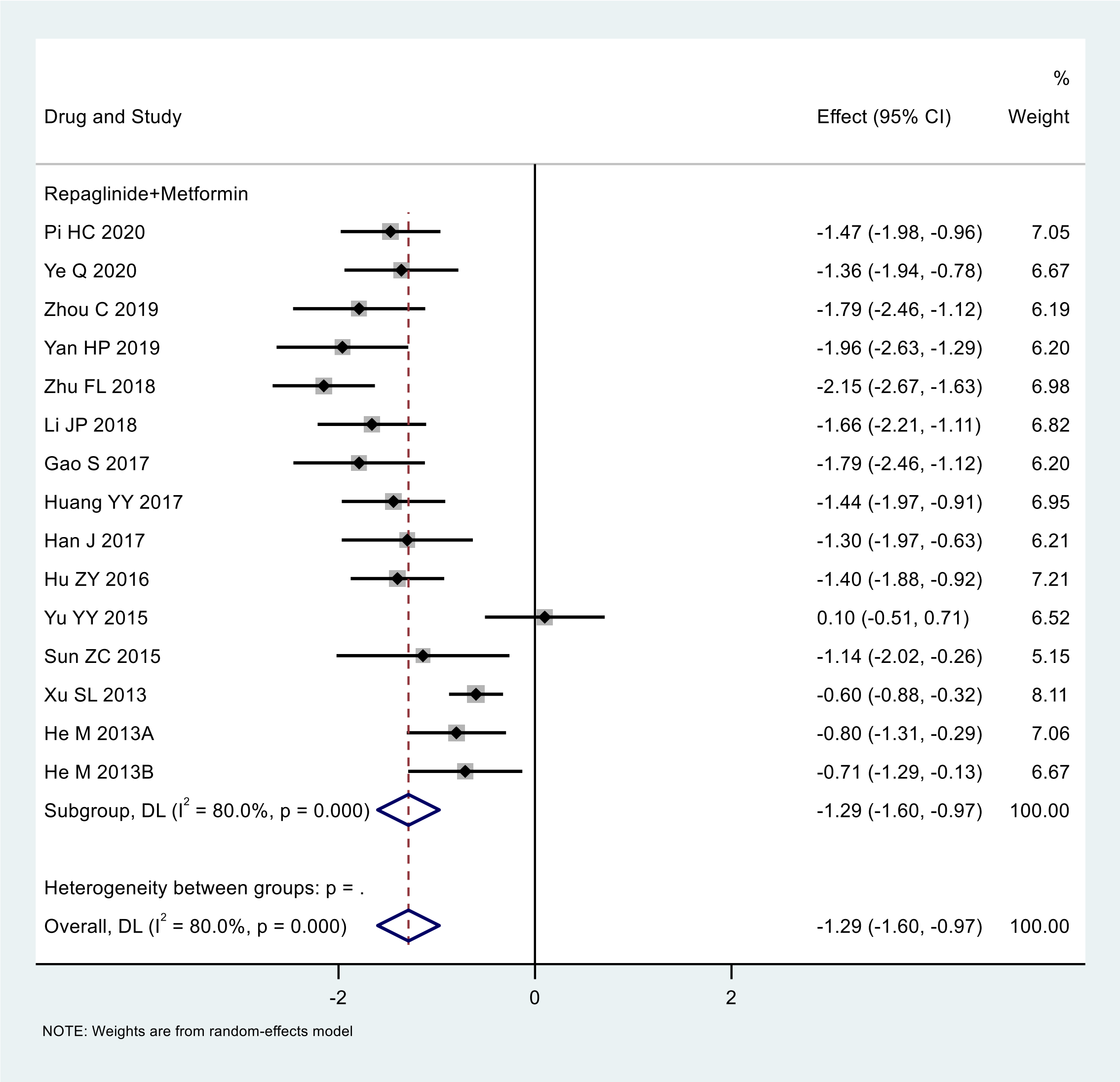

Figure S9. Meta-analysis results for change in hemoglobin Alc (%) of glinides added to metformin compared with metformin monotherapy

【Fasting plasma glucose (mmol/l)】

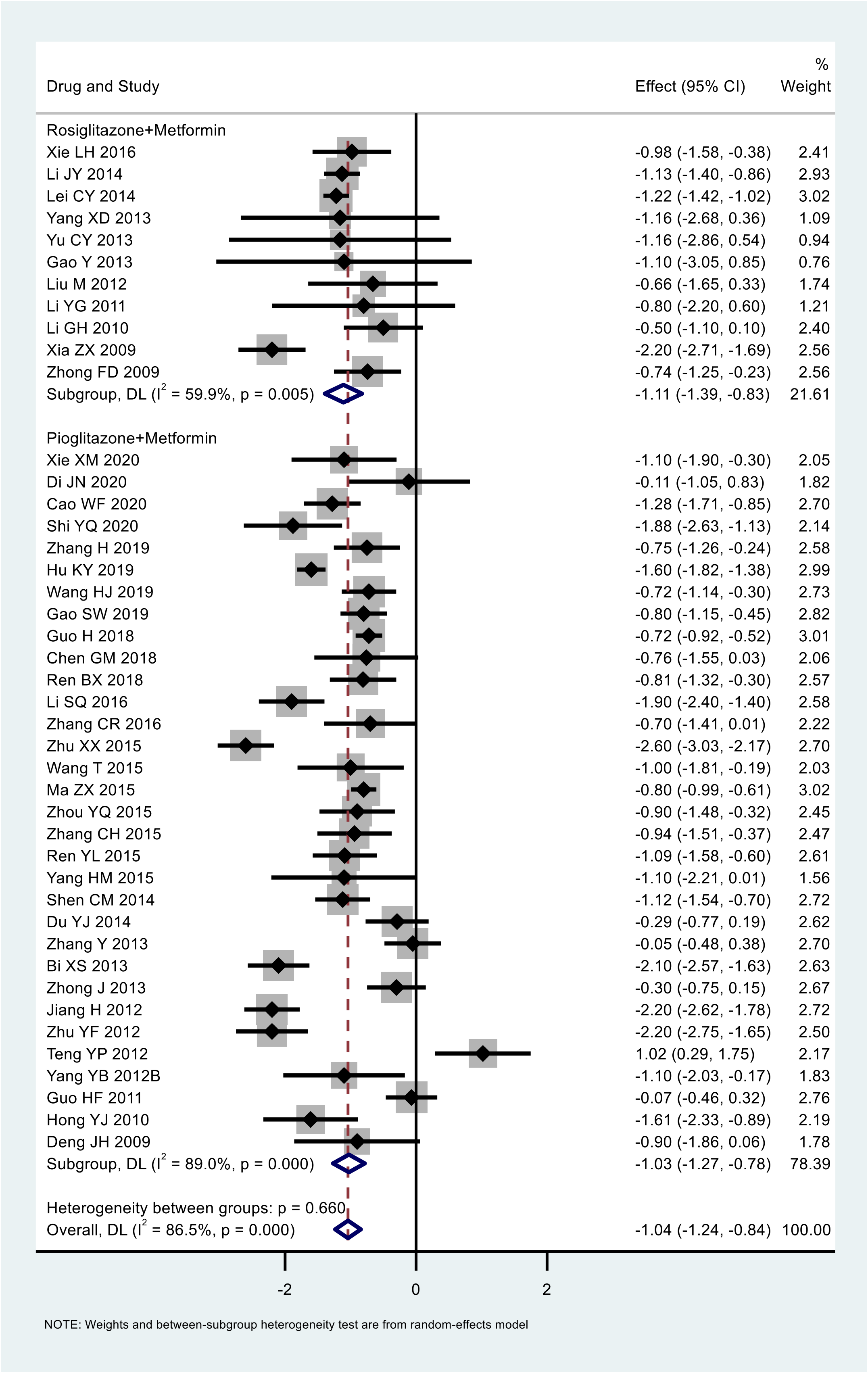

Figure S10. Meta-analysis results for change in fasting plasma glucose (mmol/l) of thiazolidinediones added to metformin compared with metformin monotherapy

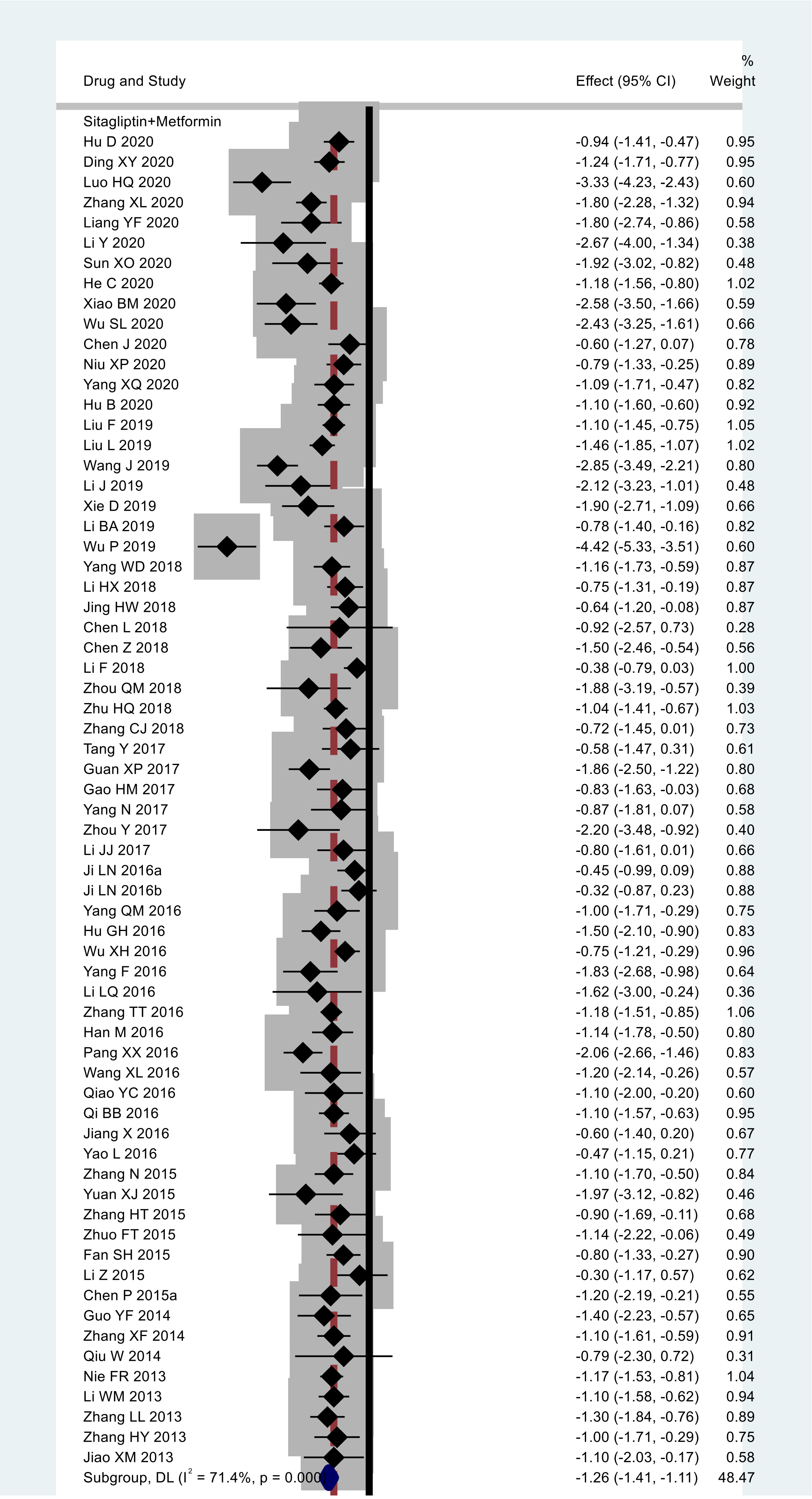
Drug and Study
Effect (95% CI)
% Weight
Sitagliptin+Metformin
Hu D 2020 -0.94 (-1.41, -0.47) 0.95
Ding XY 2020 -1.24 (-1.71, -0.77) 0.95
Luo HQ 2020 -3.33 (-4.23, -2.43) 0.60
Zhang XL 2020 -1.80 (-2.28, -1.32) 0.94
Liang YF 2020 -1.80 (-2.74, -0.86) 0.58
Li Y 2020 -2.67 (-4.00, -1.34) 0.38
Sun XO 2020 -1.92 (-3.02, -0.82) 0.48
He C 2020 -1.18 (-1.56, -0.80) 1.02
Xiao BM 2020 -2.58 (-3.50, -1.66) 0.59
Wu SL 2020 -2.43 (-3.25, -1.61) 0.66
Chen J 2020 -0.60 (-1.27, 0.07) 0.78
Niu XP 2020 -0.79 (-1.33, -0.25) 0.89
Yang XQ 2020 -1.09 (-1.71, -0.47) 0.82
Hu B 2020 -1.10 (-1.60, -0.60) 0.92
Liu F 2019 -1.10 (-1.45, -0.75) 1.05
Liu L 2019 -1.46 (-1.85, -1.07) 1.02
Wang J 2019 -2.85 (-3.49, -2.21) 0.80
Li J 2019 -2.12 (-3.23, -1.01) 0.48
Xie D 2019 -1.90 (-2.71, -1.09) 0.66
Li BA 2019 -0.78 (-1.40, -0.16) 0.82
Wu P 2019 -4.42 (-5.33, -3.51) 0.60
Yang WD 2018 -1.16 (-1.73, -0.59) 0.87
Li HX 2018 -0.75 (-1.31, -0.19) 0.87
Jing HW 2018 -0.64 (-1.20, -0.08) 0.87
Chen L 2018 -0.92 (-2.57, 0.73) 0.28
Chen Z 2018 -1.50 (-2.46, -0.54) 0.56
Li F 2018 -0.38 (-0.79, 0.03) 1.00
Zhou QM 2018 -1.88 (-3.19, -0.57) 0.39
Zhu HQ 2018 -1.04 (-1.41, -0.67) 1.03
Zhang CJ 2018 -0.72 (-1.45, 0.01) 0.73
Tang Y 2017 -0.58 (-1.47, 0.31) 0.61
Guan XP 2017 -1.86 (-2.50, -1.22) 0.80
Gao HM 2017 -0.83 (-1.63, -0.03) 0.68
Yang N 2017 -0.87 (-1.81, 0.07) 0.58
Zhou Y 2017 -2.20 (-3.48, -0.92) 0.40
Li JJ 2017 -0.80 (-1.61, 0.01) 0.66
Ji LN 2016a -0.45 (-0.99, 0.09) 0.88
Ji LN 2016b -0.32 (-0.87, 0.23) 0.88
Yang QM 2016 -1.00 (-1.71, -0.29) 0.75
Hu GH 2016 -1.50 (-2.10, -0.90) 0.83
Wu XH 2016 -0.75 (-1.21, -0.29) 0.96
Yang F 2016 -1.83 (-2.68, -0.98) 0.64
Li LQ 2016 -1.62 (-3.00, -0.24) 0.36
Zhang TT 2016 -1.18 (-1.51, -0.85) 1.06
Han M 2016 -1.14 (-1.78, -0.50) 0.80
Pang XX 2016 -2.06 (-2.66, -1.46) 0.83
Wang XL 2016 -1.20 (-2.14, -0.26) 0.57
Qiao YC 2016 -1.10 (-2.00, -0.20) 0.60
Qi BB 2016 -1.10 (-1.57, -0.63) 0.95
Jiang X 2016 -0.60 (-1.40, 0.20) 0.67
Yao L 2016 -0.47 (-1.15, 0.21) 0.77
Zhang N 2015 -1.10 (-1.70, -0.50) 0.84
Yuan XJ 2015 -1.97 (-3.12, -0.82) 0.46
Zhang HT 2015 -0.90 (-1.69, -0.11) 0.68
Zhuo FT 2015 -1.14 (-2.22, -0.06) 0.49
Fan SH 2015 -0.80 (-1.33, -0.27) 0.90
Li Z 2015 -0.30 (-1.17, 0.57) 0.62
Chen P 2015a -1.20 (-2.19, -0.21) 0.55
Guo YF 2014 -1.40 (-2.23, -0.57) 0.65
Zhang XF 2014 -1.10 (-1.61, -0.59) 0.91
Qiu W 2014 -0.79 (-2.30, 0.72) 0.31
Nie FR 2013 -1.17 (-1.53, -0.81) 1.04
Li WM 2013 -1.10 (-1.58, -0.62) 0.94
Zhang LL 2013 -1.30 (-1.84, -0.76) 0.89
Zhang HY 2013 -1.00 (-1.71, -0.29) 0.75
Jiao XM 2013 -1.10 (-2.03, -0.17) 0.58
Subgroup, DL (I² = 71.4%, p = 0.000) -1.26 (-1.41, -1.11) 48.47

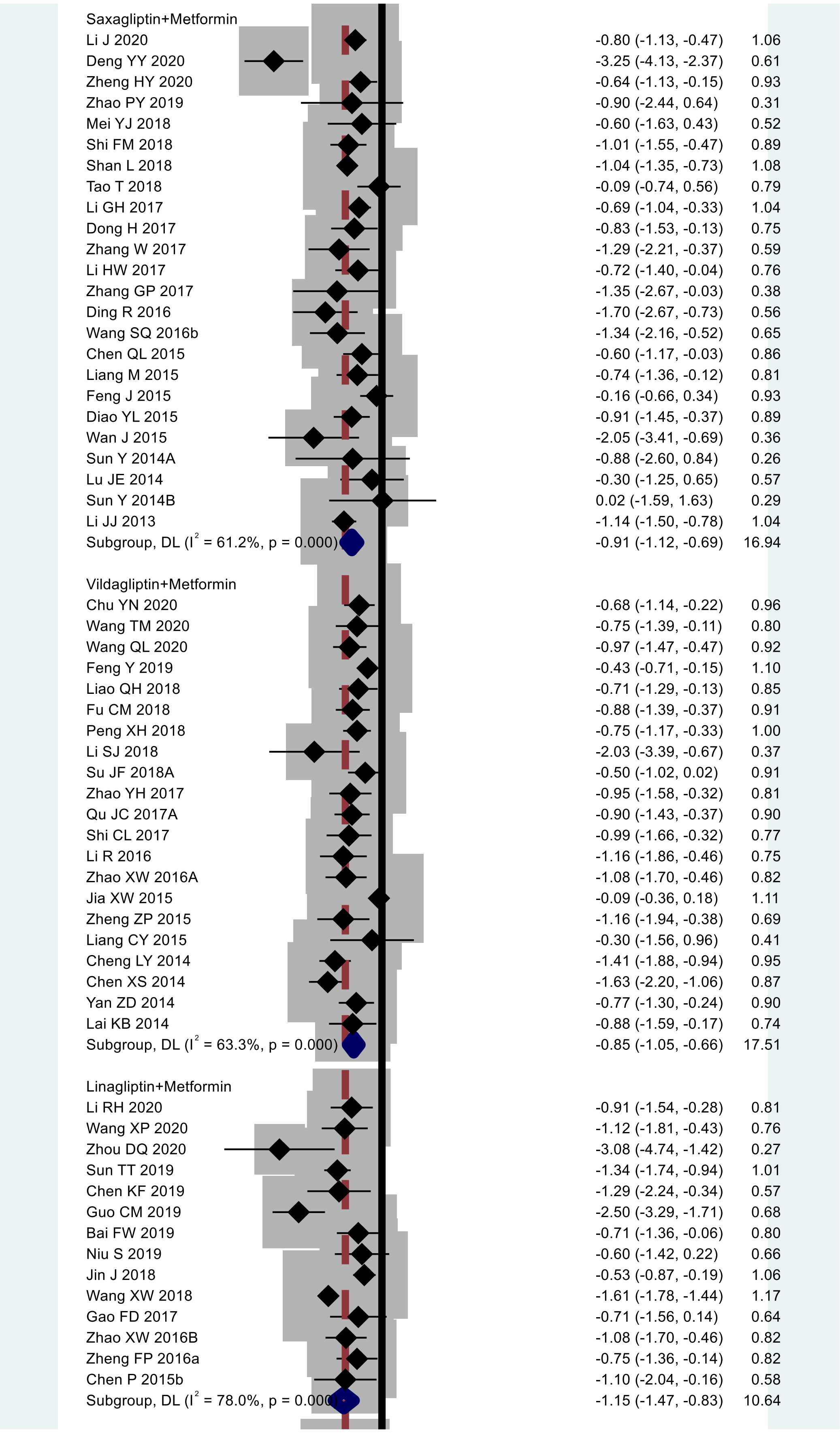
Saxagliptin+Metformin
Li J 2020 -0.80 (-1.13, -0.47) 1.06
Deng YY 2020 -3.25 (-4.13, -2.37) 0.61
Zheng HY 2020 -0.64 (-1.13, -0.15) 0.93
Zhao PY 2019 -0.90 (-2.44, 0.64) 0.31
Mei YJ 2018 -0.60 (-1.63, 0.43) 0.52
Shi FM 2018 -1.01 (-1.55, -0.47) 0.89
Shan L 2018 -1.04 (-1.35, -0.73) 1.08
Tao T 2018 -0.09 (-0.74, 0.56) 0.79
Li GH 2017 -0.69 (-1.04, -0.33) 1.04
Dong H 2017 -0.83 (-1.53, -0.13) 0.75
Zhang W 2017 -1.29 (-2.21, -0.37) 0.59
Li HW 2017 -0.72 (-1.40, -0.04) 0.76
Zhang GP 2017 -1.35 (-2.67, -0.03) 0.38
Ding R 2016 -1.70 (-2.67, -0.73) 0.56
Wang SQ 2016b -1.34 (-2.16, -0.52) 0.65
Chen QL 2015 -0.60 (-1.17, -0.03) 0.86
Liang M 2015 -0.74 (-1.36, -0.12) 0.81
Feng J 2015 -0.16 (-0.66, 0.34) 0.93
Diao YL 2015 -0.91 (-1.45, -0.37) 0.89
Wan J 2015 -2.05 (-3.41, -0.69) 0.36
Sun Y 2014A -0.88 (-2.60, 0.84) 0.26
Lu JE 2014 -0.30 (-1.25, 0.65) 0.57
Sun Y 2014B 0.02 (-1.59, 1.63) 0.29
Li JJ 2013 -1.14 (-1.50, -0.78) 1.04
Subgroup, DL (I² = 61.2%, p = 0.000) -0.91 (-1.12, -0.69) 16.94
Vildagliptin+Metformin
Chu YN 2020 -0.68 (-1.14, -0.22) 0.96
Wang TM 2020 -0.75 (-1.39, -0.11) 0.80
Wang QL 2020 -0.97 (-1.47, -0.47) 0.92
Feng Y 2019 -0.43 (-0.71, -0.15) 1.10
Liao QH 2018 -0.71 (-1.29, -0.13) 0.85
Fu CM 2018 -0.88 (-1.39, -0.37) 0.91
Peng XH 2018 -0.75 (-1.17, -0.33) 1.00
Li SJ 2018 -2.03 (-3.39, -0.67) 0.37
Su JF 2018A -0.50 (-1.02, 0.02) 0.91
Zhao YH 2017 -0.95 (-1.58, -0.32) 0.81
Qu JC 2017A -0.90 (-1.43, -0.37) 0.90
Shi CL 2017 -0.99 (-1.66, -0.32) 0.77
Li R 2016 -1.16 (-1.86, -0.46) 0.75
Zhao XW 2016A -1.08 (-1.70, -0.46) 0.82
Jia XW 2015 -0.09 (-0.36, 0.18) 1.11
Zheng ZP 2015 -1.16 (-1.94, -0.38) 0.69
Liang CY 2015 -0.30 (-1.56, 0.96) 0.41
Cheng LY 2014 -1.41 (-1.88, -0.94) 0.95
Chen XS 2014 -1.63 (-2.20, -1.06) 0.87
Yan ZD 2014 -0.77 (-1.30, -0.24) 0.90
Lai KB 2014 -0.88 (-1.59, -0.17) 0.74
Subgroup, DL (I² = 63.3%, p = 0.000) -0.85 (-1.05, -0.66) 17.51
Linagliptin+Metformin
Li RH 2020 -0.91 (-1.54, -0.28) 0.81
Wang XP 2020 -1.12 (-1.81, -0.43) 0.76
Zhou DQ 2020 -3.08 (-4.74, -1.42) 0.27
Sun TT 2019 -1.34 (-1.74, -0.94) 1.01
Chen KF 2019 -1.29 (-2.24, -0.34) 0.57
Guo CM 2019 -2.50 (-3.29, -1.71) 0.68
Bai FW 2019 -0.71 (-1.36, -0.06) 0.80
Niu S 2019 -0.60 (-1.42, 0.22) 0.66
Jin J 2018 -0.53 (-0.87, -0.19) 1.06
Wang XW 2018 -1.61 (-1.78, -1.44) 1.17
Gao FD 2017 -0.71 (-1.56, 0.14) 0.64
Zhao XW 2016B -1.08 (-1.70, -0.46) 0.82
Zheng FP 2016a -0.75 (-1.36, -0.14) 0.82
Chen P 2015b -1.10 (-2.04, -0.16) 0.58
Subgroup, DL (I² = 78.0%, p = 0.000) -1.15 (-1.47, -0.83) 10.64

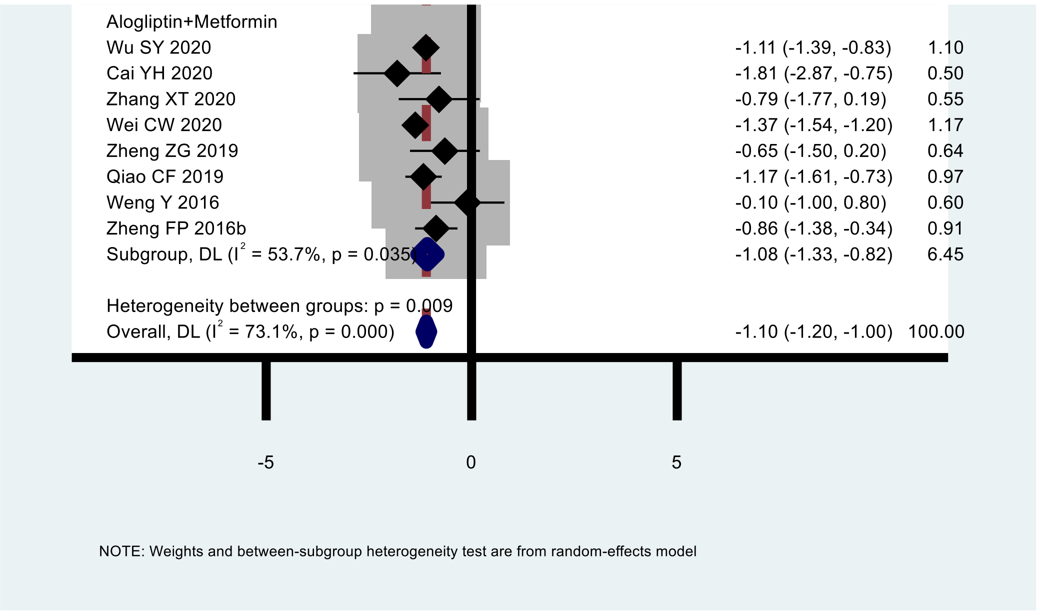

Figure S11. Meta-analysis results for change in fasting plasma glucose (mmol/l) of dipeptidyl peptidase 4 inhibitors added to metformin compared with metformin monotherapy

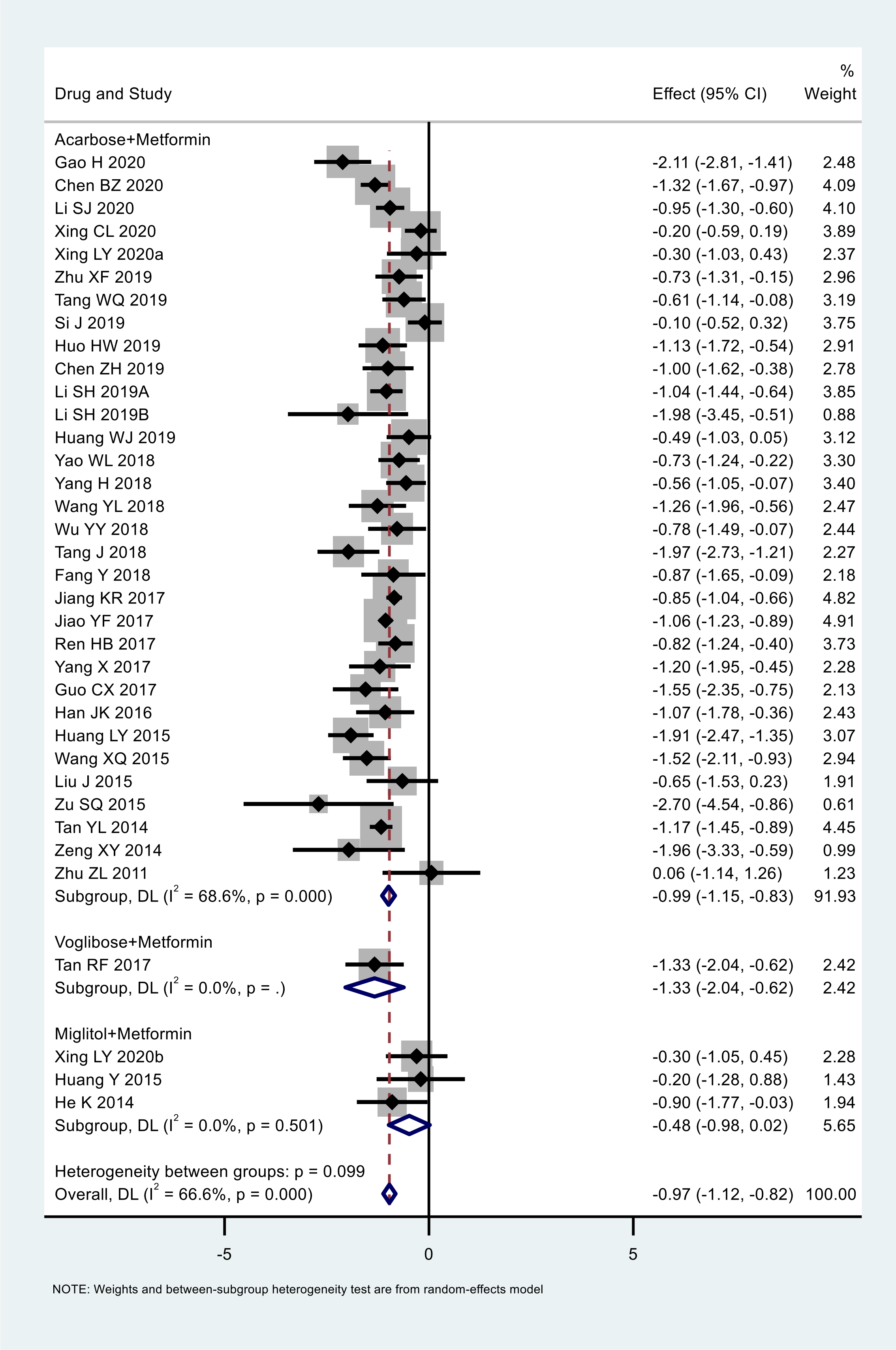

Figure S12. Meta-analysis results for change in fasting plasma glucose (mmol/l) of α-glucosidase inhibitors added to metformin compared with metformin monotherapy

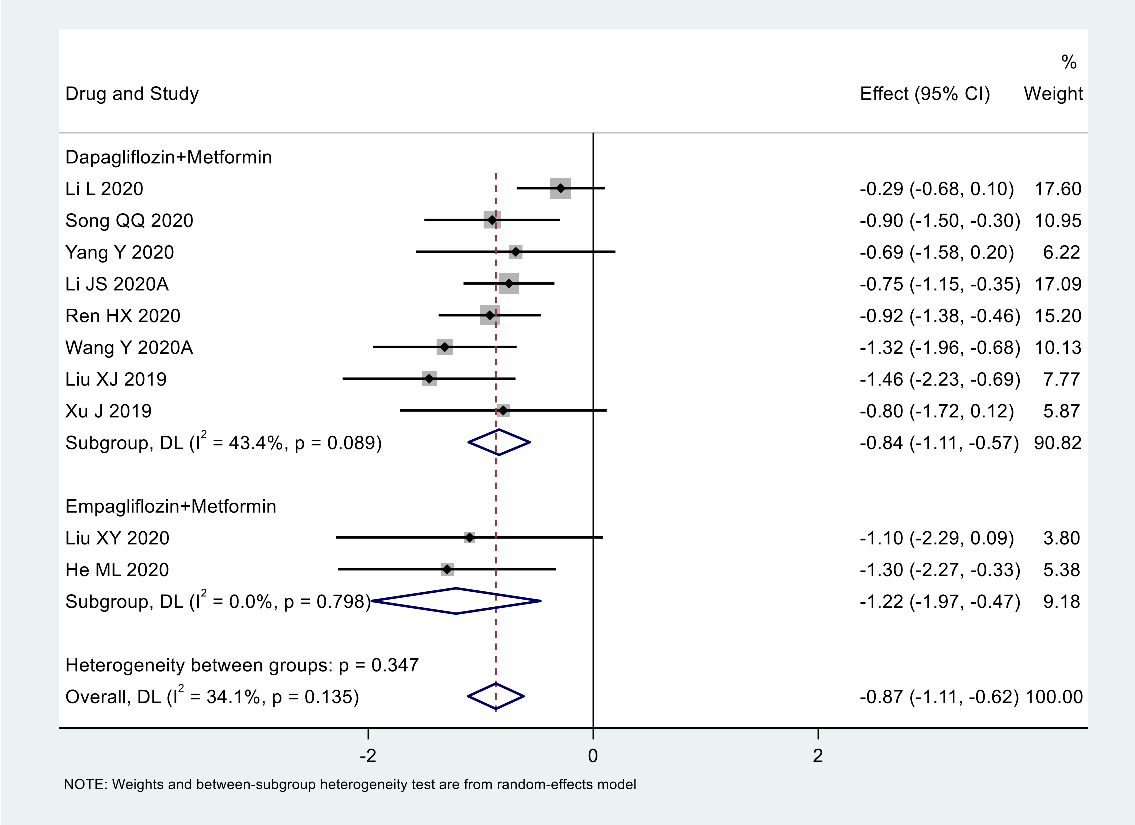

Figure S13. Meta-analysis results for change in fasting plasma glucose (mmol/l) of sodium-glucose cotransporter 2 inhibitors added to metformin compared with metformin monotherapy

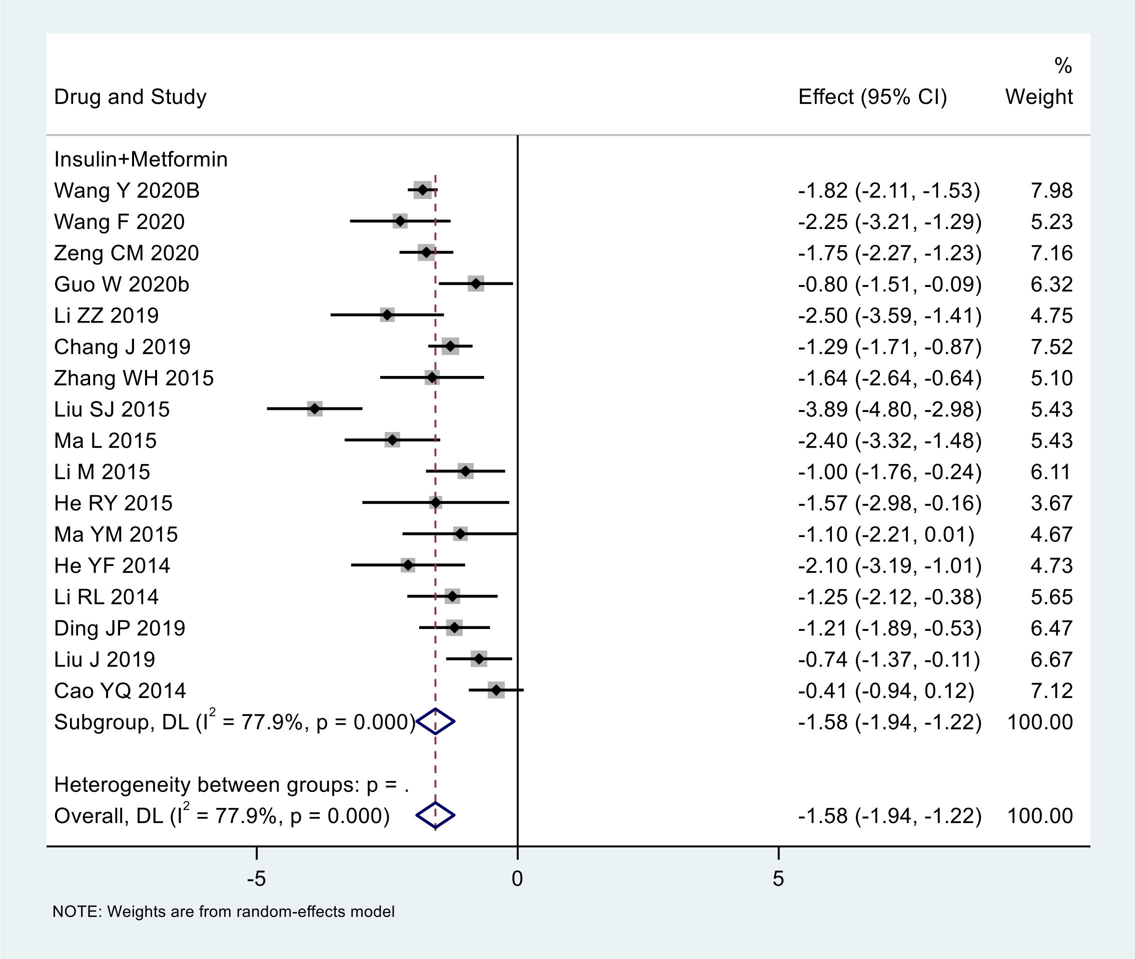

Figure S14. Meta-analysis results for change in fasting plasma glucose (mmol/l) of insulins added to metformin compared with metformin monotherapy

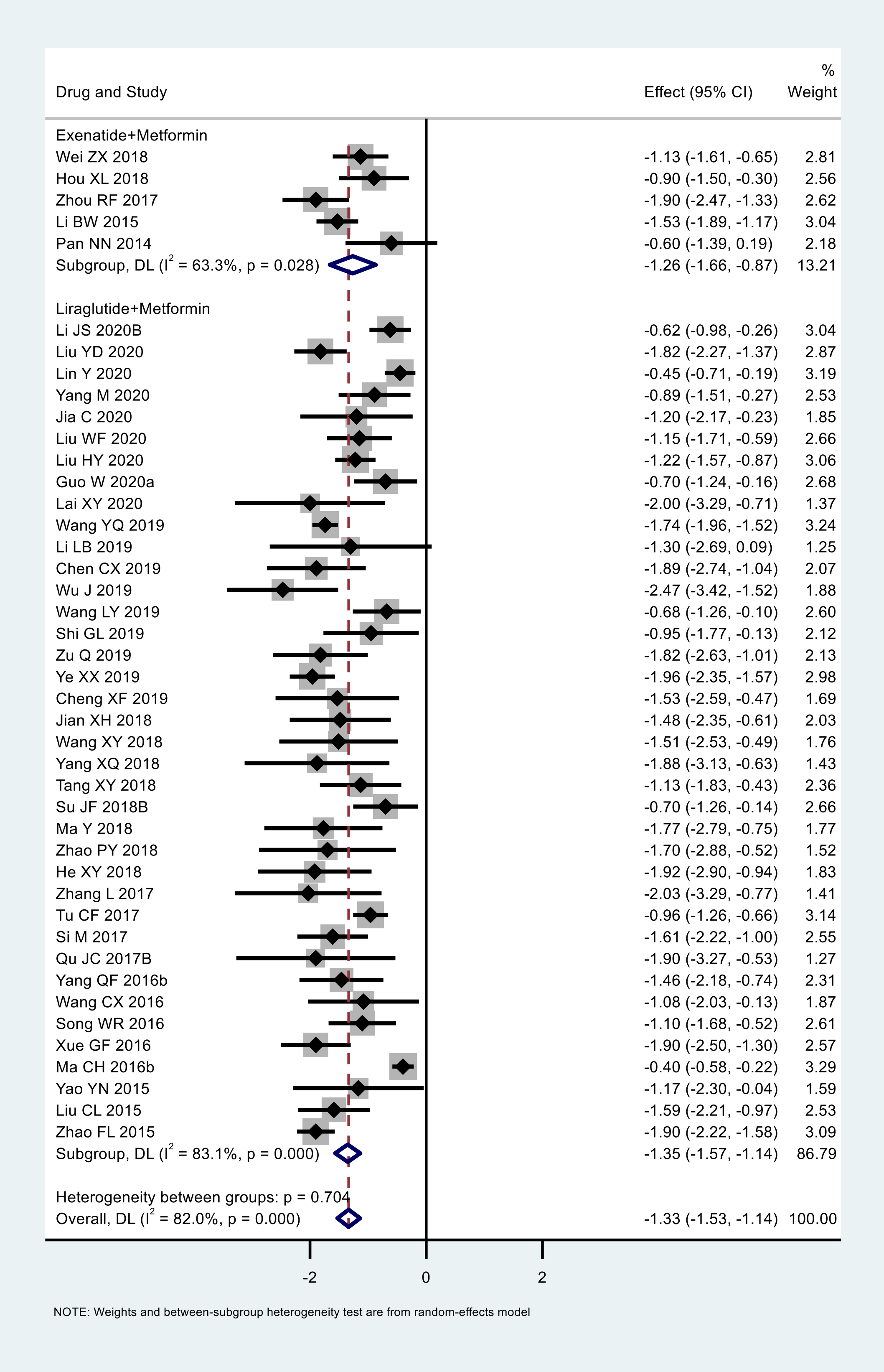

Figure S15. Meta-analysis results for change in fasting plasma glucose (mmol/l) of glucagon-like peptide-1 receptor agonists added to metformin compared with metformin monotherapy

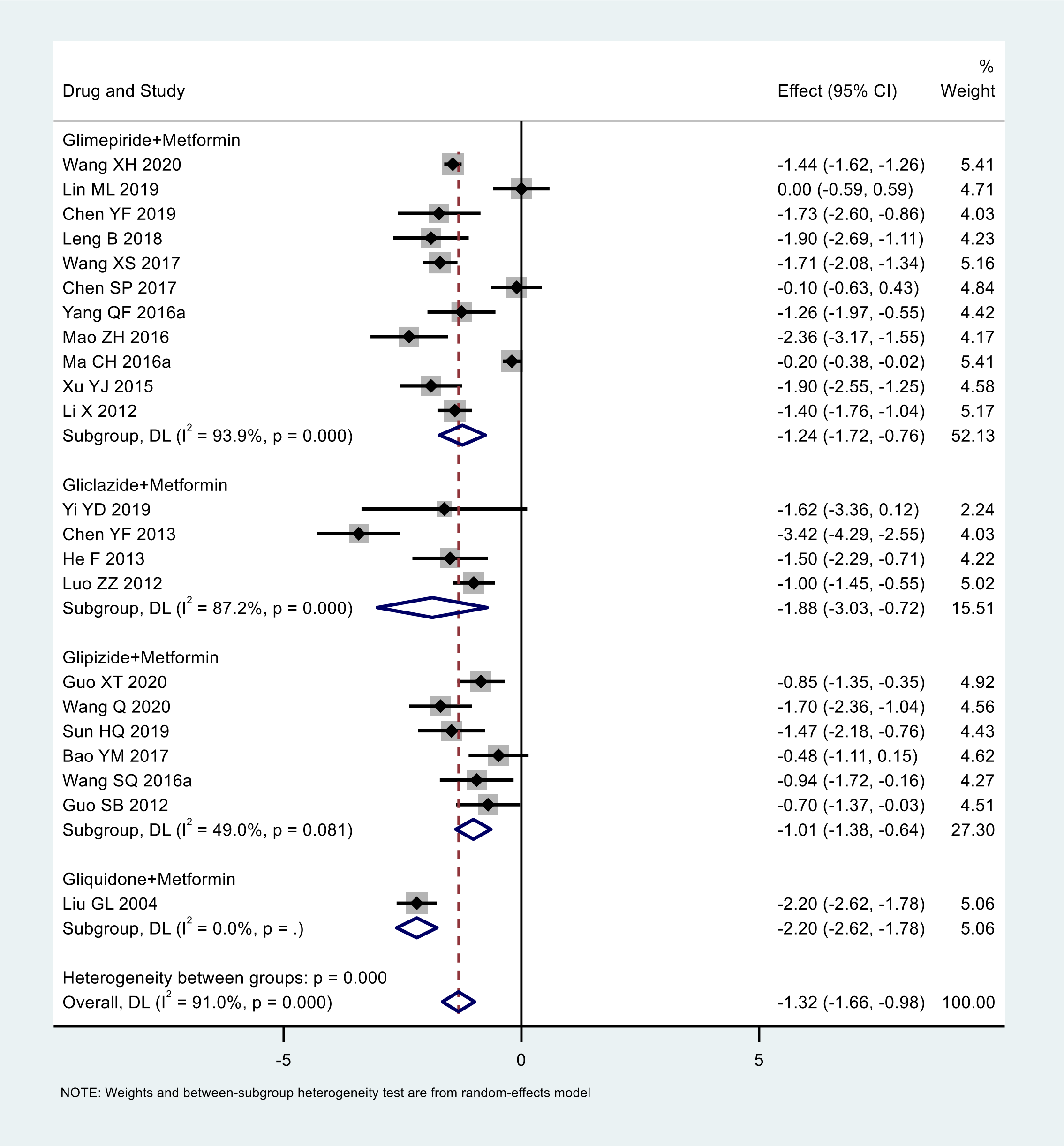

Figure S16. Meta-analysis results for change in fasting plasma glucose (mmol/l) of sulfonylureas added to metformin compared with metformin monotherapy

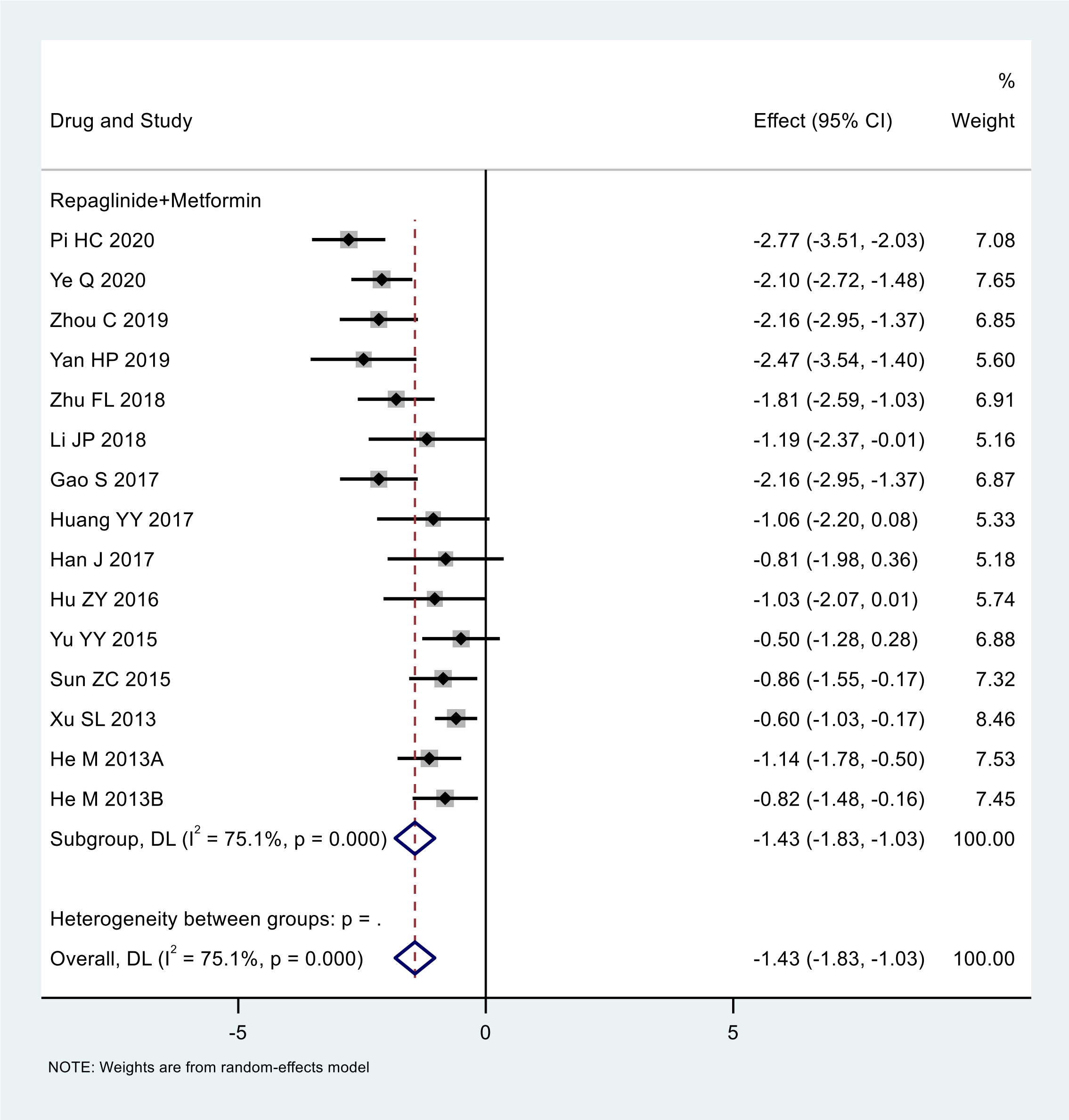

Figure S17. Meta-analysis results for change in fasting plasma glucose (mmol/l) of glinides added to metformin compared with metformin monotherapy

【2h postprandial plasma glucose (mmol/l)】

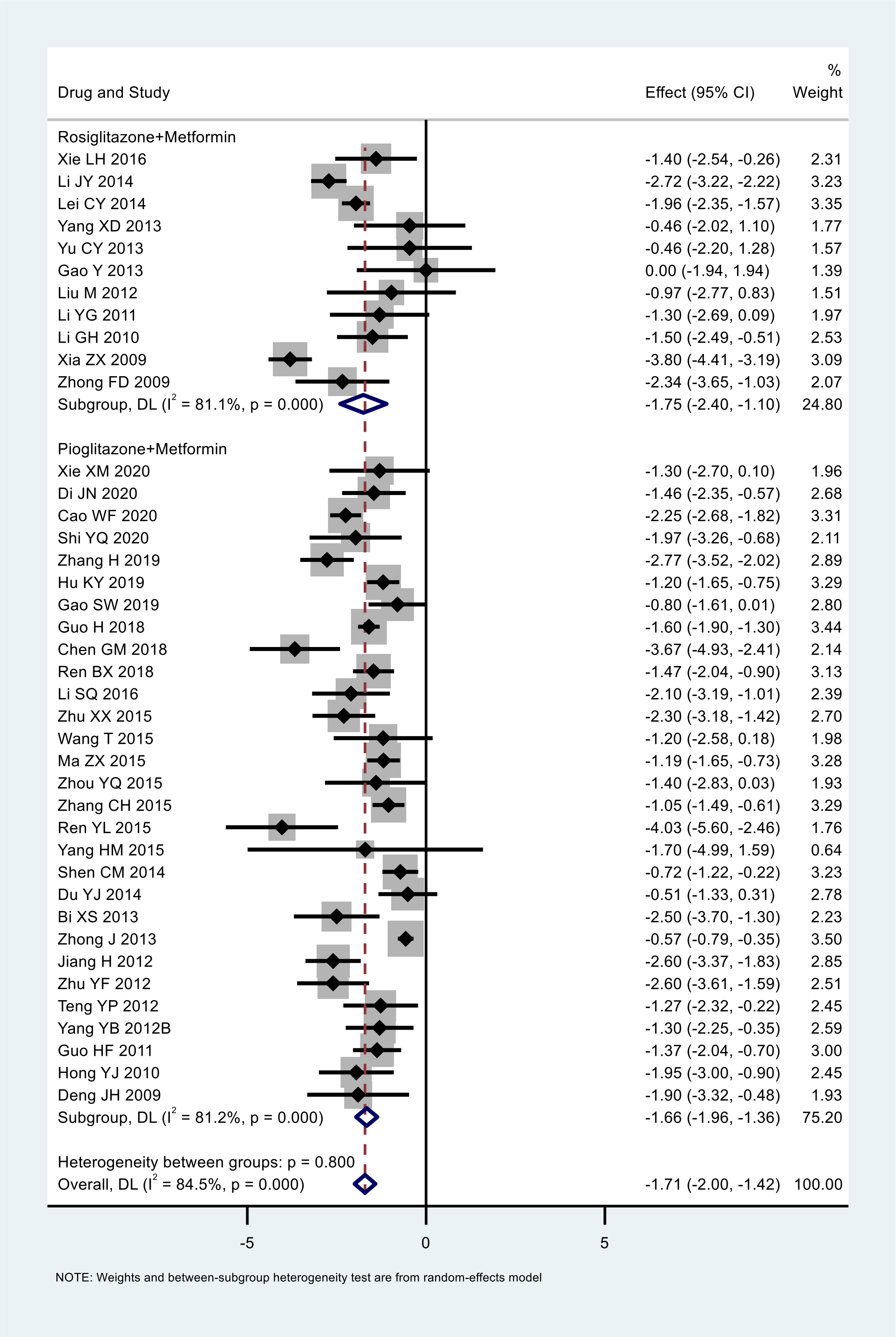

Figure S18. Meta-analysis results for change in 2h postprandial plasma glucose (mmol/l) of thiazolidinediones added to metformin compared with metformin monotherapy

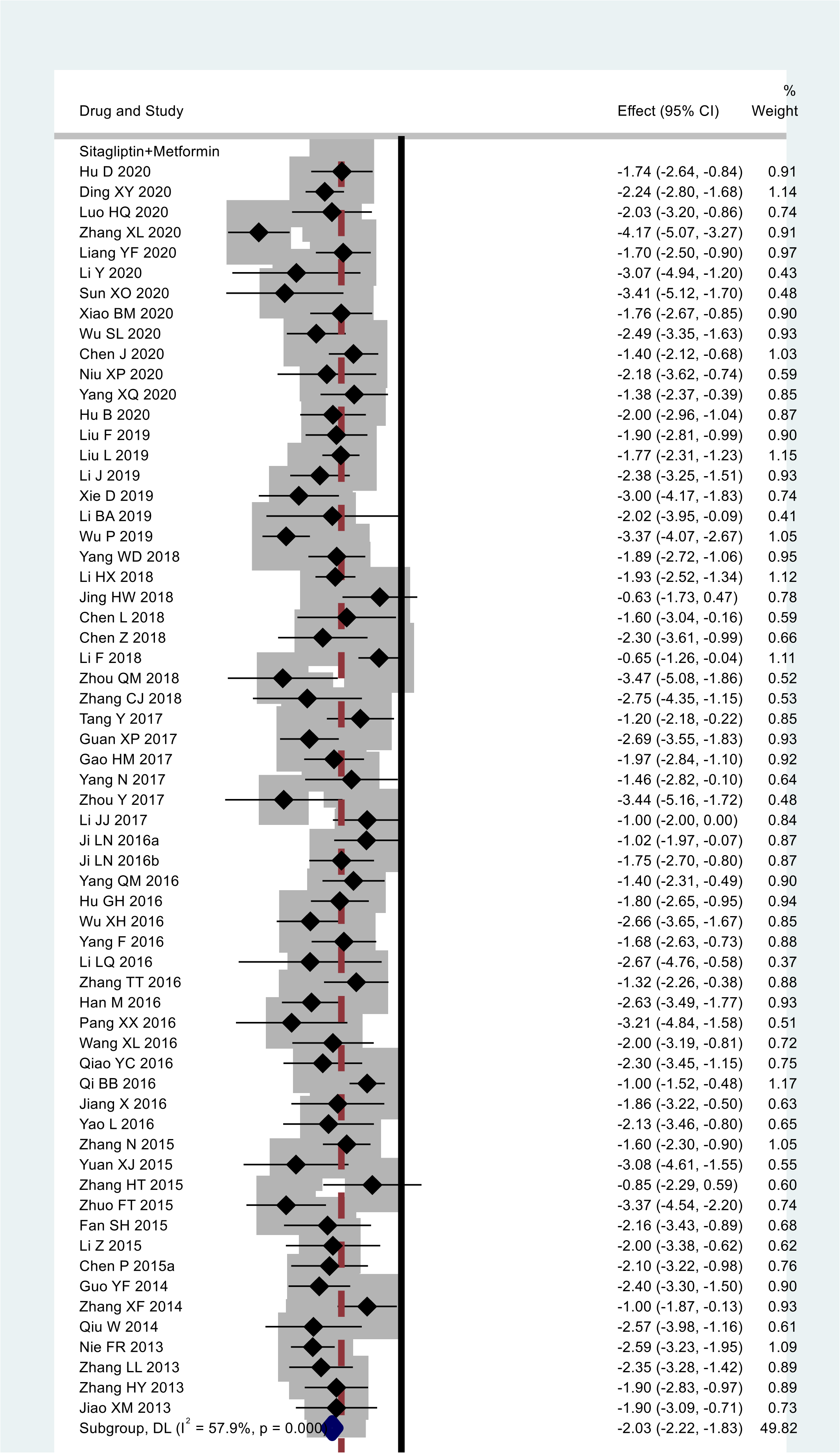

%
Drug and Study
Effect (95% CI)
Weight
Sitagliptin+Metformin
Hu D 2020 -1.74 (-2.64, -0.84) 0.91
Ding XY 2020 -2.24 (-2.80, -1.68) 1.14
Luo HQ 2020 -2.03 (-3.20, -0.86) 0.74
Zhang XL 2020 -4.17 (-5.07, -3.27) 0.91
Liang YF 2020 -1.70 (-2.50, -0.90) 0.97
Li Y 2020 -3.07 (-4.94, -1.20) 0.43
Sun XO 2020 -3.41 (-5.12, -1.70) 0.48
Xiao BM 2020 -1.76 (-2.67, -0.85) 0.90
Wu SL 2020 -2.49 (-3.35, -1.63) 0.93
Chen J 2020 -1.40 (-2.12, -0.68) 1.03
Niu XP 2020 -2.18 (-3.62, -0.74) 0.59
Yang XQ 2020 -1.38 (-2.37, -0.39) 0.85
Hu B 2020 -2.00 (-2.96, -1.04) 0.87
Liu F 2019 -1.90 (-2.81, -0.99) 0.90
Liu L 2019 -1.77 (-2.31, -1.23) 1.15
Li J 2019 -2.38 (-3.25, -1.51) 0.93
Xie D 2019 -3.00 (-4.17, -1.83) 0.74
Li BA 2019 -2.02 (-3.95, -0.09) 0.41
Wu P 2019 -3.37 (-4.07, -2.67) 1.05
Yang WD 2018 -1.89 (-2.72, -1.06) 0.95
Li HX 2018 -1.93 (-2.52, -1.34) 1.12
Jing HW 2018 -0.63 (-1.73, 0.47) 0.78
Chen L 2018 -1.60 (-3.04, -0.16) 0.59
Chen Z 2018 -2.30 (-3.61, -0.99) 0.66
Li F 2018 -0.65 (-1.26, -0.04) 1.11
Zhou QM 2018 -3.47 (-5.08, -1.86) 0.52
Zhang CJ 2018 -2.75 (-4.35, -1.15) 0.53
Tang Y 2017 -1.20 (-2.18, -0.22) 0.85
Guan XP 2017 -2.69 (-3.55, -1.83) 0.93
Gao HM 2017 -1.97 (-2.84, -1.10) 0.92
Yang N 2017 -1.46 (-2.82, -0.10) 0.64
Zhou Y 2017 -3.44 (-5.16, -1.72) 0.48
Li JJ 2017 -1.00 (-2.00, 0.00) 0.84
Ji LN 2016a -1.02 (-1.97, -0.07) 0.87
Ji LN 2016b -1.75 (-2.70, -0.80) 0.87
Yang QM 2016 -1.40 (-2.31, -0.49) 0.90
Hu GH 2016 -1.80 (-2.65, -0.95) 0.94
Wu XH 2016 -2.66 (-3.65, -1.67) 0.85
Yang F 2016 -1.68 (-2.63, -0.73) 0.88
Li LQ 2016 -2.67 (-4.76, -0.58) 0.37
Zhang TT 2016 -1.32 (-2.26, -0.38) 0.88
Han M 2016 -2.63 (-3.49, -1.77) 0.93
Pang XX 2016 -3.21 (-4.84, -1.58) 0.51
Wang XL 2016 -2.00 (-3.19, -0.81) 0.72
Qiao YC 2016 -2.30 (-3.45, -1.15) 0.75
Qi BB 2016 -1.00 (-1.52, -0.48) 1.17
Jiang X 2016 -1.86 (-3.22, -0.50) 0.63
Yao L 2016 -2.13 (-3.46, -0.80) 0.65
Zhang N 2015 -1.60 (-2.30, -0.90) 1.05
Yuan XJ 2015 -3.08 (-4.61, -1.55) 0.55
Zhang HT 2015 -0.85 (-2.29, 0.59) 0.60
Zhuo FT 2015 -3.37 (-4.54, -2.20) 0.74
Fan SH 2015 -2.16 (-3.43, -0.89) 0.68
Li Z 2015 -2.00 (-3.38, -0.62) 0.62
Chen P 2015a -2.10 (-3.22, -0.98) 0.76
Guo YF 2014 -2.40 (-3.30, -1.50) 0.90
Zhang XF 2014 -1.00 (-1.87, -0.13) 0.93
Qiu W 2014 -2.57 (-3.98, -1.16) 0.61
Nie FR 2013 -2.59 (-3.23, -1.95) 1.09
Zhang LL 2013 -2.35 (-3.28, -1.42) 0.89
Zhang HY 2013 -1.90 (-2.83, -0.97) 0.89
Jiao XM 2013 -1.90 (-3.09, -0.71) 0.73
Subgroup, DL (I² = 57.9%, p = 0.000) -2.03 (-2.22, -1.83) 49.82

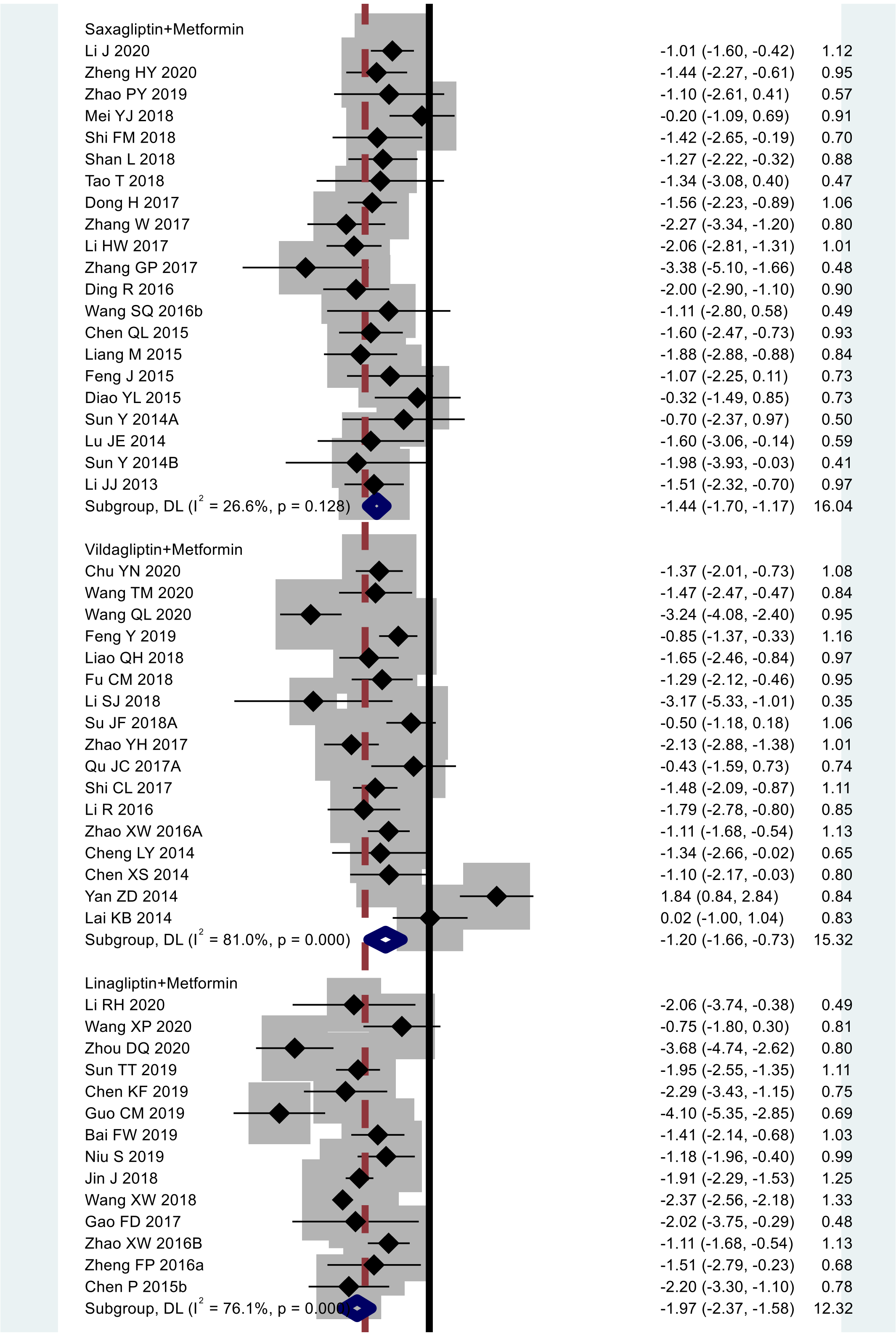
Saxagliptin+Metformin
Li J 2020 -1.01 (-1.60, -0.42) 1.12
Zheng HY 2020 -1.44 (-2.27, -0.61) 0.95
Zhao PY 2019 -1.10 (-2.61, 0.41) 0.57
Mei YJ 2018 -0.20 (-1.09, 0.69) 0.91
Shi FM 2018 -1.42 (-2.65, -0.19) 0.70
Shan L 2018 -1.27 (-2.22, -0.32) 0.88
Tao T 2018 -1.34 (-3.08, 0.40) 0.47
Dong H 2017 -1.56 (-2.23, -0.89) 1.06
Zhang W 2017 -2.27 (-3.34, -1.20) 0.80
Li HW 2017 -2.06 (-2.81, -1.31) 1.01
Zhang GP 2017 -3.38 (-5.10, -1.66) 0.48
Ding R 2016 -2.00 (-2.90, -1.10) 0.90
Wang SQ 2016b -1.11 (-2.80, 0.58) 0.49
Chen QL 2015 -1.60 (-2.47, -0.73) 0.93
Liang M 2015 -1.88 (-2.88, -0.88) 0.84
Feng J 2015 -1.07 (-2.25, 0.11) 0.73
Diao YL 2015 -0.32 (-1.49, 0.85) 0.73
Sun Y 2014A -0.70 (-2.37, 0.97) 0.50
Lu JE 2014 -1.60 (-3.06, -0.14) 0.59
Sun Y 2014B -1.98 (-3.93, -0.03) 0.41
Li JJ 2013 -1.51 (-2.32, -0.70) 0.97
Subgroup, DL (I^2 = 26.6%, p = 0.128) -1.44 (-1.70, -1.17) 16.04
Vildagliptin+Metformin
Chu YN 2020 -1.37 (-2.01, -0.73) 1.08
Wang TM 2020 -1.47 (-2.47, -0.47) 0.84
Wang QL 2020 -3.24 (-4.08, -2.40) 0.95
Feng Y 2019 -0.85 (-1.37, -0.33) 1.16
Liao QH 2018 -1.65 (-2.46, -0.84) 0.97
Fu CM 2018 -1.29 (-2.12, -0.46) 0.95
Li SJ 2018 -3.17 (-5.33, -1.01) 0.35
Su JF 2018A -0.50 (-1.18, 0.18) 1.06
Zhao YH 2017 -2.13 (-2.88, -1.38) 1.01
Qu JC 2017A -0.43 (-1.59, 0.73) 0.74
Shi CL 2017 -1.48 (-2.09, -0.87) 1.11
Li R 2016 -1.79 (-2.78, -0.80) 0.85
Zhao XW 2016A -1.11 (-1.68, -0.54) 1.13
Cheng LY 2014 -1.34 (-2.66, -0.02) 0.65
Chen XS 2014 -1.10 (-2.17, -0.03) 0.80
Yan ZD 2014 1.84 (0.84, 2.84) 0.84
Lai KB 2014 0.02 (-1.00, 1.04) 0.83
Subgroup, DL (I^2 = 81.0%, p = 0.000) -1.20 (-1.66, -0.73) 15.32
Linagliptin+Metformin
Li RH 2020 -2.06 (-3.74, -0.38) 0.49
Wang XP 2020 -0.75 (-1.80, 0.30) 0.81
Zhou DQ 2020 -3.68 (-4.74, -2.62) 0.80
Sun TT 2019 -1.95 (-2.55, -1.35) 1.11
Chen KF 2019 -2.29 (-3.43, -1.15) 0.75
Guo CM 2019 -4.10 (-5.35, -2.85) 0.69
Bai FW 2019 -1.41 (-2.14, -0.68) 1.03
Niu S 2019 -1.18 (-1.96, -0.40) 0.99
Jin J 2018 -1.91 (-2.29, -1.53) 1.25
Wang XW 2018 -2.37 (-2.56, -2.18) 1.33
Gao FD 2017 -2.02 (-3.75, -0.29) 0.48
Zhao XW 2016B -1.11 (-1.68, -0.54) 1.13
Zheng FP 2016a -1.51 (-2.79, -0.23) 0.68
Chen P 2015b -2.20 (-3.30, -1.10) 0.78
Subgroup, DL (I^2 = 76.1%, p = 0.000) -1.97 (-2.37, -1.58) 12.32

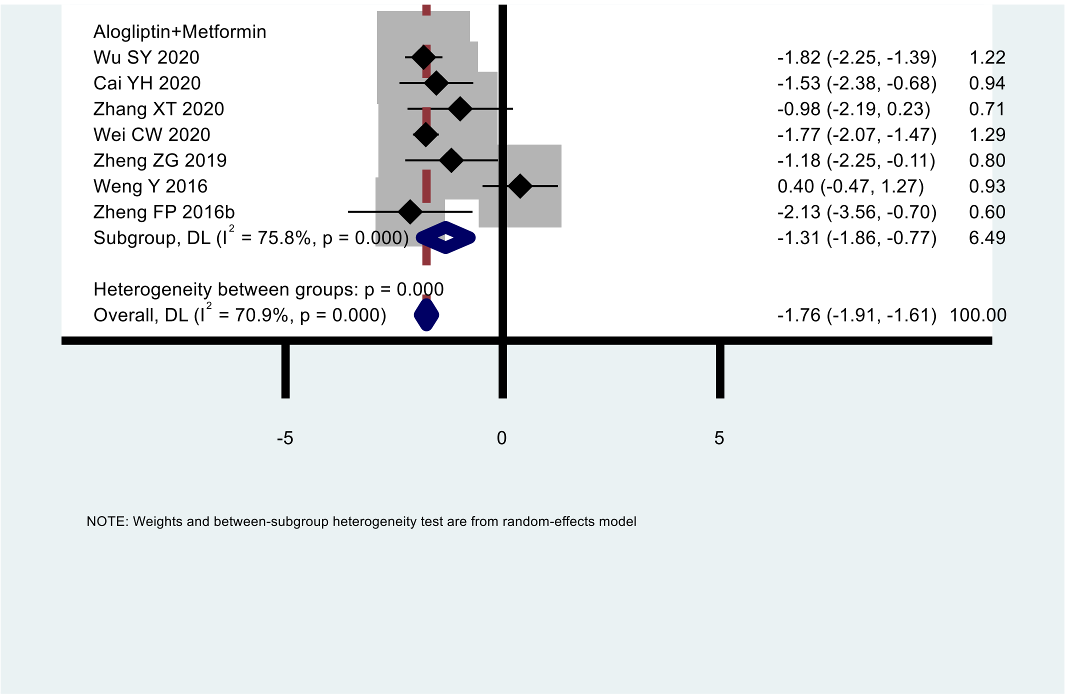

Figure S19. Meta-analysis results for change in 2h postprandial plasma glucose (mmol/l) of dipeptidyl peptidase 4 inhibitors added to metformin compared with metformin monotherapy

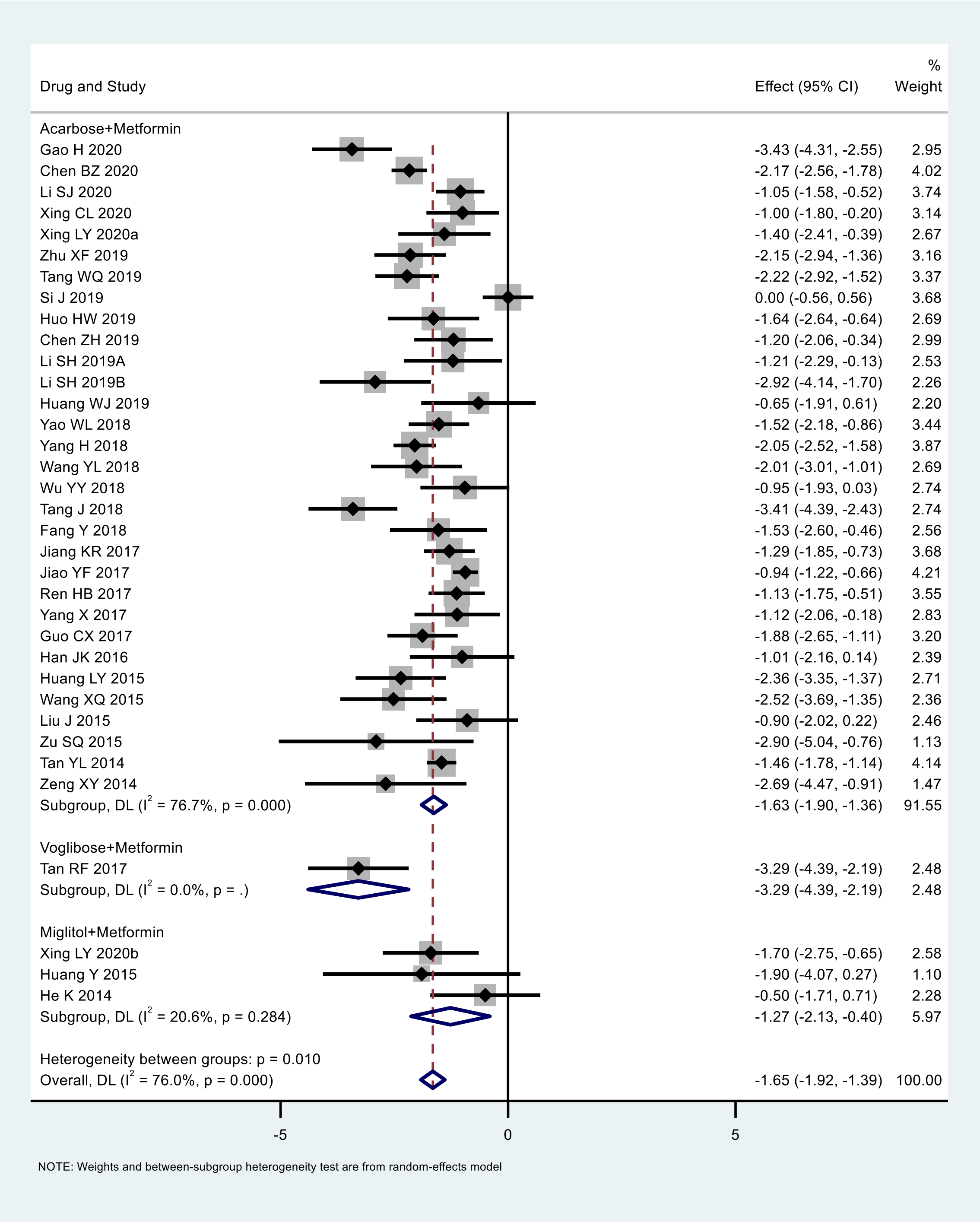

Figure S20. Meta-analysis results for change in 2h postprandial plasma glucose (mmol/l) of α-glucosidase inhibitors added to metformin compared with metformin monotherapy

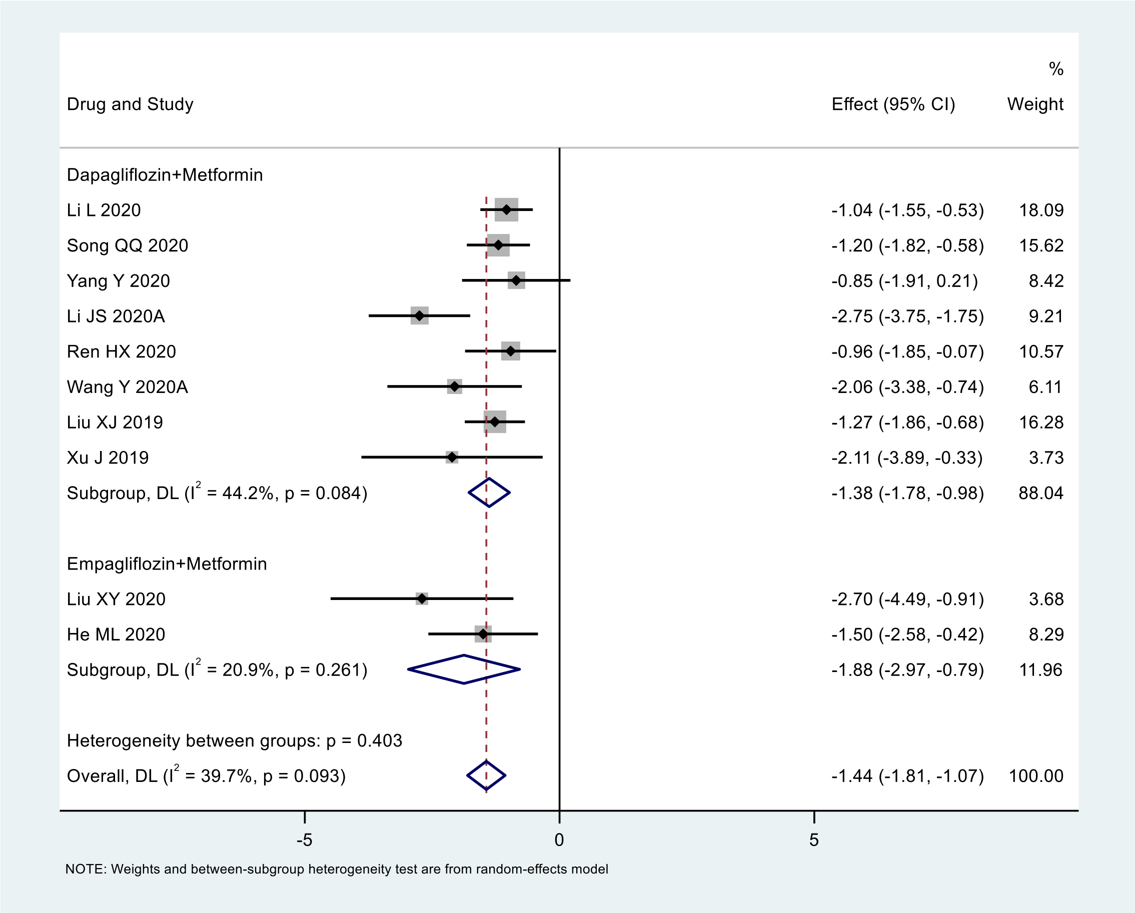

Figure S21. Meta-analysis results for change in 2h postprandial plasma glucose (mmol/l) of sodium-glucose cotransporter 2 inhibitors added to metformin compared with metformin monotherapy

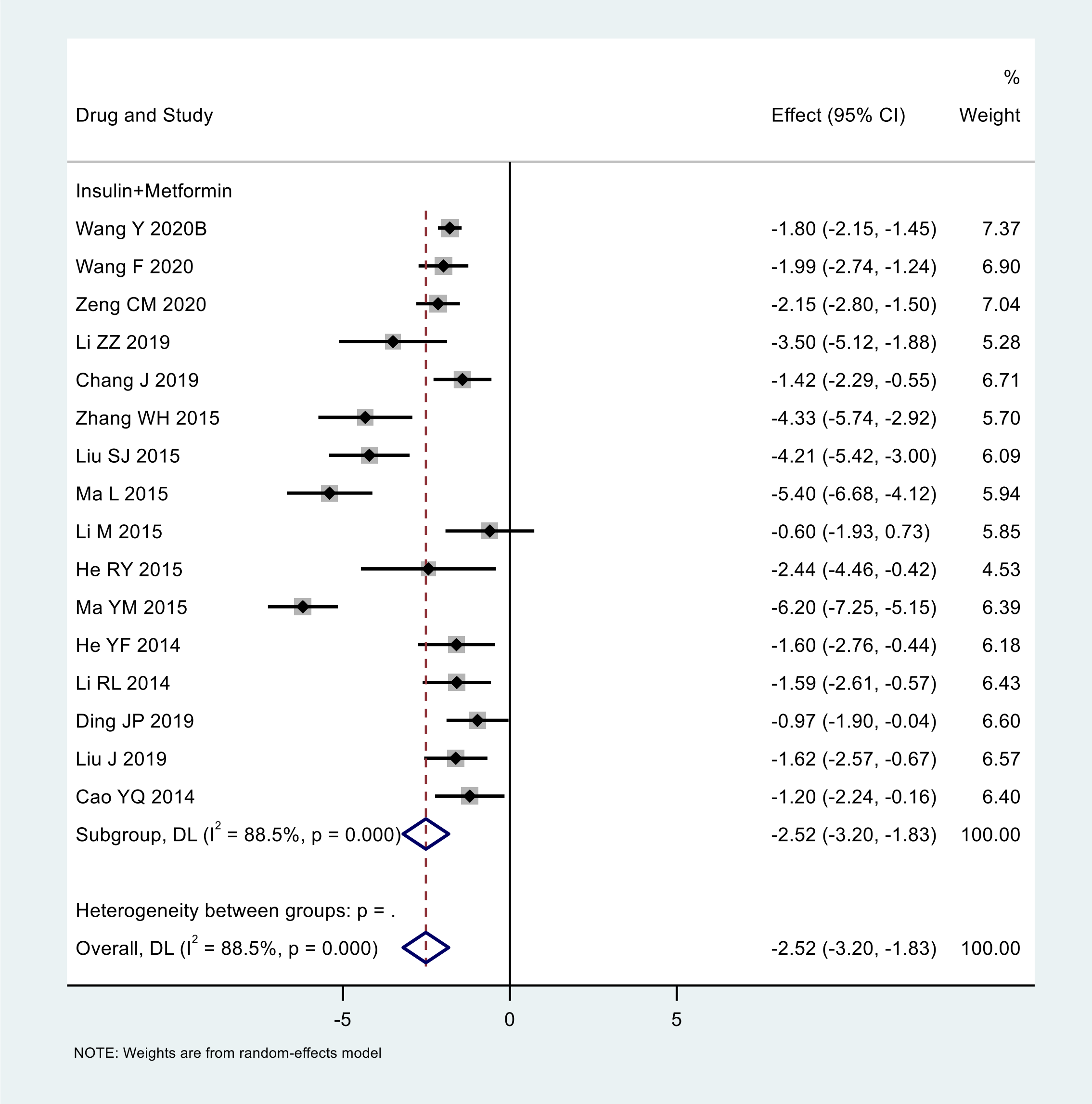

Figure S22. Meta-analysis results for change in 2h postprandial plasma glucose (mmol/l) of insulins added to metformin compared with metformin monotherapy

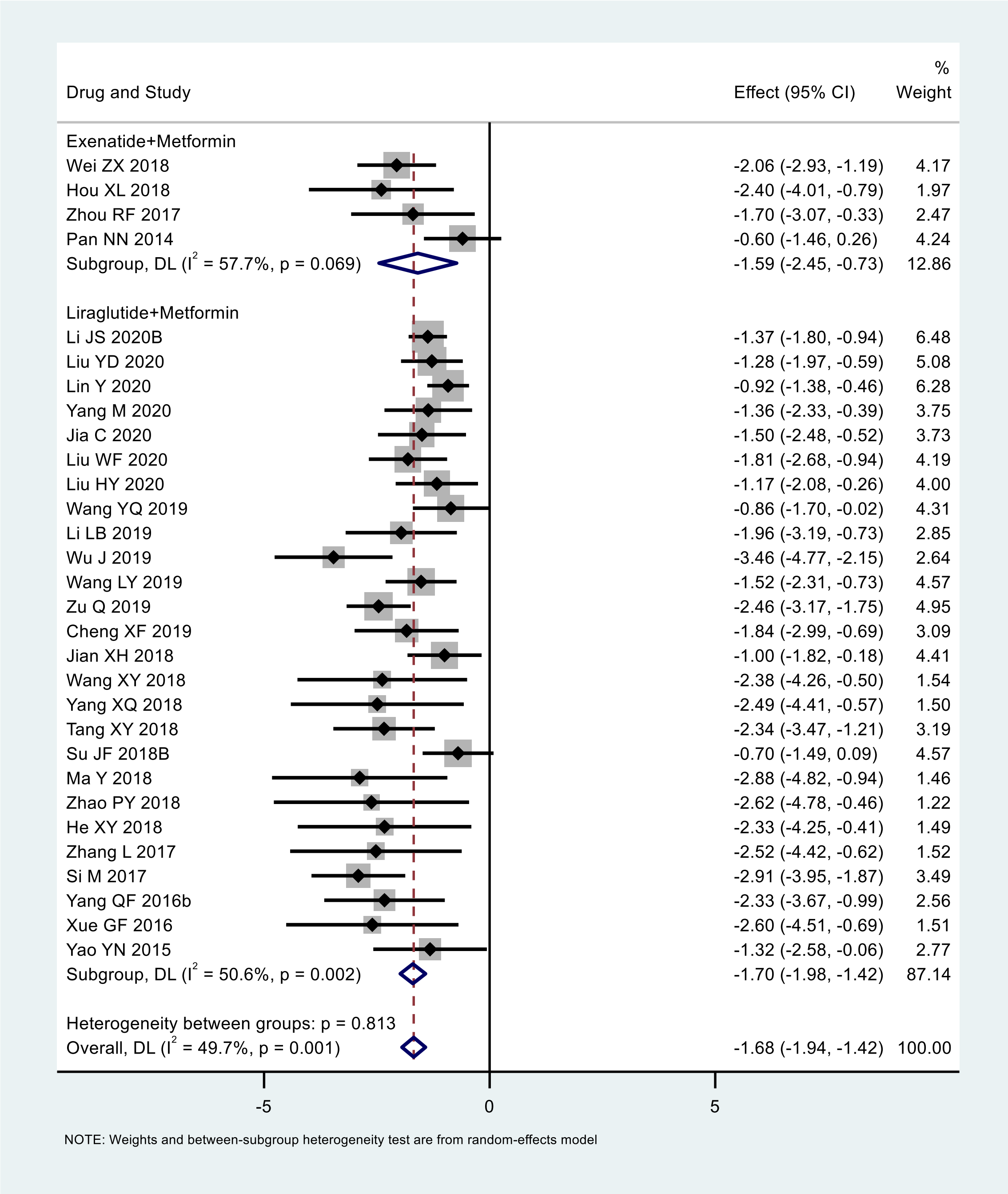

Figure S23. Meta-analysis results for change in 2h postprandial plasma glucose (mmol/l) of glucagon-like peptide-1 receptor agonists added to metformin compared with metformin monotherapy

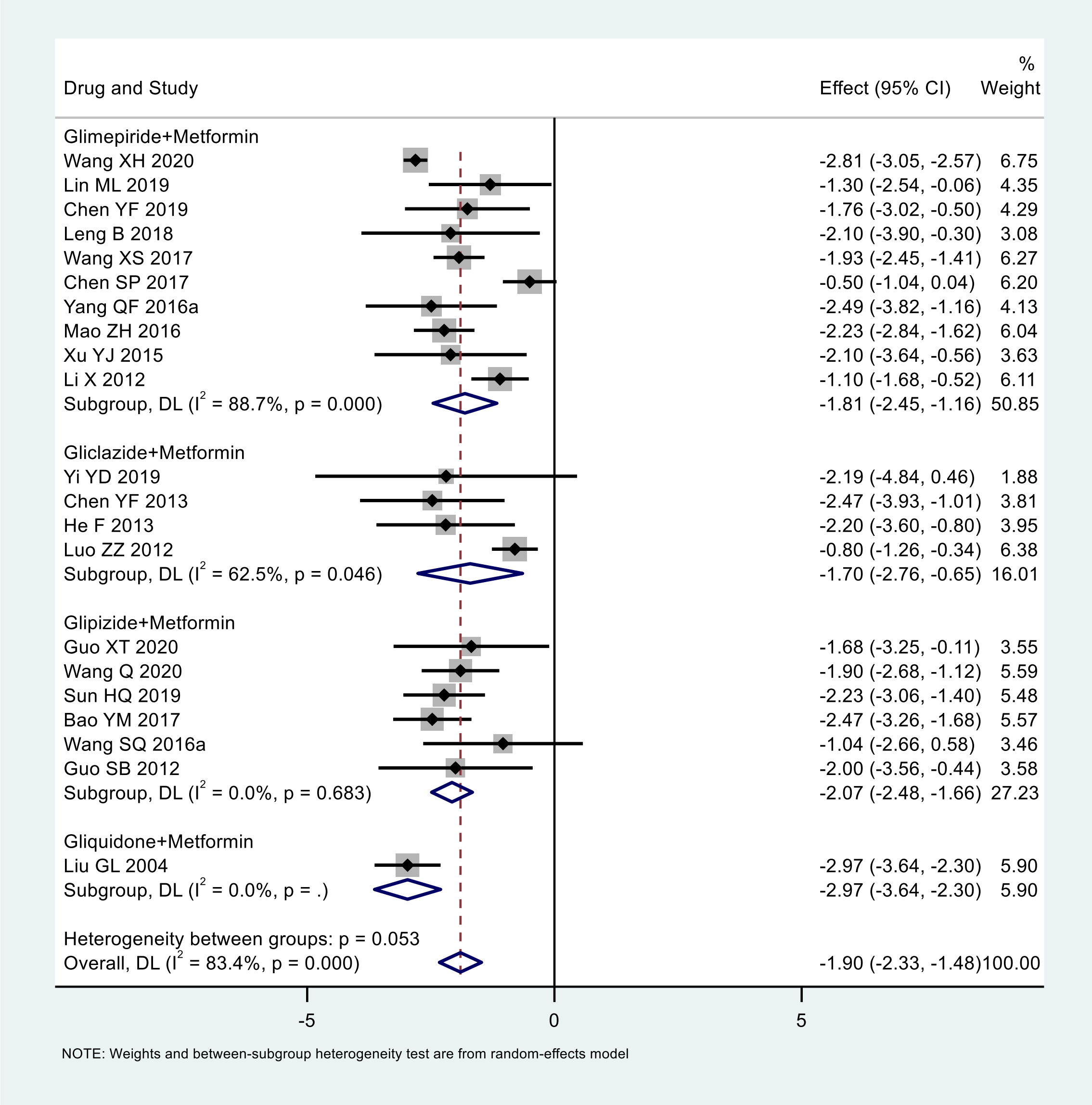

Figure S24. Meta-analysis results for change in 2h postprandial plasma glucose (mmol/l) of sulfonylureas added to metformin compared with metformin monotherapy

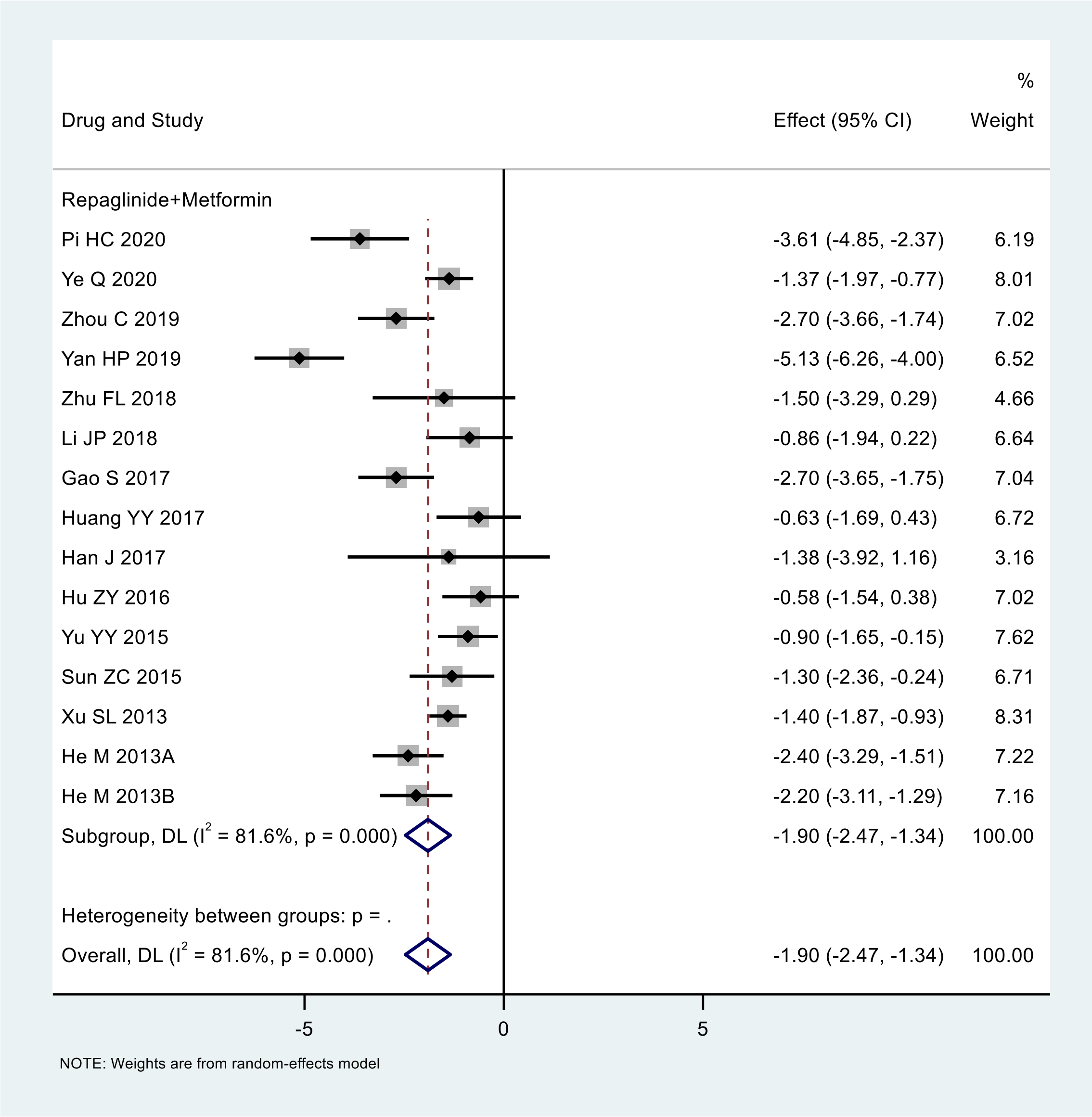

Figure S25. Meta-analysis results for change in 2h postprandial plasma glucose (mmol/l) of glinides added to metformin compared with metformin monotherapy

【Body mass index (kg/m^2)】

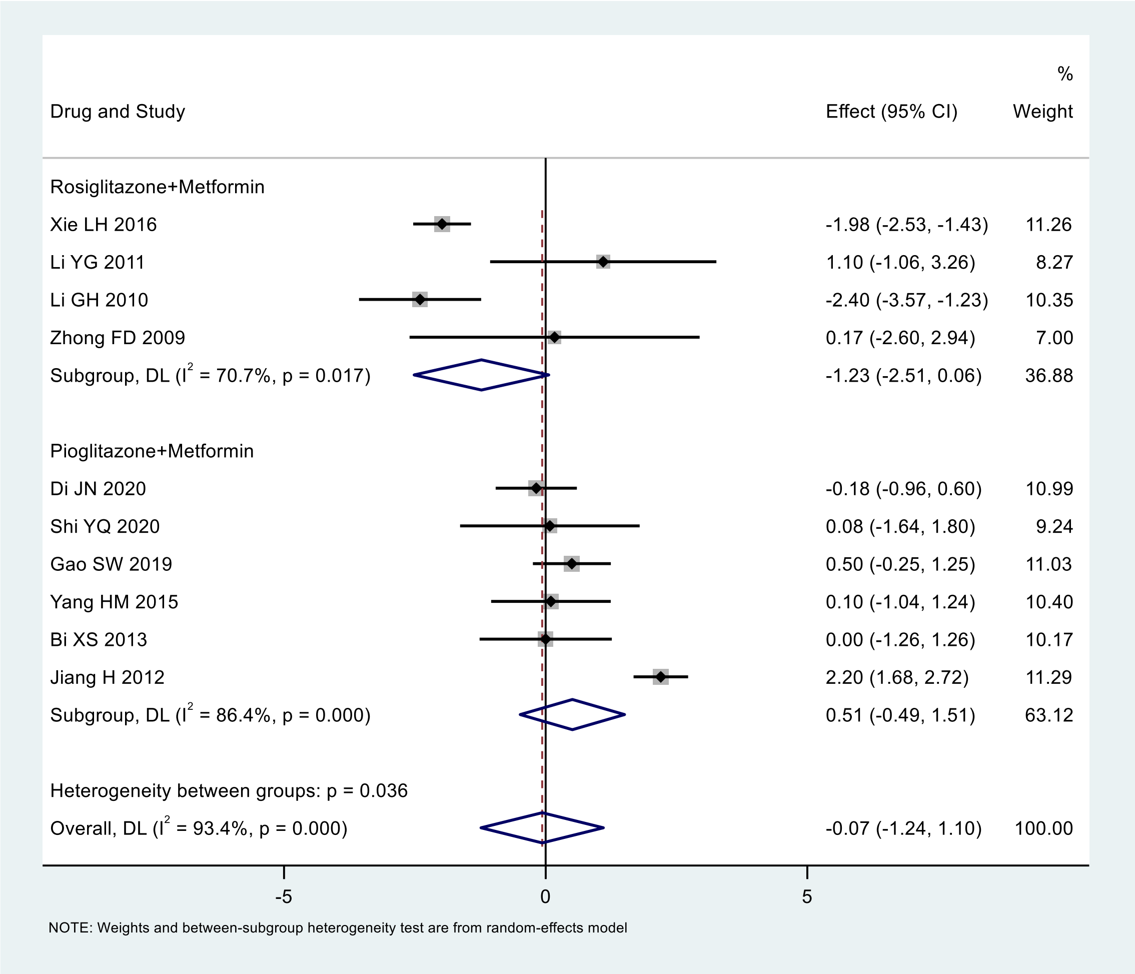

Figure S26. Meta-analysis results for change in body mass index (kg/m^2) of thiazolidinediones added to metformin compared with metformin monotherapy

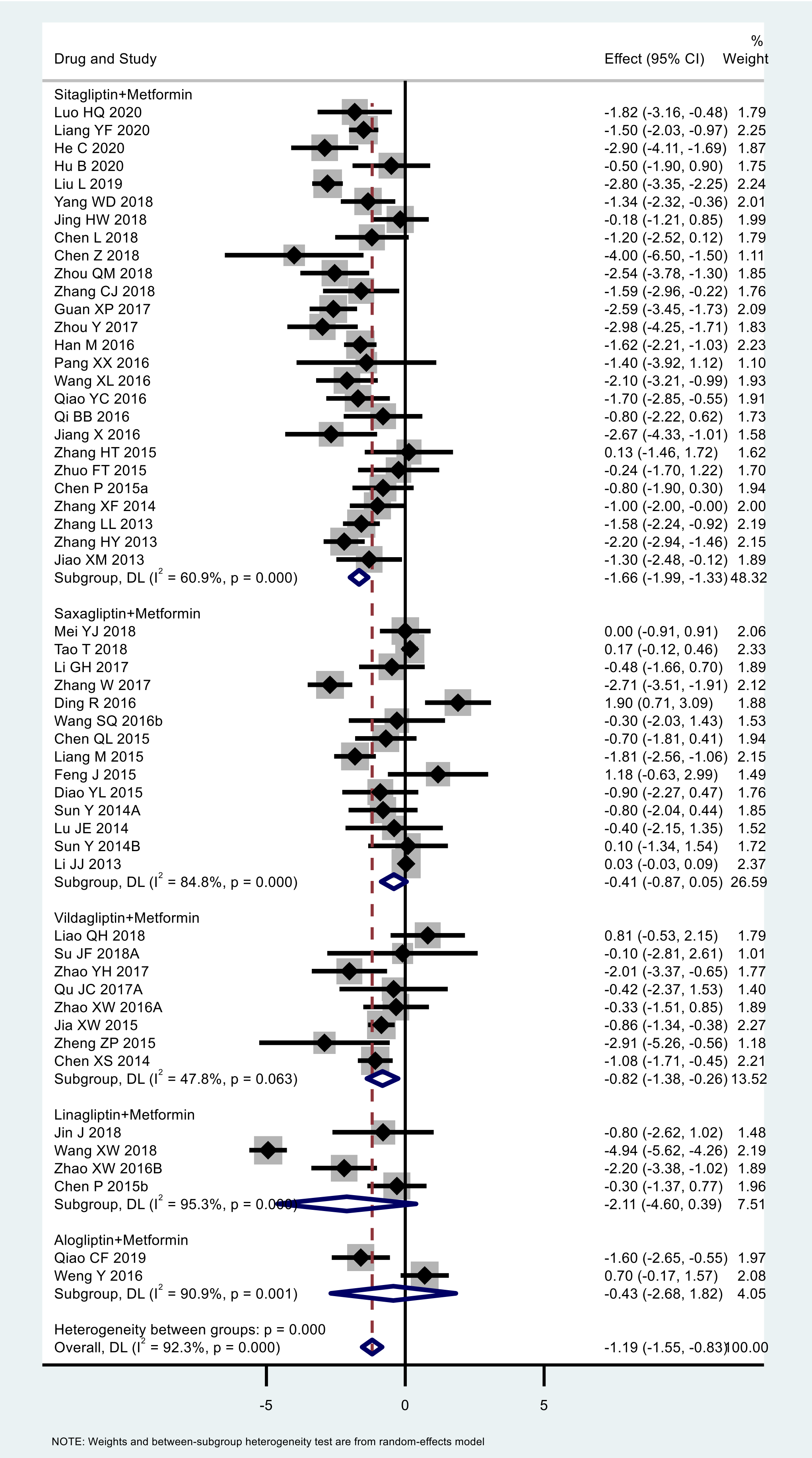

Figure S27. Meta-analysis results for change in body mass index (kg/m^2) of dipeptidyl peptidase 4 inhibitors added to metformin compared with metformin monotherapy

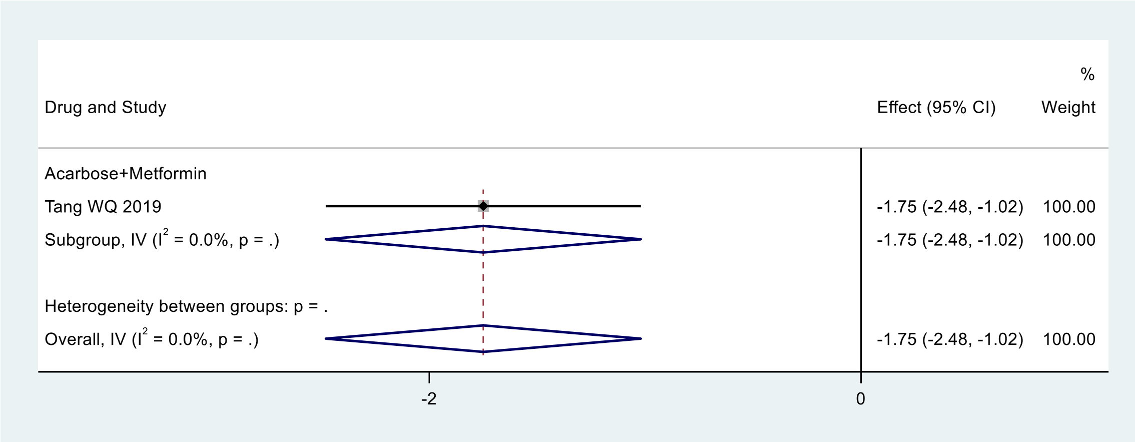

Figure S28. Meta-analysis results for change in body mass index (kg/m^2) of α-glucosidase inhibitors added to metformin compared with metformin monotherapy

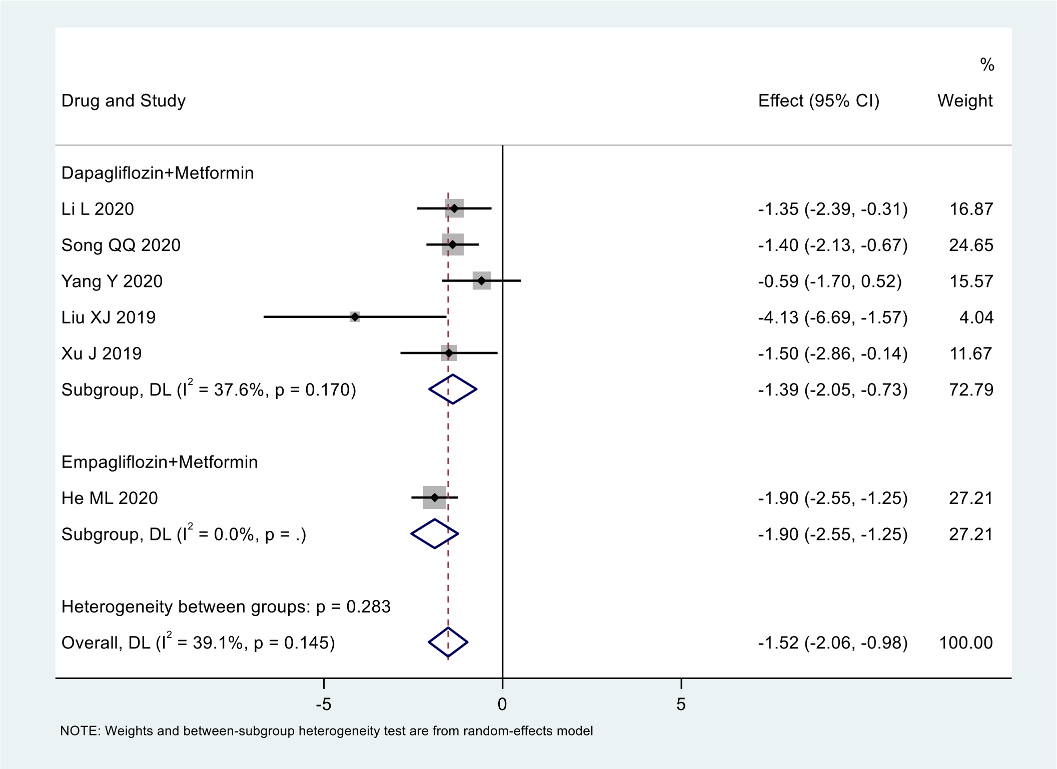

Figure S29. Meta-analysis results for change in body mass index (kg/m^2) of sodium-glucose cotransporter 2 inhibitors added to metformin compared with metformin monotherapy

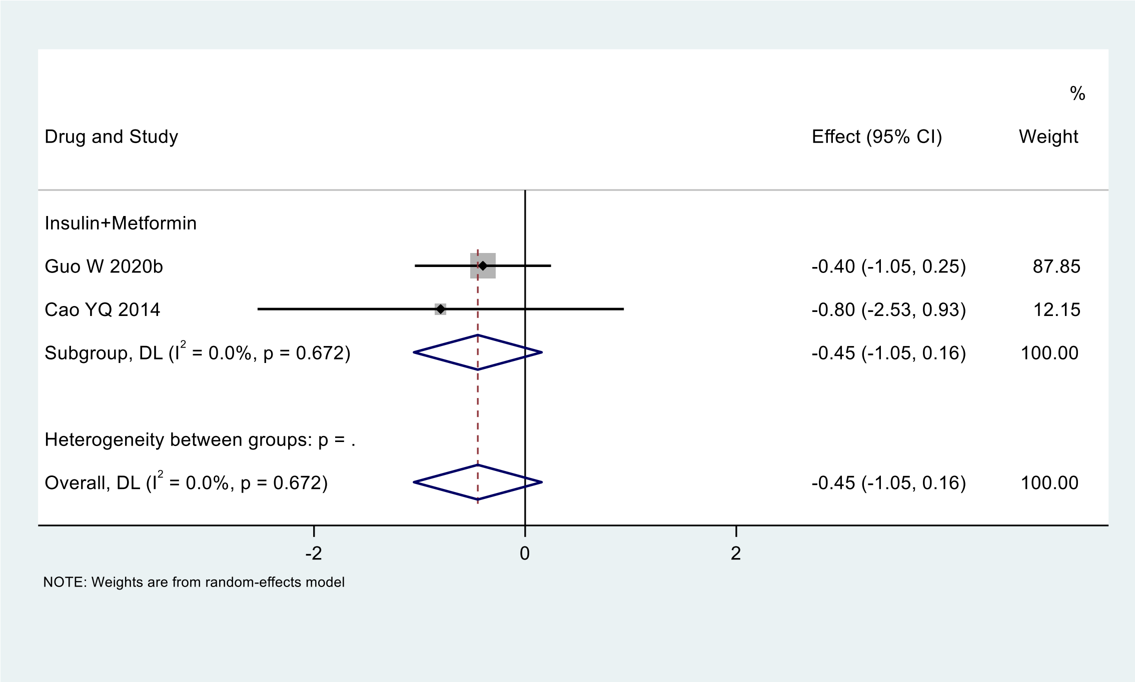

Figure S30. Meta-analysis results for change in body mass index (kg/m^2) of insulins added to metformin compared with metformin monotherapy

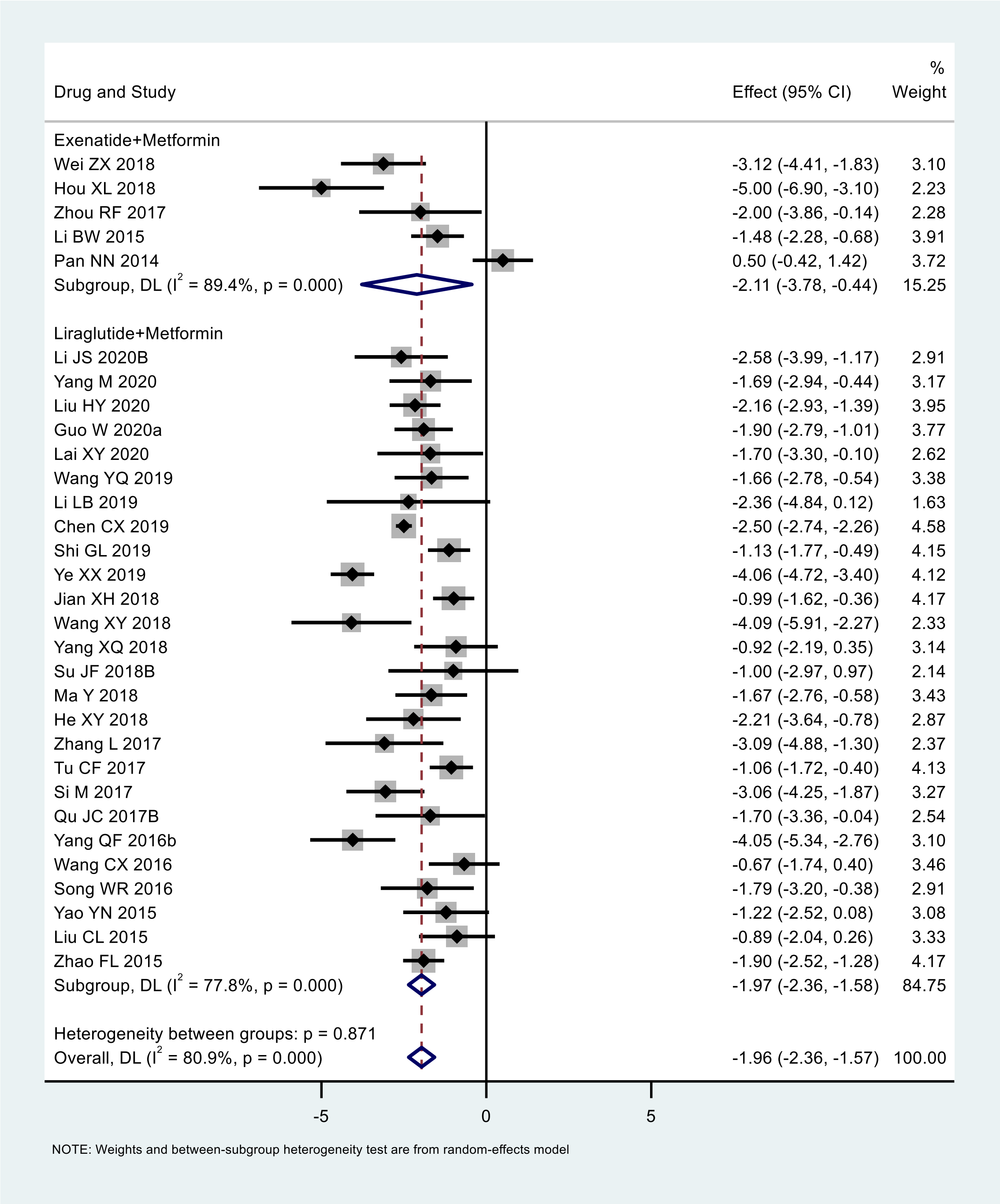

Figure S31. Meta-analysis results for change in body mass index (kg/m^2) of glucagon-like peptide-1 receptor agonists added to metformin compared with metformin monotherapy

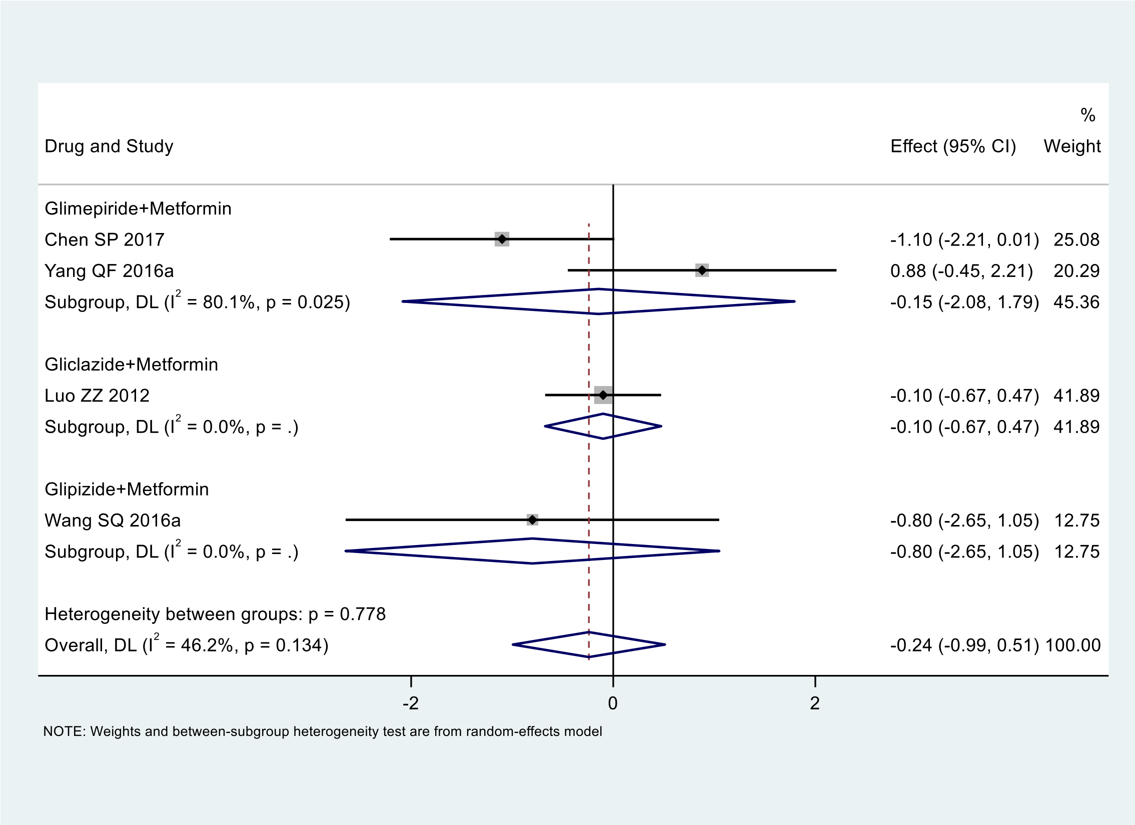

Figure S32. Meta-analysis results for change in body mass index (kg/m^2) of sulfonylureas added to metformin compared with metformin monotherapy

【Total cholesterol (mmol/l)】

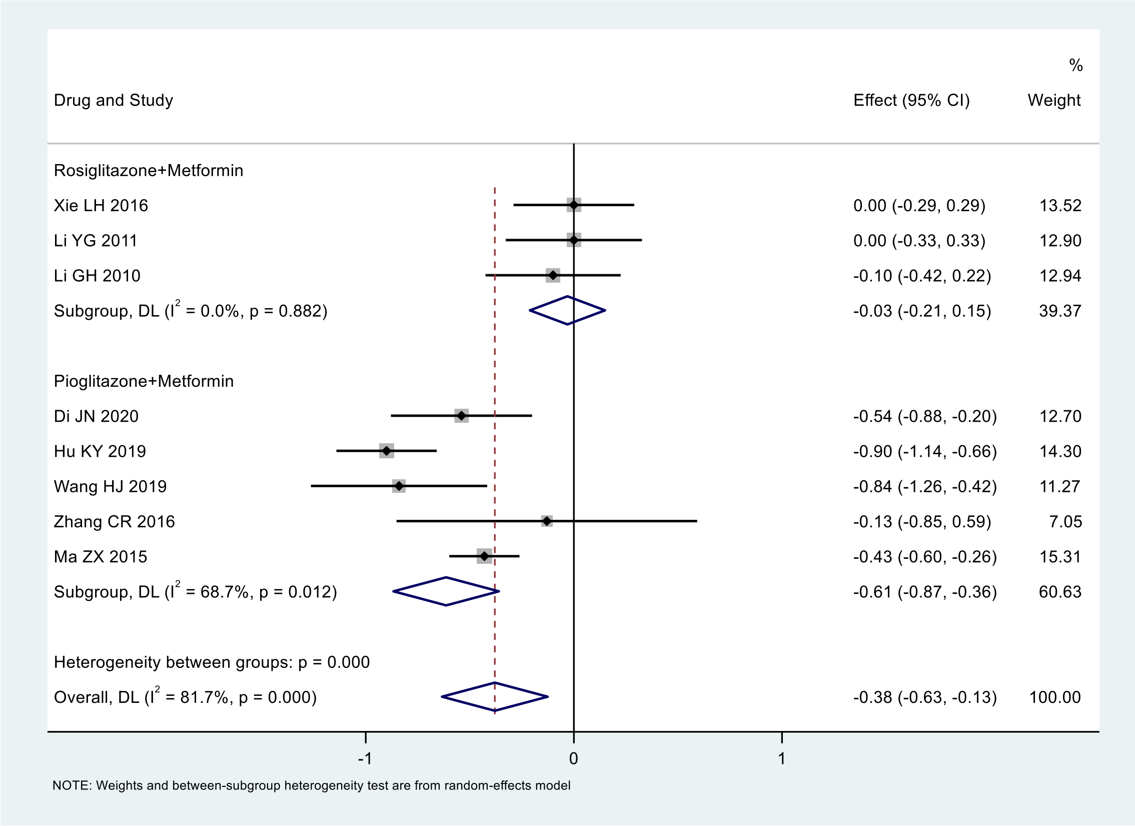

Figure S33. Meta-analysis results for change in total cholesterol (mmol/l) of thiazolidinediones added to metformin compared with metformin monotherapy

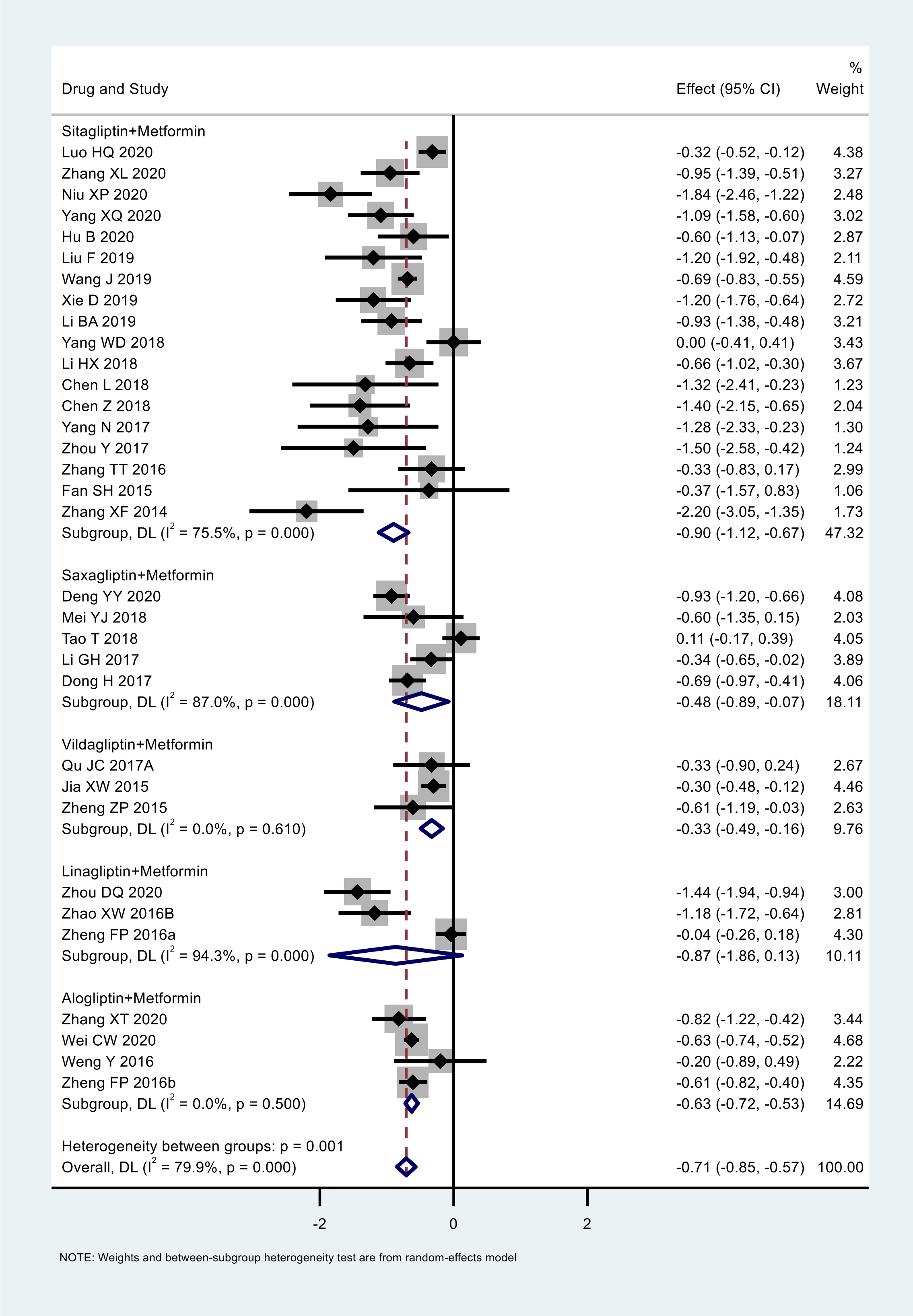

Figure S34. Meta-analysis results for change in total cholesterol (mmol/l) of dipeptidyl peptidase 4 inhibitors added to metformin compared with metformin monotherapy

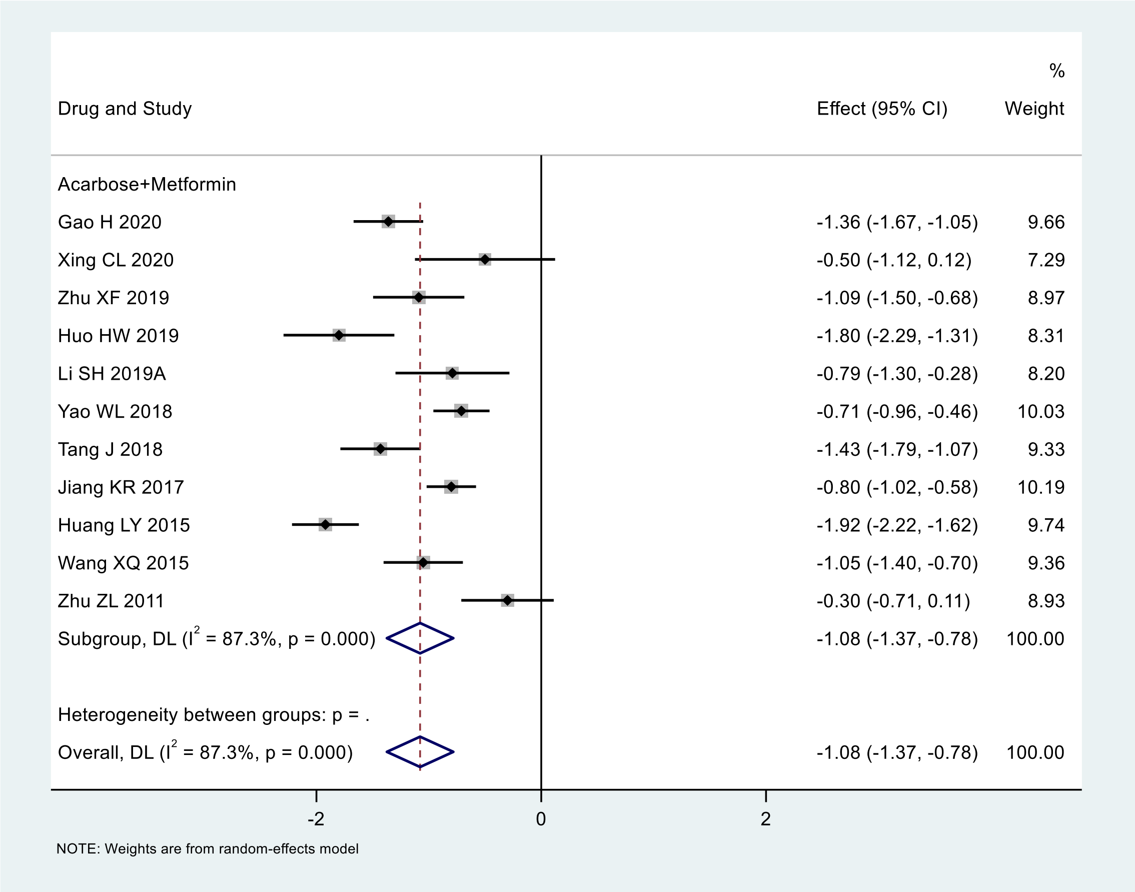

Figure S35. Meta-analysis results for change in total cholesterol (mmol/l) of α-glucosidase inhibitors added to metformin compared with metformin monotherapy

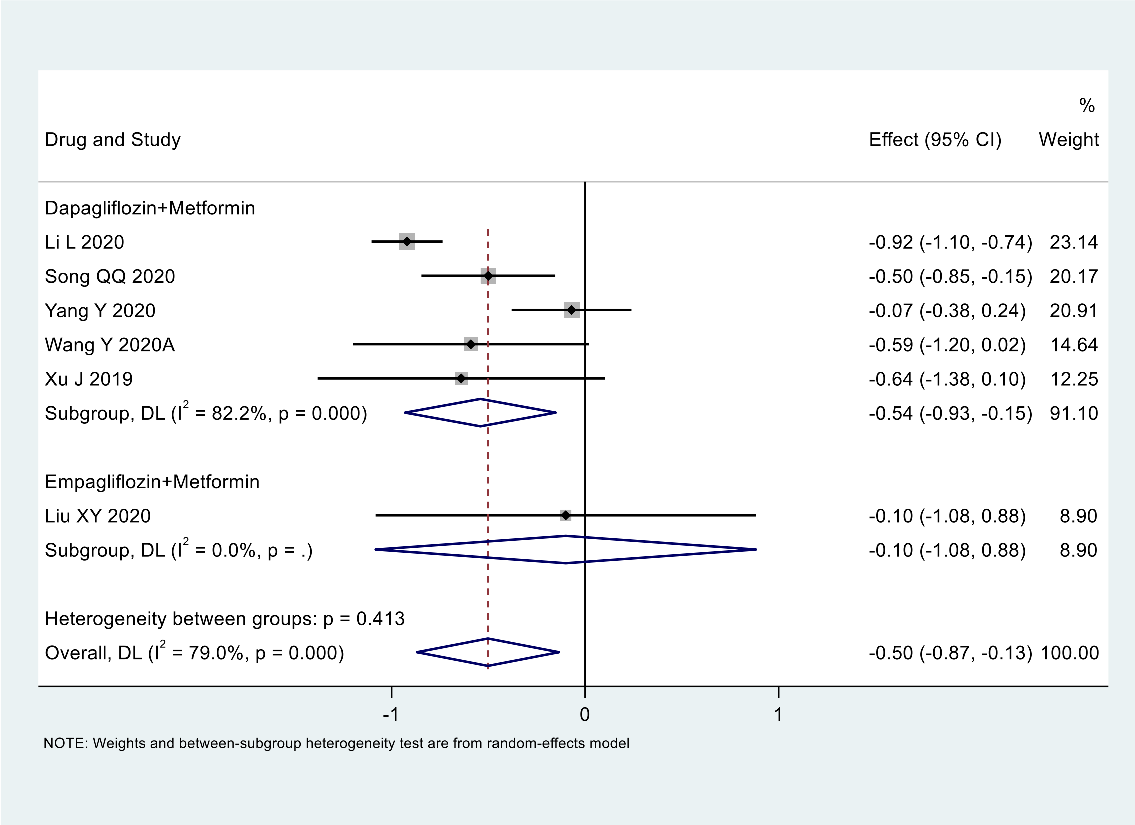

Figure S36. Meta-analysis results for change in total cholesterol (mmol/l) of sodium-glucose cotransporter 2 inhibitors added to metformin compared with metformin monotherapy

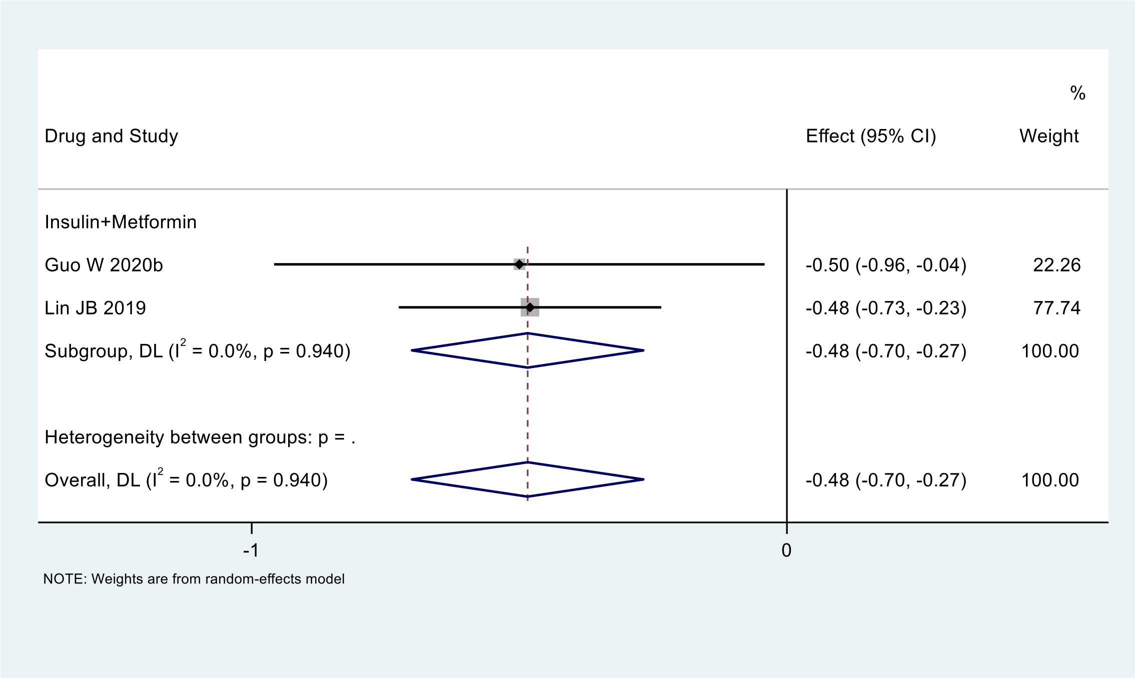

Figure S37. Meta-analysis results for change in total cholesterol (mmol/l) of insulins added to metformin compared with metformin monotherapy

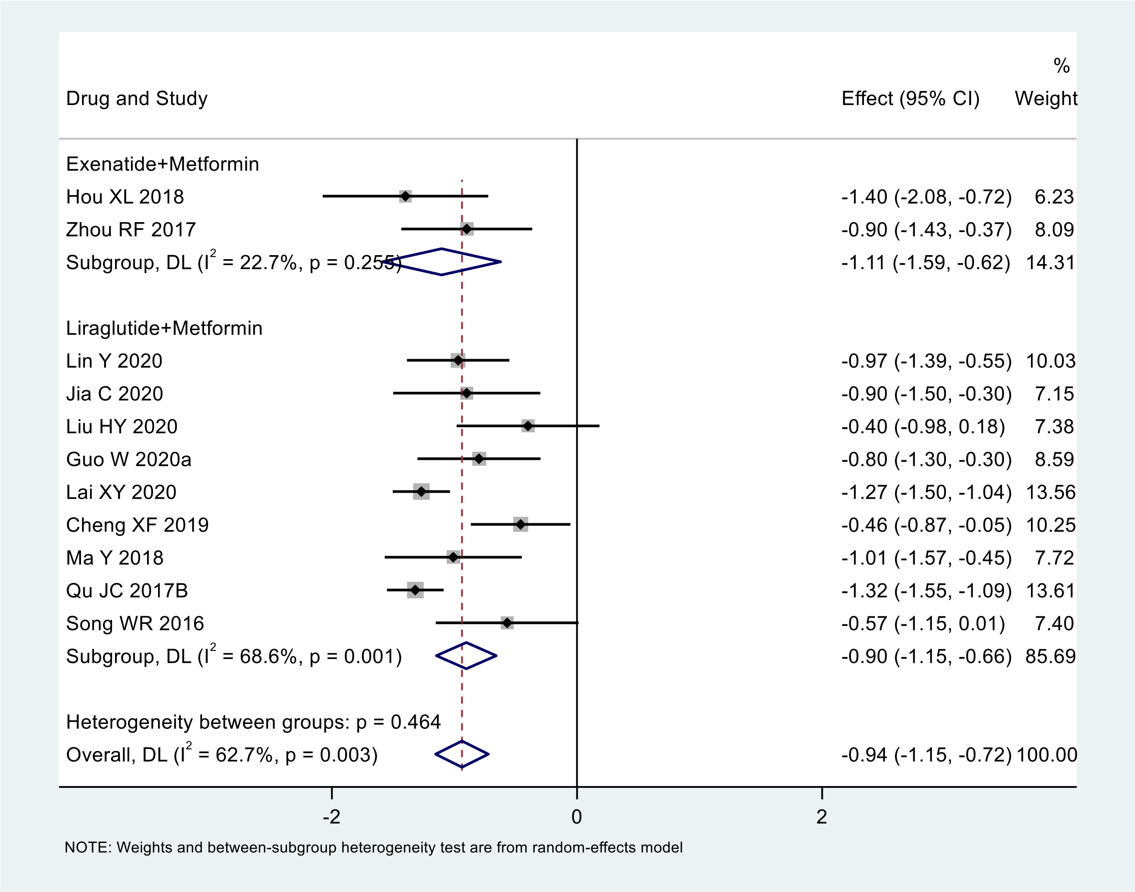

Figure S38. Meta-analysis results for change in total cholesterol (mmol/l) of glucagon-like peptide-1 receptor agonists added to metformin compared with metformin monotherapy

【High density lipoprotein-cholesterol (mmol/l)】

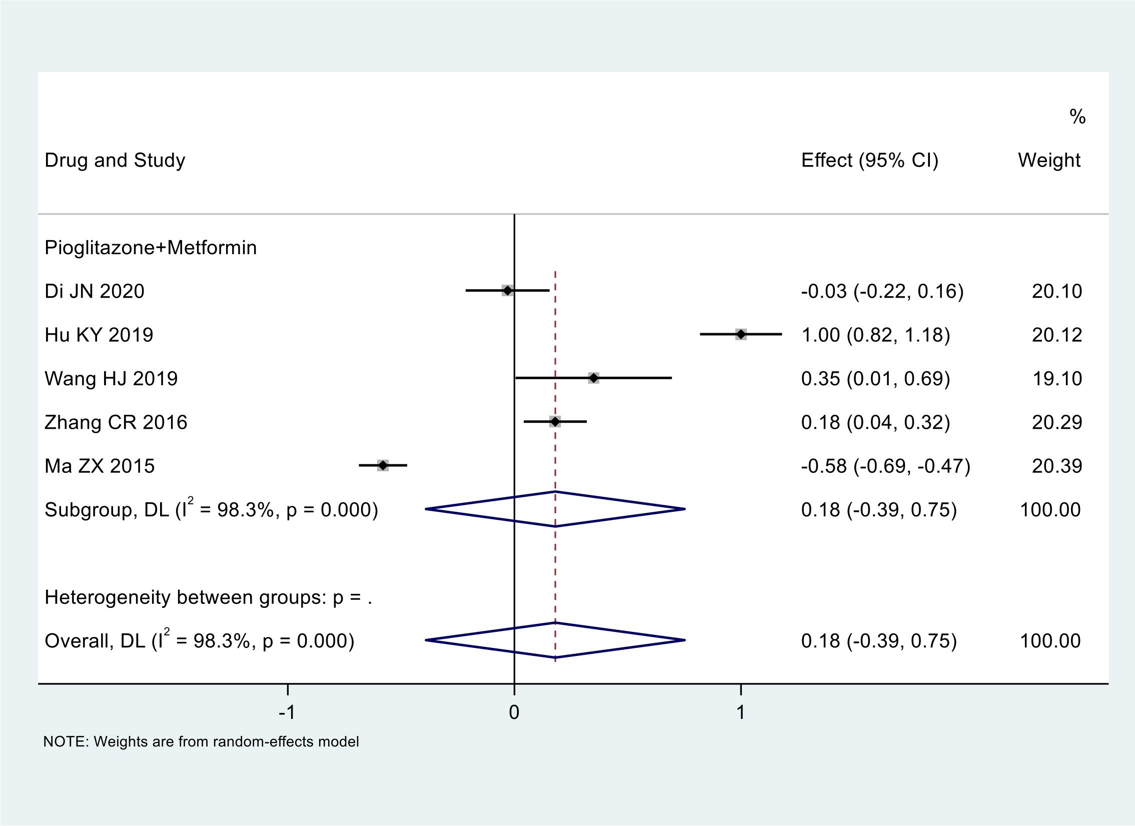

Figure S39. Meta-analysis results for change in high density lipoprotein-cholesterol (mmol/l) of thiazolidinediones added to metformin compared with metformin monotherapy

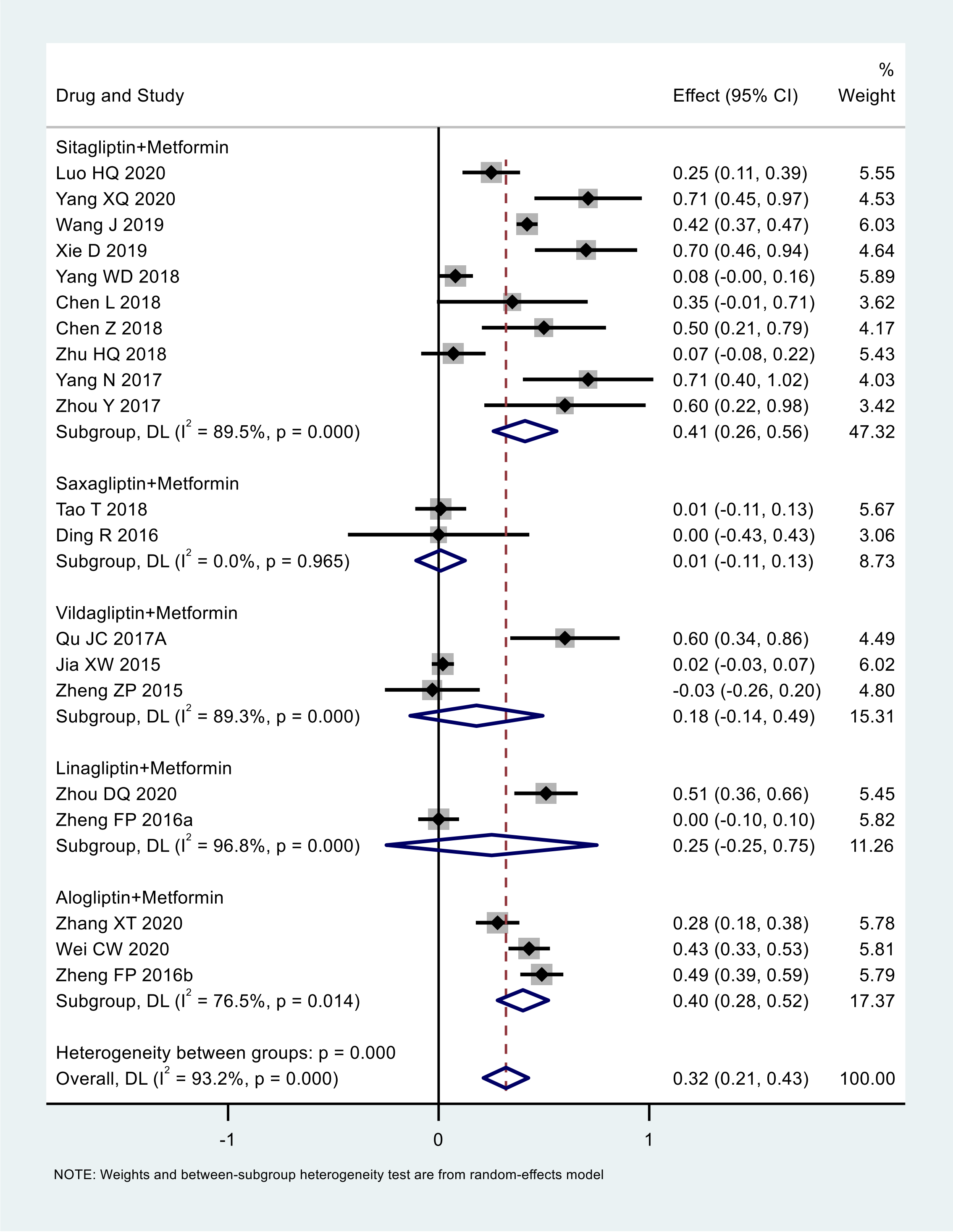

Figure S40. Meta-analysis results for change in high density lipoprotein-cholesterol (mmol/l) of dipeptidyl peptidase 4 inhibitors added to metformin compared with metformin monotherapy

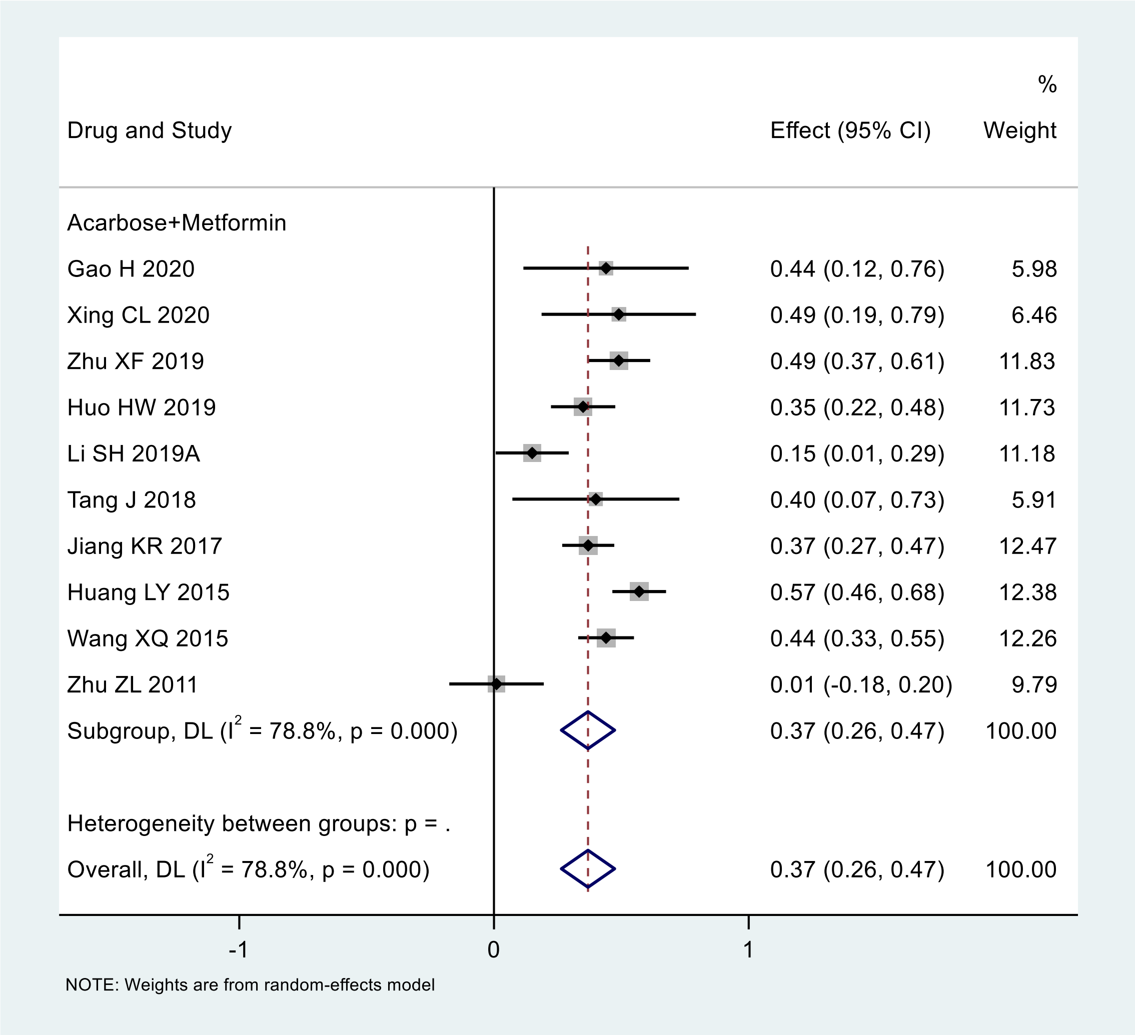

Figure S41. Meta-analysis results for change in high density lipoprotein-cholesterol (mmol/l) of α-glucosidase inhibitors added to metformin compared with metformin monotherapy

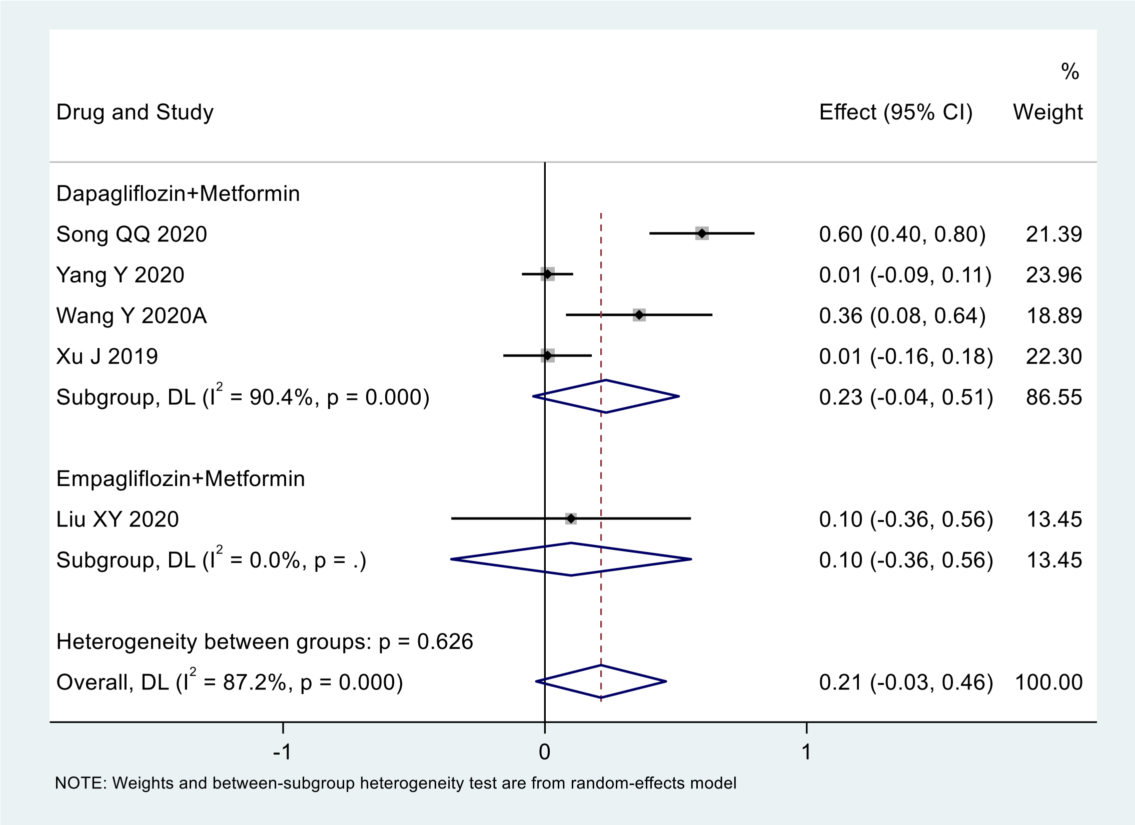

Figure S42. Meta-analysis results for change in high density lipoprotein-cholesterol (mmol/l) of sodium-glucose cotransporter 2 inhibitors added to metformin compared with metformin monotherapy

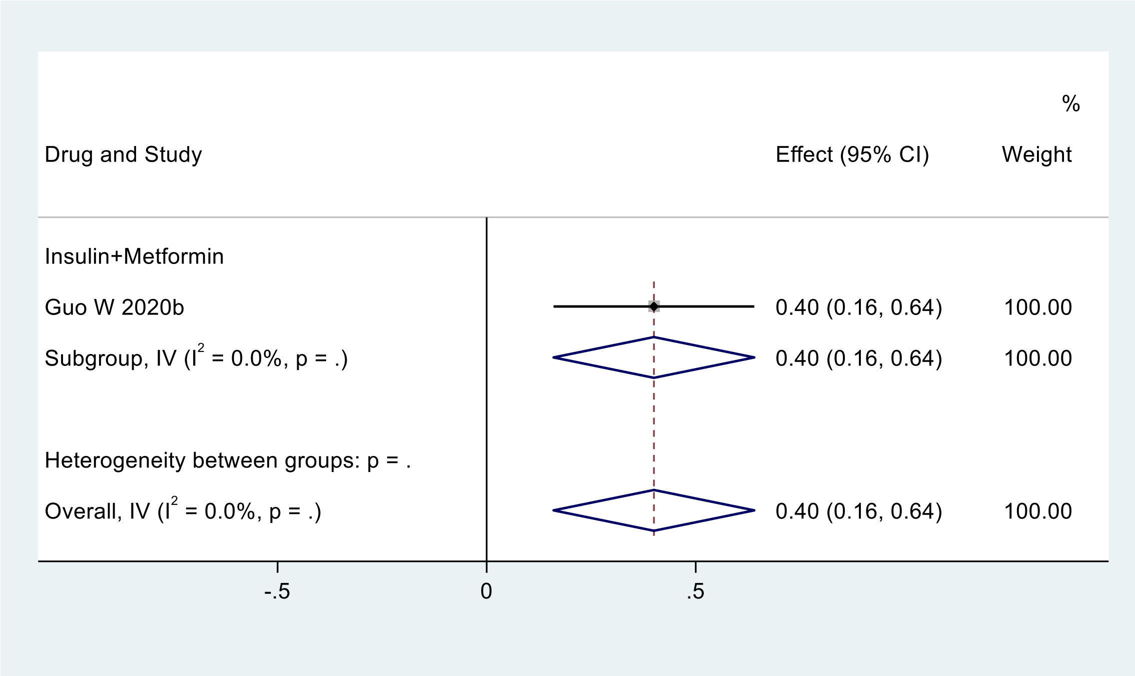

Figure S43. Meta-analysis results for change in high density lipoprotein-cholesterol (mmol/l) of insulins added to metformin compared with metformin monotherapy

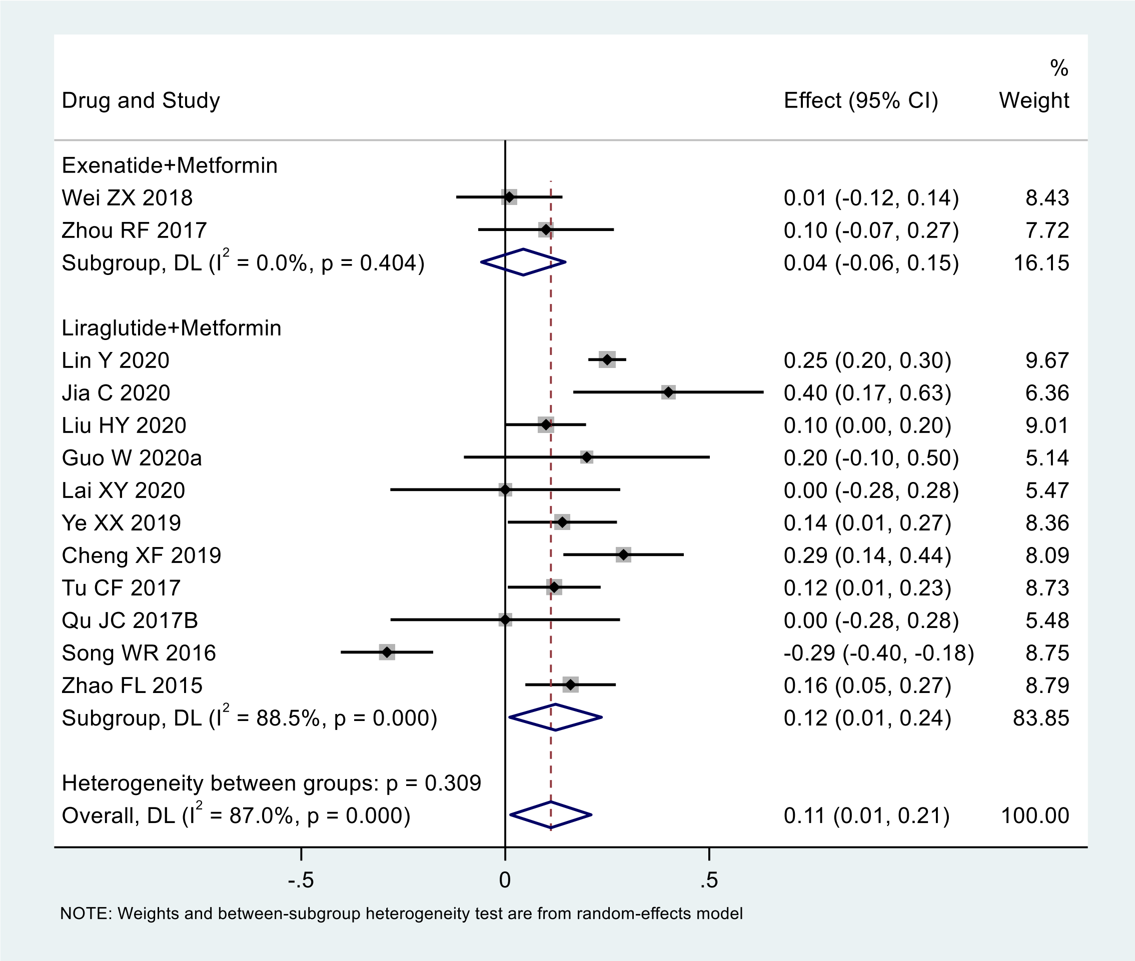

Figure S44. Meta-analysis results for change in high density lipoprotein-cholesterol (mmol/l) of glucagon-like peptide-1 receptor agonists added to metformin compared with metformin monotherapy

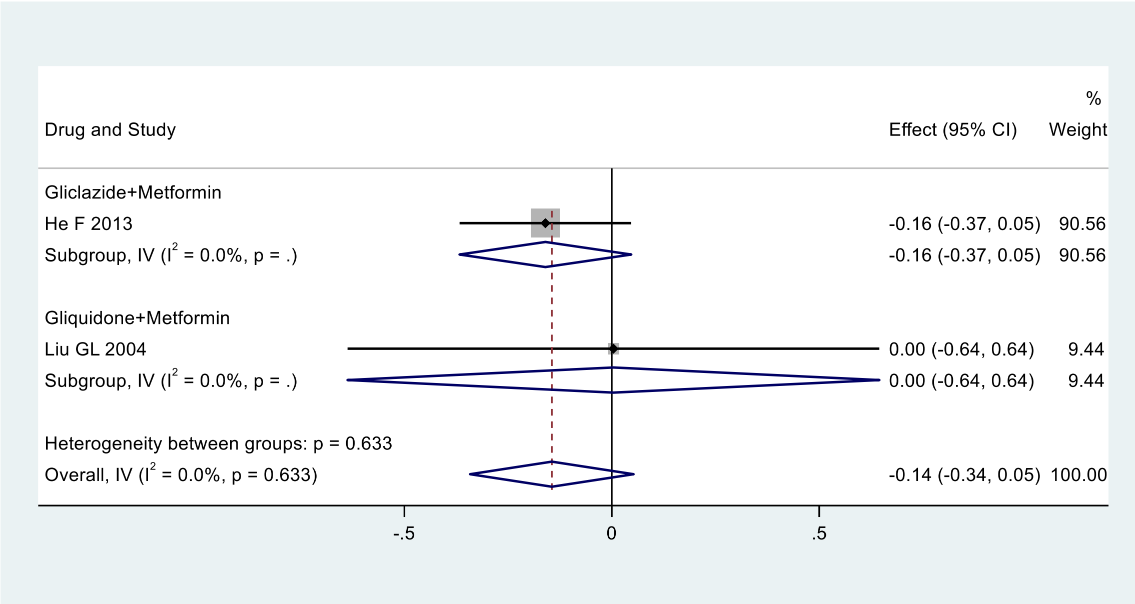

Figure S45. Meta-analysis results for change in high density lipoprotein-cholesterol (mmol/l) of sulfonylureas added to metformin compared with metformin monotherapy

【Systolic blood pressure (mmHg)】

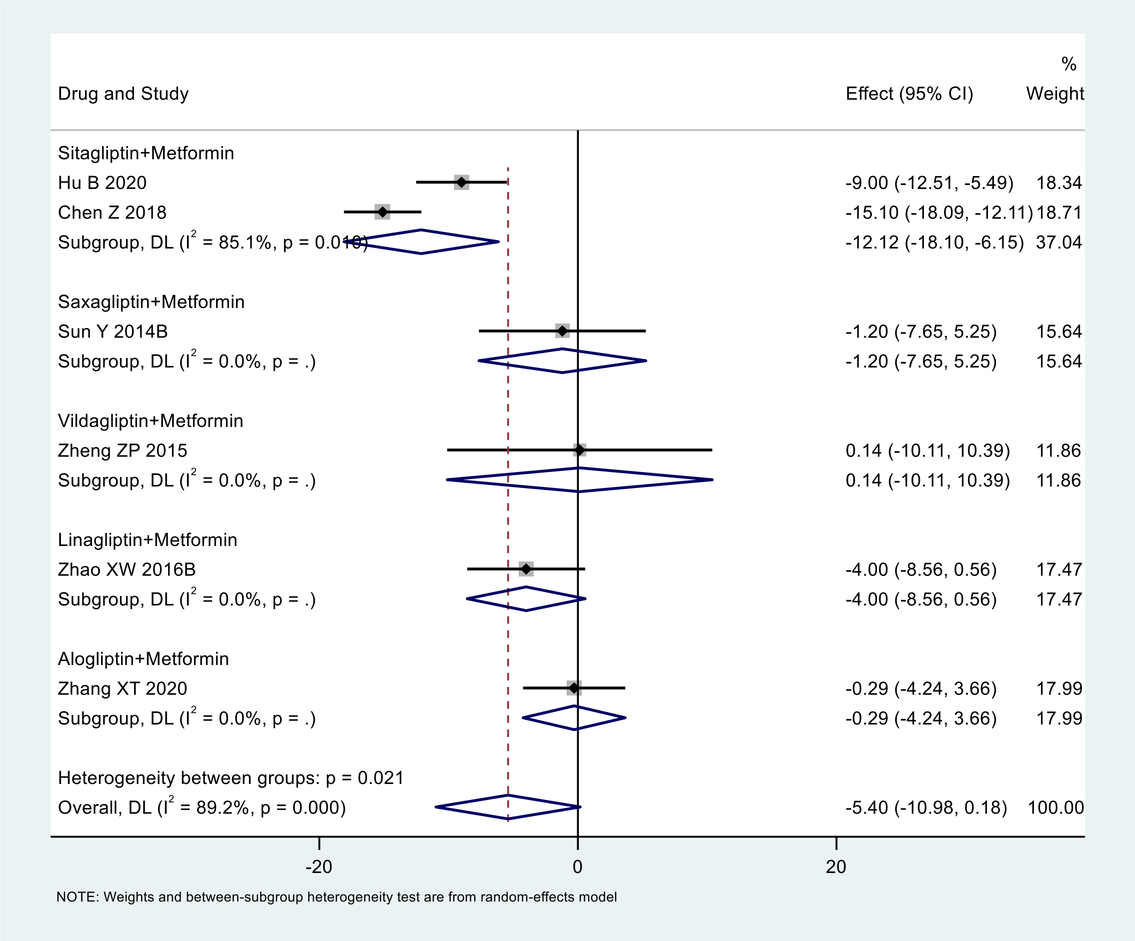

Figure S46. Meta-analysis results for change in systolic blood pressure (mmHg) of dipeptidyl peptidase 4 inhibitors added to metformin compared with metformin monotherapy

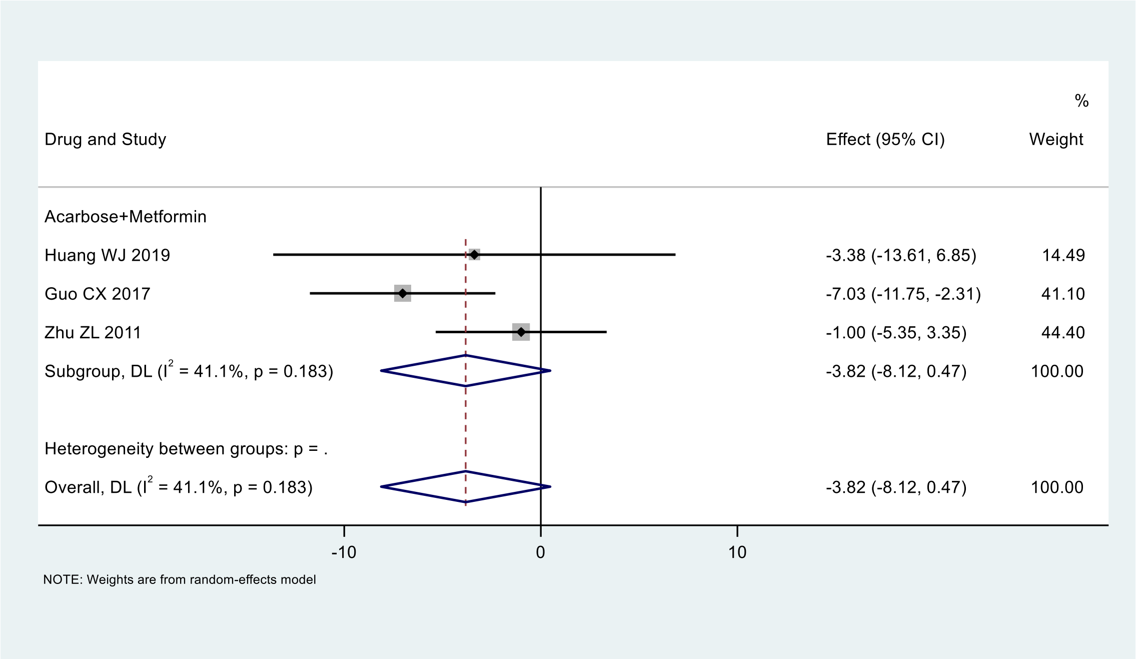

Figure S47. Meta-analysis results for change in systolic blood pressure (mmHg) of α-glucosidase inhibitors added to metformin compared with metformin monotherapy

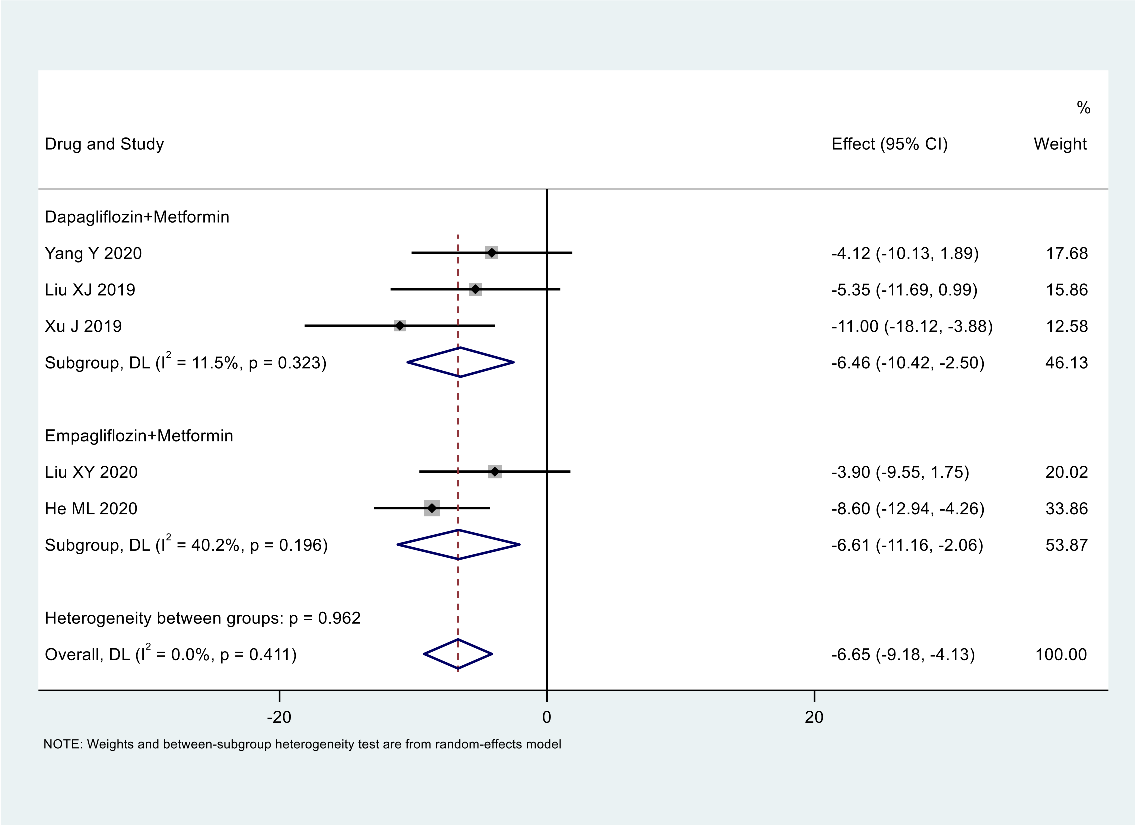

Figure S48. Meta-analysis results for change in systolic blood pressure (mmHg) of sodium-glucose cotransporter 2 inhibitors added to metformin compared with metformin monotherapy

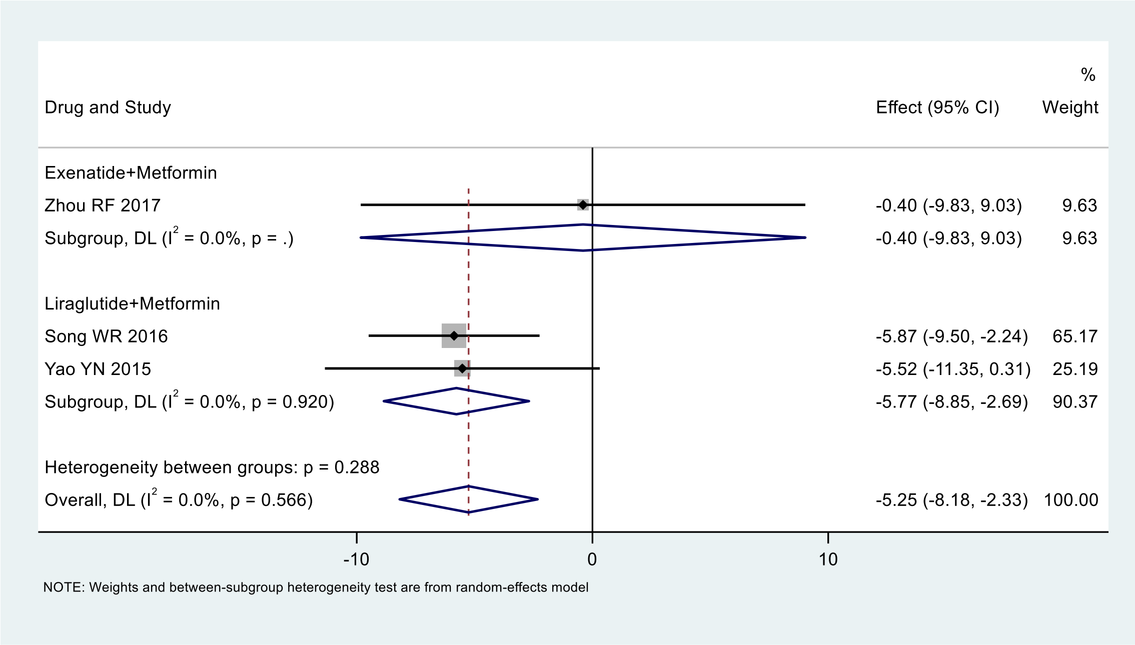

Figure S49. Meta-analysis results for change in systolic blood pressure (mmHg) of glucagon-like peptide-1 receptor agonists added to metformin compared with metformin monotherapy

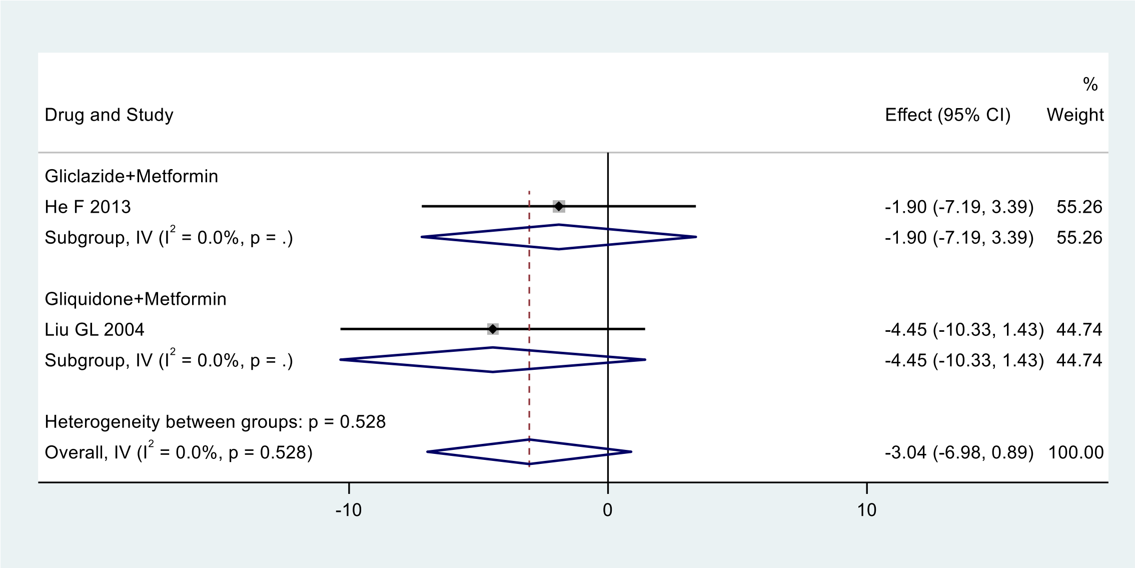

Figure S50. Meta-analysis results for change in systolic blood pressure (mmHg) of sulfonylureas added to metformin compared with metformin monotherapy

【Hypoglycemia】

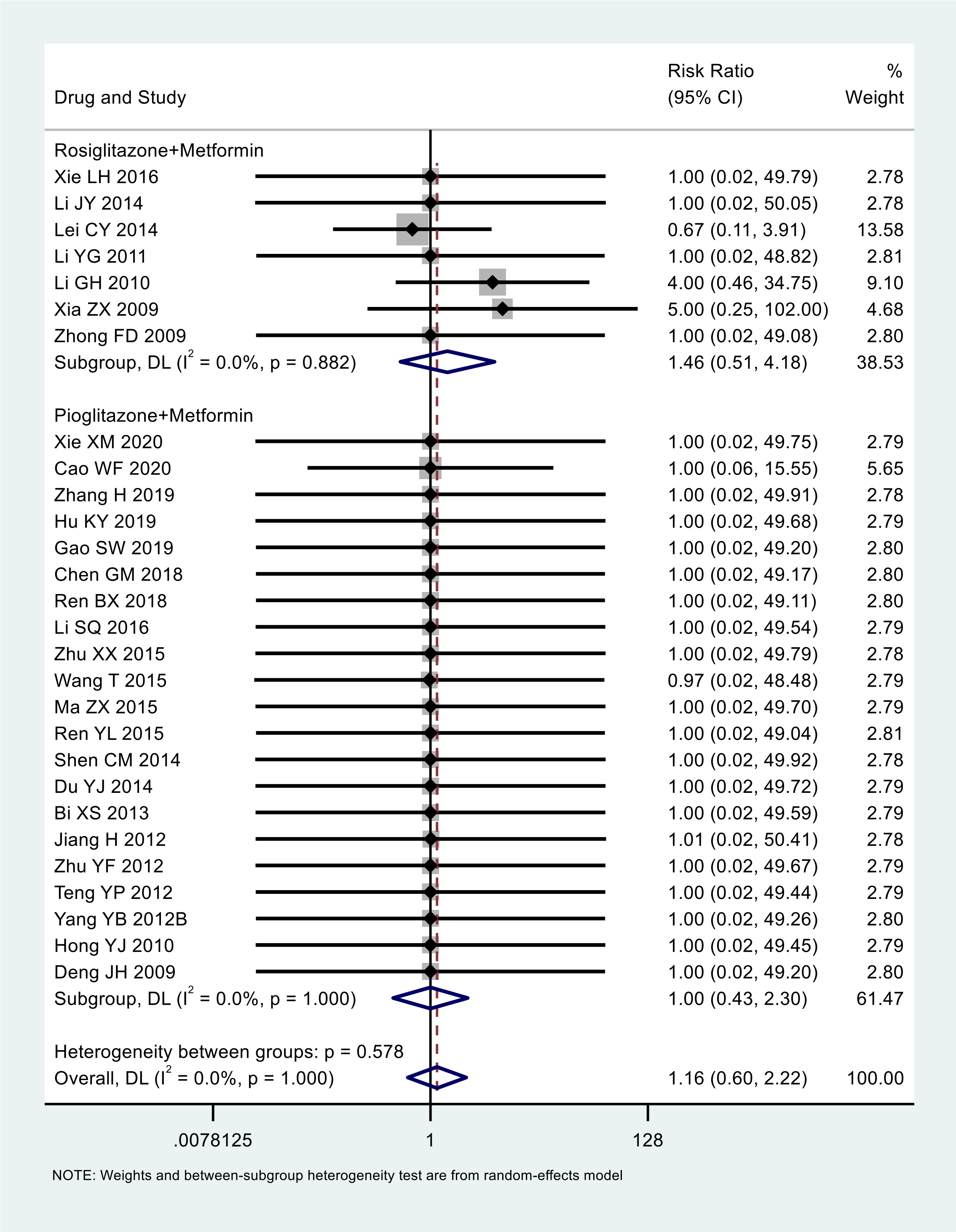

Figure S51. Meta-analysis results for incidence of hypoglycemia of thiazolidinediones added to metformin compared with metformin monotherapy

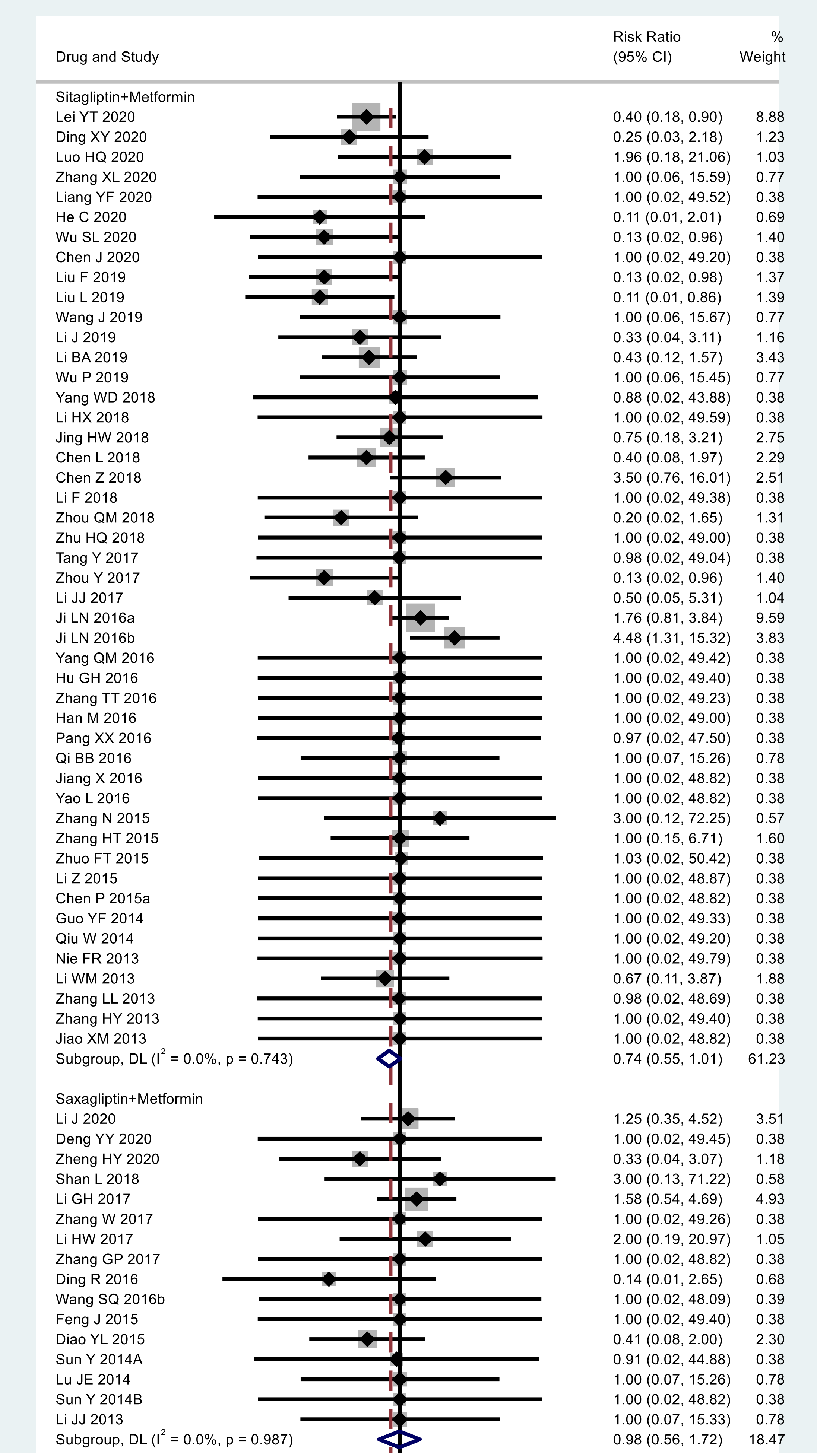
Drug and Study
Risk Ratio (95% CI)
% Weight
Sitagliptin+Metformin
Lei YT 2020 0.40 (0.18, 0.90) 8.88
Ding XY 2020 0.25 (0.03, 2.18) 1.23
Luo HQ 2020 1.96 (0.18, 21.06) 1.03
Zhang XL 2020 1.00 (0.06, 15.59) 0.77
Liang YF 2020 1.00 (0.02, 49.52) 0.38
He C 2020 0.11 (0.01, 2.01) 0.69
Wu SL 2020 0.13 (0.02, 0.96) 1.40
Chen J 2020 1.00 (0.02, 49.20) 0.38
Liu F 2019 0.13 (0.02, 0.98) 1.37
Liu L 2019 0.11 (0.01, 0.86) 1.39
Wang J 2019 1.00 (0.06, 15.67) 0.77
Li J 2019 0.33 (0.04, 3.11) 1.16
Li BA 2019 0.43 (0.12, 1.57) 3.43
Wu P 2019 1.00 (0.06, 15.45) 0.77
Yang WD 2018 0.88 (0.02, 43.88) 0.38
Li HX 2018 1.00 (0.02, 49.59) 0.38
Jing HW 2018 0.75 (0.18, 3.21) 2.75
Chen L 2018 0.40 (0.08, 1.97) 2.29
Chen Z 2018 3.50 (0.76, 16.01) 2.51
Li F 2018 1.00 (0.02, 49.38) 0.38
Zhou QM 2018 0.20 (0.02, 1.65) 1.31
Zhu HQ 2018 1.00 (0.02, 49.00) 0.38
Tang Y 2017 0.98 (0.02, 49.04) 0.38
Zhou Y 2017 0.13 (0.02, 0.96) 1.40
Li JJ 2017 0.50 (0.05, 5.31) 1.04
Ji LN 2016a 1.76 (0.81, 3.84) 9.59
Ji LN 2016b 4.48 (1.31, 15.32) 3.83
Yang QM 2016 1.00 (0.02, 49.42) 0.38
Hu GH 2016 1.00 (0.02, 49.40) 0.38
Zhang TT 2016 1.00 (0.02, 49.23) 0.38
Han M 2016 1.00 (0.02, 49.00) 0.38
Pang XX 2016 0.97 (0.02, 47.50) 0.38
Qi BB 2016 1.00 (0.07, 15.26) 0.78
Jiang X 2016 1.00 (0.02, 48.82) 0.38
Yao L 2016 1.00 (0.02, 48.82) 0.38
Zhang N 2015 3.00 (0.12, 72.25) 0.57
Zhang HT 2015 1.00 (0.15, 6.71) 1.60
Zhuo FT 2015 1.03 (0.02, 50.42) 0.38
Li Z 2015 1.00 (0.02, 48.87) 0.38
Chen P 2015a 1.00 (0.02, 48.82) 0.38
Guo YF 2014 1.00 (0.02, 49.33) 0.38
Qiu W 2014 1.00 (0.02, 49.20) 0.38
Nie FR 2013 1.00 (0.02, 49.79) 0.38
Li WM 2013 0.67 (0.11, 3.87) 1.88
Zhang LL 2013 0.98 (0.02, 48.69) 0.38
Zhang HY 2013 1.00 (0.02, 49.40) 0.38
Jiao XM 2013 1.00 (0.02, 48.82) 0.38
Subgroup, DL (I^2 = 0.0%, p = 0.743) 0.74 (0.55, 1.01) 61.23
Saxagliptin+Metformin
Li J 2020 1.25 (0.35, 4.52) 3.51
Deng YY 2020 1.00 (0.02, 49.45) 0.38
Zheng HY 2020 0.33 (0.04, 3.07) 1.18
Shan L 2018 3.00 (0.13, 71.22) 0.58
Li GH 2017 1.58 (0.54, 4.69) 4.93
Zhang W 2017 1.00 (0.02, 49.26) 0.38
Li HW 2017 2.00 (0.19, 20.97) 1.05
Zhang GP 2017 1.00 (0.02, 48.82) 0.38
Ding R 2016 0.14 (0.01, 2.65) 0.68
Wang SQ 2016b 1.00 (0.02, 48.09) 0.39
Feng J 2015 1.00 (0.02, 49.40) 0.38
Diao YL 2015 0.41 (0.08, 2.00) 2.30
Sun Y 2014A 0.91 (0.02, 44.88) 0.38
Lu JE 2014 1.00 (0.07, 15.26) 0.78
Sun Y 2014B 1.00 (0.02, 48.82) 0.38
Li JJ 2013 1.00 (0.07, 15.33) 0.78
Subgroup, DL (I^2 = 0.0%, p = 0.987) 0.98 (0.56, 1.72) 18.47

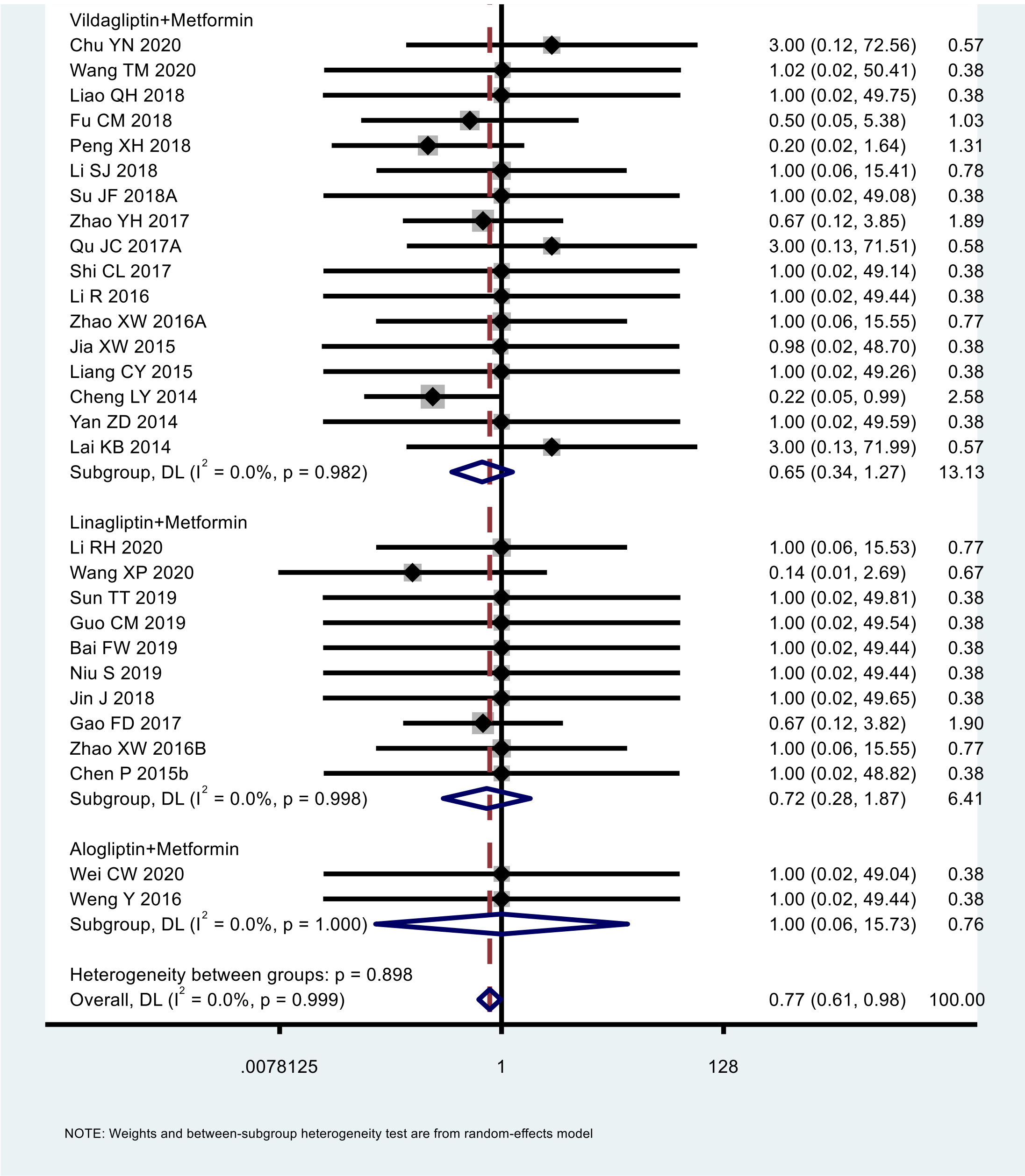

Figure S52. Meta-analysis results for incidence of hypoglycemia of dipeptidyl peptidase 4 inhibitors added to metformin compared with metformin monotherapy

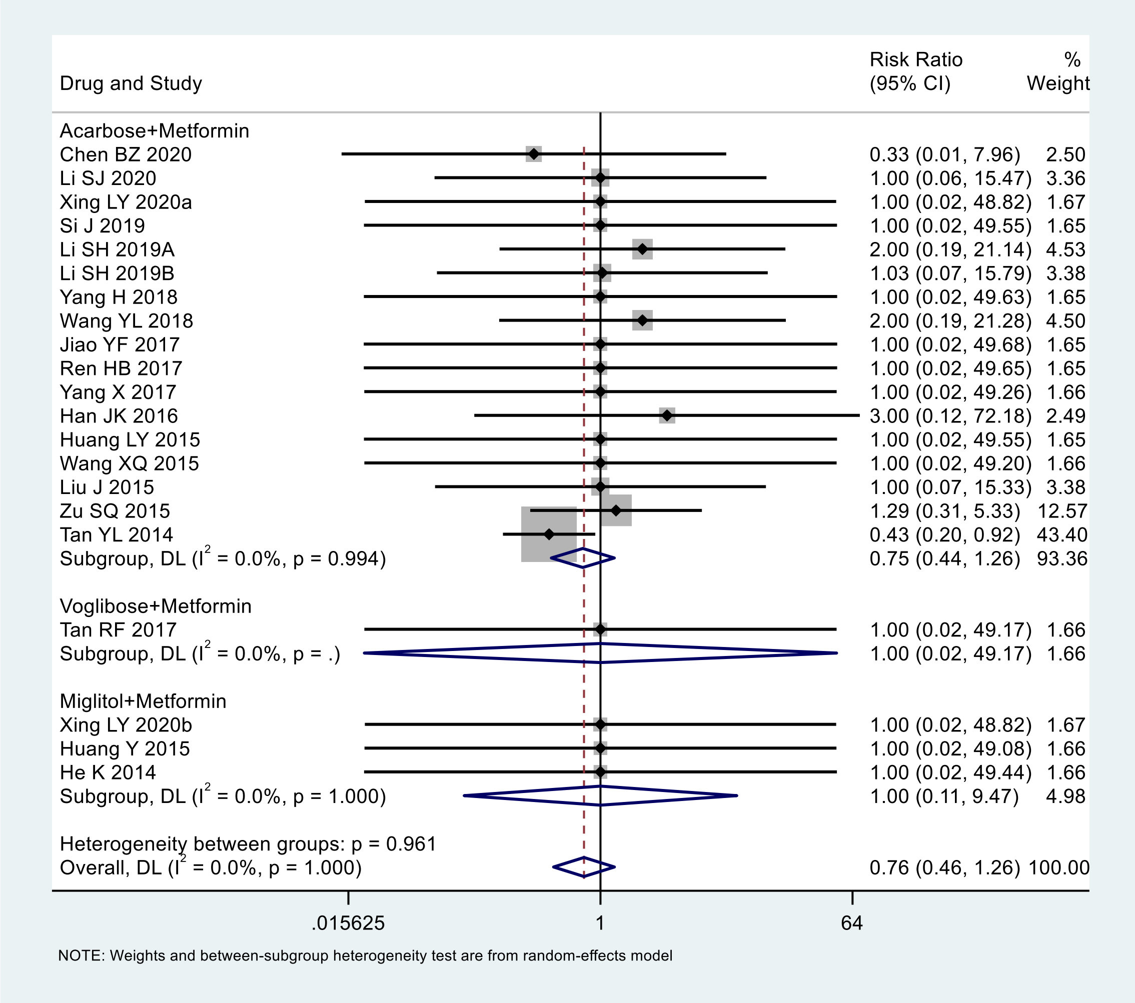

Figure S53. Meta-analysis results for incidence of hypoglycemia of α-glucosidase inhibitors added to metformin compared with metformin monotherapy

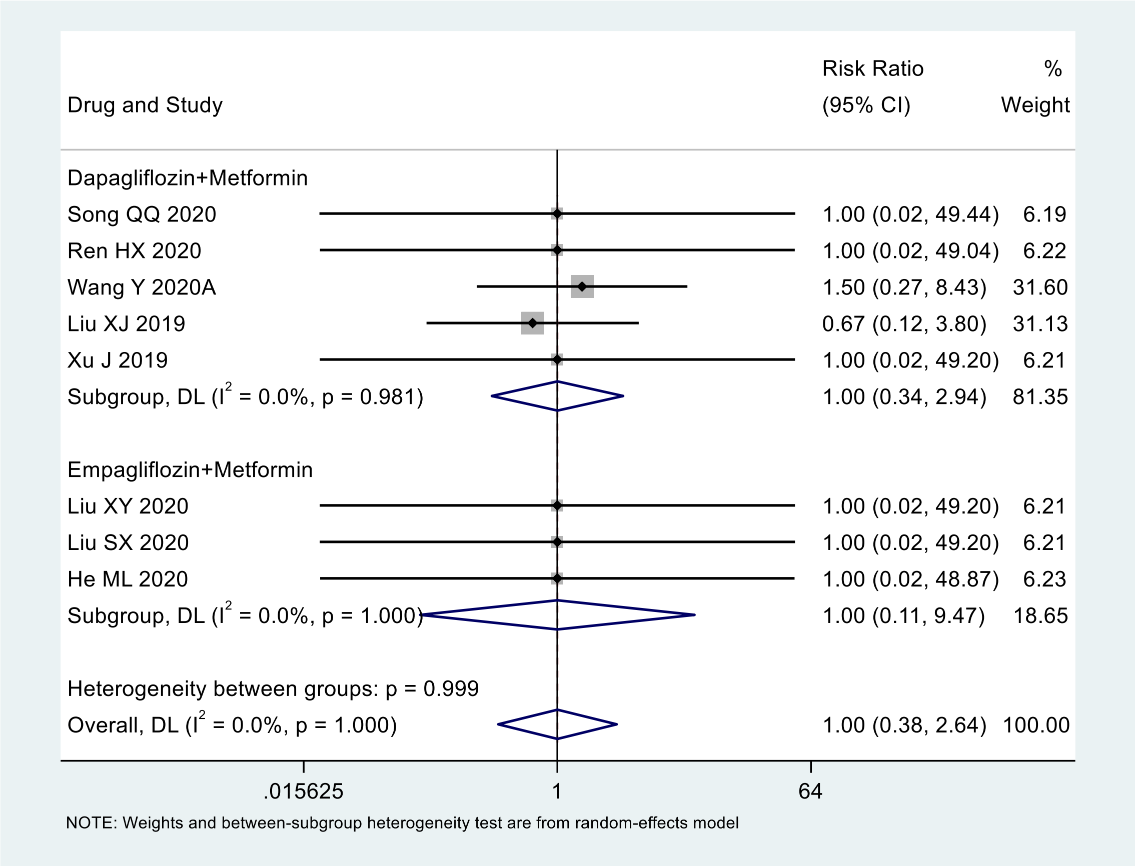

Figure S54. Meta-analysis results for incidence of hypoglycemia of sodium-glucose cotransporter 2 inhibitors added to metformin compared with metformin monotherapy

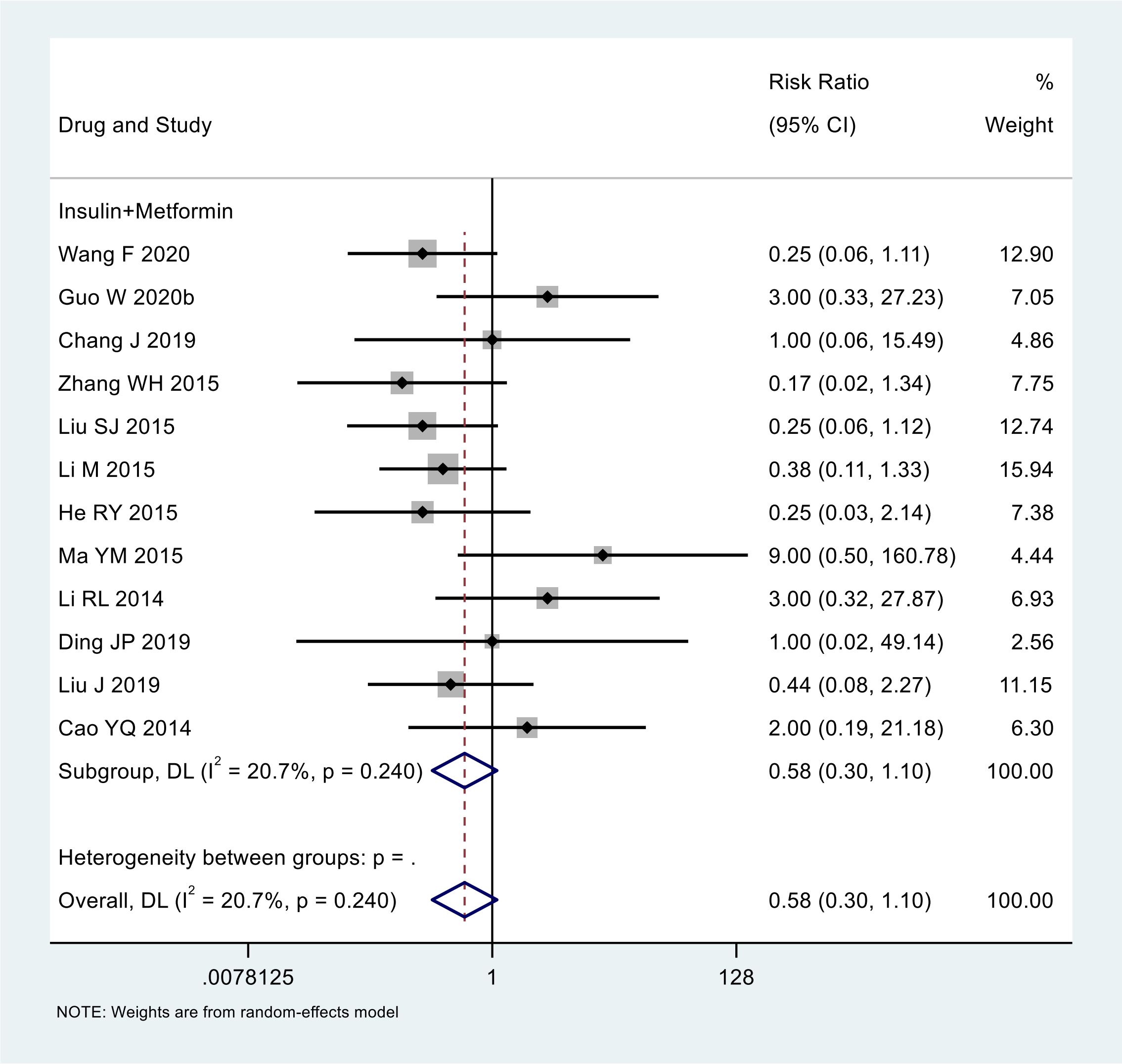

Figure S55. Meta-analysis results for incidence of hypoglycemia of insulins added to metformin compared with metformin monotherapy

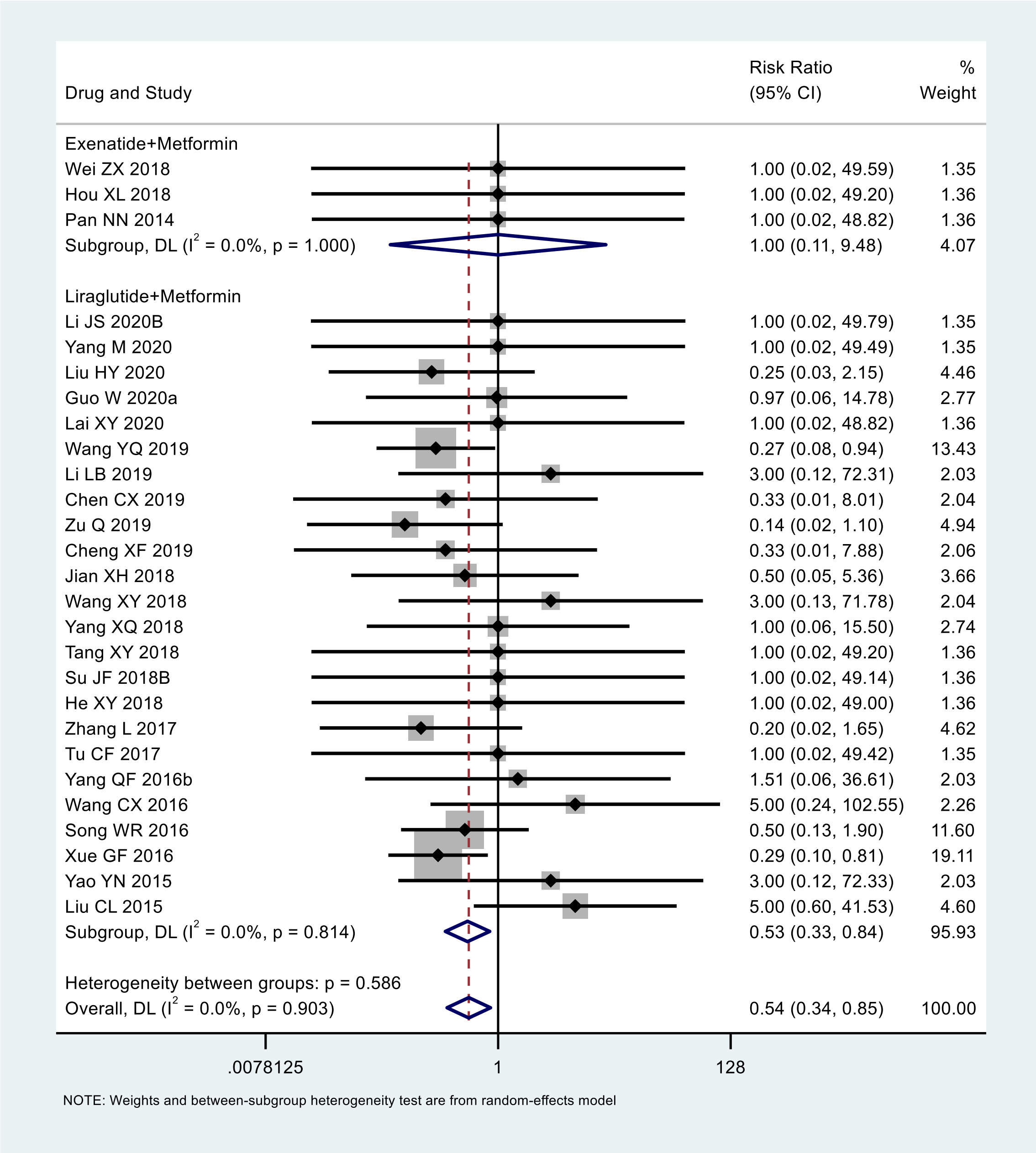

Figure S56. Meta-analysis results for incidence of hypoglycemia of glucagon-like peptide-1 receptor agonists added to metformin compared with metformin monotherapy

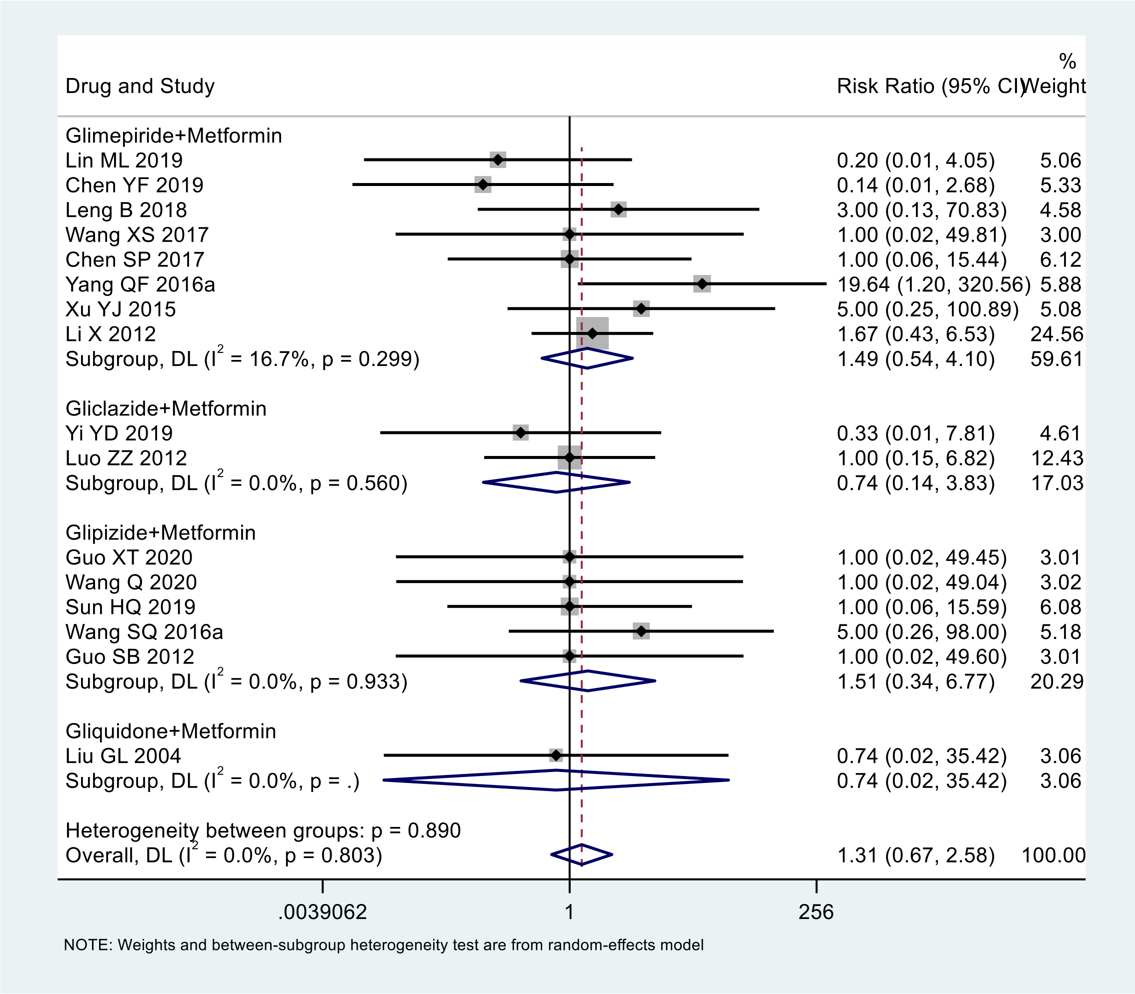

Figure S57. Meta-analysis results for incidence of hypoglycemia of sulfonylureas added to metformin compared with metformin monotherapy

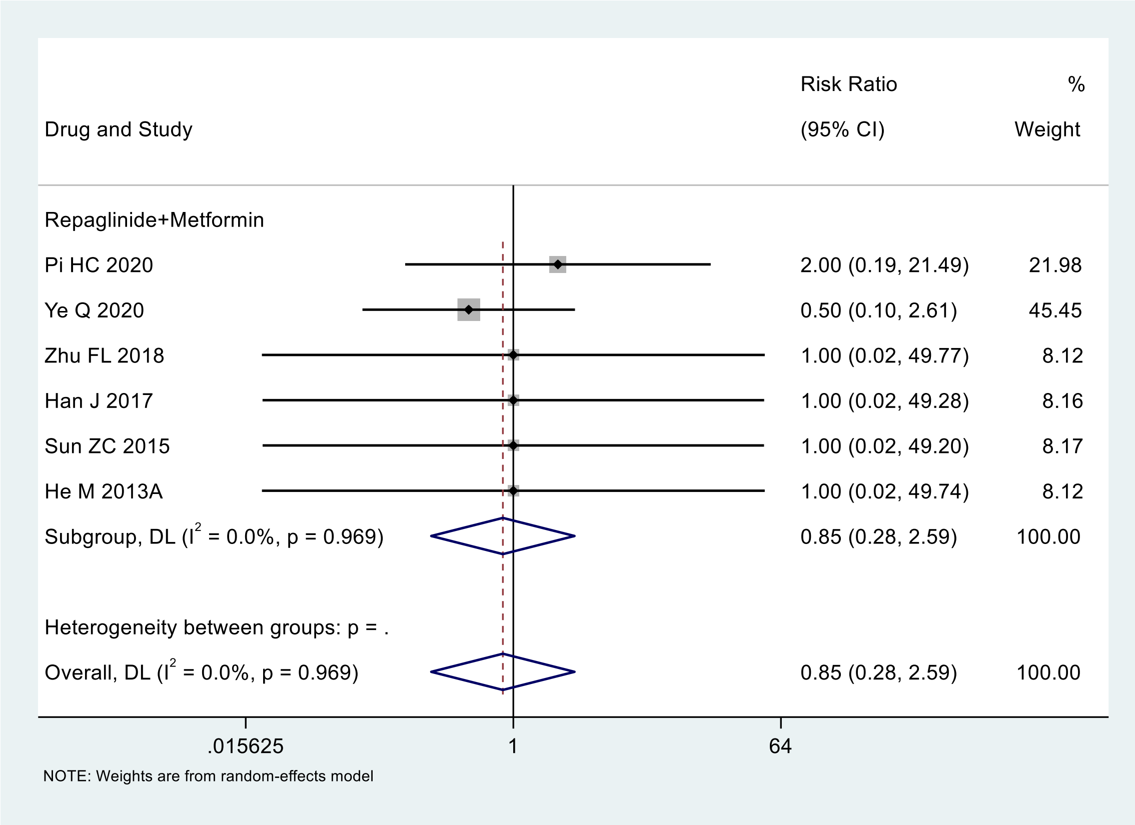

Figure S58. Meta-analysis results for incidence of hypoglycemia of glinides added to metformin compared with metformin monotherapy

【Mortality and vascular outcomes】

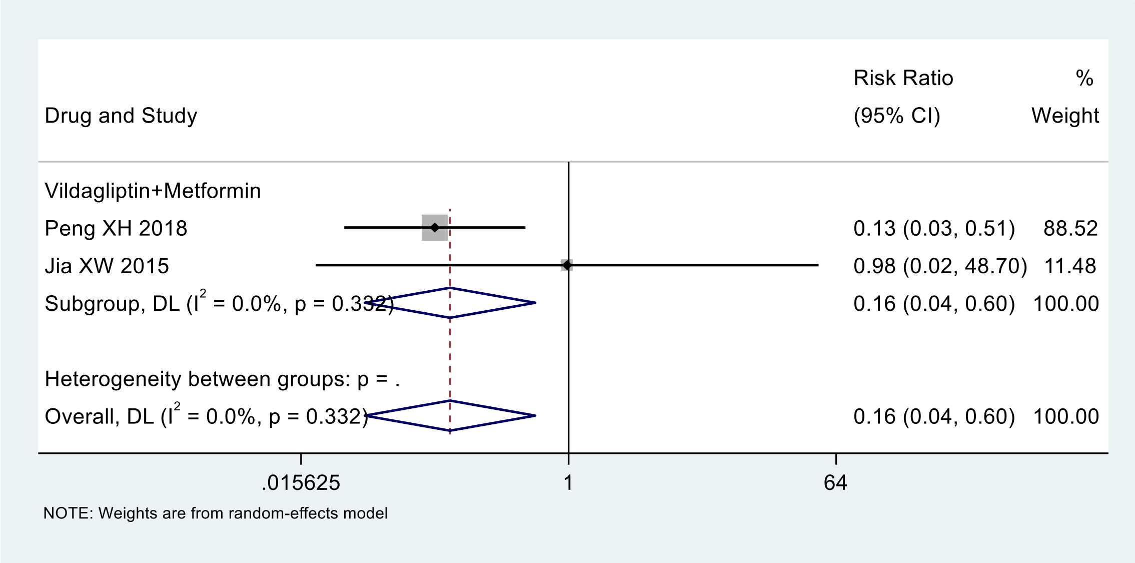

Figure S59. Meta-analysis results for incidence of complications of vildagliptin added to metformin compared with metformin monotherapy

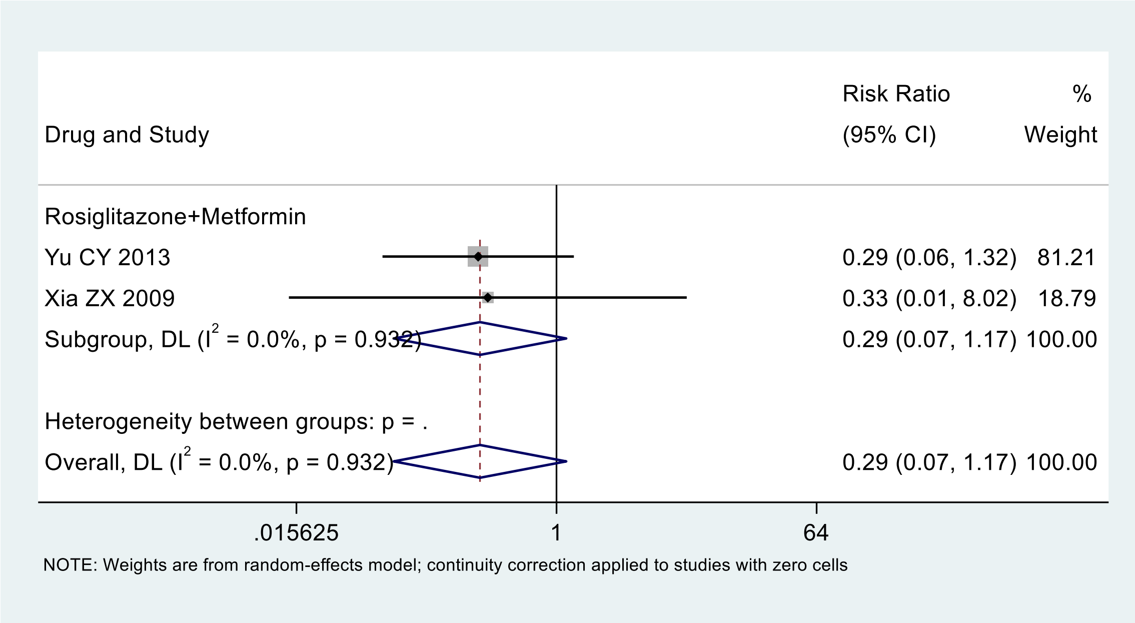

Figure S60. Meta-analysis results for incidence of complications of rosiglitazone added to metformin compared with metformin monotherapy

【Sensitivity analysis】

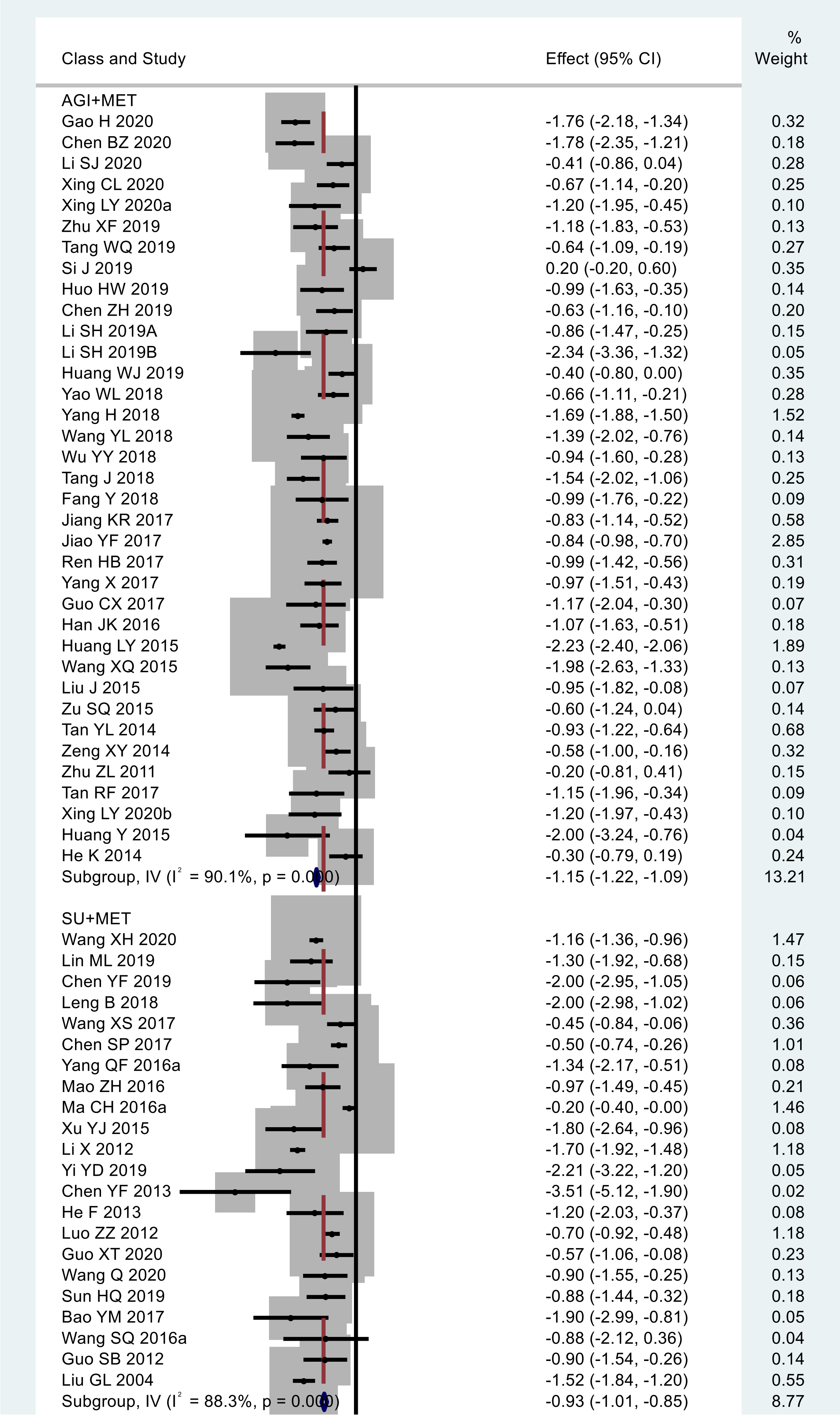

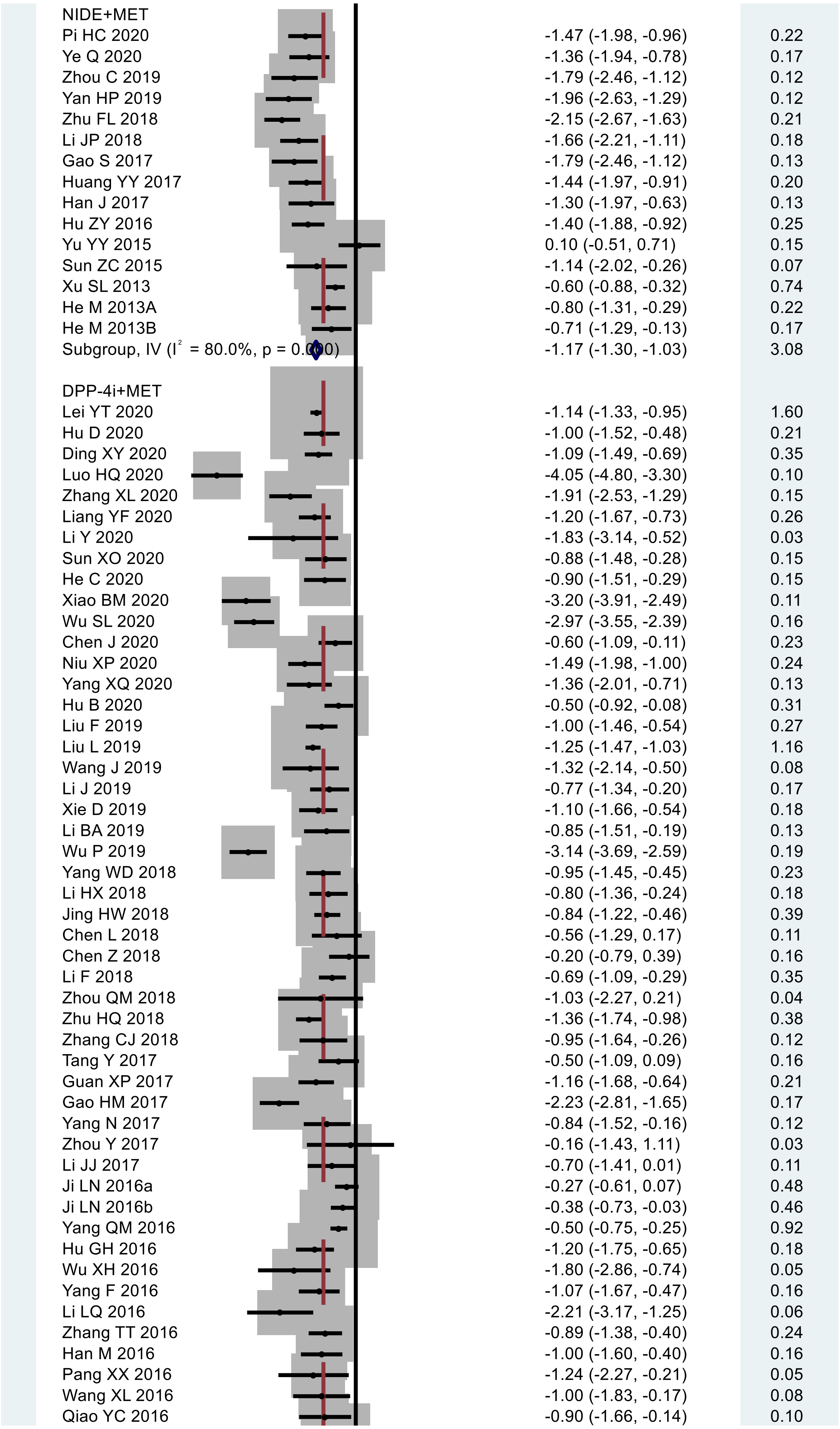
NIDE+MET
Pi HC 2020 -1.47 (-1.98, -0.96) 0.22
Ye Q 2020 -1.36 (-1.94, -0.78) 0.17
Zhou C 2019 -1.79 (-2.46, -1.12) 0.12
Yan HP 2019 -1.96 (-2.63, -1.29) 0.12
Zhu FL 2018 -2.15 (-2.67, -1.63) 0.21
Li JP 2018 -1.66 (-2.21, -1.11) 0.18
Gao S 2017 -1.79 (-2.46, -1.12) 0.13
Huang YY 2017 -1.44 (-1.97, -0.91) 0.20
Han J 2017 -1.30 (-1.97, -0.63) 0.13
Hu ZY 2016 -1.40 (-1.88, -0.92) 0.25
Yu YY 2015 0.10 (-0.51, 0.71) 0.15
Sun ZC 2015 -1.14 (-2.02, -0.26) 0.07
Xu SL 2013 -0.60 (-0.88, -0.32) 0.74
He M 2013A -0.80 (-1.31, -0.29) 0.22
He M 2013B -0.71 (-1.29, -0.13) 0.17
Subgroup, IV (I² = 80.0%, p = 0.000) -1.17 (-1.30, -1.03) 3.08
DPP-4i+MET
Lei YT 2020 -1.14 (-1.33, -0.95) 1.60
Hu D 2020 -1.00 (-1.52, -0.48) 0.21
Ding XY 2020 -1.09 (-1.49, -0.69) 0.35
Luo HQ 2020 -4.05 (-4.80, -3.30) 0.10
Zhang XL 2020 -1.91 (-2.53, -1.29) 0.15
Liang YF 2020 -1.20 (-1.67, -0.73) 0.26
Li Y 2020 -1.83 (-3.14, -0.52) 0.03
Sun XO 2020 -0.88 (-1.48, -0.28) 0.15
He C 2020 -0.90 (-1.51, -0.29) 0.15
Xiao BM 2020 -3.20 (-3.91, -2.49) 0.11
Wu SL 2020 -2.97 (-3.55, -2.39) 0.16
Chen J 2020 -0.60 (-1.09, -0.11) 0.23
Niu XP 2020 -1.49 (-1.98, -1.00) 0.24
Yang XQ 2020 -1.36 (-2.01, -0.71) 0.13
Hu B 2020 -0.50 (-0.92, -0.08) 0.31
Liu F 2019 -1.00 (-1.46, -0.54) 0.27
Liu L 2019 -1.25 (-1.47, -1.03) 1.16
Wang J 2019 -1.32 (-2.14, -0.50) 0.08
Li J 2019 -0.77 (-1.34, -0.20) 0.17
Xie D 2019 -1.10 (-1.66, -0.54) 0.18
Li BA 2019 -0.85 (-1.51, -0.19) 0.13
Wu P 2019 -3.14 (-3.69, -2.59) 0.19
Yang WD 2018 -0.95 (-1.45, -0.45) 0.23
Li HX 2018 -0.80 (-1.36, -0.24) 0.18
Jing HW 2018 -0.84 (-1.22, -0.46) 0.39
Chen L 2018 -0.56 (-1.29, 0.17) 0.11
Chen Z 2018 -0.20 (-0.79, 0.39) 0.16
Li F 2018 -0.69 (-1.09, -0.29) 0.35
Zhou QM 2018 -1.03 (-2.27, 0.21) 0.04
Zhu HQ 2018 -1.36 (-1.74, -0.98) 0.38
Zhang CJ 2018 -0.95 (-1.64, -0.26) 0.12
Tang Y 2017 -0.50 (-1.09, 0.09) 0.16
Guan XP 2017 -1.16 (-1.68, -0.64) 0.21
Gao HM 2017 -2.23 (-2.81, -1.65) 0.17
Yang N 2017 -0.84 (-1.52, -0.16) 0.12
Zhou Y 2017 -0.16 (-1.43, 1.11) 0.03
Li JJ 2017 -0.70 (-1.41, 0.01) 0.11
Ji LN 2016a -0.27 (-0.61, 0.07) 0.48
Ji LN 2016b -0.38 (-0.73, -0.03) 0.46
Yang QM 2016 -0.50 (-0.75, -0.25) 0.92
Hu GH 2016 -1.20 (-1.75, -0.65) 0.18
Wu XH 2016 -1.80 (-2.86, -0.74) 0.05
Yang F 2016 -1.07 (-1.67, -0.47) 0.16
Li LQ 2016 -2.21 (-3.17, -1.25) 0.06
Zhang TT 2016 -0.89 (-1.38, -0.40) 0.24
Han M 2016 -1.00 (-1.60, -0.40) 0.16
Pang XX 2016 -1.24 (-2.27, -0.21) 0.05
Wang XL 2016 -1.00 (-1.83, -0.17) 0.08
Qiao YC 2016 -0.90 (-1.66, -0.14) 0.10

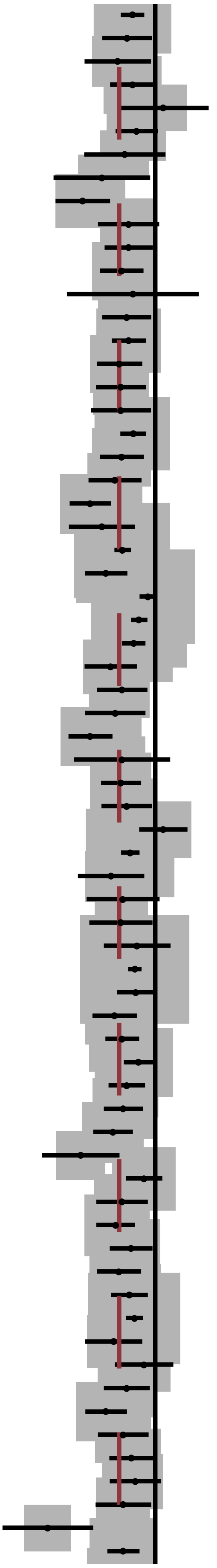

Qi BB 2016	-0.60 (-0.91, -0.29)	0.59
Jiang X 2016	-0.74 (-1.39, -0.09)	0.13
Yao L 2016	-0.99 (-1.85, -0.13)	0.08
Zhang N 2015	-0.60 (-1.19, -0.01)	0.16
Yuan XJ 2015	0.20 (-0.99, 1.39)	0.04
Zhang HT 2015	-0.49 (-1.05, 0.07)	0.18
Zhuo FT 2015	-0.80 (-1.86, 0.26)	0.05
Fan SH 2015	-1.40 (-2.66, -0.14)	0.04
Li Z 2015	-1.90 (-2.61, -1.19)	0.11
Chen P 2015a	-0.70 (-1.50, 0.10)	0.09
Guo YF 2014	-0.70 (-1.33, -0.07)	0.14
Zhang XF 2014	-0.88 (-1.45, -0.31)	0.17
Qiu W 2014	-0.59 (-2.31, 1.13)	0.02
Nie FR 2013	-0.75 (-1.39, -0.11)	0.14
Li WM 2013	-0.70 (-1.15, -0.25)	0.28
Zhang LL 2013	-0.94 (-1.53, -0.35)	0.16
Zhang HY 2013	-0.90 (-1.55, -0.25)	0.13
Jiao XM 2013	-0.90 (-1.68, -0.12)	0.09
Li J 2020	-0.58 (-0.92, -0.24)	0.49
Deng YY 2020	-0.88 (-1.45, -0.31)	0.17
Zheng HY 2020	-1.06 (-1.75, -0.37)	0.12
Zhao PY 2019	-1.70 (-2.24, -1.16)	0.19
Mei YJ 2018	-1.40 (-2.26, -0.54)	0.08
Shi FM 2018	-0.86 (-1.07, -0.65)	1.24
Shan L 2018	-1.29 (-1.84, -0.74)	0.19
Tao T 2018	-0.20 (-0.41, 0.01)	1.23
Li GH 2017	-0.43 (-0.64, -0.22)	1.26
Dong H 2017	-0.57 (-0.88, -0.26)	0.59
Zhang W 2017	-1.17 (-1.85, -0.49)	0.12
Li HW 2017	-0.87 (-1.52, -0.22)	0.13
Zhang GP 2017	-1.05 (-1.84, -0.26)	0.09
Ding R 2016	-1.70 (-2.27, -1.13)	0.17
Wang SQ 2016b	-0.87 (-2.12, 0.38)	0.04
Chen QL 2015	-0.90 (-1.42, -0.38)	0.21
Liang M 2015	-0.75 (-1.41, -0.09)	0.13
Feng J 2015	0.20 (-0.43, 0.83)	0.14
Diao YL 2015	-0.66 (-0.90, -0.42)	0.95
Wan J 2015	-1.16 (-2.03, -0.29)	0.07
Sun Y 2014A	-0.85 (-1.80, 0.10)	0.06
Lu JE 2014	-0.90 (-1.72, -0.08)	0.08
Sun Y 2014B	-0.48 (-1.35, 0.39)	0.07
Li JJ 2013	-0.54 (-0.71, -0.37)	1.89
Chu YN 2020	-0.51 (-1.00, -0.02)	0.23
Wang TM 2020	-1.07 (-1.65, -0.49)	0.17
Wang QL 2020	-0.87 (-1.31, -0.43)	0.29
Feng Y 2019	-0.44 (-0.83, -0.05)	0.37
Liao QH 2018	-0.75 (-1.22, -0.28)	0.25
Fu CM 2018	-0.84 (-1.35, -0.33)	0.21
Peng XH 2018	-1.11 (-1.63, -0.59)	0.21
Li SJ 2018	-1.95 (-2.96, -0.94)	0.06
Su JF 2018A	-0.30 (-0.77, 0.17)	0.25
Zhao YH 2017	-0.87 (-1.54, -0.20)	0.13
Qu JC 2017A	-1.04 (-1.54, -0.54)	0.22
Shi CL 2017	-0.64 (-1.20, -0.08)	0.18
Li R 2016	-0.95 (-1.53, -0.37)	0.17
Zhao XW 2016A	-0.68 (-1.16, -0.20)	0.24
Jia XW 2015	-0.55 (-0.78, -0.32)	1.09
Zheng ZP 2015	-1.09 (-1.84, -0.34)	0.10
Liang CY 2015	-0.30 (-1.06, 0.46)	0.10
Cheng LY 2014	-0.75 (-1.34, -0.16)	0.16
Chen XS 2014	-1.29 (-1.83, -0.75)	0.19
Yan ZD 2014	-0.84 (-1.50, -0.18)	0.13
Lai KB 2014	-0.63 (-1.20, -0.06)	0.17
Li RH 2020	-0.53 (-1.20, 0.14)	0.13
Wang XP 2020	-0.84 (-1.57, -0.11)	0.11
Zhou DQ 2020	-2.81 (-3.99, -1.63)	0.04
Sun TT 2019	-0.84 (-1.26, -0.42)	0.32

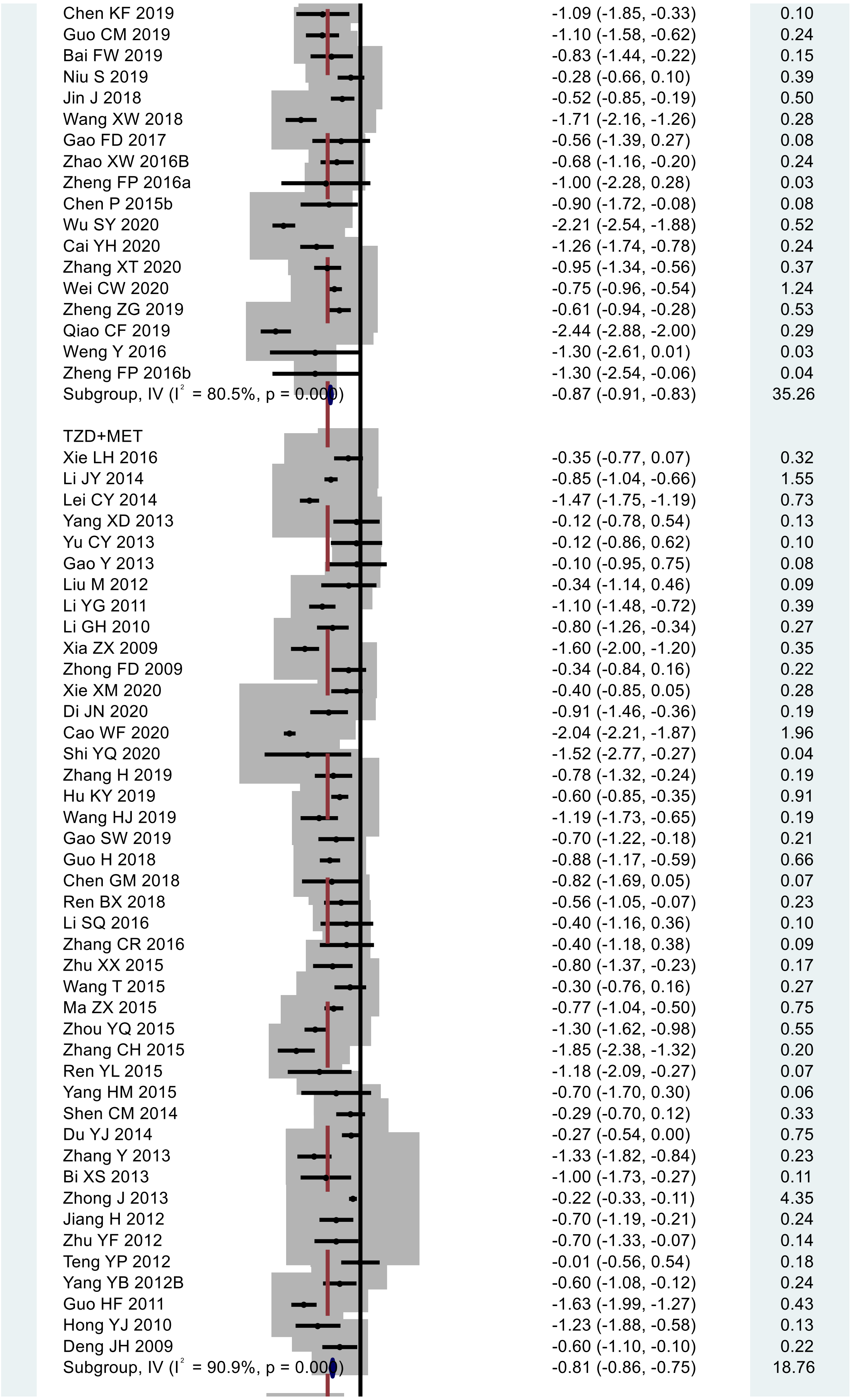
Chen KF 2019 -1.09 (-1.85, -0.33) 0.10
Guo CM 2019 -1.10 (-1.58, -0.62) 0.24
Bai FW 2019 -0.83 (-1.44, -0.22) 0.15
Niu S 2019 -0.28 (-0.66, 0.10) 0.39
Jin J 2018 -0.52 (-0.85, -0.19) 0.50
Wang XW 2018 -1.71 (-2.16, -1.26) 0.28
Gao FD 2017 -0.56 (-1.39, 0.27) 0.08
Zhao XW 2016B -0.68 (-1.16, -0.20) 0.24
Zheng FP 2016a -1.00 (-2.28, 0.28) 0.03
Chen P 2015b -0.90 (-1.72, -0.08) 0.08
Wu SY 2020 -2.21 (-2.54, -1.88) 0.52
Cai YH 2020 -1.26 (-1.74, -0.78) 0.24
Zhang XT 2020 -0.95 (-1.34, -0.56) 0.37
Wei CW 2020 -0.75 (-0.96, -0.54) 1.24
Zheng ZG 2019 -0.61 (-0.94, -0.28) 0.53
Qiao CF 2019 -2.44 (-2.88, -2.00) 0.29
Weng Y 2016 -1.30 (-2.61, 0.01) 0.03
Zheng FP 2016b -1.30 (-2.54, -0.06) 0.04
Subgroup, IV (I² = 80.5%, p = 0.000) -0.87 (-0.91, -0.83) 35.26
TZD+MET
Xie LH 2016 -0.35 (-0.77, 0.07) 0.32
Li JY 2014 -0.85 (-1.04, -0.66) 1.55
Lei CY 2014 -1.47 (-1.75, -1.19) 0.73
Yang XD 2013 -0.12 (-0.78, 0.54) 0.13
Yu CY 2013 -0.12 (-0.86, 0.62) 0.10
Gao Y 2013 -0.10 (-0.95, 0.75) 0.08
Liu M 2012 -0.34 (-1.14, 0.46) 0.09
Li YG 2011 -1.10 (-1.48, -0.72) 0.39
Li GH 2010 -0.80 (-1.26, -0.34) 0.27
Xia ZX 2009 -1.60 (-2.00, -1.20) 0.35
Zhong FD 2009 -0.34 (-0.84, 0.16) 0.22
Xie XM 2020 -0.40 (-0.85, 0.05) 0.28
Di JN 2020 -0.91 (-1.46, -0.36) 0.19
Cao WF 2020 -2.04 (-2.21, -1.87) 1.96
Shi YQ 2020 -1.52 (-2.77, -0.27) 0.04
Zhang H 2019 -0.78 (-1.32, -0.24) 0.19
Hu KY 2019 -0.60 (-0.85, -0.35) 0.91
Wang HJ 2019 -1.19 (-1.73, -0.65) 0.19
Gao SW 2019 -0.70 (-1.22, -0.18) 0.21
Guo H 2018 -0.88 (-1.17, -0.59) 0.66
Chen GM 2018 -0.82 (-1.69, 0.05) 0.07
Ren BX 2018 -0.56 (-1.05, -0.07) 0.23
Li SQ 2016 -0.40 (-1.16, 0.36) 0.10
Zhang CR 2016 -0.40 (-1.18, 0.38) 0.09
Zhu XX 2015 -0.80 (-1.37, -0.23) 0.17
Wang T 2015 -0.30 (-0.76, 0.16) 0.27
Ma ZX 2015 -0.77 (-1.04, -0.50) 0.75
Zhou YQ 2015 -1.30 (-1.62, -0.98) 0.55
Zhang CH 2015 -1.85 (-2.38, -1.32) 0.20
Ren YL 2015 -1.18 (-2.09, -0.27) 0.07
Yang HM 2015 -0.70 (-1.70, 0.30) 0.06
Shen CM 2014 -0.29 (-0.70, 0.12) 0.33
Du YJ 2014 -0.27 (-0.54, 0.00) 0.75
Zhang Y 2013 -1.33 (-1.82, -0.84) 0.23
Bi XS 2013 -1.00 (-1.73, -0.27) 0.11
Zhong J 2013 -0.22 (-0.33, -0.11) 4.35
Jiang H 2012 -0.70 (-1.19, -0.21) 0.24
Zhu YF 2012 -0.70 (-1.33, -0.07) 0.14
Teng YP 2012 -0.01 (-0.56, 0.54) 0.18
Yang YB 2012B -0.60 (-1.08, -0.12) 0.24
Guo HF 2011 -1.63 (-1.99, -1.27) 0.43
Hong YJ 2010 -1.23 (-1.88, -0.58) 0.13
Deng JH 2009 -0.60 (-1.10, -0.10) 0.22
Subgroup, IV (I² = 90.9%, p = 0.000) -0.81 (-0.86, -0.75) 18.76

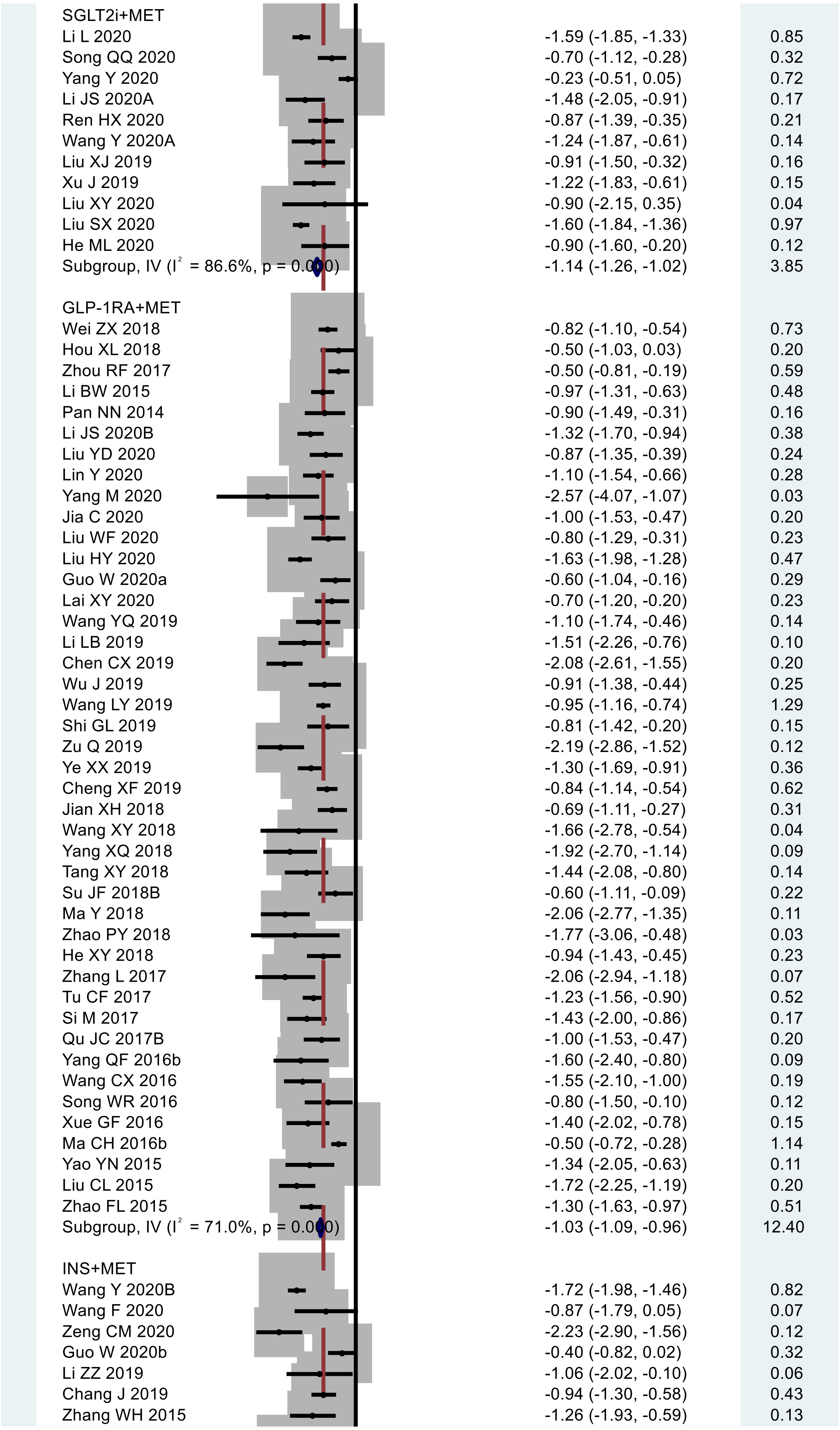
SGLT2i+MET
Li L 2020 -1.59 (-1.85, -1.33) 0.85
Song QQ 2020 -0.70 (-1.12, -0.28) 0.32
Yang Y 2020 -0.23 (-0.51, 0.05) 0.72
Li JS 2020A -1.48 (-2.05, -0.91) 0.17
Ren HX 2020 -0.87 (-1.39, -0.35) 0.21
Wang Y 2020A -1.24 (-1.87, -0.61) 0.14
Liu XJ 2019 -0.91 (-1.50, -0.32) 0.16
Xu J 2019 -1.22 (-1.83, -0.61) 0.15
Liu XY 2020 -0.90 (-2.15, 0.35) 0.04
Liu SX 2020 -1.60 (-1.84, -1.36) 0.97
He ML 2020 -0.90 (-1.60, -0.20) 0.12
Subgroup, IV (I² = 86.6%, p = 0.0[illegible]0) -1.14 (-1.26, -1.02) 3.85
GLP-1RA+MET
Wei ZX 2018 -0.82 (-1.10, -0.54) 0.73
Hou XL 2018 -0.50 (-1.03, 0.03) 0.20
Zhou RF 2017 -0.50 (-0.81, -0.19) 0.59
Li BW 2015 -0.97 (-1.31, -0.63) 0.48
Pan NN 2014 -0.90 (-1.49, -0.31) 0.16
Li JS 2020B -1.32 (-1.70, -0.94) 0.38
Liu YD 2020 -0.87 (-1.35, -0.39) 0.24
Lin Y 2020 -1.10 (-1.54, -0.66) 0.28
Yang M 2020 -2.57 (-4.07, -1.07) 0.03
Jia C 2020 -1.00 (-1.53, -0.47) 0.20
Liu WF 2020 -0.80 (-1.29, -0.31) 0.23
Liu HY 2020 -1.63 (-1.98, -1.28) 0.47
Guo W 2020a -0.60 (-1.04, -0.16) 0.29
Lai XY 2020 -0.70 (-1.20, -0.20) 0.23
Wang YQ 2019 -1.10 (-1.74, -0.46) 0.14
Li LB 2019 -1.51 (-2.26, -0.76) 0.10
Chen CX 2019 -2.08 (-2.61, -1.55) 0.20
Wu J 2019 -0.91 (-1.38, -0.44) 0.25
Wang LY 2019 -0.95 (-1.16, -0.74) 1.29
Shi GL 2019 -0.81 (-1.42, -0.20) 0.15
Zu Q 2019 -2.19 (-2.86, -1.52) 0.12
Ye XX 2019 -1.30 (-1.69, -0.91) 0.36
Cheng XF 2019 -0.84 (-1.14, -0.54) 0.62
Jian XH 2018 -0.69 (-1.11, -0.27) 0.31
Wang XY 2018 -1.66 (-2.78, -0.54) 0.04
Yang XQ 2018 -1.92 (-2.70, -1.14) 0.09
Tang XY 2018 -1.44 (-2.08, -0.80) 0.14
Su JF 2018B -0.60 (-1.11, -0.09) 0.22
Ma Y 2018 -2.06 (-2.77, -1.35) 0.11
Zhao PY 2018 -1.77 (-3.06, -0.48) 0.03
He XY 2018 -0.94 (-1.43, -0.45) 0.23
Zhang L 2017 -2.06 (-2.94, -1.18) 0.07
Tu CF 2017 -1.23 (-1.56, -0.90) 0.52
Si M 2017 -1.43 (-2.00, -0.86) 0.17
Qu JC 2017B -1.00 (-1.53, -0.47) 0.20
Yang QF 2016b -1.60 (-2.40, -0.80) 0.09
Wang CX 2016 -1.55 (-2.10, -1.00) 0.19
Song WR 2016 -0.80 (-1.50, -0.10) 0.12
Xue GF 2016 -1.40 (-2.02, -0.78) 0.15
Ma CH 2016b -0.50 (-0.72, -0.28) 1.14
Yao YN 2015 -1.34 (-2.05, -0.63) 0.11
Liu CL 2015 -1.72 (-2.25, -1.19) 0.20
Zhao FL 2015 -1.30 (-1.63, -0.97) 0.51
Subgroup, IV (I² = 71.0%, p = 0.0[illegible]0) -1.03 (-1.09, -0.96) 12.40
INS+MET
Wang Y 2020B -1.72 (-1.98, -1.46) 0.82
Wang F 2020 -0.87 (-1.79, 0.05) 0.07
Zeng CM 2020 -2.23 (-2.90, -1.56) 0.12
Guo W 2020b -0.40 (-0.82, 0.02) 0.32
Li ZZ 2019 -1.06 (-2.02, -0.10) 0.06
Chang J 2019 -0.94 (-1.30, -0.58) 0.43
Zhang WH 2015 -1.26 (-1.93, -0.59) 0.13

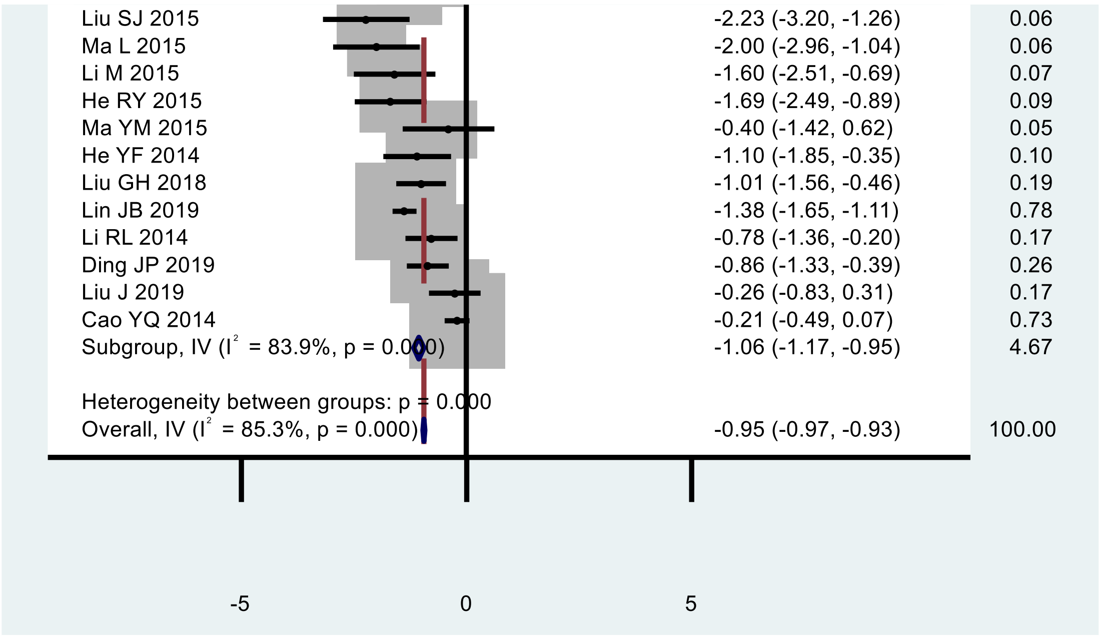

Figure S61. Sensitivity analysis results for change in hemoglobin Alc (%) of glucose-lowering drugs added to metformin compared with metformin monotherapy

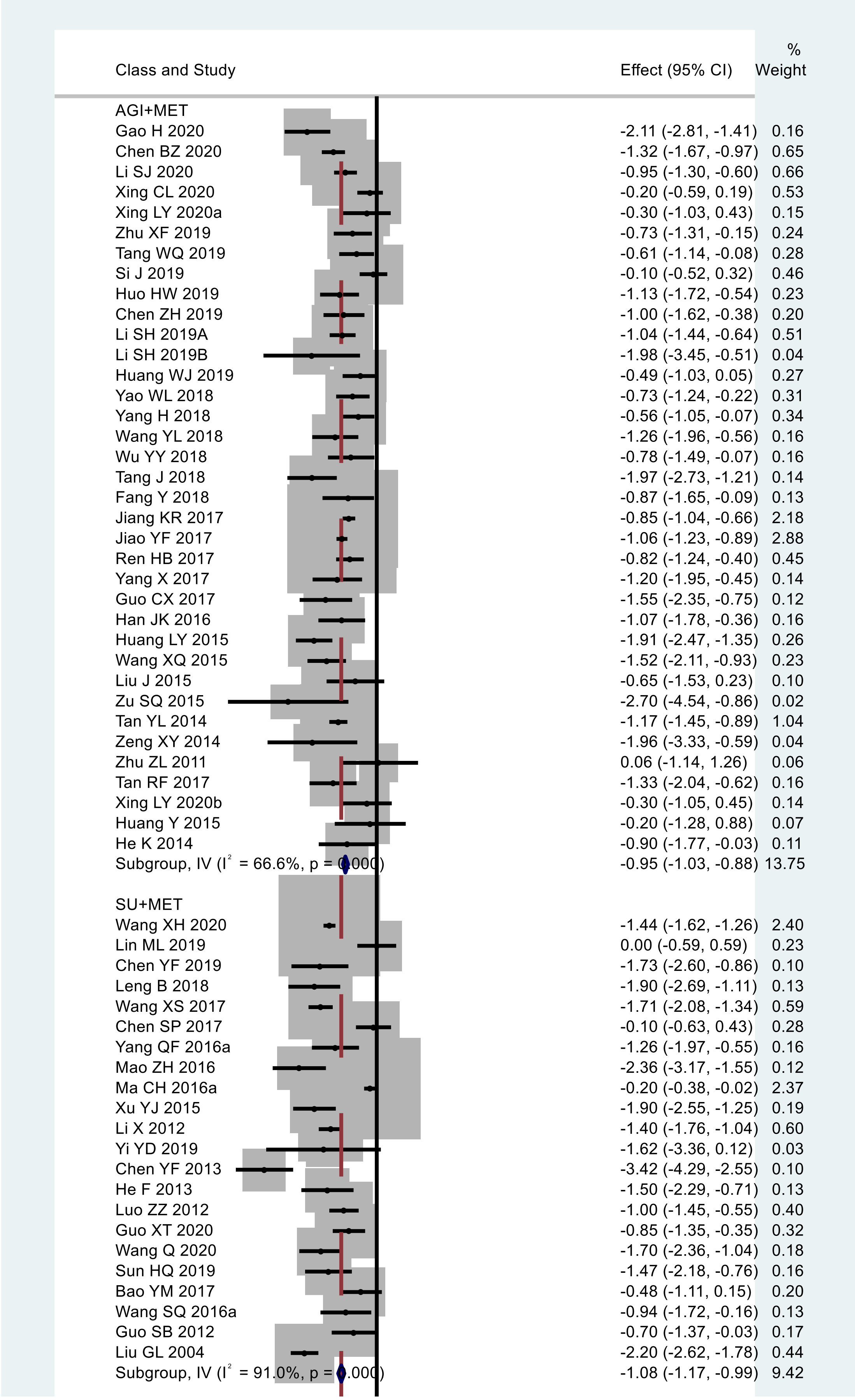
Class and Study
Effect (95% CI)
% Weight
AGI+MET
Gao H 2020 -2.11 (-2.81, -1.41) 0.16
Chen BZ 2020 -1.32 (-1.67, -0.97) 0.65
Li SJ 2020 -0.95 (-1.30, -0.60) 0.66
Xing CL 2020 -0.20 (-0.59, 0.19) 0.53
Xing LY 2020a -0.30 (-1.03, 0.43) 0.15
Zhu XF 2019 -0.73 (-1.31, -0.15) 0.24
Tang WQ 2019 -0.61 (-1.14, -0.08) 0.28
Si J 2019 -0.10 (-0.52, 0.32) 0.46
Huo HW 2019 -1.13 (-1.72, -0.54) 0.23
Chen ZH 2019 -1.00 (-1.62, -0.38) 0.20
Li SH 2019A -1.04 (-1.44, -0.64) 0.51
Li SH 2019B -1.98 (-3.45, -0.51) 0.04
Huang WJ 2019 -0.49 (-1.03, 0.05) 0.27
Yao WL 2018 -0.73 (-1.24, -0.22) 0.31
Yang H 2018 -0.56 (-1.05, -0.07) 0.34
Wang YL 2018 -1.26 (-1.96, -0.56) 0.16
Wu YY 2018 -0.78 (-1.49, -0.07) 0.16
Tang J 2018 -1.97 (-2.73, -1.21) 0.14
Fang Y 2018 -0.87 (-1.65, -0.09) 0.13
Jiang KR 2017 -0.85 (-1.04, -0.66) 2.18
Jiao YF 2017 -1.06 (-1.23, -0.89) 2.88
Ren HB 2017 -0.82 (-1.24, -0.40) 0.45
Yang X 2017 -1.20 (-1.95, -0.45) 0.14
Guo CX 2017 -1.55 (-2.35, -0.75) 0.12
Han JK 2016 -1.07 (-1.78, -0.36) 0.16
Huang LY 2015 -1.91 (-2.47, -1.35) 0.26
Wang XQ 2015 -1.52 (-2.11, -0.93) 0.23
Liu J 2015 -0.65 (-1.53, 0.23) 0.10
Zu SQ 2015 -2.70 (-4.54, -0.86) 0.02
Tan YL 2014 -1.17 (-1.45, -0.89) 1.04
Zeng XY 2014 -1.96 (-3.33, -0.59) 0.04
Zhu ZL 2011 0.06 (-1.14, 1.26) 0.06
Tan RF 2017 -1.33 (-2.04, -0.62) 0.16
Xing LY 2020b -0.30 (-1.05, 0.45) 0.14
Huang Y 2015 -0.20 (-1.28, 0.88) 0.07
He K 2014 -0.90 (-1.77, -0.03) 0.11
Subgroup, IV (I² = 66.6%, p = 0.000) -0.95 (-1.03, -0.88) 13.75
SU+MET
Wang XH 2020 -1.44 (-1.62, -1.26) 2.40
Lin ML 2019 0.00 (-0.59, 0.59) 0.23
Chen YF 2019 -1.73 (-2.60, -0.86) 0.10
Leng B 2018 -1.90 (-2.69, -1.11) 0.13
Wang XS 2017 -1.71 (-2.08, -1.34) 0.59
Chen SP 2017 -0.10 (-0.63, 0.43) 0.28
Yang QF 2016a -1.26 (-1.97, -0.55) 0.16
Mao ZH 2016 -2.36 (-3.17, -1.55) 0.12
Ma CH 2016a -0.20 (-0.38, -0.02) 2.37
Xu YJ 2015 -1.90 (-2.55, -1.25) 0.19
Li X 2012 -1.40 (-1.76, -1.04) 0.60
Yi YD 2019 -1.62 (-3.36, 0.12) 0.03
Chen YF 2013 -3.42 (-4.29, -2.55) 0.10
He F 2013 -1.50 (-2.29, -0.71) 0.13
Luo ZZ 2012 -1.00 (-1.45, -0.55) 0.40
Guo XT 2020 -0.85 (-1.35, -0.35) 0.32
Wang Q 2020 -1.70 (-2.36, -1.04) 0.18
Sun HQ 2019 -1.47 (-2.18, -0.76) 0.16
Bao YM 2017 -0.48 (-1.11, 0.15) 0.20
Wang SQ 2016a -0.94 (-1.72, -0.16) 0.13
Guo SB 2012 -0.70 (-1.37, -0.03) 0.17
Liu GL 2004 -2.20 (-2.62, -1.78) 0.44
Subgroup, IV (I² = 91.0%, p = 0.000) -1.08 (-1.17, -0.99) 9.42

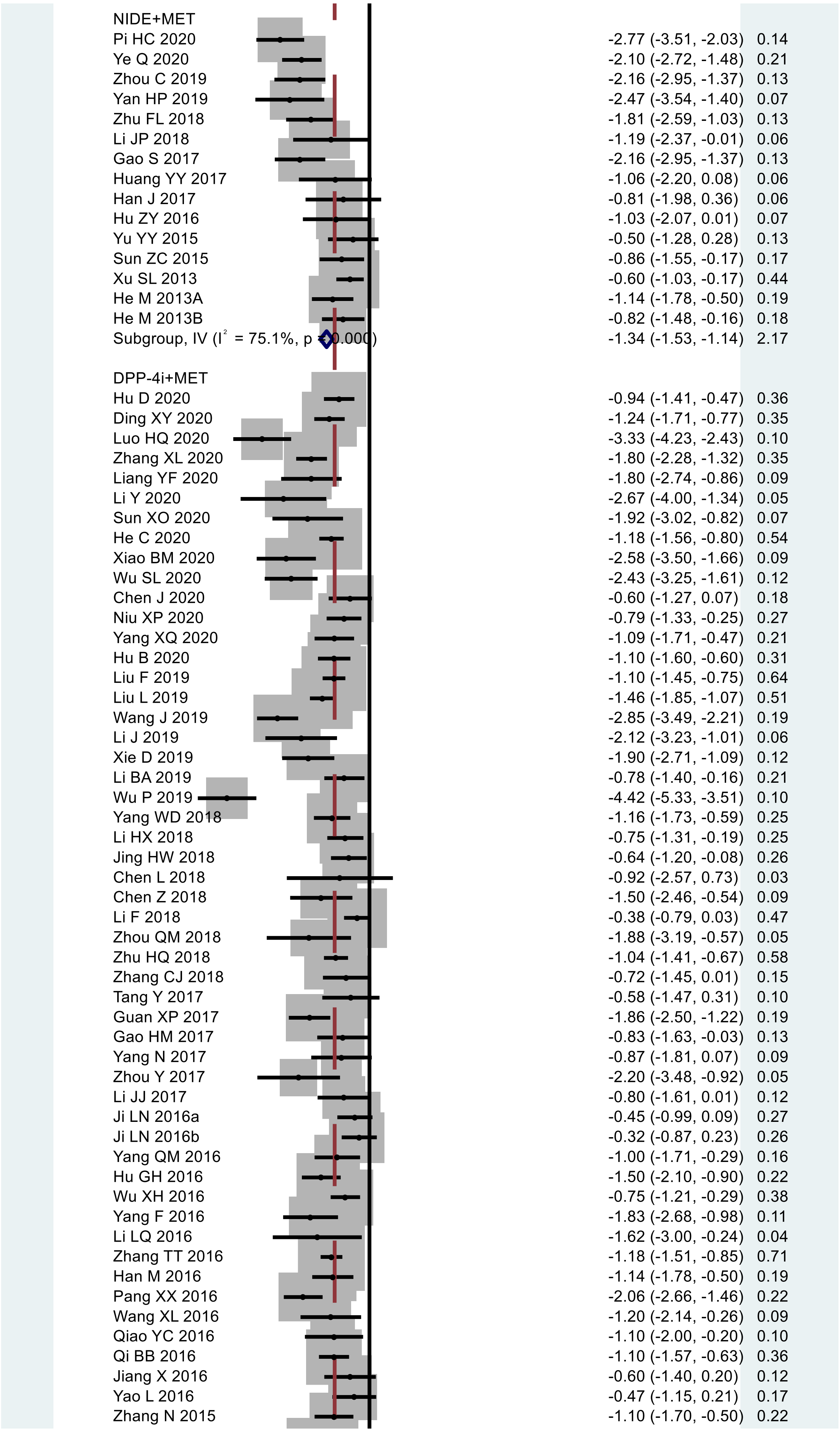
NIDE+MET
Pi HC 2020 -2.77 (-3.51, -2.03) 0.14
Ye Q 2020 -2.10 (-2.72, -1.48) 0.21
Zhou C 2019 -2.16 (-2.95, -1.37) 0.13
Yan HP 2019 -2.47 (-3.54, -1.40) 0.07
Zhu FL 2018 -1.81 (-2.59, -1.03) 0.13
Li JP 2018 -1.19 (-2.37, -0.01) 0.06
Gao S 2017 -2.16 (-2.95, -1.37) 0.13
Huang YY 2017 -1.06 (-2.20, 0.08) 0.06
Han J 2017 -0.81 (-1.98, 0.36) 0.06
Hu ZY 2016 -1.03 (-2.07, 0.01) 0.07
Yu YY 2015 -0.50 (-1.28, 0.28) 0.13
Sun ZC 2015 -0.86 (-1.55, -0.17) 0.17
Xu SL 2013 -0.60 (-1.03, -0.17) 0.44
He M 2013A -1.14 (-1.78, -0.50) 0.19
He M 2013B -0.82 (-1.48, -0.16) 0.18
Subgroup, IV (I^2 = 75.1%, p 0.000) -1.34 (-1.53, -1.14) 2.17
DPP-4i+MET
Hu D 2020 -0.94 (-1.41, -0.47) 0.36
Ding XY 2020 -1.24 (-1.71, -0.77) 0.35
Luo HQ 2020 -3.33 (-4.23, -2.43) 0.10
Zhang XL 2020 -1.80 (-2.28, -1.32) 0.35
Liang YF 2020 -1.80 (-2.74, -0.86) 0.09
Li Y 2020 -2.67 (-4.00, -1.34) 0.05
Sun XO 2020 -1.92 (-3.02, -0.82) 0.07
He C 2020 -1.18 (-1.56, -0.80) 0.54
Xiao BM 2020 -2.58 (-3.50, -1.66) 0.09
Wu SL 2020 -2.43 (-3.25, -1.61) 0.12
Chen J 2020 -0.60 (-1.27, 0.07) 0.18
Niu XP 2020 -0.79 (-1.33, -0.25) 0.27
Yang XQ 2020 -1.09 (-1.71, -0.47) 0.21
Hu B 2020 -1.10 (-1.60, -0.60) 0.31
Liu F 2019 -1.10 (-1.45, -0.75) 0.64
Liu L 2019 -1.46 (-1.85, -1.07) 0.51
Wang J 2019 -2.85 (-3.49, -2.21) 0.19
Li J 2019 -2.12 (-3.23, -1.01) 0.06
Xie D 2019 -1.90 (-2.71, -1.09) 0.12
Li BA 2019 -0.78 (-1.40, -0.16) 0.21
Wu P 2019 -4.42 (-5.33, -3.51) 0.10
Yang WD 2018 -1.16 (-1.73, -0.59) 0.25
Li HX 2018 -0.75 (-1.31, -0.19) 0.25
Jing HW 2018 -0.64 (-1.20, -0.08) 0.26
Chen L 2018 -0.92 (-2.57, 0.73) 0.03
Chen Z 2018 -1.50 (-2.46, -0.54) 0.09
Li F 2018 -0.38 (-0.79, 0.03) 0.47
Zhou QM 2018 -1.88 (-3.19, -0.57) 0.05
Zhu HQ 2018 -1.04 (-1.41, -0.67) 0.58
Zhang CJ 2018 -0.72 (-1.45, 0.01) 0.15
Tang Y 2017 -0.58 (-1.47, 0.31) 0.10
Guan XP 2017 -1.86 (-2.50, -1.22) 0.19
Gao HM 2017 -0.83 (-1.63, -0.03) 0.13
Yang N 2017 -0.87 (-1.81, 0.07) 0.09
Zhou Y 2017 -2.20 (-3.48, -0.92) 0.05
Li JJ 2017 -0.80 (-1.61, 0.01) 0.12
Ji LN 2016a -0.45 (-0.99, 0.09) 0.27
Ji LN 2016b -0.32 (-0.87, 0.23) 0.26
Yang QM 2016 -1.00 (-1.71, -0.29) 0.16
Hu GH 2016 -1.50 (-2.10, -0.90) 0.22
Wu XH 2016 -0.75 (-1.21, -0.29) 0.38
Yang F 2016 -1.83 (-2.68, -0.98) 0.11
Li LQ 2016 -1.62 (-3.00, -0.24) 0.04
Zhang TT 2016 -1.18 (-1.51, -0.85) 0.71
Han M 2016 -1.14 (-1.78, -0.50) 0.19
Pang XX 2016 -2.06 (-2.66, -1.46) 0.22
Wang XL 2016 -1.20 (-2.14, -0.26) 0.09
Qiao YC 2016 -1.10 (-2.00, -0.20) 0.10
Qi BB 2016 -1.10 (-1.57, -0.63) 0.36
Jiang X 2016 -0.60 (-1.40, 0.20) 0.12
Yao L 2016 -0.47 (-1.15, 0.21) 0.17
Zhang N 2015 -1.10 (-1.70, -0.50) 0.22

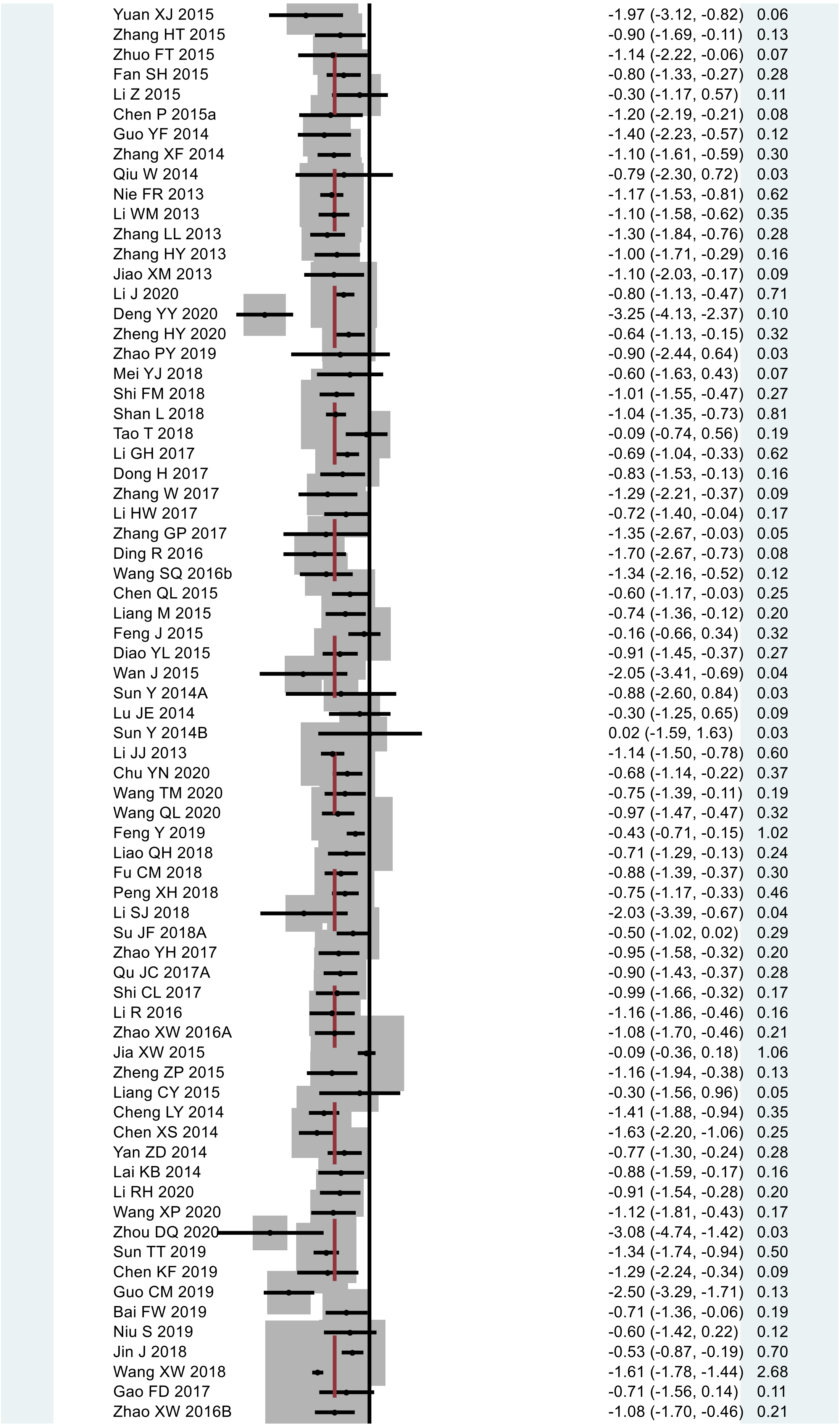
Yuan XJ 2015 -1.97 (-3.12, -0.82) 0.06
Zhang HT 2015 -0.90 (-1.69, -0.11) 0.13
Zhuo FT 2015 -1.14 (-2.22, -0.06) 0.07
Fan SH 2015 -0.80 (-1.33, -0.27) 0.28
Li Z 2015 -0.30 (-1.17, 0.57) 0.11
Chen P 2015a -1.20 (-2.19, -0.21) 0.08
Guo YF 2014 -1.40 (-2.23, -0.57) 0.12
Zhang XF 2014 -1.10 (-1.61, -0.59) 0.30
Qiu W 2014 -0.79 (-2.30, 0.72) 0.03
Nie FR 2013 -1.17 (-1.53, -0.81) 0.62
Li WM 2013 -1.10 (-1.58, -0.62) 0.35
Zhang LL 2013 -1.30 (-1.84, -0.76) 0.28
Zhang HY 2013 -1.00 (-1.71, -0.29) 0.16
Jiao XM 2013 -1.10 (-2.03, -0.17) 0.09
Li J 2020 -0.80 (-1.13, -0.47) 0.71
Deng YY 2020 -3.25 (-4.13, -2.37) 0.10
Zheng HY 2020 -0.64 (-1.13, -0.15) 0.32
Zhao PY 2019 -0.90 (-2.44, 0.64) 0.03
Mei YJ 2018 -0.60 (-1.63, 0.43) 0.07
Shi FM 2018 -1.01 (-1.55, -0.47) 0.27
Shan L 2018 -1.04 (-1.35, -0.73) 0.81
Tao T 2018 -0.09 (-0.74, 0.56) 0.19
Li GH 2017 -0.69 (-1.04, -0.33) 0.62
Dong H 2017 -0.83 (-1.53, -0.13) 0.16
Zhang W 2017 -1.29 (-2.21, -0.37) 0.09
Li HW 2017 -0.72 (-1.40, -0.04) 0.17
Zhang GP 2017 -1.35 (-2.67, -0.03) 0.05
Ding R 2016 -1.70 (-2.67, -0.73) 0.08
Wang SQ 2016b -1.34 (-2.16, -0.52) 0.12
Chen QL 2015 -0.60 (-1.17, -0.03) 0.25
Liang M 2015 -0.74 (-1.36, -0.12) 0.20
Feng J 2015 -0.16 (-0.66, 0.34) 0.32
Diao YL 2015 -0.91 (-1.45, -0.37) 0.27
Wan J 2015 -2.05 (-3.41, -0.69) 0.04
Sun Y 2014A -0.88 (-2.60, 0.84) 0.03
Lu JE 2014 -0.30 (-1.25, 0.65) 0.09
Sun Y 2014B 0.02 (-1.59, 1.63) 0.03
Li JJ 2013 -1.14 (-1.50, -0.78) 0.60
Chu YN 2020 -0.68 (-1.14, -0.22) 0.37
Wang TM 2020 -0.75 (-1.39, -0.11) 0.19
Wang QL 2020 -0.97 (-1.47, -0.47) 0.32
Feng Y 2019 -0.43 (-0.71, -0.15) 1.02
Liao QH 2018 -0.71 (-1.29, -0.13) 0.24
Fu CM 2018 -0.88 (-1.39, -0.37) 0.30
Peng XH 2018 -0.75 (-1.17, -0.33) 0.46
Li SJ 2018 -2.03 (-3.39, -0.67) 0.04
Su JF 2018A -0.50 (-1.02, 0.02) 0.29
Zhao YH 2017 -0.95 (-1.58, -0.32) 0.20
Qu JC 2017A -0.90 (-1.43, -0.37) 0.28
Shi CL 2017 -0.99 (-1.66, -0.32) 0.17
Li R 2016 -1.16 (-1.86, -0.46) 0.16
Zhao XW 2016A -1.08 (-1.70, -0.46) 0.21
Jia XW 2015 -0.09 (-0.36, 0.18) 1.06
Zheng ZP 2015 -1.16 (-1.94, -0.38) 0.13
Liang CY 2015 -0.30 (-1.56, 0.96) 0.05
Cheng LY 2014 -1.41 (-1.88, -0.94) 0.35
Chen XS 2014 -1.63 (-2.20, -1.06) 0.25
Yan ZD 2014 -0.77 (-1.30, -0.24) 0.28
Lai KB 2014 -0.88 (-1.59, -0.17) 0.16
Li RH 2020 -0.91 (-1.54, -0.28) 0.20
Wang XP 2020 -1.12 (-1.81, -0.43) 0.17
Zhou DQ 2020 -3.08 (-4.74, -1.42) 0.03
Sun TT 2019 -1.34 (-1.74, -0.94) 0.50
Chen KF 2019 -1.29 (-2.24, -0.34) 0.09
Guo CM 2019 -2.50 (-3.29, -1.71) 0.13
Bai FW 2019 -0.71 (-1.36, -0.06) 0.19
Niu S 2019 -0.60 (-1.42, 0.22) 0.12
Jin J 2018 -0.53 (-0.87, -0.19) 0.70
Wang XW 2018 -1.61 (-1.78, -1.44) 2.68
Gao FD 2017 -0.71 (-1.56, 0.14) 0.11
Zhao XW 2016B -1.08 (-1.70, -0.46) 0.21

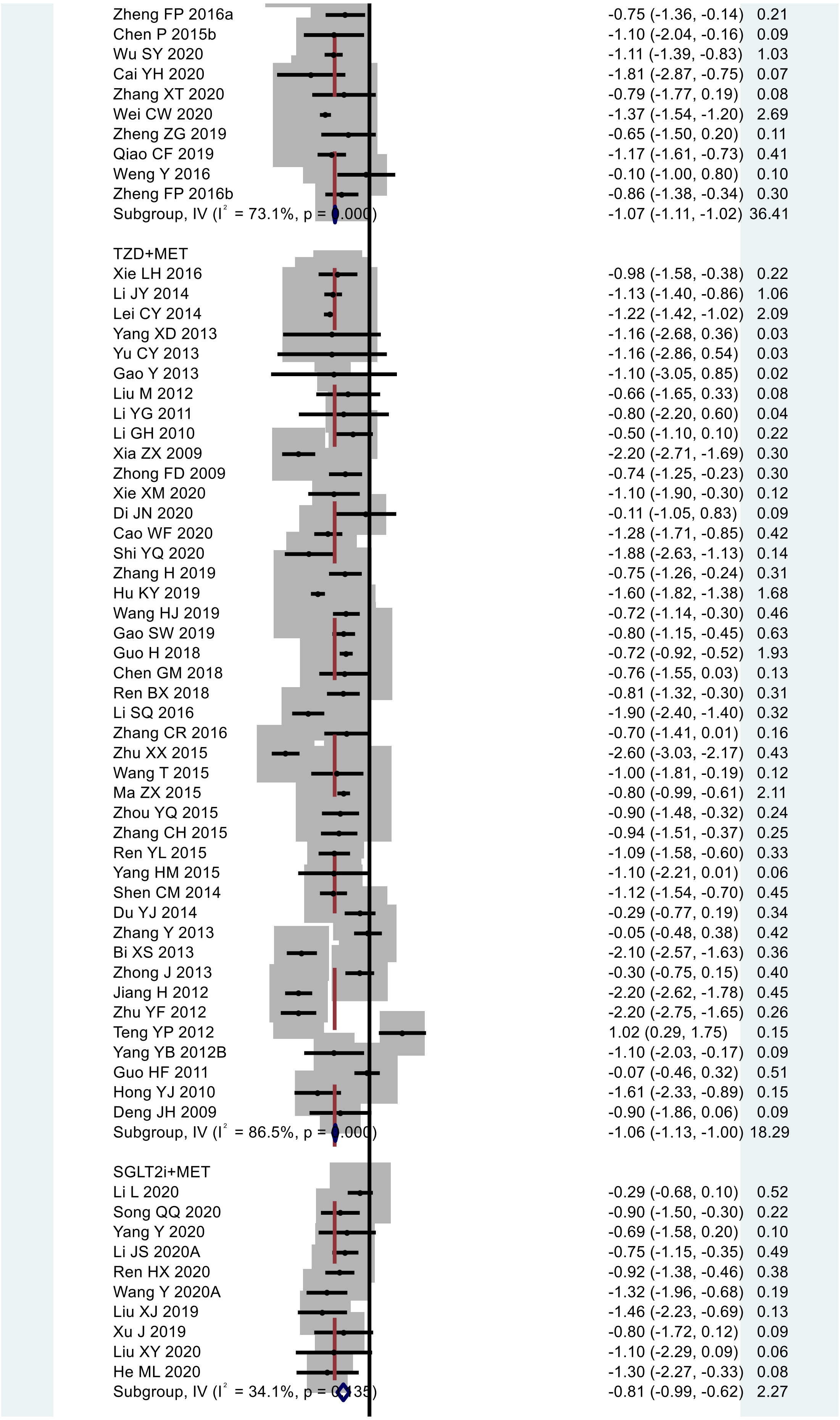
Zheng FP 2016a -0.75 (-1.36, -0.14) 0.21
Chen P 2015b -1.10 (-2.04, -0.16) 0.09
Wu SY 2020 -1.11 (-1.39, -0.83) 1.03
Cai YH 2020 -1.81 (-2.87, -0.75) 0.07
Zhang XT 2020 -0.79 (-1.77, 0.19) 0.08
Wei CW 2020 -1.37 (-1.54, -1.20) 2.69
Zheng ZG 2019 -0.65 (-1.50, 0.20) 0.11
Qiao CF 2019 -1.17 (-1.61, -0.73) 0.41
Weng Y 2016 -0.10 (-1.00, 0.80) 0.10
Zheng FP 2016b -0.86 (-1.38, -0.34) 0.30
Subgroup, IV (I^2 = 73.1%, p = .000) -1.07 (-1.11, -1.02) 36.41
TZD+MET
Xie LH 2016 -0.98 (-1.58, -0.38) 0.22
Li JY 2014 -1.13 (-1.40, -0.86) 1.06
Lei CY 2014 -1.22 (-1.42, -1.02) 2.09
Yang XD 2013 -1.16 (-2.68, 0.36) 0.03
Yu CY 2013 -1.16 (-2.86, 0.54) 0.03
Gao Y 2013 -1.10 (-3.05, 0.85) 0.02
Liu M 2012 -0.66 (-1.65, 0.33) 0.08
Li YG 2011 -0.80 (-2.20, 0.60) 0.04
Li GH 2010 -0.50 (-1.10, 0.10) 0.22
Xia ZX 2009 -2.20 (-2.71, -1.69) 0.30
Zhong FD 2009 -0.74 (-1.25, -0.23) 0.30
Xie XM 2020 -1.10 (-1.90, -0.30) 0.12
Di JN 2020 -0.11 (-1.05, 0.83) 0.09
Cao WF 2020 -1.28 (-1.71, -0.85) 0.42
Shi YQ 2020 -1.88 (-2.63, -1.13) 0.14
Zhang H 2019 -0.75 (-1.26, -0.24) 0.31
Hu KY 2019 -1.60 (-1.82, -1.38) 1.68
Wang HJ 2019 -0.72 (-1.14, -0.30) 0.46
Gao SW 2019 -0.80 (-1.15, -0.45) 0.63
Guo H 2018 -0.72 (-0.92, -0.52) 1.93
Chen GM 2018 -0.76 (-1.55, 0.03) 0.13
Ren BX 2018 -0.81 (-1.32, -0.30) 0.31
Li SQ 2016 -1.90 (-2.40, -1.40) 0.32
Zhang CR 2016 -0.70 (-1.41, 0.01) 0.16
Zhu XX 2015 -2.60 (-3.03, -2.17) 0.43
Wang T 2015 -1.00 (-1.81, -0.19) 0.12
Ma ZX 2015 -0.80 (-0.99, -0.61) 2.11
Zhou YQ 2015 -0.90 (-1.48, -0.32) 0.24
Zhang CH 2015 -0.94 (-1.51, -0.37) 0.25
Ren YL 2015 -1.09 (-1.58, -0.60) 0.33
Yang HM 2015 -1.10 (-2.21, 0.01) 0.06
Shen CM 2014 -1.12 (-1.54, -0.70) 0.45
Du YJ 2014 -0.29 (-0.77, 0.19) 0.34
Zhang Y 2013 -0.05 (-0.48, 0.38) 0.42
Bi XS 2013 -2.10 (-2.57, -1.63) 0.36
Zhong J 2013 -0.30 (-0.75, 0.15) 0.40
Jiang H 2012 -2.20 (-2.62, -1.78) 0.45
Zhu YF 2012 -2.20 (-2.75, -1.65) 0.26
Teng YP 2012 1.02 (0.29, 1.75) 0.15
Yang YB 2012B -1.10 (-2.03, -0.17) 0.09
Guo HF 2011 -0.07 (-0.46, 0.32) 0.51
Hong YJ 2010 -1.61 (-2.33, -0.89) 0.15
Deng JH 2009 -0.90 (-1.86, 0.06) 0.09
Subgroup, IV (I^2 = 86.5%, p = .000) -1.06 (-1.13, -1.00) 18.29
SGLT2i+MET
Li L 2020 -0.29 (-0.68, 0.10) 0.52
Song QQ 2020 -0.90 (-1.50, -0.30) 0.22
Yang Y 2020 -0.69 (-1.58, 0.20) 0.10
Li JS 2020A -0.75 (-1.15, -0.35) 0.49
Ren HX 2020 -0.92 (-1.38, -0.46) 0.38
Wang Y 2020A -1.32 (-1.96, -0.68) 0.19
Liu XJ 2019 -1.46 (-2.23, -0.69) 0.13
Xu J 2019 -0.80 (-1.72, 0.12) 0.09
Liu XY 2020 -1.10 (-2.29, 0.09) 0.06
He ML 2020 -1.30 (-2.27, -0.33) 0.08
Subgroup, IV (I^2 = 34.1%, p = 0 35) -0.81 (-0.99, -0.62) 2.27

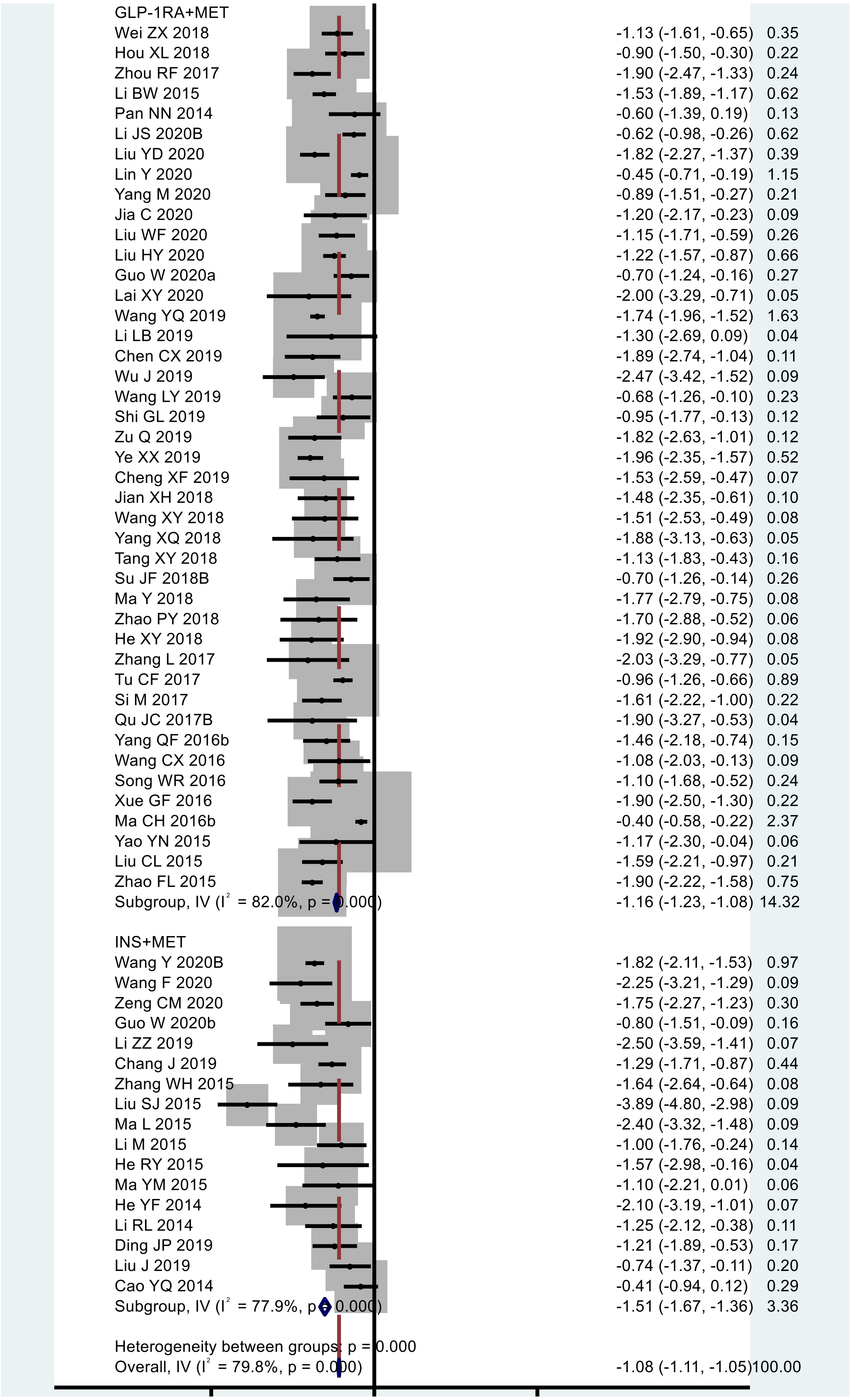

Figure S62. Sensitivity analysis results for change in fasting plasma glucose (mmol/l) of glucose-lowering drugs added to metformin compared with metformin monotherapy

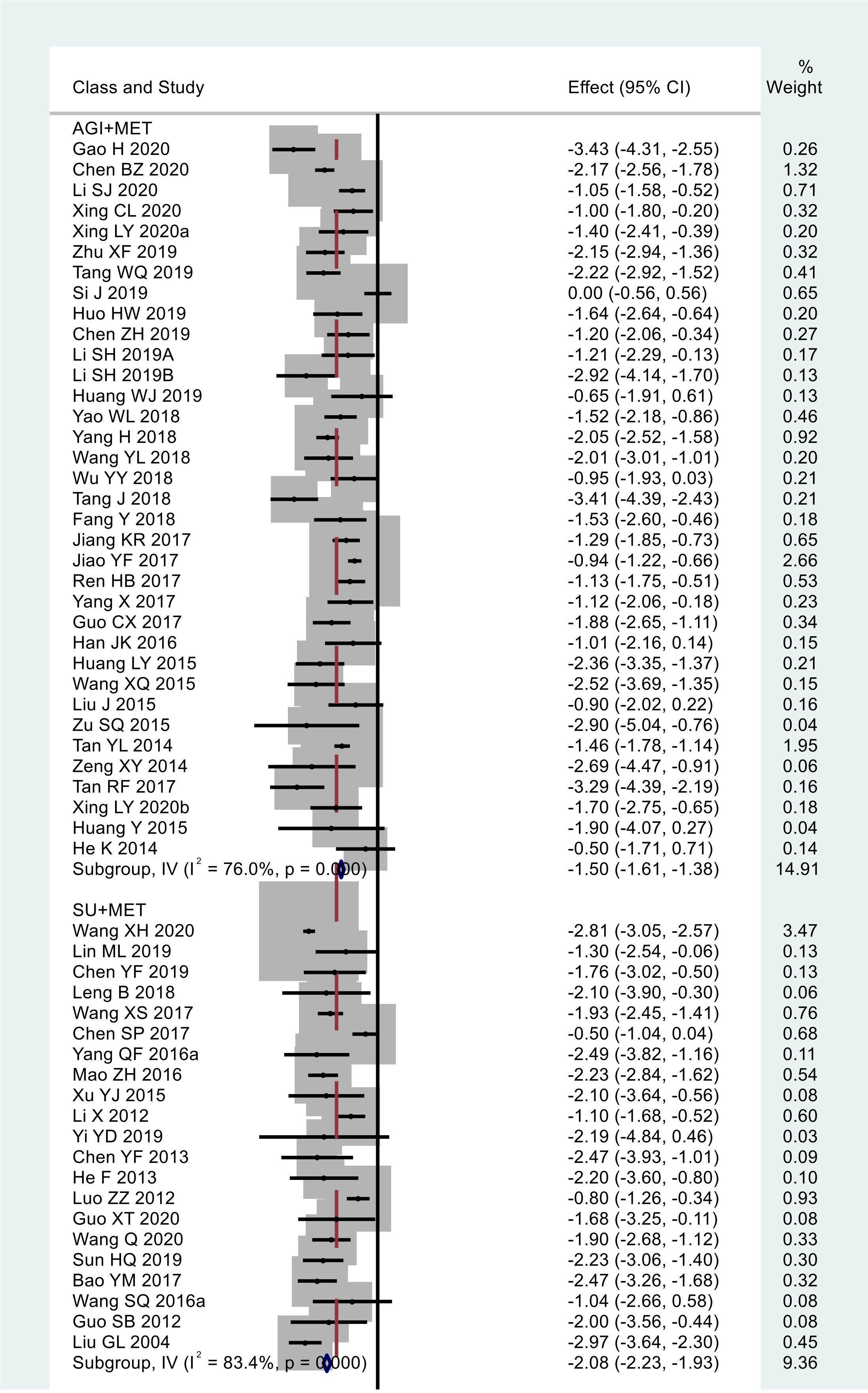
Class and Study
Effect (95% CI)
% Weight
AGI+MET
Gao H 2020 -3.43 (-4.31, -2.55) 0.26
Chen BZ 2020 -2.17 (-2.56, -1.78) 1.32
Li SJ 2020 -1.05 (-1.58, -0.52) 0.71
Xing CL 2020 -1.00 (-1.80, -0.20) 0.32
Xing LY 2020a -1.40 (-2.41, -0.39) 0.20
Zhu XF 2019 -2.15 (-2.94, -1.36) 0.32
Tang WQ 2019 -2.22 (-2.92, -1.52) 0.41
Si J 2019 0.00 (-0.56, 0.56) 0.65
Huo HW 2019 -1.64 (-2.64, -0.64) 0.20
Chen ZH 2019 -1.20 (-2.06, -0.34) 0.27
Li SH 2019A -1.21 (-2.29, -0.13) 0.17
Li SH 2019B -2.92 (-4.14, -1.70) 0.13
Huang WJ 2019 -0.65 (-1.91, 0.61) 0.13
Yao WL 2018 -1.52 (-2.18, -0.86) 0.46
Yang H 2018 -2.05 (-2.52, -1.58) 0.92
Wang YL 2018 -2.01 (-3.01, -1.01) 0.20
Wu YY 2018 -0.95 (-1.93, 0.03) 0.21
Tang J 2018 -3.41 (-4.39, -2.43) 0.21
Fang Y 2018 -1.53 (-2.60, -0.46) 0.18
Jiang KR 2017 -1.29 (-1.85, -0.73) 0.65
Jiao YF 2017 -0.94 (-1.22, -0.66) 2.66
Ren HB 2017 -1.13 (-1.75, -0.51) 0.53
Yang X 2017 -1.12 (-2.06, -0.18) 0.23
Guo CX 2017 -1.88 (-2.65, -1.11) 0.34
Han JK 2016 -1.01 (-2.16, 0.14) 0.15
Huang LY 2015 -2.36 (-3.35, -1.37) 0.21
Wang XQ 2015 -2.52 (-3.69, -1.35) 0.15
Liu J 2015 -0.90 (-2.02, 0.22) 0.16
Zu SQ 2015 -2.90 (-5.04, -0.76) 0.04
Tan YL 2014 -1.46 (-1.78, -1.14) 1.95
Zeng XY 2014 -2.69 (-4.47, -0.91) 0.06
Tan RF 2017 -3.29 (-4.39, -2.19) 0.16
Xing LY 2020b -1.70 (-2.75, -0.65) 0.18
Huang Y 2015 -1.90 (-4.07, 0.27) 0.04
He K 2014 -0.50 (-1.71, 0.71) 0.14
Subgroup, IV (I² = 76.0%, p = 0.000) -1.50 (-1.61, -1.38) 14.91
SU+MET
Wang XH 2020 -2.81 (-3.05, -2.57) 3.47
Lin ML 2019 -1.30 (-2.54, -0.06) 0.13
Chen YF 2019 -1.76 (-3.02, -0.50) 0.13
Leng B 2018 -2.10 (-3.90, -0.30) 0.06
Wang XS 2017 -1.93 (-2.45, -1.41) 0.76
Chen SP 2017 -0.50 (-1.04, 0.04) 0.68
Yang QF 2016a -2.49 (-3.82, -1.16) 0.11
Mao ZH 2016 -2.23 (-2.84, -1.62) 0.54
Xu YJ 2015 -2.10 (-3.64, -0.56) 0.08
Li X 2012 -1.10 (-1.68, -0.52) 0.60
Yi YD 2019 -2.19 (-4.84, 0.46) 0.03
Chen YF 2013 -2.47 (-3.93, -1.01) 0.09
He F 2013 -2.20 (-3.60, -0.80) 0.10
Luo ZZ 2012 -0.80 (-1.26, -0.34) 0.93
Guo XT 2020 -1.68 (-3.25, -0.11) 0.08
Wang Q 2020 -1.90 (-2.68, -1.12) 0.33
Sun HQ 2019 -2.23 (-3.06, -1.40) 0.30
Bao YM 2017 -2.47 (-3.26, -1.68) 0.32
Wang SQ 2016a -1.04 (-2.66, 0.58) 0.08
Guo SB 2012 -2.00 (-3.56, -0.44) 0.08
Liu GL 2004 -2.97 (-3.64, -2.30) 0.45
Subgroup, IV (I² = 83.4%, p = 0.000) -2.08 (-2.23, -1.93) 9.36

**NIDE+MET**		
Pi HC 2020	-3.61 (-4.85, -2.37)	0.13
Ye Q 2020	-1.37 (-1.97, -0.77)	0.55
Zhou C 2019	-2.70 (-3.66, -1.74)	0.22
Yan HP 2019	-5.13 (-6.26, -4.00)	0.16
Zhu FL 2018	-1.50 (-3.29, 0.29)	0.06
Li JP 2018	-0.86 (-1.94, 0.22)	0.17
Gao S 2017	-2.70 (-3.65, -1.75)	0.22
Huang YY 2017	-0.63 (-1.69, 0.43)	0.18
Han J 2017	-1.38 (-3.92, 1.16)	0.03
Hu ZY 2016	-0.58 (-1.54, 0.38)	0.22
Yu YY 2015	-0.90 (-1.65, -0.15)	0.36
Sun ZC 2015	-1.30 (-2.36, -0.24)	0.18
Xu SL 2013	-1.40 (-1.87, -0.93)	0.93
He M 2013A	-2.40 (-3.29, -1.51)	0.25
He M 2013B	-2.20 (-3.11, -1.29)	0.24
Subgroup, IV (I^2 = 81.6%, p = 0.000)	-1.73 (-1.96, -1.50)	3.91
**DPP-4i+MET**		
Hu D 2020	-1.74 (-2.64, -0.84)	0.25
Ding XY 2020	-2.24 (-2.80, -1.68)	0.64
Luo HQ 2020	-2.03 (-3.20, -0.86)	0.15
Zhang XL 2020	-4.17 (-5.07, -3.27)	0.25
Liang YF 2020	-1.70 (-2.50, -0.90)	0.31
Li Y 2020	-3.07 (-4.94, -1.20)	0.06
Sun XO 2020	-3.41 (-5.12, -1.70)	0.07
Xiao BM 2020	-1.76 (-2.67, -0.85)	0.24
Wu SL 2020	-2.49 (-3.35, -1.63)	0.27
Chen J 2020	-1.40 (-2.12, -0.68)	0.38
Niu XP 2020	-2.18 (-3.62, -0.74)	0.10
Yang XQ 2020	-1.38 (-2.37, -0.39)	0.21
Hu B 2020	-2.00 (-2.96, -1.04)	0.22
Liu F 2019	-1.90 (-2.81, -0.99)	0.25
Liu L 2019	-1.77 (-2.31, -1.23)	0.68
Li J 2019	-2.38 (-3.25, -1.51)	0.27
Xie D 2019	-3.00 (-4.17, -1.83)	0.15
Li BA 2019	-2.02 (-3.95, -0.09)	0.05
Wu P 2019	-3.37 (-4.07, -2.67)	0.42
Yang WD 2018	-1.89 (-2.72, -1.06)	0.29
Li HX 2018	-1.93 (-2.52, -1.34)	0.59
Jing HW 2018	-0.63 (-1.73, 0.47)	0.17
Chen L 2018	-1.60 (-3.04, -0.16)	0.10
Chen Z 2018	-2.30 (-3.61, -0.99)	0.12
Li F 2018	-0.65 (-1.26, -0.04)	0.54
Zhou QM 2018	-3.47 (-5.08, -1.86)	0.08
Zhang CJ 2018	-2.75 (-4.35, -1.15)	0.08
Tang Y 2017	-1.20 (-2.18, -0.22)	0.21
Guan XP 2017	-2.69 (-3.55, -1.83)	0.27
Gao HM 2017	-1.97 (-2.84, -1.10)	0.26
Yang N 2017	-1.46 (-2.82, -0.10)	0.11
Zhou Y 2017	-3.44 (-5.16, -1.72)	0.07
Li JJ 2017	-1.00 (-2.00, 0.00)	0.20
Ji LN 2016a	-1.02 (-1.97, -0.07)	0.22
Ji LN 2016b	-1.75 (-2.70, -0.80)	0.22
Yang QM 2016	-1.40 (-2.31, -0.49)	0.24
Hu GH 2016	-1.80 (-2.65, -0.95)	0.28
Wu XH 2016	-2.66 (-3.65, -1.67)	0.21
Yang F 2016	-1.68 (-2.63, -0.73)	0.22
Li LQ 2016	-2.67 (-4.76, -0.58)	0.05
Zhang TT 2016	-1.32 (-2.26, -0.38)	0.23
Han M 2016	-2.63 (-3.49, -1.77)	0.27
Pang XX 2016	-3.21 (-4.84, -1.58)	0.08
Wang XL 2016	-2.00 (-3.19, -0.81)	0.14
Qiao YC 2016	-2.30 (-3.45, -1.15)	0.15
Qi BB 2016	-1.00 (-1.52, -0.48)	0.75
Jiang X 2016	-1.86 (-3.22, -0.50)	0.11
Yao L 2016	-2.13 (-3.46, -0.80)	0.11
Zhang N 2015	-1.60 (-2.30, -0.90)	0.42
Yuan XJ 2015	-3.08 (-4.61, -1.55)	0.09
Zhang HT 2015	-0.85 (-2.29, 0.59)	0.10
Zhuo FT 2015	-3.37 (-4.54, -2.20)	0.15

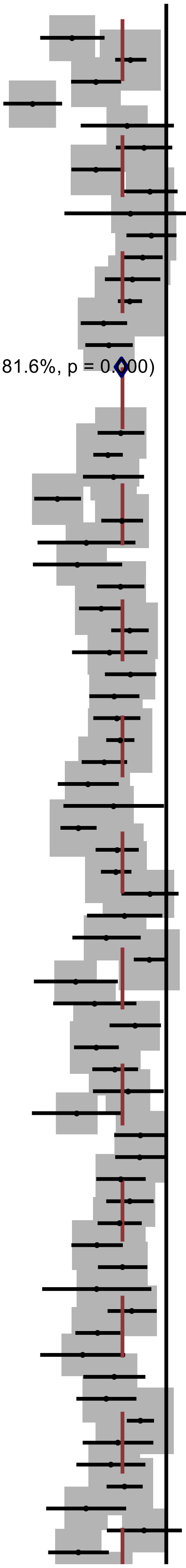

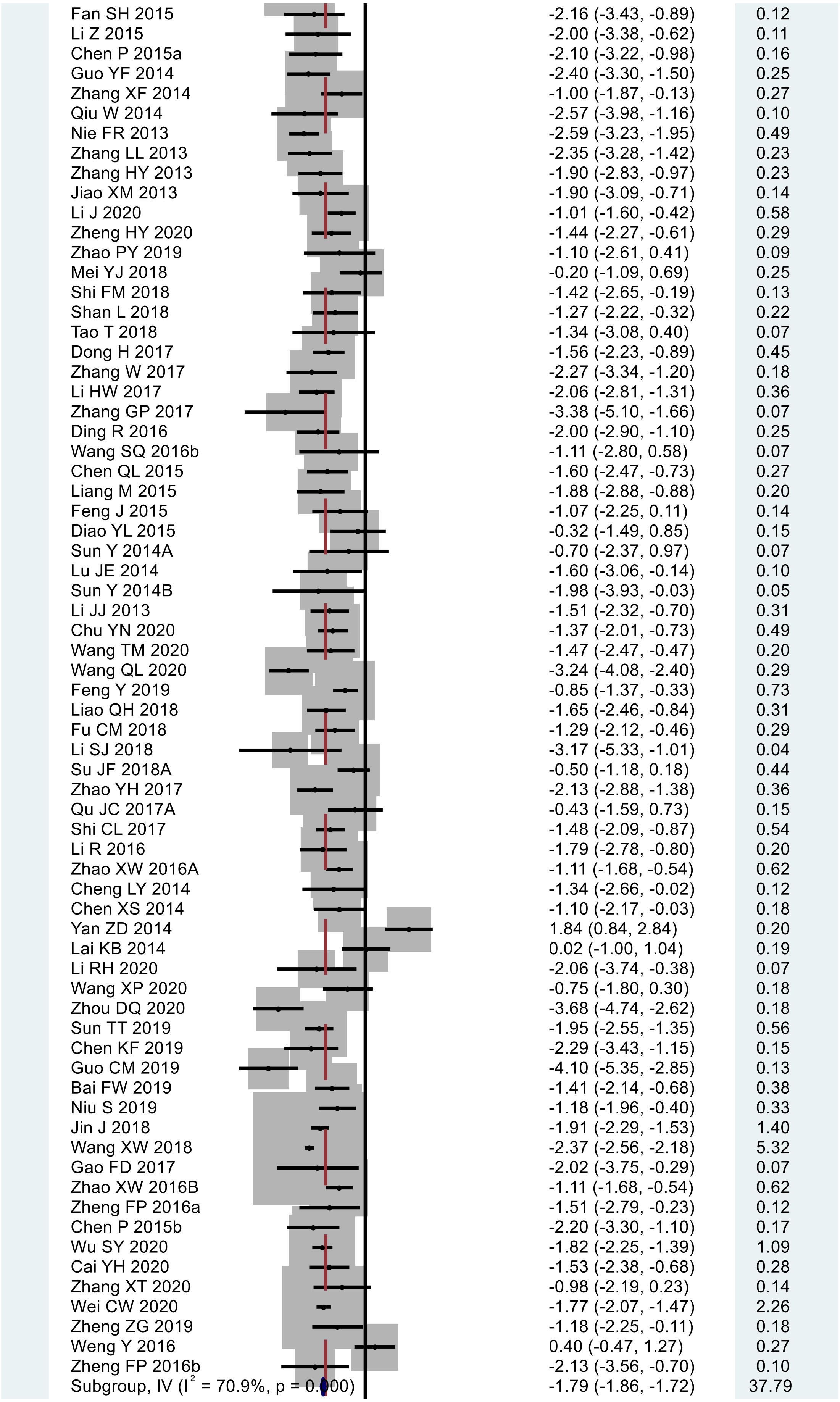
Fan SH 2015 -2.16 (-3.43, -0.89) 0.12
Li Z 2015 -2.00 (-3.38, -0.62) 0.11
Chen P 2015a -2.10 (-3.22, -0.98) 0.16
Guo YF 2014 -2.40 (-3.30, -1.50) 0.25
Zhang XF 2014 -1.00 (-1.87, -0.13) 0.27
Qiu W 2014 -2.57 (-3.98, -1.16) 0.10
Nie FR 2013 -2.59 (-3.23, -1.95) 0.49
Zhang LL 2013 -2.35 (-3.28, -1.42) 0.23
Zhang HY 2013 -1.90 (-2.83, -0.97) 0.23
Jiao XM 2013 -1.90 (-3.09, -0.71) 0.14
Li J 2020 -1.01 (-1.60, -0.42) 0.58
Zheng HY 2020 -1.44 (-2.27, -0.61) 0.29
Zhao PY 2019 -1.10 (-2.61, 0.41) 0.09
Mei YJ 2018 -0.20 (-1.09, 0.69) 0.25
Shi FM 2018 -1.42 (-2.65, -0.19) 0.13
Shan L 2018 -1.27 (-2.22, -0.32) 0.22
Tao T 2018 -1.34 (-3.08, 0.40) 0.07
Dong H 2017 -1.56 (-2.23, -0.89) 0.45
Zhang W 2017 -2.27 (-3.34, -1.20) 0.18
Li HW 2017 -2.06 (-2.81, -1.31) 0.36
Zhang GP 2017 -3.38 (-5.10, -1.66) 0.07
Ding R 2016 -2.00 (-2.90, -1.10) 0.25
Wang SQ 2016b -1.11 (-2.80, 0.58) 0.07
Chen QL 2015 -1.60 (-2.47, -0.73) 0.27
Liang M 2015 -1.88 (-2.88, -0.88) 0.20
Feng J 2015 -1.07 (-2.25, 0.11) 0.14
Diao YL 2015 -0.32 (-1.49, 0.85) 0.15
Sun Y 2014A -0.70 (-2.37, 0.97) 0.07
Lu JE 2014 -1.60 (-3.06, -0.14) 0.10
Sun Y 2014B -1.98 (-3.93, -0.03) 0.05
Li JJ 2013 -1.51 (-2.32, -0.70) 0.31
Chu YN 2020 -1.37 (-2.01, -0.73) 0.49
Wang TM 2020 -1.47 (-2.47, -0.47) 0.20
Wang QL 2020 -3.24 (-4.08, -2.40) 0.29
Feng Y 2019 -0.85 (-1.37, -0.33) 0.73
Liao QH 2018 -1.65 (-2.46, -0.84) 0.31
Fu CM 2018 -1.29 (-2.12, -0.46) 0.29
Li SJ 2018 -3.17 (-5.33, -1.01) 0.04
Su JF 2018A -0.50 (-1.18, 0.18) 0.44
Zhao YH 2017 -2.13 (-2.88, -1.38) 0.36
Qu JC 2017A -0.43 (-1.59, 0.73) 0.15
Shi CL 2017 -1.48 (-2.09, -0.87) 0.54
Li R 2016 -1.79 (-2.78, -0.80) 0.20
Zhao XW 2016A -1.11 (-1.68, -0.54) 0.62
Cheng LY 2014 -1.34 (-2.66, -0.02) 0.12
Chen XS 2014 -1.10 (-2.17, -0.03) 0.18
Yan ZD 2014 1.84 (0.84, 2.84) 0.20
Lai KB 2014 0.02 (-1.00, 1.04) 0.19
Li RH 2020 -2.06 (-3.74, -0.38) 0.07
Wang XP 2020 -0.75 (-1.80, 0.30) 0.18
Zhou DQ 2020 -3.68 (-4.74, -2.62) 0.18
Sun TT 2019 -1.95 (-2.55, -1.35) 0.56
Chen KF 2019 -2.29 (-3.43, -1.15) 0.15
Guo CM 2019 -4.10 (-5.35, -2.85) 0.13
Bai FW 2019 -1.41 (-2.14, -0.68) 0.38
Niu S 2019 -1.18 (-1.96, -0.40) 0.33
Jin J 2018 -1.91 (-2.29, -1.53) 1.40
Wang XW 2018 -2.37 (-2.56, -2.18) 5.32
Gao FD 2017 -2.02 (-3.75, -0.29) 0.07
Zhao XW 2016B -1.11 (-1.68, -0.54) 0.62
Zheng FP 2016a -1.51 (-2.79, -0.23) 0.12
Chen P 2015b -2.20 (-3.30, -1.10) 0.17
Wu SY 2020 -1.82 (-2.25, -1.39) 1.09
Cai YH 2020 -1.53 (-2.38, -0.68) 0.28
Zhang XT 2020 -0.98 (-2.19, 0.23) 0.14
Wei CW 2020 -1.77 (-2.07, -1.47) 2.26
Zheng ZG 2019 -1.18 (-2.25, -0.11) 0.18
Weng Y 2016 0.40 (-0.47, 1.27) 0.27
Zheng FP 2016b -2.13 (-3.56, -0.70) 0.10
Subgroup, IV (I² = 70.9%, p = 0.000) -1.79 (-1.86, -1.72) 37.79

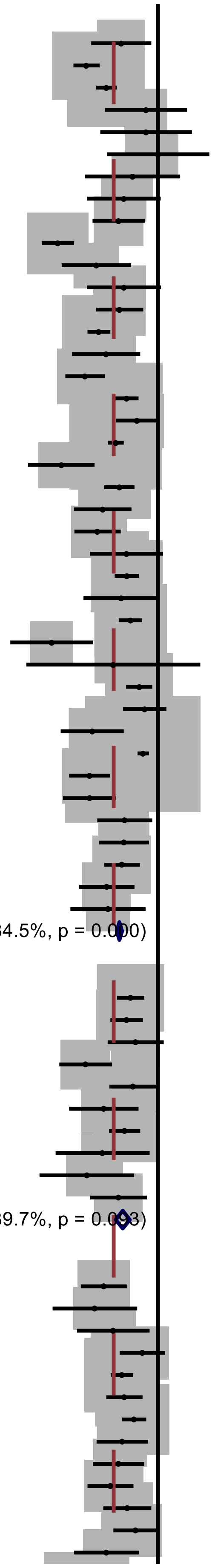

TZD+MET		
Xie LH 2016	-1.40 (-2.54, -0.26)	0.15
Li JY 2014	-2.72 (-3.22, -2.22)	0.82
Lei CY 2014	-1.96 (-2.35, -1.57)	1.30
Yang XD 2013	-0.46 (-2.02, 1.10)	0.08
Yu CY 2013	-0.46 (-2.20, 1.28)	0.07
Gao Y 2013	0.00 (-1.94, 1.94)	0.05
Liu M 2012	-0.97 (-2.77, 0.83)	0.06
Li YG 2011	-1.30 (-2.69, 0.09)	0.10
Li GH 2010	-1.50 (-2.49, -0.51)	0.21
Xia ZX 2009	-3.80 (-4.41, -3.19)	0.55
Zhong FD 2009	-2.34 (-3.65, -1.03)	0.12
Xie XM 2020	-1.30 (-2.70, 0.10)	0.10
Di JN 2020	-1.46 (-2.35, -0.57)	0.25
Cao WF 2020	-2.25 (-2.68, -1.82)	1.07
Shi YQ 2020	-1.97 (-3.26, -0.68)	0.12
Zhang H 2019	-2.77 (-3.52, -2.02)	0.36
Hu KY 2019	-1.20 (-1.65, -0.75)	0.99
Gao SW 2019	-0.80 (-1.61, 0.01)	0.31
Guo H 2018	-1.60 (-1.90, -1.30)	2.23
Chen GM 2018	-3.67 (-4.93, -2.41)	0.13
Ren BX 2018	-1.47 (-2.04, -0.90)	0.61
Li SQ 2016	-2.10 (-3.19, -1.01)	0.17
Zhu XX 2015	-2.30 (-3.18, -1.42)	0.26
Wang T 2015	-1.20 (-2.58, 0.18)	0.11
Ma ZX 2015	-1.19 (-1.65, -0.73)	0.96
Zhou YQ 2015	-1.40 (-2.83, 0.03)	0.10
Zhang CH 2015	-1.05 (-1.49, -0.61)	1.02
Ren YL 2015	-4.03 (-5.60, -2.46)	0.08
Yang HM 2015	-1.70 (-4.99, 1.59)	0.02
Shen CM 2014	-0.72 (-1.22, -0.22)	0.80
Du YJ 2014	-0.51 (-1.33, 0.31)	0.30
Bi XS 2013	-2.50 (-3.70, -1.30)	0.14
Zhong J 2013	-0.57 (-0.79, -0.35)	4.24
Jiang H 2012	-2.60 (-3.37, -1.83)	0.34
Zhu YF 2012	-2.60 (-3.61, -1.59)	0.20
Teng YP 2012	-1.27 (-2.32, -0.22)	0.18
Yang YB 2012B	-1.30 (-2.25, -0.35)	0.22
Guo HF 2011	-1.37 (-2.04, -0.70)	0.44
Hong YJ 2010	-1.95 (-3.00, -0.90)	0.18
Deng JH 2009	-1.90 (-3.32, -0.48)	0.10
Subgroup, IV (I^2 = 84.5%, p = 0.0[illegible]0)	-1.47 (-1.57, -1.37)	19.53
SGLT2i+MET		
Li L 2020	-1.04 (-1.55, -0.53)	0.76
Song QQ 2020	-1.20 (-1.82, -0.58)	0.52
Yang Y 2020	-0.85 (-1.91, 0.21)	0.18
Li JS 2020A	-2.75 (-3.75, -1.75)	0.20
Ren HX 2020	-0.96 (-1.85, -0.07)	0.25
Wang Y 2020A	-2.06 (-3.38, -0.74)	0.12
Liu XJ 2019	-1.27 (-1.86, -0.68)	0.58
Xu J 2019	-2.11 (-3.89, -0.33)	0.06
Liu XY 2020	-2.70 (-4.49, -0.91)	0.06
He ML 2020	-1.50 (-2.58, -0.42)	0.17
Subgroup, IV (I^2 = 39.7%, p = 0.0[illegible]3)	-1.34 (-1.61, -1.08)	2.91
GLP-1RA+MET		
Wei ZX 2018	-2.06 (-2.93, -1.19)	0.26
Hou XL 2018	-2.40 (-4.01, -0.79)	0.08
Zhou RF 2017	-1.70 (-3.07, -0.33)	0.11
Pan NN 2014	-0.60 (-1.46, 0.26)	0.27
Li JS 2020B	-1.37 (-1.80, -0.94)	1.11
Liu YD 2020	-1.28 (-1.97, -0.59)	0.43
Lin Y 2020	-0.92 (-1.38, -0.46)	0.94
Yang M 2020	-1.36 (-2.33, -0.39)	0.21
Jia C 2020	-1.50 (-2.48, -0.52)	0.21
Liu WF 2020	-1.81 (-2.68, -0.94)	0.27
Liu HY 2020	-1.17 (-2.08, -0.26)	0.24
Wang YQ 2019	-0.86 (-1.70, -0.02)	0.28
Li LB 2019	-1.96 (-3.19, -0.73)	0.13

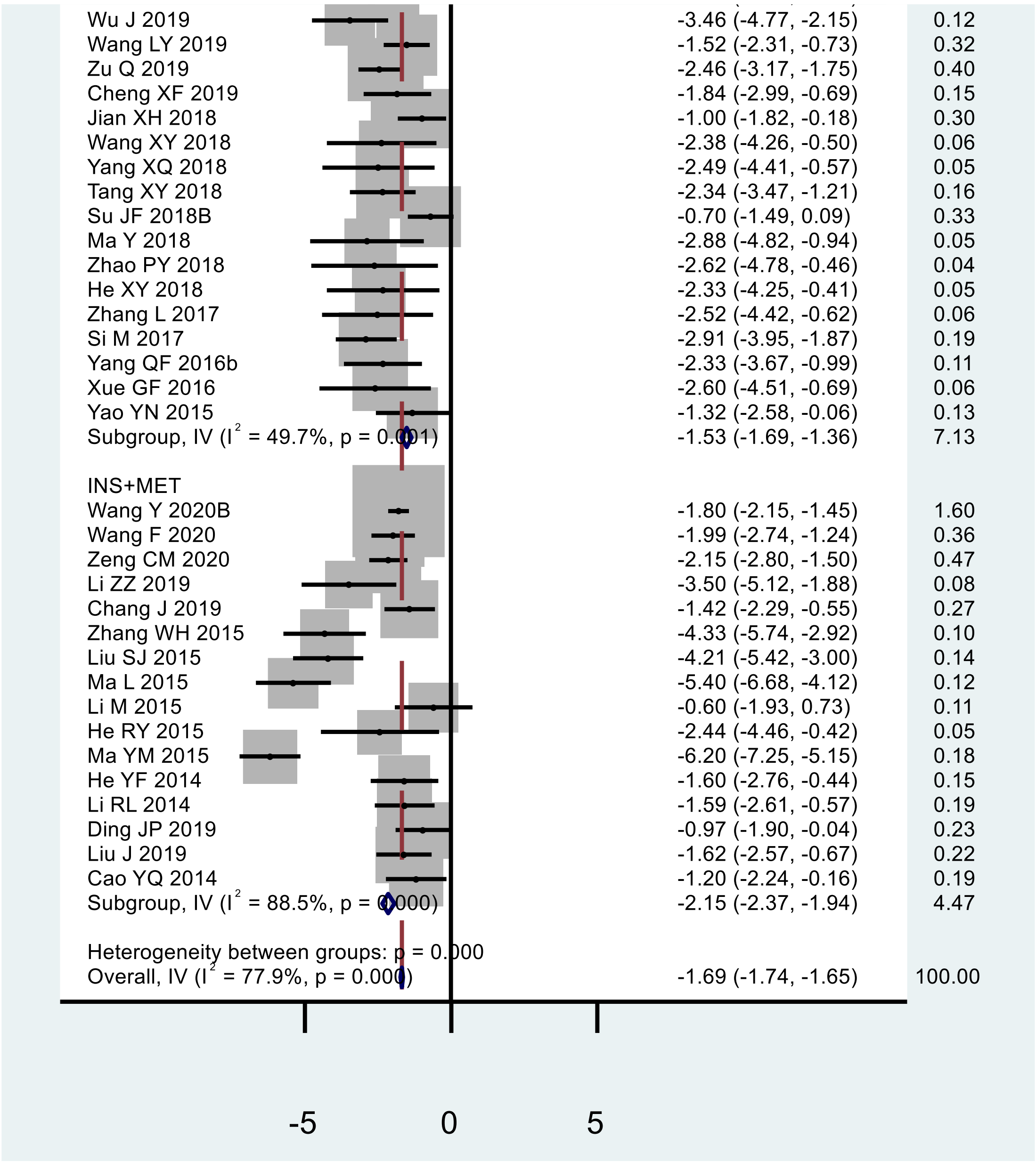

Figure S63. Sensitivity analysis results for change in 2h postprandial plasma glucose (mmol/l) of glucose-lowering drugs added to metformin compared with metformin monotherapy

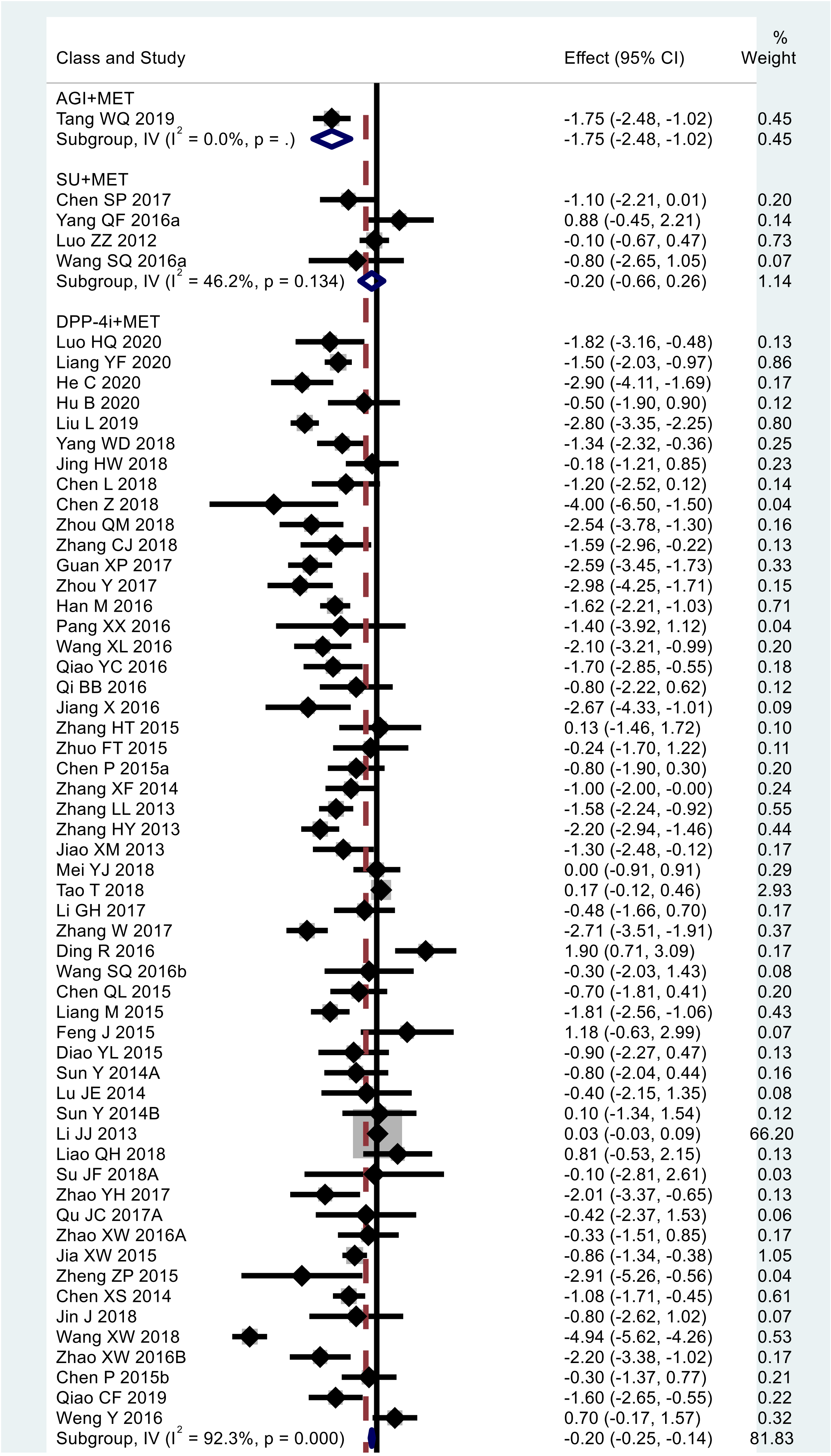
Class and Study
Effect (95% CI)
% Weight
AGI+MET
Tang WQ 2019 -1.75 (-2.48, -1.02) 0.45
Subgroup, IV (I^2 = 0.0%, p = .) -1.75 (-2.48, -1.02) 0.45
SU+MET
Chen SP 2017 -1.10 (-2.21, 0.01) 0.20
Yang QF 2016a 0.88 (-0.45, 2.21) 0.14
Luo ZZ 2012 -0.10 (-0.67, 0.47) 0.73
Wang SQ 2016a -0.80 (-2.65, 1.05) 0.07
Subgroup, IV (I^2 = 46.2%, p = 0.134) -0.20 (-0.66, 0.26) 1.14
DPP-4i+MET
Luo HQ 2020 -1.82 (-3.16, -0.48) 0.13
Liang YF 2020 -1.50 (-2.03, -0.97) 0.86
He C 2020 -2.90 (-4.11, -1.69) 0.17
Hu B 2020 -0.50 (-1.90, 0.90) 0.12
Liu L 2019 -2.80 (-3.35, -2.25) 0.80
Yang WD 2018 -1.34 (-2.32, -0.36) 0.25
Jing HW 2018 -0.18 (-1.21, 0.85) 0.23
Chen L 2018 -1.20 (-2.52, 0.12) 0.14
Chen Z 2018 -4.00 (-6.50, -1.50) 0.04
Zhou QM 2018 -2.54 (-3.78, -1.30) 0.16
Zhang CJ 2018 -1.59 (-2.96, -0.22) 0.13
Guan XP 2017 -2.59 (-3.45, -1.73) 0.33
Zhou Y 2017 -2.98 (-4.25, -1.71) 0.15
Han M 2016 -1.62 (-2.21, -1.03) 0.71
Pang XX 2016 -1.40 (-3.92, 1.12) 0.04
Wang XL 2016 -2.10 (-3.21, -0.99) 0.20
Qiao YC 2016 -1.70 (-2.85, -0.55) 0.18
Qi BB 2016 -0.80 (-2.22, 0.62) 0.12
Jiang X 2016 -2.67 (-4.33, -1.01) 0.09
Zhang HT 2015 0.13 (-1.46, 1.72) 0.10
Zhuo FT 2015 -0.24 (-1.70, 1.22) 0.11
Chen P 2015a -0.80 (-1.90, 0.30) 0.20
Zhang XF 2014 -1.00 (-2.00, -0.00) 0.24
Zhang LL 2013 -1.58 (-2.24, -0.92) 0.55
Zhang HY 2013 -2.20 (-2.94, -1.46) 0.44
Jiao XM 2013 -1.30 (-2.48, -0.12) 0.17
Mei YJ 2018 0.00 (-0.91, 0.91) 0.29
Tao T 2018 0.17 (-0.12, 0.46) 2.93
Li GH 2017 -0.48 (-1.66, 0.70) 0.17
Zhang W 2017 -2.71 (-3.51, -1.91) 0.37
Ding R 2016 1.90 (0.71, 3.09) 0.17
Wang SQ 2016b -0.30 (-2.03, 1.43) 0.08
Chen QL 2015 -0.70 (-1.81, 0.41) 0.20
Liang M 2015 -1.81 (-2.56, -1.06) 0.43
Feng J 2015 1.18 (-0.63, 2.99) 0.07
Diao YL 2015 -0.90 (-2.27, 0.47) 0.13
Sun Y 2014A -0.80 (-2.04, 0.44) 0.16
Lu JE 2014 -0.40 (-2.15, 1.35) 0.08
Sun Y 2014B 0.10 (-1.34, 1.54) 0.12
Li JJ 2013 0.03 (-0.03, 0.09) 66.20
Liao QH 2018 0.81 (-0.53, 2.15) 0.13
Su JF 2018A -0.10 (-2.81, 2.61) 0.03
Zhao YH 2017 -2.01 (-3.37, -0.65) 0.13
Qu JC 2017A -0.42 (-2.37, 1.53) 0.06
Zhao XW 2016A -0.33 (-1.51, 0.85) 0.17
Jia XW 2015 -0.86 (-1.34, -0.38) 1.05
Zheng ZP 2015 -2.91 (-5.26, -0.56) 0.04
Chen XS 2014 -1.08 (-1.71, -0.45) 0.61
Jin J 2018 -0.80 (-2.62, 1.02) 0.07
Wang XW 2018 -4.94 (-5.62, -4.26) 0.53
Zhao XW 2016B -2.20 (-3.38, -1.02) 0.17
Chen P 2015b -0.30 (-1.37, 0.77) 0.21
Qiao CF 2019 -1.60 (-2.65, -0.55) 0.22
Weng Y 2016 0.70 (-0.17, 1.57) 0.32
Subgroup, IV (I^2 = 92.3%, p = 0.000) -0.20 (-0.25, -0.14) 81.83

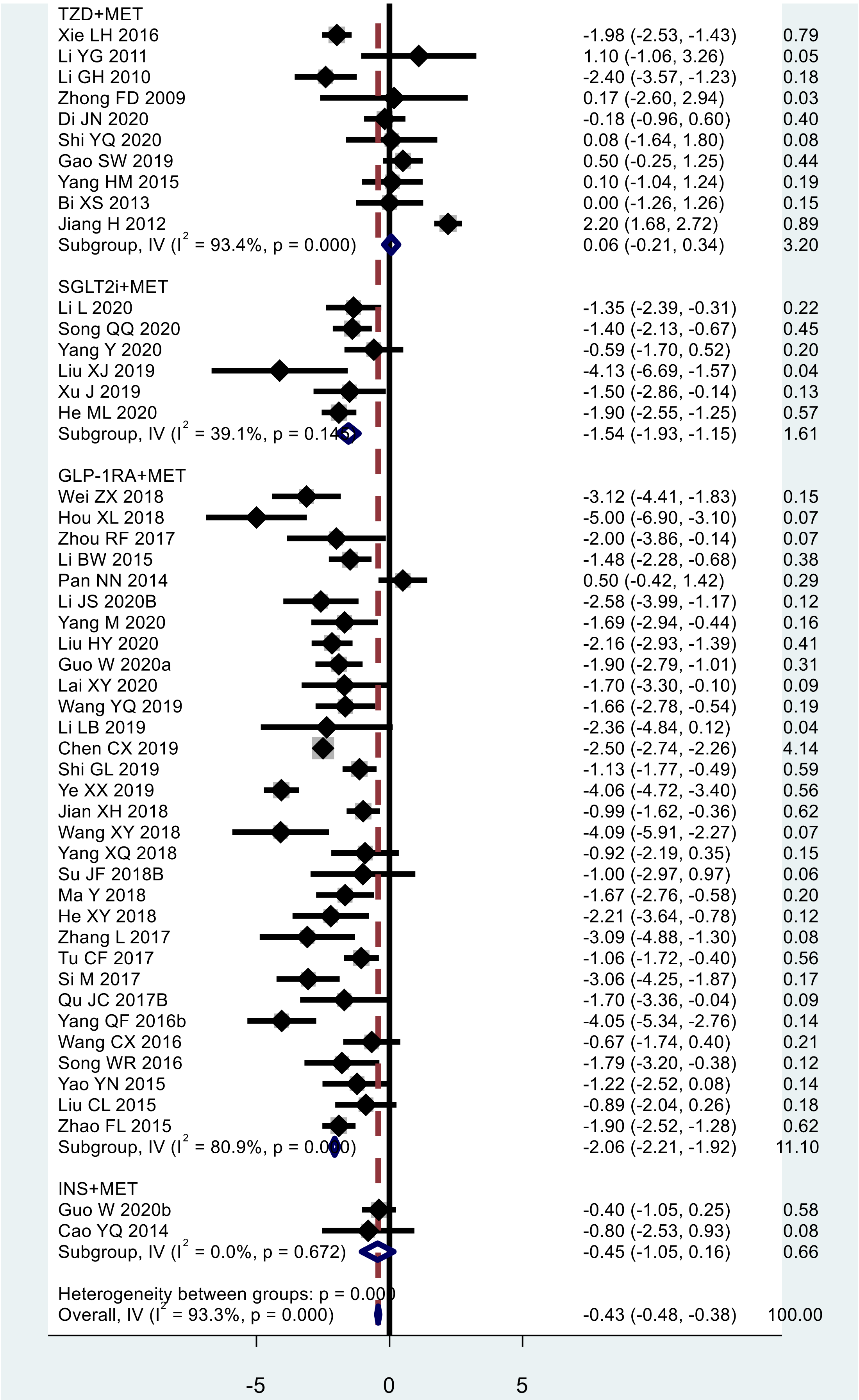

Figure S64. Sensitivity analysis results for change in body mass index (kg/m^2) of glucose-lowering drugs added to metformin compared with metformin monotherapy

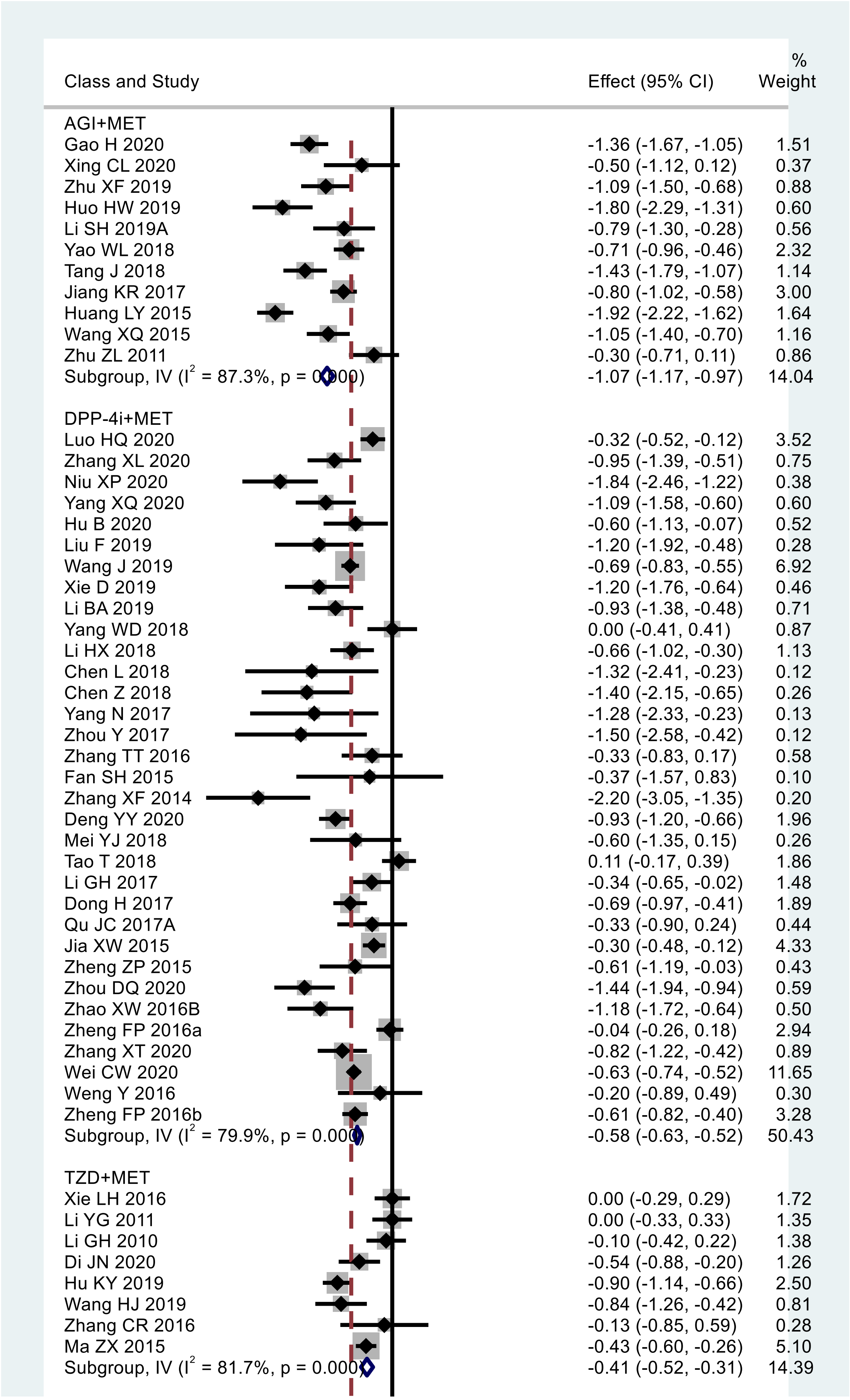
Class and Study
Effect (95% CI)
% Weight
AGI+MET
Gao H 2020 -1.36 (-1.67, -1.05) 1.51
Xing CL 2020 -0.50 (-1.12, 0.12) 0.37
Zhu XF 2019 -1.09 (-1.50, -0.68) 0.88
Huo HW 2019 -1.80 (-2.29, -1.31) 0.60
Li SH 2019A -0.79 (-1.30, -0.28) 0.56
Yao WL 2018 -0.71 (-0.96, -0.46) 2.32
Tang J 2018 -1.43 (-1.79, -1.07) 1.14
Jiang KR 2017 -0.80 (-1.02, -0.58) 3.00
Huang LY 2015 -1.92 (-2.22, -1.62) 1.64
Wang XQ 2015 -1.05 (-1.40, -0.70) 1.16
Zhu ZL 2011 -0.30 (-0.71, 0.11) 0.86
Subgroup, IV (I^2 = 87.3%, p = 0.000) -1.07 (-1.17, -0.97) 14.04
DPP-4i+MET
Luo HQ 2020 -0.32 (-0.52, -0.12) 3.52
Zhang XL 2020 -0.95 (-1.39, -0.51) 0.75
Niu XP 2020 -1.84 (-2.46, -1.22) 0.38
Yang XQ 2020 -1.09 (-1.58, -0.60) 0.60
Hu B 2020 -0.60 (-1.13, -0.07) 0.52
Liu F 2019 -1.20 (-1.92, -0.48) 0.28
Wang J 2019 -0.69 (-0.83, -0.55) 6.92
Xie D 2019 -1.20 (-1.76, -0.64) 0.46
Li BA 2019 -0.93 (-1.38, -0.48) 0.71
Yang WD 2018 0.00 (-0.41, 0.41) 0.87
Li HX 2018 -0.66 (-1.02, -0.30) 1.13
Chen L 2018 -1.32 (-2.41, -0.23) 0.12
Chen Z 2018 -1.40 (-2.15, -0.65) 0.26
Yang N 2017 -1.28 (-2.33, -0.23) 0.13
Zhou Y 2017 -1.50 (-2.58, -0.42) 0.12
Zhang TT 2016 -0.33 (-0.83, 0.17) 0.58
Fan SH 2015 -0.37 (-1.57, 0.83) 0.10
Zhang XF 2014 -2.20 (-3.05, -1.35) 0.20
Deng YY 2020 -0.93 (-1.20, -0.66) 1.96
Mei YJ 2018 -0.60 (-1.35, 0.15) 0.26
Tao T 2018 0.11 (-0.17, 0.39) 1.86
Li GH 2017 -0.34 (-0.65, -0.02) 1.48
Dong H 2017 -0.69 (-0.97, -0.41) 1.89
Qu JC 2017A -0.33 (-0.90, 0.24) 0.44
Jia XW 2015 -0.30 (-0.48, -0.12) 4.33
Zheng ZP 2015 -0.61 (-1.19, -0.03) 0.43
Zhou DQ 2020 -1.44 (-1.94, -0.94) 0.59
Zhao XW 2016B -1.18 (-1.72, -0.64) 0.50
Zheng FP 2016a -0.04 (-0.26, 0.18) 2.94
Zhang XT 2020 -0.82 (-1.22, -0.42) 0.89
Wei CW 2020 -0.63 (-0.74, -0.52) 11.65
Weng Y 2016 -0.20 (-0.89, 0.49) 0.30
Zheng FP 2016b -0.61 (-0.82, -0.40) 3.28
Subgroup, IV (I^2 = 79.9%, p = 0.000) -0.58 (-0.63, -0.52) 50.43
TZD+MET
Xie LH 2016 0.00 (-0.29, 0.29) 1.72
Li YG 2011 0.00 (-0.33, 0.33) 1.35
Li GH 2010 -0.10 (-0.42, 0.22) 1.38
Di JN 2020 -0.54 (-0.88, -0.20) 1.26
Hu KY 2019 -0.90 (-1.14, -0.66) 2.50
Wang HJ 2019 -0.84 (-1.26, -0.42) 0.81
Zhang CR 2016 -0.13 (-0.85, 0.59) 0.28
Ma ZX 2015 -0.43 (-0.60, -0.26) 5.10
Subgroup, IV (I^2 = 81.7%, p = 0.000) -0.41 (-0.52, -0.31) 14.39

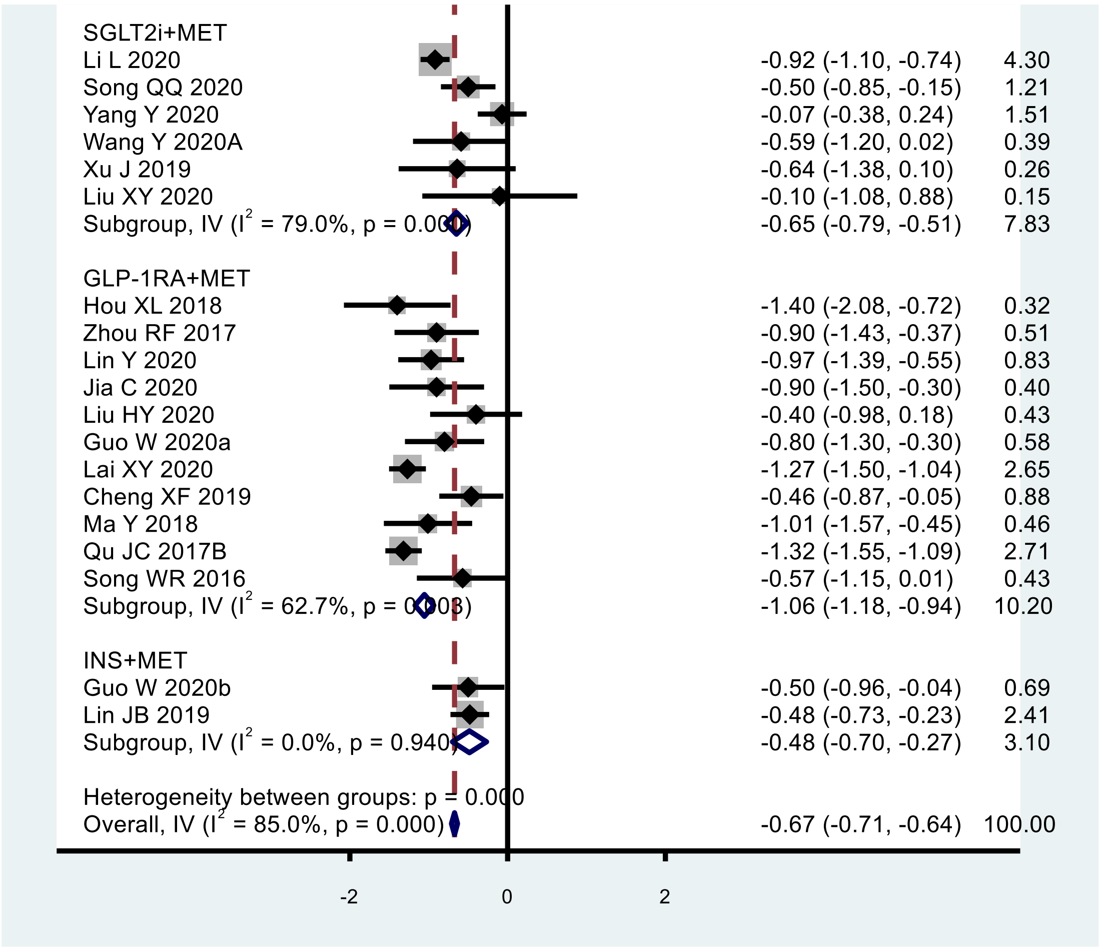

Figure S65. Sensitivity analysis results for change in total cholesterol (mmol/l) of glucose-lowering drugs added to metformin compared with metformin monotherapy

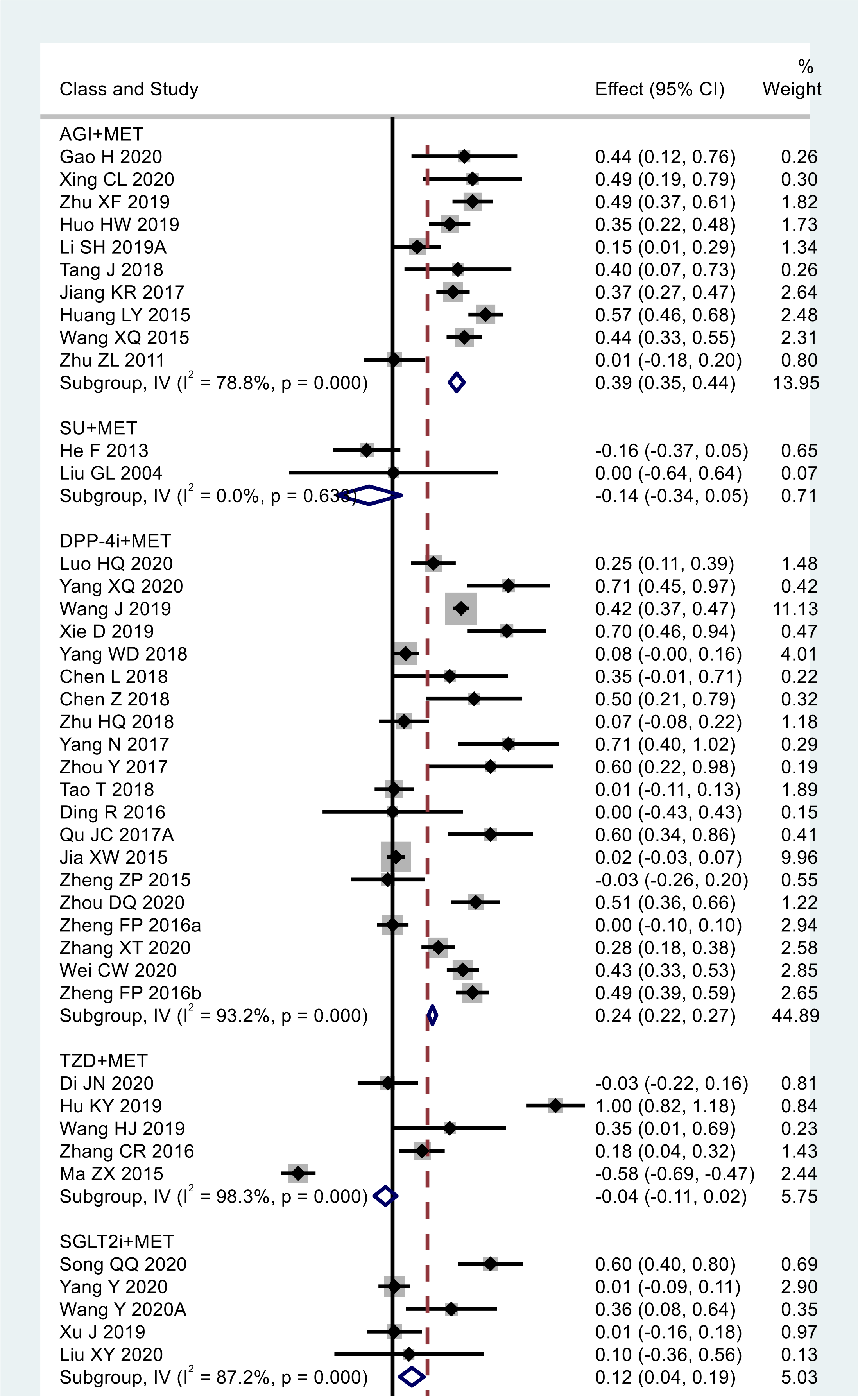
Class and Study
Effect (95% CI)
% Weight
AGI+MET
Gao H 2020 0.44 (0.12, 0.76) 0.26
Xing CL 2020 0.49 (0.19, 0.79) 0.30
Zhu XF 2019 0.49 (0.37, 0.61) 1.82
Huo HW 2019 0.35 (0.22, 0.48) 1.73
Li SH 2019A 0.15 (0.01, 0.29) 1.34
Tang J 2018 0.40 (0.07, 0.73) 0.26
Jiang KR 2017 0.37 (0.27, 0.47) 2.64
Huang LY 2015 0.57 (0.46, 0.68) 2.48
Wang XQ 2015 0.44 (0.33, 0.55) 2.31
Zhu ZL 2011 0.01 (-0.18, 0.20) 0.80
Subgroup, IV (I² = 78.8%, p = 0.000) 0.39 (0.35, 0.44) 13.95
SU+MET
He F 2013 -0.16 (-0.37, 0.05) 0.65
Liu GL 2004 0.00 (-0.64, 0.64) 0.07
Subgroup, IV (I² = 0.0%, p = 0.636) -0.14 (-0.34, 0.05) 0.71
DPP-4i+MET
Luo HQ 2020 0.25 (0.11, 0.39) 1.48
Yang XQ 2020 0.71 (0.45, 0.97) 0.42
Wang J 2019 0.42 (0.37, 0.47) 11.13
Xie D 2019 0.70 (0.46, 0.94) 0.47
Yang WD 2018 0.08 (-0.00, 0.16) 4.01
Chen L 2018 0.35 (-0.01, 0.71) 0.22
Chen Z 2018 0.50 (0.21, 0.79) 0.32
Zhu HQ 2018 0.07 (-0.08, 0.22) 1.18
Yang N 2017 0.71 (0.40, 1.02) 0.29
Zhou Y 2017 0.60 (0.22, 0.98) 0.19
Tao T 2018 0.01 (-0.11, 0.13) 1.89
Ding R 2016 0.00 (-0.43, 0.43) 0.15
Qu JC 2017A 0.60 (0.34, 0.86) 0.41
Jia XW 2015 0.02 (-0.03, 0.07) 9.96
Zheng ZP 2015 -0.03 (-0.26, 0.20) 0.55
Zhou DQ 2020 0.51 (0.36, 0.66) 1.22
Zheng FP 2016a 0.00 (-0.10, 0.10) 2.94
Zhang XT 2020 0.28 (0.18, 0.38) 2.58
Wei CW 2020 0.43 (0.33, 0.53) 2.85
Zheng FP 2016b 0.49 (0.39, 0.59) 2.65
Subgroup, IV (I² = 93.2%, p = 0.000) 0.24 (0.22, 0.27) 44.89
TZD+MET
Di JN 2020 -0.03 (-0.22, 0.16) 0.81
Hu KY 2019 1.00 (0.82, 1.18) 0.84
Wang HJ 2019 0.35 (0.01, 0.69) 0.23
Zhang CR 2016 0.18 (0.04, 0.32) 1.43
Ma ZX 2015 -0.58 (-0.69, -0.47) 2.44
Subgroup, IV (I² = 98.3%, p = 0.000) -0.04 (-0.11, 0.02) 5.75
SGLT2i+MET
Song QQ 2020 0.60 (0.40, 0.80) 0.69
Yang Y 2020 0.01 (-0.09, 0.11) 2.90
Wang Y 2020A 0.36 (0.08, 0.64) 0.35
Xu J 2019 0.01 (-0.16, 0.18) 0.97
Liu XY 2020 0.10 (-0.36, 0.56) 0.13
Subgroup, IV (I² = 87.2%, p = 0.000) 0.12 (0.04, 0.19) 5.03

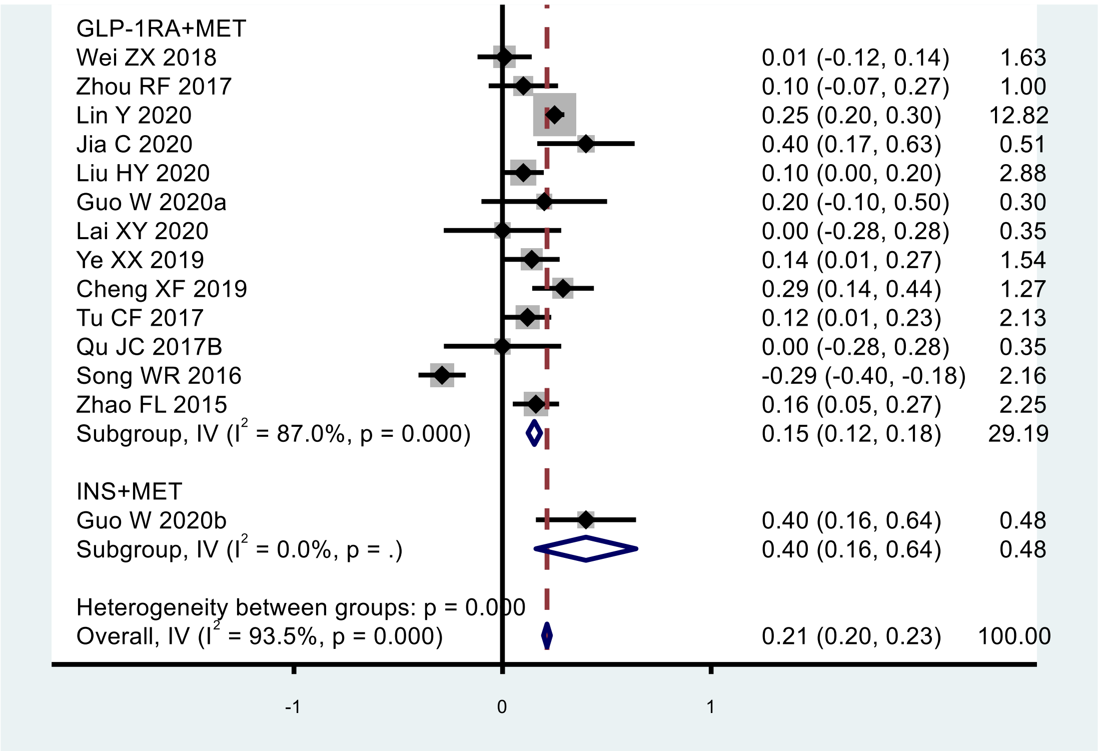

Figure S66. Sensitivity analysis results for change in high density lipoprotein-cholesterol (mmol/l) of glucose-lowering drugs added to metformin compared with metformin monotherapy

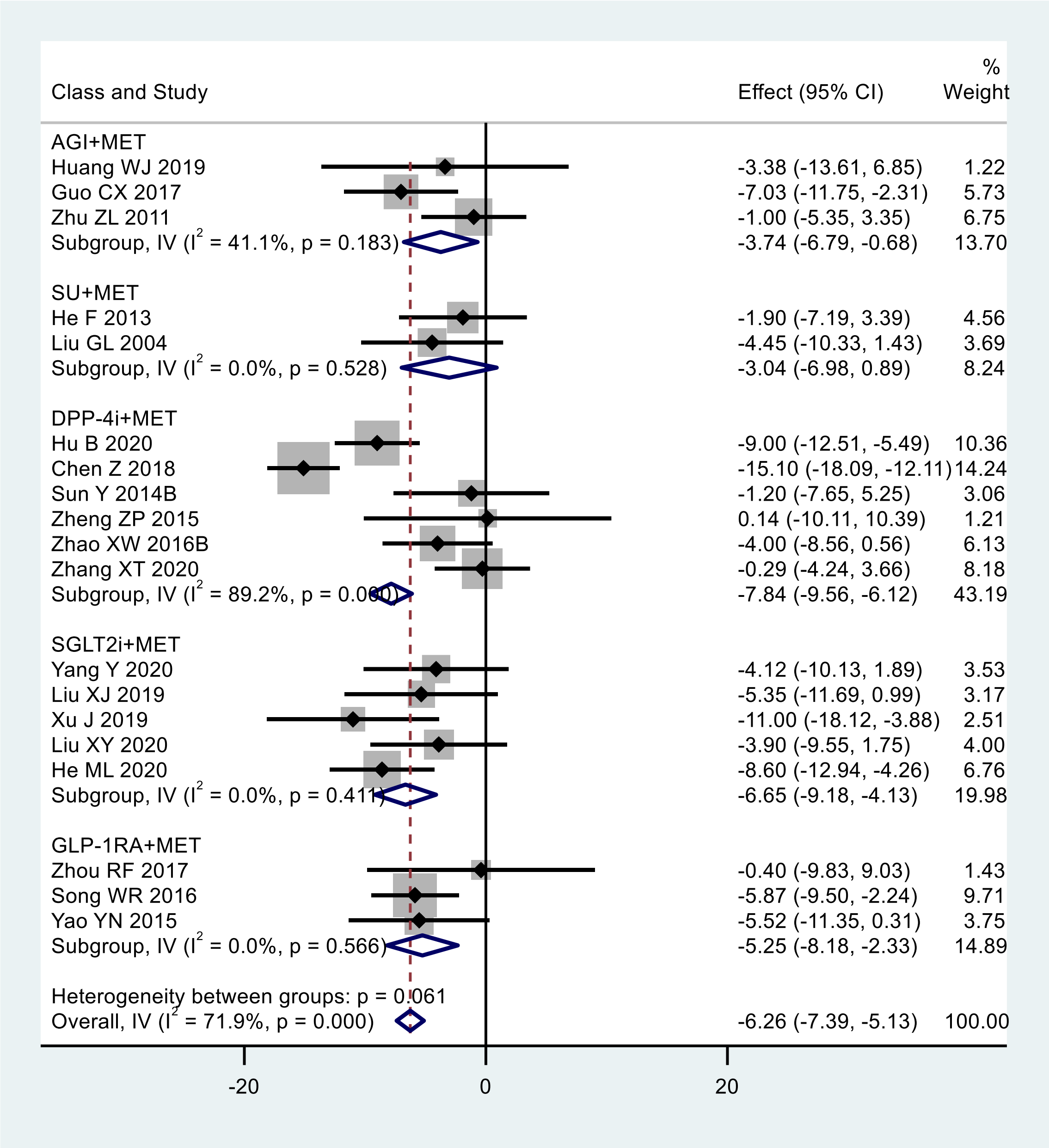

Figure S67. Sensitivity analysis results for change in systolic blood pressure (mmHg) of glucose-lowering drugs added to metformin compared with metformin monotherapy

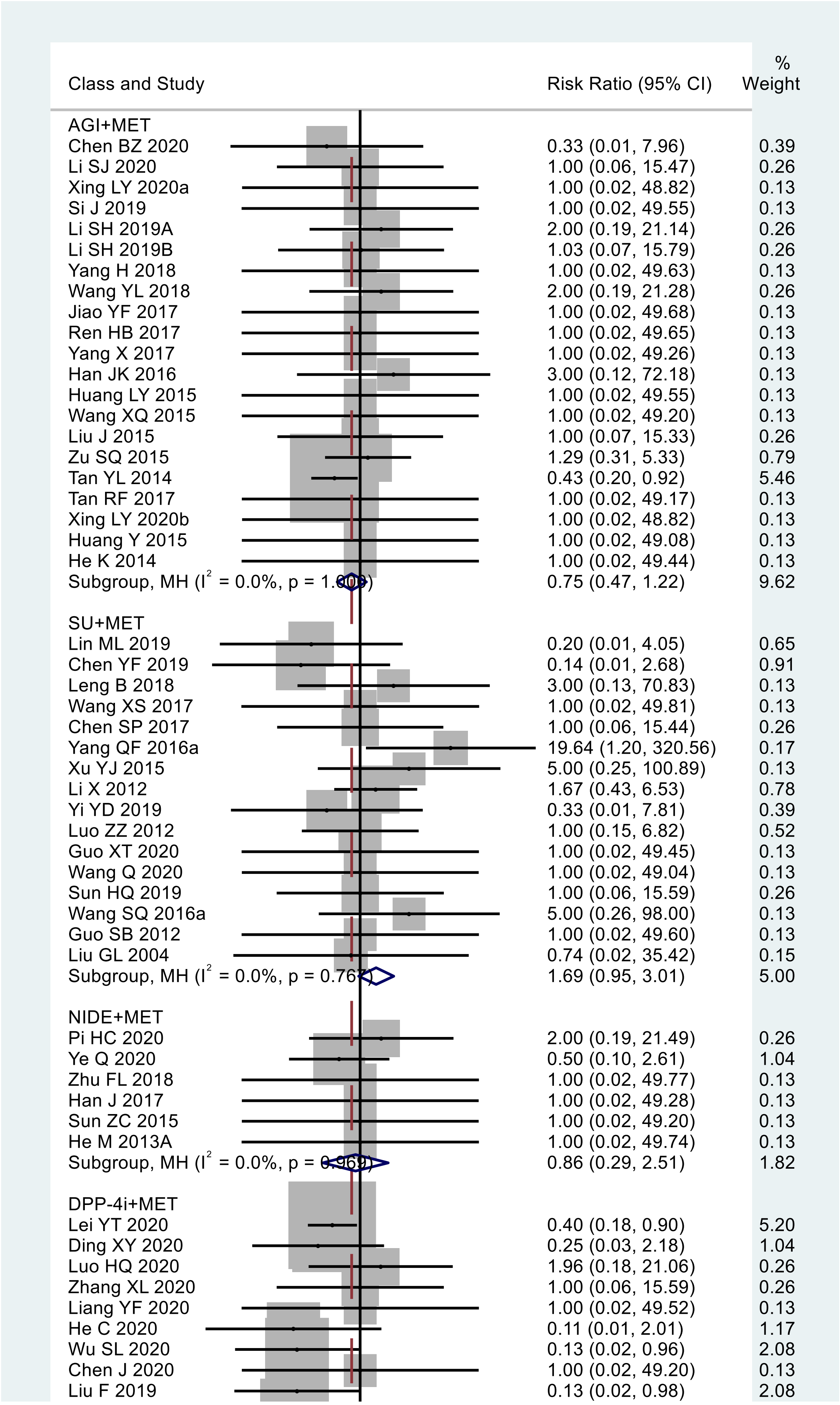

%
Class and Study
Risk Ratio (95% CI)
Weight
AGI+MET
Chen BZ 2020 0.33 (0.01, 7.96) 0.39
Li SJ 2020 1.00 (0.06, 15.47) 0.26
Xing LY 2020a 1.00 (0.02, 48.82) 0.13
Si J 2019 1.00 (0.02, 49.55) 0.13
Li SH 2019A 2.00 (0.19, 21.14) 0.26
Li SH 2019B 1.03 (0.07, 15.79) 0.26
Yang H 2018 1.00 (0.02, 49.63) 0.13
Wang YL 2018 2.00 (0.19, 21.28) 0.26
Jiao YF 2017 1.00 (0.02, 49.68) 0.13
Ren HB 2017 1.00 (0.02, 49.65) 0.13
Yang X 2017 1.00 (0.02, 49.26) 0.13
Han JK 2016 3.00 (0.12, 72.18) 0.13
Huang LY 2015 1.00 (0.02, 49.55) 0.13
Wang XQ 2015 1.00 (0.02, 49.20) 0.13
Liu J 2015 1.00 (0.07, 15.33) 0.26
Zu SQ 2015 1.29 (0.31, 5.33) 0.79
Tan YL 2014 0.43 (0.20, 0.92) 5.46
Tan RF 2017 1.00 (0.02, 49.17) 0.13
Xing LY 2020b 1.00 (0.02, 48.82) 0.13
Huang Y 2015 1.00 (0.02, 49.08) 0.13
He K 2014 1.00 (0.02, 49.44) 0.13
Subgroup, MH (I^2 = 0.0%, p = 1.000) 0.75 (0.47, 1.22) 9.62
SU+MET
Lin ML 2019 0.20 (0.01, 4.05) 0.65
Chen YF 2019 0.14 (0.01, 2.68) 0.91
Leng B 2018 3.00 (0.13, 70.83) 0.13
Wang XS 2017 1.00 (0.02, 49.81) 0.13
Chen SP 2017 1.00 (0.06, 15.44) 0.26
Yang QF 2016a 19.64 (1.20, 320.56) 0.17
Xu YJ 2015 5.00 (0.25, 100.89) 0.13
Li X 2012 1.67 (0.43, 6.53) 0.78
Yi YD 2019 0.33 (0.01, 7.81) 0.39
Luo ZZ 2012 1.00 (0.15, 6.82) 0.52
Guo XT 2020 1.00 (0.02, 49.45) 0.13
Wang Q 2020 1.00 (0.02, 49.04) 0.13
Sun HQ 2019 1.00 (0.06, 15.59) 0.26
Wang SQ 2016a 5.00 (0.26, 98.00) 0.13
Guo SB 2012 1.00 (0.02, 49.60) 0.13
Liu GL 2004 0.74 (0.02, 35.42) 0.15
Subgroup, MH (I^2 = 0.0%, p = 0.767) 1.69 (0.95, 3.01) 5.00
NIDE+MET
Pi HC 2020 2.00 (0.19, 21.49) 0.26
Ye Q 2020 0.50 (0.10, 2.61) 1.04
Zhu FL 2018 1.00 (0.02, 49.77) 0.13
Han J 2017 1.00 (0.02, 49.28) 0.13
Sun ZC 2015 1.00 (0.02, 49.20) 0.13
He M 2013A 1.00 (0.02, 49.74) 0.13
Subgroup, MH (I^2 = 0.0%, p = 0.969) 0.86 (0.29, 2.51) 1.82
DPP-4i+MET
Lei YT 2020 0.40 (0.18, 0.90) 5.20
Ding XY 2020 0.25 (0.03, 2.18) 1.04
Luo HQ 2020 1.96 (0.18, 21.06) 0.26
Zhang XL 2020 1.00 (0.06, 15.59) 0.26
Liang YF 2020 1.00 (0.02, 49.52) 0.13
He C 2020 0.11 (0.01, 2.01) 1.17
Wu SL 2020 0.13 (0.02, 0.96) 2.08
Chen J 2020 1.00 (0.02, 49.20) 0.13
Liu F 2019 0.13 (0.02, 0.98) 2.08

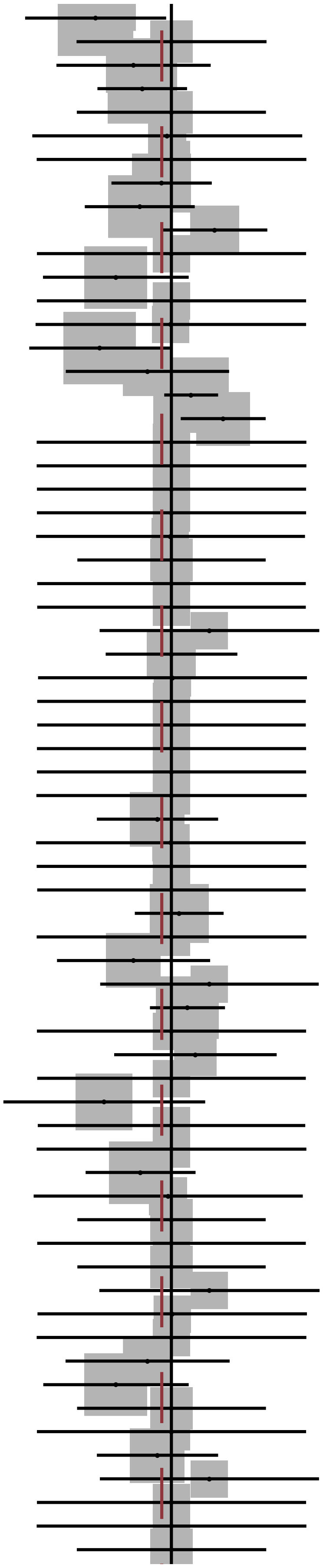

Liu L 2019	0.11 (0.01, 0.86)	2.34
Wang J 2019	1.00 (0.06, 15.67)	0.26
Li J 2019	0.33 (0.04, 3.11)	0.78
Li BA 2019	0.43 (0.12, 1.57)	1.82
Wu P 2019	1.00 (0.06, 15.45)	0.26
Yang WD 2018	0.88 (0.02, 43.88)	0.14
Li HX 2018	1.00 (0.02, 49.59)	0.13
Jing HW 2018	0.75 (0.18, 3.21)	1.04
Chen L 2018	0.40 (0.08, 1.97)	1.30
Chen Z 2018	3.50 (0.76, 16.01)	0.52
Li F 2018	1.00 (0.02, 49.38)	0.13
Zhou QM 2018	0.20 (0.02, 1.65)	1.30
Zhu HQ 2018	1.00 (0.02, 49.00)	0.13
Tang Y 2017	0.98 (0.02, 49.04)	0.13
Zhou Y 2017	0.13 (0.02, 0.96)	2.08
Li JJ 2017	0.50 (0.05, 5.31)	0.52
Ji LN 2016a	1.76 (0.81, 3.84)	2.35
Ji LN 2016b	4.48 (1.31, 15.32)	0.77
Yang QM 2016	1.00 (0.02, 49.42)	0.13
Hu GH 2016	1.00 (0.02, 49.40)	0.13
Zhang TT 2016	1.00 (0.02, 49.23)	0.13
Han M 2016	1.00 (0.02, 49.00)	0.13
Pang XX 2016	0.97 (0.02, 47.50)	0.13
Qi BB 2016	1.00 (0.07, 15.26)	0.26
Jiang X 2016	1.00 (0.02, 48.82)	0.13
Yao L 2016	1.00 (0.02, 48.82)	0.13
Zhang N 2015	3.00 (0.12, 72.25)	0.13
Zhang HT 2015	1.00 (0.15, 6.71)	0.52
Zhuo FT 2015	1.03 (0.02, 50.42)	0.13
Li Z 2015	1.00 (0.02, 48.87)	0.13
Chen P 2015a	1.00 (0.02, 48.82)	0.13
Guo YF 2014	1.00 (0.02, 49.33)	0.13
Qiu W 2014	1.00 (0.02, 49.20)	0.13
Nie FR 2013	1.00 (0.02, 49.79)	0.13
Li WM 2013	0.67 (0.11, 3.87)	0.78
Zhang LL 2013	0.98 (0.02, 48.69)	0.13
Zhang HY 2013	1.00 (0.02, 49.40)	0.13
Jiao XM 2013	1.00 (0.02, 48.82)	0.13
Li J 2020	1.25 (0.35, 4.52)	1.04
Deng YY 2020	1.00 (0.02, 49.45)	0.13
Zheng HY 2020	0.33 (0.04, 3.07)	0.78
Shan L 2018	3.00 (0.13, 71.22)	0.13
Li GH 2017	1.58 (0.54, 4.69)	1.31
Zhang W 2017	1.00 (0.02, 49.26)	0.13
Li HW 2017	2.00 (0.19, 20.97)	0.26
Zhang GP 2017	1.00 (0.02, 48.82)	0.13
Ding R 2016	0.14 (0.01, 2.65)	0.91
Wang SQ 2016b	1.00 (0.02, 48.09)	0.13
Feng J 2015	1.00 (0.02, 49.40)	0.13
Diao YL 2015	0.41 (0.08, 2.00)	1.29
Sun Y 2014A	0.91 (0.02, 44.88)	0.14
Lu JE 2014	1.00 (0.07, 15.26)	0.26
Sun Y 2014B	1.00 (0.02, 48.82)	0.13
Li JJ 2013	1.00 (0.07, 15.33)	0.26
Chu YN 2020	3.00 (0.12, 72.56)	0.13
Wang TM 2020	1.02 (0.02, 50.41)	0.13
Liao QH 2018	1.00 (0.02, 49.75)	0.13
Fu CM 2018	0.50 (0.05, 5.38)	0.52
Peng XH 2018	0.20 (0.02, 1.64)	1.30
Li SJ 2018	1.00 (0.06, 15.41)	0.26
Su JF 2018A	1.00 (0.02, 49.08)	0.13
Zhao YH 2017	0.67 (0.12, 3.85)	0.78
Qu JC 2017A	3.00 (0.13, 71.51)	0.13
Shi CL 2017	1.00 (0.02, 49.14)	0.13
Li R 2016	1.00 (0.02, 49.44)	0.13
Zhao XW 2016A	1.00 (0.06, 15.55)	0.26

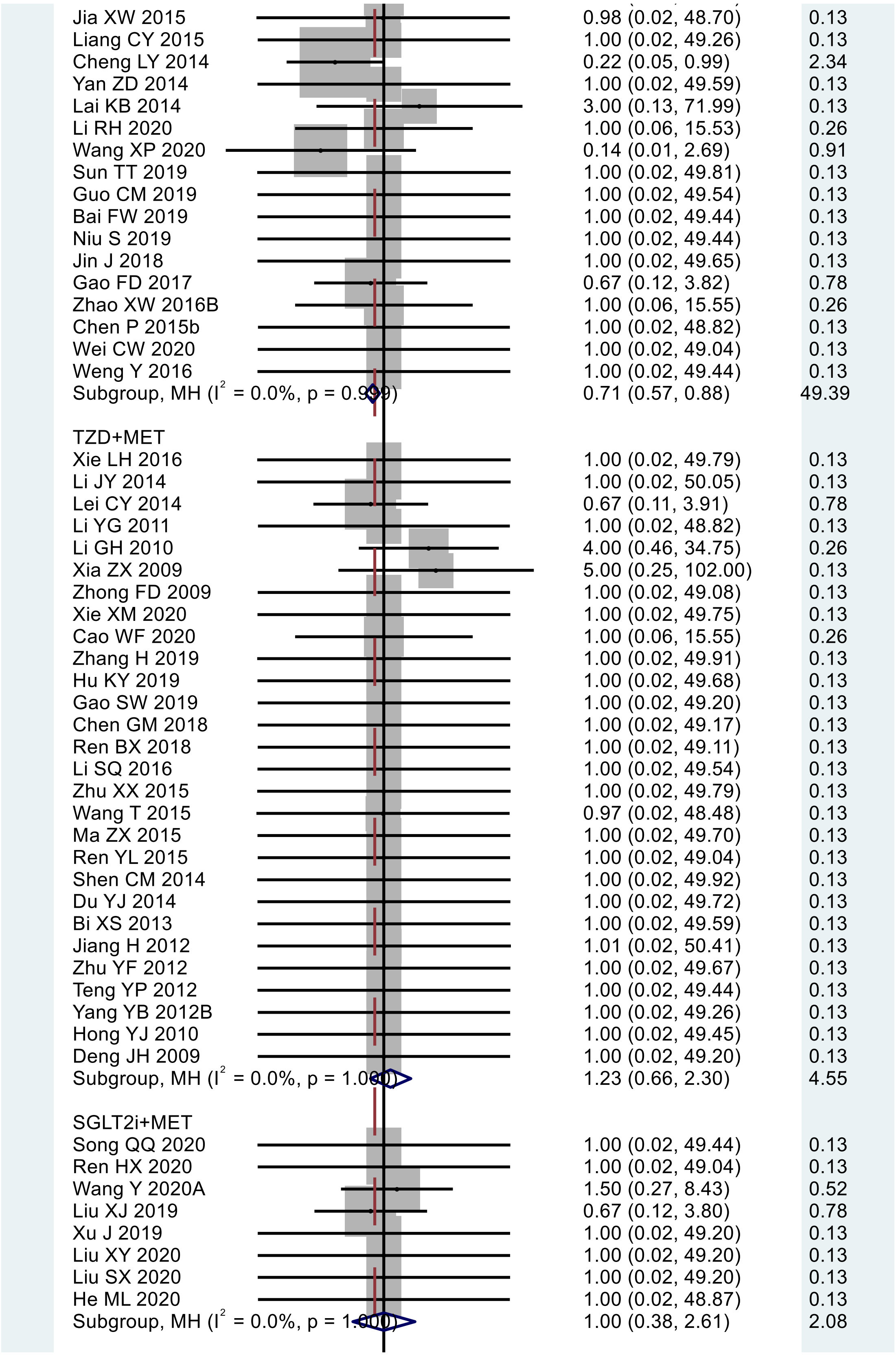
Jia XW 2015 0.98 (0.02, 48.70) 0.13
Liang CY 2015 1.00 (0.02, 49.26) 0.13
Cheng LY 2014 0.22 (0.05, 0.99) 2.34
Yan ZD 2014 1.00 (0.02, 49.59) 0.13
Lai KB 2014 3.00 (0.13, 71.99) 0.13
Li RH 2020 1.00 (0.06, 15.53) 0.26
Wang XP 2020 0.14 (0.01, 2.69) 0.91
Sun TT 2019 1.00 (0.02, 49.81) 0.13
Guo CM 2019 1.00 (0.02, 49.54) 0.13
Bai FW 2019 1.00 (0.02, 49.44) 0.13
Niu S 2019 1.00 (0.02, 49.44) 0.13
Jin J 2018 1.00 (0.02, 49.65) 0.13
Gao FD 2017 0.67 (0.12, 3.82) 0.78
Zhao XW 2016B 1.00 (0.06, 15.55) 0.26
Chen P 2015b 1.00 (0.02, 48.82) 0.13
Wei CW 2020 1.00 (0.02, 49.04) 0.13
Weng Y 2016 1.00 (0.02, 49.44) 0.13
Subgroup, MH (I^2 = 0.0%, p = 0.999) 0.71 (0.57, 0.88) 49.39
TZD+MET
Xie LH 2016 1.00 (0.02, 49.79) 0.13
Li JY 2014 1.00 (0.02, 50.05) 0.13
Lei CY 2014 0.67 (0.11, 3.91) 0.78
Li YG 2011 1.00 (0.02, 48.82) 0.13
Li GH 2010 4.00 (0.46, 34.75) 0.26
Xia ZX 2009 5.00 (0.25, 102.00) 0.13
Zhong FD 2009 1.00 (0.02, 49.08) 0.13
Xie XM 2020 1.00 (0.02, 49.75) 0.13
Cao WF 2020 1.00 (0.06, 15.55) 0.26
Zhang H 2019 1.00 (0.02, 49.91) 0.13
Hu KY 2019 1.00 (0.02, 49.68) 0.13
Gao SW 2019 1.00 (0.02, 49.20) 0.13
Chen GM 2018 1.00 (0.02, 49.17) 0.13
Ren BX 2018 1.00 (0.02, 49.11) 0.13
Li SQ 2016 1.00 (0.02, 49.54) 0.13
Zhu XX 2015 1.00 (0.02, 49.79) 0.13
Wang T 2015 0.97 (0.02, 48.48) 0.13
Ma ZX 2015 1.00 (0.02, 49.70) 0.13
Ren YL 2015 1.00 (0.02, 49.04) 0.13
Shen CM 2014 1.00 (0.02, 49.92) 0.13
Du YJ 2014 1.00 (0.02, 49.72) 0.13
Bi XS 2013 1.00 (0.02, 49.59) 0.13
Jiang H 2012 1.01 (0.02, 50.41) 0.13
Zhu YF 2012 1.00 (0.02, 49.67) 0.13
Teng YP 2012 1.00 (0.02, 49.44) 0.13
Yang YB 2012B 1.00 (0.02, 49.26) 0.13
Hong YJ 2010 1.00 (0.02, 49.45) 0.13
Deng JH 2009 1.00 (0.02, 49.20) 0.13
Subgroup, MH (I^2 = 0.0%, p = 1.000) 1.23 (0.66, 2.30) 4.55
SGLT2i+MET
Song QQ 2020 1.00 (0.02, 49.44) 0.13
Ren HX 2020 1.00 (0.02, 49.04) 0.13
Wang Y 2020A 1.50 (0.27, 8.43) 0.52
Liu XJ 2019 0.67 (0.12, 3.80) 0.78
Xu J 2019 1.00 (0.02, 49.20) 0.13
Liu XY 2020 1.00 (0.02, 49.20) 0.13
Liu SX 2020 1.00 (0.02, 49.20) 0.13
He ML 2020 1.00 (0.02, 48.87) 0.13
Subgroup, MH (I^2 = 0.0%, p = 1.000) 1.00 (0.38, 2.61) 2.08

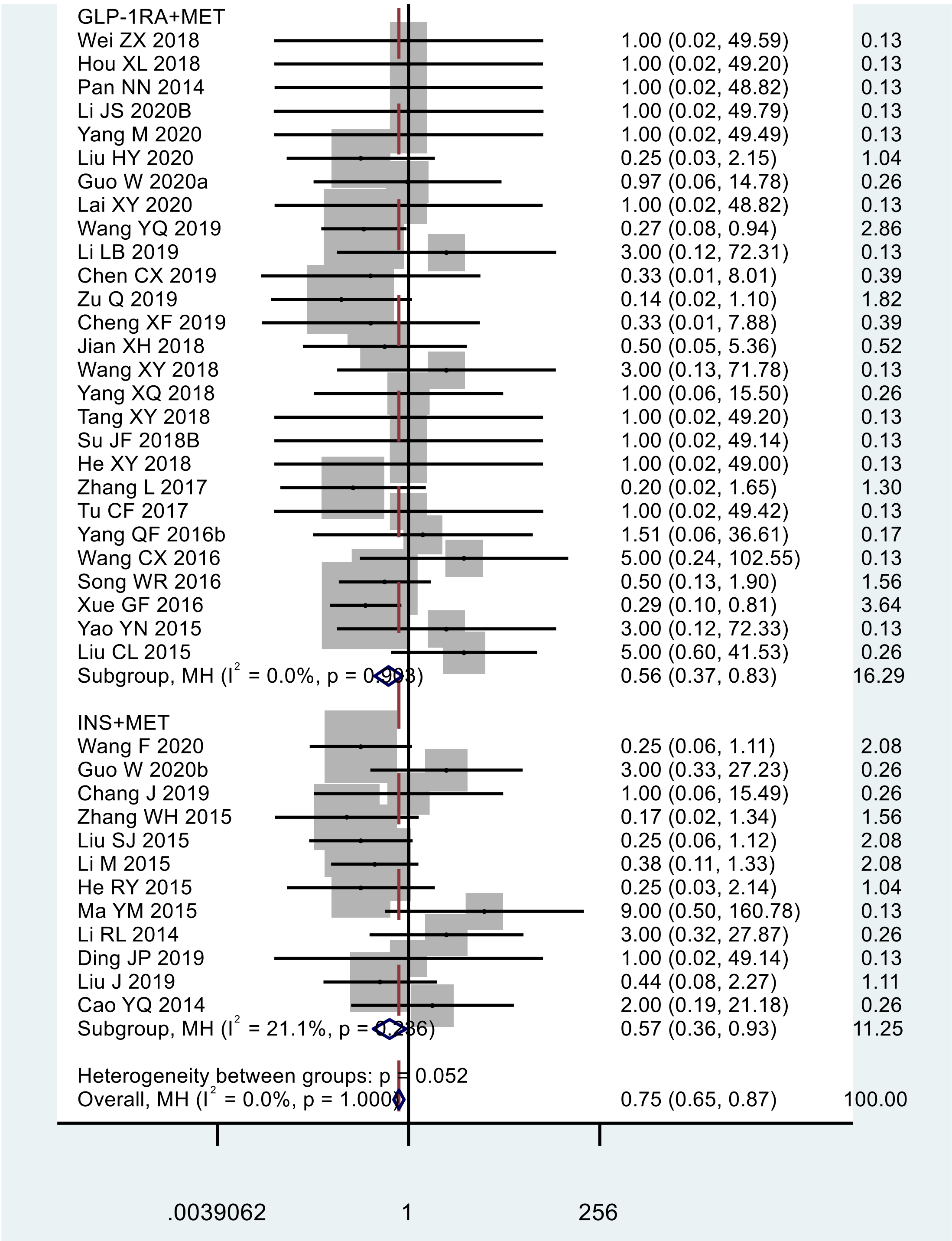

Figure S68. Sensitivity analysis results for incidence of hypoglycemia of glucose-lowering drugs added to metformin compared with metformin monotherapy

Reference

1. Gao, H. Efficacy observation of acarbose combined with metformin in the treatment of type 2 diabetes complicated with hyperlipidemia. *Journal of Practical Diabetology* **2020**, *16*, 8.
2. Chen, B.Z. Effect of acarbose combined with metformin on glycosylated hemoglobin and serum β2-microglobulin in elderly patients with type 2 diabetes. *Journal of North Pharmacy* **2020**, *17*, 61-63.
3. Li, S.J. Clinical study of acarbose combined with metformin in the treatment of type 2 diabetes. *Special Health* **2020**, 38.
4. Xing, C.L. Efficacy observation of acarbose combined with metformin in the treatment of newly diagnosed type 2 diabetes complicated with hyperlipidemia. *Chinese Community Doctors* **2020**, *36*, 47-48.
5. Xing, L.Y.; Zhang, J.J. Comparison of therapeutic effect of miglitol and acarbose combined with metformin in elderly patients with type 2 diabetes. *Diabetes New World* **2020**, *23*, 59-61.
6. Zhu, X.F. Effect of metformin hydrochloride sustained release tablets combined with acarbose on blood glucose index and quality of life in patients with type 2 diabetes. *Modern Practical Medicine* **2019**, *31*, 584-586.
7. Tang, W.Q. Efficacy analysis of acarbose combined with metformin in the treatment of type 2 diabetes. *Journal of Frontiers of Medicine* **2019**, *9*, 121.
8. Si, J. Clinical analysis of acarbose combined with metformin in the treatment of type 2 diabetes. *Medical Diet and Health* **2019**, 66, 68.
9. Huo, H.W. Effect evaluation of acarbose combined with metformin in newly diagnosed type 2 diabetes patients with hyperlipidemia. *Strait Pharmaceutical Journal* **2019**, *31*, 169-171.
10. Chen, Z.H. Effect of metformin combined with acarbose on glycemic control in patients with type 2 diabetes in community. *Home Medicine* **2019**, 81.
11. Li, S.H. Effect of acarbose combined with metformin on glycolipid metabolism in patients with type 2 diabetes complicated by hyperlipidemia. *Clin. Med.* **2019**, *39*, 101-103.
12. Li, S.H. Clinical analysis of metformin hydrochloride sustained-release tablets combined with acarbose in the treatment of 65 cases of type 2 diabetes. *Strait Pharmaceutical Journal* **2019**, *31*, 187-188.
13. Huang, W.J. Effect of acarbose combined with metformin on insulin resistance and body weight in patients with type 2 diabetes. *Drug Evaluation* **2019**, *16*, 49-50.
14. Yao, W.L. Efficacy observation of acarbose combined with metformin in the treatment of newly diagnosed type 2 diabetes with hyperlipidemia. *Strait Pharmaceutical Journal* **2018**, *30*, 203-205.
15. Yang, H.; Wang, S.J.; Zuo, H.; Qi, T.; Liu, S.F. Efficacy observation of metformin combined with acarbose in the treatment of newly diagnosed type 2 diabetes. *Hainan Medical Journal* **2018**, *29*, 1442-1444.
16. Wang, Y.L.; Zhang, C.H. Effect and adverse reaction rate analysis of metformin combined with acarbose in the treatment of patients with type 2 diabetes. *Diabetes World* **2018**, *15*, 52.
17. Wu, Y.Y.; Chen, Y.M. Effect of metformin enteric-coated tablets combined with acarbose tablets in the treatment of type 2 diabetes. *Journal of Frontiers of Medicine* **2018**, *8*.
18. Tang, J. Clinical efficacy of acarbose combined with metformin in the treatment of newly diagnosed type 2 diabetes complicated with hyperlipidemia. *Chinese Journal of Clinical Rational Drug Use* **2018**, *11*, 44-46.
19. Fang, Y. Clinical efficacy observation of acarbose combined with metformin in the treatment of type 2 diabetes. *World Latest Medicine Information* **2018**, *18*, 122.
20. Jiang, K.R. Clinical efficacy analysis of metformin combined with acarbose in the treatment of type 2 diabetes complicated with hyperlipidemia. *Diabetes New World* **2017**, *20*, 91-93.
21. Jiao, Y.F. Efficacy and adverse reaction analysis of glucobay and metformin in the treatment of type 2 diabetes. *Health Literature* **2017**, 157.
22. Ren, H.B. Effect observation of metformin combined with acarbose in the treatment of type 2 diabetes. *Contemporary Medical Symposium* **2017**, *15*, 115-116.
23. Yang, X. Clinical effect of acarbose and metformin in the treatment of newly diagnosed type 2 diabetes. *Clinical Research and Practice* **2017**, *2*, 52-53.
24. Guo, C.X.; Li, M. Efficacy observation of metformin hydrochloride combined with acarbose in the treatment of type 2 diabetes with hypertension and hyperlipidemia. *Electronic Journal of Clinical Medical Literature* **2017**, *4*, 20169-20171.
25. Han, J.K.; Pang, W.Y.; Yang, X.Z.; Xue, L. Efficacy observation of metformin combined with acarbose in the treatment of type 2 diabetes. *China Health Standard Management* **2016**, *7*, 116-118.
26. Huang, L.Y.; Liao, B.; Yang, M.; Liu, J.H.; Quan, H.M.; Qin, A.P. Efficacy analysis of acarbose combined with metformin in the treatment of newly diagnosed type 2 diabetes with hyperlipidemia. *Medical Aesthetics and Cosmetology* **2015**, 211.
27. Wang, X.Q. Efficacy observation of acarbose combined with metformin in the treatment of newly diagnosed type 2 diabetes patients with hyperlipidemia. *Electronic Journal of Practical Gynecological Endocrinology* **2015**, 172-173.
28. Liu, J. Efficacy observation of acarbose combined with metformin in the treatment of type 2 diabetes. *The Medical Forum* **2015**, 5047-5049.
29. Zu, S.Q. Clinical treatment of elderly patients with type 2 diabetes. *Health Protection and Promotion* **2015**, 45.
30. Tan, Y.L.; Huang, Y.N. Effect study of acarbose combined with metformin on glycemic control and islet function in elderly patients with refractory type 2 diabetes. *Clinical Medicine & Engineering* **2014**, *21*, 1422-1423.
31. Zeng, X.Y. Clinical analysis of acarbose combined with metformin in the treatment of elderly patients with type 2

diabetes. *Jilin Medical Journal* **2014**, 2159-2160.
32. Zhu, Z.L.; Qiu, X.C.; Zhu, H.P.; Ding, Z.J. Efficacy observation of acarbose combined with metformin in the treatment of patients newly diagnosed with type 2 diabetes. *Heilongjiang Medicine Journal* **2011**, 223-225.
33. Tan, R.F. Efficacy observation of voglibose combined with metformin in the treatment of newly diagnosed type 2 diabetes. *Strait Pharmaceutical Journal* **2017**, *29*, 110-112.
34. Huang, Y. Efficacy observation of miglitol combined with metformin in the treatment of type 2 diabetes. *Chinese Baby* **2015**, 206, 207.
35. He, K.; Liu, L.H.; Shi, Z.N.; Yuan, Y.H. Efficacy observation of miglitol combined with metformin in the treatment of type 2 diabetes. *People's Military Surgeon* **2014**, *57*, 1216-1217.
36. Wang, X.H. Effect analysis of glimepiride combined with metformin in the treatment of elderly patients with type 2 diabetes. *Contemporary Medical Symposium* **2020**, *18*, 122-123.
37. Lin, M.L. To observe the efficacy of glimepiride combined with metformin in the treatment of type 2 diabetes. *Psychological Doctor* **2019**, *25*, 155-156.
38. Chen, Y.F. Clinical efficacy of glimepiride dispersible tablets combined with metformin hydrochloride tablets in the treatment of diabetes and its effect on glycemic level of patients. *Diabetes New World* **2019**, *22*, 85-86.
39. Leng, B. Clinical efficacy analysis of glimepiride combined with metformin in the treatment of type 2 diabetes. *Psychological Doctor* **2018**, *24*, 41-42.
40. Wang, X.S. Effect study of the combination of glimepiride and metformin in the treatment of elderly patients with type 2 diabetes. *Contemporary Medical Symposium* **2017**, *15*, 131-132.
41. Chen, S.P. Efficacy and safety study of glimepiride combined with metformin in the treatment of type 2 diabetes in the elderly. *Diabetes New World* **2017**, *20*, 79-80.
42. Yang, Q.F.; Nie, F.R.; Xu, W.L. Clinical efficacy and safety of metformin combined with liraglutide in the treatment of type 2 diabetes. *Lingnan Journal of Emergency Medicine* **2016**, *21*, 40-42.
43. Mao, Z.H.; Tai, S.E.G.L. Effect of glimepiride combined with metformin on insulin resistance in patient with type 2 diabetes. *Chinese and Foreign Medical Research* **2016**, *14*, 134-135.
44. Ma, C.H. Effect of liraglutide on insulin resistance in patients with type 2 diabetes, Xinjiang Medical University.
45. Xu, Y.J. Clinical observation of glimepiride combined with metformin in the treatment of 39 cases of type 2 diabetes. *Chinese Journal of Ethnomedicine and Ethnopharmacy* **2015**, 85.
46. Li, X. Efficacy of glimepiride combined with metformin in the treatment of type 2 diabetes. *Clin. Med.* **2012**, 57-58.
47. Yi, Y.D. Clinical effect observation of gliclazide combined with metformin in the treatment of type 2 diabetes. *Contemporary Medicine* **2019**, *25*, 164-165.
48. Chen, Y.F.; Mai, W.H. Efficacy and safety of gliclazide extended-release tablets in the treatment of type 2 diabetes. *China Health Care & Nutrition* **2013**, 578-579.
49. He, F. Clinical efficacy analysis of metformin combined with gliclazide sustained-release tablets in the treatment of type 2 diabetes. *Healthmust-Readmagazine* **2013**, 420.
50. Luo, Z.Z.; Wang, Y.L.; He, S.X. Clinical efficacy study of metformin combined with gliclazide sustained release capsule in the treatment of type 2 diabetes. *China Medicine and Pharmacy* **2012**, *2*, 100-101.
51. Guo, X.T. Effect of glipizide combined with metformin on serum insulin level in patients with type 2 diabetes. *Modern Diagnosis and Treatment* **2020**, *31*, 710-712.
52. Wang, Q.; Bian, Y.J.; Wang, J. Effect observation of glipizide combined with metformin in the treatment of elderly patients with type 2 diabetes. *Diabetes World* **2020**, *17*, 53-54.
53. Sun, H.Q. Clinical effect study of glipizide combined with metformin in the treatment of elderly patients with newly diagnosed type 2 diabetes. *Diabetes New World* **2019**, *22*, 57-58.
54. Bao, Y.M.; Zheng, L.; Chen, S.; Wu, X.D. Efficacy observation of metformin hydrochloride and glipizide alone or in combination in elderly patients with type 2 diabetes. *Modern Practical Medicine* **2017**, *29*, 341-343.
55. Wang, S.Q. A randomized controlled study of metformin monotherapy and different combination regimens in the treatment of type 2 diabetes. *Guide of China Medicine* **2016**, *14*, 26-27.
56. Guo, S.B.; Gao, S.X. Clinical efficacy study of metformin combined with glipizide in the treatment of type 2 diabetes. *Medical Recapitulate* **2012**, *18*, 1606-1607.
57. Liu, G.L.; Zhao, X.J.; Du, J.; Wang, Q.Y.; Zhang, J.; Zheng, X.M.; Cao, C.P.; Ren, L.P. Clinical efficacy observation of glurenorm in the treatment of type 2 diabetes. *Liaoning Journal of Practical Diabetology* **2004**, 58-60.
58. Pi, H.C. Effect of repaglinide combined with metformin in the treatment of patients with type 2 diabetes in community. *Strait Pharmaceutical Journal* **2020**, *32*, 181-182.
59. Ye, Q.; Zhou, X.; Li, Y. Effect of repaglinide combined with metformin on blood glucose fluctuation and oxidative stress in elderly patients with type 2 diabetes. *Journal of Clinical and Experimental Medicine* **2020**, *19*, 2192-2196.
60. Zhou, C. Effect of repaglinide combined with metformin in the treatment of type 2 diabetes. *Medical Journal of Chinese People's Health* **2019**, *31*, 34-35.
61. Yan, H.P. Efficacy study of metformin combined with repaglinide in the treatment of type 2 diabetes. *China Health Vision* **2019**, 38-39.
62. Zhu, L.F. Metformin combined with repaglinide in the treatment of type 2 diabetes. *Modern Medicine and Health Research* **2018**, *2*, 98-99.
63. Li, J.P. Effect analysis of metformin combined with repaglinide in the treatment of newly diagnosed type 2 diabetes with hyperglycemia. *Journal of Practical Diabetology* **2018**, *14*, 25-26.

64. Gao, S. Effect of repaglinide combined with metformin on glycemic control and quality of life in patients with type 2 diabetes. *Journal of North Pharmacy* **2017**, *14*, 65-66.
65. Huang, Y.Y. Effect analysis of repaglinide and metformin in the treatment of newly diagnosed type 2 diabetes. *Diabetes New World* **2017**, *20*, 91-92.
66. Han, J. Effect study of repaglinide combined with metformin in patients with newly diagnosed type 2 diabetes. *Henan Medical Research* **2017**, *26*, 3363-3364.
67. Hu, Z.Y. Effect study of repaglinide combined with metformin in the treatment of newly diagnosed type 2 diabetes. *Medical Information* **2016**, *29*, 64-65.
68. Yu, Y.Y. Effect analysis of repaglinide combined with metformin in the treatment of obese type 2 diabetes patients. *Journal of North Pharmacy* **2015**, *12*, 23-24.
69. Sun, Z.C. Efficacy of repaglinide combined with metformin in the treatment of type 2 diabetes. *Diabetes New World* **2015**, 29-30.
70. Xu, S.L. Efficacy of repaglinide combined with metformin in the treatment of newly diagnosed type 2 diabetes. *China Practical Medicine* **2013**, *8*, 144-146.
71. He, M. Clinical observation of repaglinide combined with metformin in the treatment of type 2 diabetes. *China Practical Medicine* **2013**, *8*, 144-145.
72. He, M. Clinical observation of repaglinide in the treatment of 61 cases of newly diagnosed type 2 diabetes. *Chinese Journal of Modern Drug Application* **2013**, *7*, 149-150.
73. Lei, Y.T. Effect of metformin combined with DPP-4 inhibitor on serum HbA1c. *Chinese Journal of Urban and Rural Enterprise Hygiene* **2020**, *35*, 165-166.
74. Hu, D. Clinical effect observation of sitagliptin phosphate combined with metformin in the treatment of newly diagnosed type 2 diabetes. *Medical Community* **2020**, *0*, 34.
75. Ding, X.Y. To explore the effect of sitagliptin combined with metformin on blood glucose level and efficacy in patients with type 2 diabetes. *Diabetes New World* **2020**, *23*, 66-68.
76. Luo, H.Q.; Tan, Q.M.; Xu, H.X.; Zheng, Q.Y.; Chen, B.L. Efficacy observation of metformin combined with sitagliptin in the treatment of type 2 diabetes complicated with metabolic syndrome. *Smart Healthcare* **2020**, *6*, 125-127.
77. Zhang, X.L. Efficacy and safety of sitagliptin combined with metformin in the treatment of type 2 diabetes with abdominal obesity. *Special Health* **2020**, 78.
78. Liang, Y.F. Clinical efficacy of sitagliptin combined with metformin in the treatment of obese type 2 diabetes and its effect on body fat content. *Chinese Journal of Clinical Rational Drug Use* **2020**, *13*, 87-88.
79. Li, Y. Clinical effect of sitagliptin phosphate combined with metformin in the treatment of patients with newly diagnosed type 2 diabetes. *Medical Journal of Chinese People's Health* **2020**, *32*, 36-37.
80. Sun, X.O. Efficacy study of sitagliptin phosphate combined with metformin in the treatment of newly diagnosed type 2 diabetes. *Chinese Journal of Modern Drug Application* **2020**, *14*, 185-187.
81. He, C. Efficacy and safety of sitagliptin combined with metformin in the treatment of type 2 diabetes with abdominal obesity. *Electronic Journal of Clinical Medical Literature* **2020**, *7*, 165.
82. Xiao, B.M.; Ye, Y.X. Effect observation of sitagliptin combined with metformin in the treatment of young and middle-aged patients with type 2 diabetes. *Healthmust-Readmagazine* **2020**, 85.
83. Wu, S.L. Clinical effect of sitagliptin phosphate combined with metformin in the treatment of newly diagnosed type 2 diabetes. *Henan Medical Research* **2020**, *29*, 2196-2198.
84. Chen, J. Clinical effect observation of metformin combined with sitagliptin in the treatment of type 2 diabetes. *Chinese Journal of Clinical Rational Drug Use* **2020**, *13*, 7-8.
85. Niu, X.P. Effect analysis of sitagliptin phosphate combined with metformin in the treatment of type 2 diabetes. *Diabetes New World* **2020**, *23*, 39-40.
86. Yang, X.Q. Clinical effect of sitagliptin phosphate combined with metformin in the treatment of newly diagnosed type 2 diabetes. *Chinese and Foreign Medical Research* **2020**, *18*, 18-20.
87. Hu, B.; Fan, H.; Yao, J. The effect of metformin combined with sitagliptin on type 2 diabetes mellitus and the islets function. *Acta Medica Mediterr.* **2020**, *36*, 3631-3634.
88. Liu, F. Effect of sitagliptin phosphate combined with metformin in the treatment of type 2 diabetes. *Shenzhen Journal of Integrated Traditional Chinese and Western Medicine* **2019**, *29*, 108-110.
89. Liu, L. Clinical effect and safety of sitagliptin combined with metformin in the treatment of type 2 diabetes. *Guide of China Medicine* **2019**, *17*, 126-127.
90. Wang, J. Effect of metformin combined with dipeptidyl peptidase 4 inhibitor on metabolic indexes in patients with type 2 diabetes complicated with metabolic syndrome. *Zhejiang Practical Medicine* **2019**, *24*, 172-174.
91. Li, J.; Chen, C.; Song, W.R. Efficacy observation of sitagliptin combined with metformin in the treatment of type 2 diabetes. *Evaluation and Analysis of Drug-Use in Hospitals of China* **2019**, *19*, 441-442.
92. Xie, D. Analysis of the value of sitagliptin combined with metformin in the treatment of type 2 diabetes with abdominal obesity. *Diabetes World* **2019**, *16*, 86-87.
93. Li, B.A. Effect of sitagliptin phosphate combined with metformin on glycolipid metabolism in patients with type 2 diabetes. *Tibetan Medicine* **2019**, *40*, 63-65.
94. Wu, P. Effect of sitagliptin phosphate combined with metformin in the treatment of patients newly diagnosed with type 2 diabetes. *Cardiovascular Disease Electronic Journal of Integrated Traditional Chinese and Western Medicine* **2019**, *7*, 57-59.

95. Yang, W.D. Clinical efficacy analysis of metformin combined with sitagliptin in the treatment of patients newly diagnosed with type 2 diabetes. *Drug Evaluation Research* **2018**, *41*, 643-647.
96. Li, H.X. Evaluation of the clinical effect of sitagliptin combined with metformin hydrochloride enteric-coated capsules in the treatment of type 2 diabetes. *Cardiovascular Disease Electronic Journal of Integrated Traditional Chinese and Western Medicine* **2018**, *6*, 186-187.
97. Jing, H.W.; Hao, L.H. Effect of metformin combined with sitagliptin phosphate on HOMA-IR、Apelin and vaspin levels in patients with diabetes mellitus. *Clinical Research and Practice* **2018**, *3*, 8-10.
98. Chen, L. Clinical effect of sitagliptin phosphate combined with metformin in the treatment of newly diagnosed type 2 diabetes. *Chinese Journal of Practical Medicine* **2018**, *45*, 121-123.
99. Chen, Z.; He, Y.; Ma, W.Q.; Wang, G.J. Clinical effect and safety of sitagliptin combined with metformin in the treatment of type 2 diabetes with abdominal obesity. *China Medicine* **2018**, *13*, 545-549.
100. Li, F.; Qu, Z.L. Clinical study of sitagliptin combined with metformin in the treatment of type 2 diabetes complicated with nonalcoholic fatty liver disease. *The Chinese Journal of Clinical Pharmacology* **2018**, *34*, 518-520.
101. Zhou, Q.M. Effect of sitagliptin combined with metformin on blood glucose and insulin resistance in patients with newly diagnosed type 2 diabetes. *Journal of Practical Diabetology* **2018**, *14*, 57.
102. Zhu, H.Q.; Xiong, W.H. Efficacy of sitagliptin combined with metformin in the treatment of newly diagnosed type 2 diabetes. *Chinese Journal of Rational Drug Use* **2018**, *15*, 55-58.
103. Zhang, C.J.; Tang, H.X.; Bao, X.J.; Tian, J.X.; Xin, L.J. Clinical efficacy observation of sitagliptin in the treatment of type 2 diabetes. *Psychological Doctor* **2018**, *24*, 91-92.
104. Tang, Y.; Yang, Y. Clinical effect observation of metformin combined with sitagliptin phosphate in the treatment of type 2 diabetes. *Journal of Aerospace Medicine* **2017**, *28*, 139-140.
105. Guan, X.P.; Sun, H.B.; Liu, J. Effect analysis of sitagliptin combined with metformin in the treatment of patients with type 2 diabetes. *Diabetes New World* **2017**, *20*, 82-83.
106. Gao, H.M. Application effect of sitagliptin phosphate and metformin in the treatment of incipient type 2 diabetes. *Journal of North Pharmacy* **2017**, *14*, 88.
107. Yang, N.; Wei, Q.M.; Wang, X. Efficacy of sitagliptin phosphate combined with metformin in the treatment of elderly patients with type 2 diabetes and its effect on blood glucose, insulin and lipid metabolism. *Chinese Journal of Practical Medicine* **2017**, *44*, 63-66.
108. Zhou, Y.; Han, L. Efficacy of sitagliptin phosphate combined with metformin in the treatment of patients with newly diagnosed type 2 diabetes. *Anhui Medical Journal* **2017**, *38*, 476-478.
109. Li, J.J.; Zhou, L.; Liu, X.L. Clinical efficacy observation of sitagliptin combined with metformin in the treatment of type 2 diabetes. *Medical Journal of Chinese People's Health* **2017**, *29*, 19-20.
110. Ji, L.N.; Han, P.; Wang, X.Y.; Liu, J.D.; Zheng, S.X.; Jou, Y.M.; O'Neill, E.A.; Golm, G.T.; Engel, S.S.; Kaufman, K.D.; et al. Randomized clinical trial of the safety and efficacy of sitagliptin and metformin co-administered to Chinese patients with type 2 diabetes mellitus. *Journal of Diabetes Investigation* **2016**, *7*.
111. Yang, Q.M. Efficacy and safety of sitagliptin combined with metformin in the treatment of newly diagnosed type 2 diabetes. *Journal of Frontiers of Medicine* **2016**, *6*, 207-208.
112. Hu, G.H. Clinical effect observation of metformin combined with sitagliptin in the treatment of newly diagnosed type 2 diabetes. *Clinical Research* **2016**, *24*, 234-235.
113. Wu, X.H. Effect observation of sitagliptin combined with metformin in the treatment of type 2 diabetes. *Chinese Journal of Critical Care Medicine* **2016**, *36*, 99-100.
114. Yang, F. Clinical efficacy of sitagliptin combined with metformin in the treatment of type 2 diabetes. *Journal of Today Health* **2016**, *15*, 26.
115. Li, L.Q. Clinical effect of metformin combined with sitagliptin phosphate in the treatment of newly diagnosed type 2 diabetes. *The World Clinical Medicine* **2016**, *10*, 104, 108.
116. Zhang, T.T. Efficacy of metformin combined with sitagliptin in the treatment of patients with type 2 diabetes and its effect on improvement of blood lipid levels. *Modern Practical Medicine* **2016**, 1037-1038.
117. Han, M. Efficacy of sitagliptin combined with metformin in the treatment of type 2 diabetes. *World Latest Medicine Information* **2016**, 79-80.
118. Pang, X.X. Effect of sitagliptin combined with metformin in the treatment of type 2 diabetes. *Journal of Practical Diabetology* **2016**, 42-43.
119. Wang, X.L. Efficacy analysis of sitagliptin in the treatment of diabetes. *Clinical Research and Practice* **2016**, 41.
120. Qiao, Y.C. Clinical study of sitagliptin phosphate combined with metformin in the treatment of newly diagnosed type 2 diabetes. *Clinical Research* **2016**, *24*, 71-72.
121. Qi, B.B.; Song, W.C.; Jiang, D.M.; Ji, M. Efficacy of dipeptidyl peptidase-4 inhibitor sitagliptin combined with metformin in the treatment of type 2 diabetes. *Jiangsu Medical Journal* **2016**, 1728-1729.
122. Jiang, X. Clinical effect observation of sitagliptin combined with metformin in the treatment of type 2 diabetes. *Continuing Medical Education* **2016**, 156-157.
123. Yao, L.; Wu, Y.T.; Zhang, W.; Xia, C.Q.; Tian, G.X.; Wang, X.B. Clinical efficacy of sitagliptin phosphate combined with metformin in the treatment of type 2 diabetes. *Chinese Journal of Clinical Healthcare* **2016**, 352-354.
124. Zhang, N.; Wang, Y.; Liu, Y.L. Effect of sitagliptin combined with metformin in the treatment of type 2 diabetes. *International Medicine and Health Guidance News* **2015**, *21*, 3211-3213.
125. Yuan, X.J.; Ma, L.F.; Yang, H. Efficacy evaluation of metformin combined with sitagliptin phosphate in the treatment

of newly diagnosed type 2 diabetes. *Chinese Journal of Modern Drug Application* **2015**, *9*, 123-124.
126. Zhang, H.T.; Wang, F.; Dong, L. Efficacy observation of sitagliptin combined with metformin in the treatment of patients newly diagnosed with type 2 diabetes. *Journal of Practical Diabetology* **2015**, 22-23.
127. Zhuo, F.T.; Zhang, L.T.; Gao, X.H. Effect of sitagliptin on pancreatic B cell function in the treatment of newly diagnosed type 2 diabetes. *Practical Pharmacy and Clinical Remedies* **2015**, 622-624.
128. Fan, S.H. Effect observation of sitagliptin combined with metformin in the treatment of newly diagnosed type 2 diabetes. *Modern Practical Medicine* **2015**, 1632-1634.
129. Li, Z.; He, X.T.; Li, T.; Sun, Z.H. Clinical analysis of sitagliptin combined with metformin in the treatment of type 2 diabetes. *China Health Standard Management* **2015**, 86-87.
130. Chen, P. Clinical efficacy observation of DPP- IV inhibitor combined with metformin in type 2 diabetes. *Journal of North Pharmacy* **2015**, 88-89.
131. Guo, Y.F.; Zhang, D.Q. Clinical effect observation of sitagliptin phosphate tablets combined with metformin in the treatment of elderly patients with type 2 diabetes. *China Modern Medicine* **2014**, 93-94.
132. Zhang, X.F.; Yang, X.H.; Dong, L.L. Clinical observation of sitagliptin phosphate combined with metformin in the treatment of newly diagnosed type 2 diabetes. *Hebei Medical Journal* **2014**, *36*, 2785-2787.
133. Qiu, W. Metformin combined with sitagliptin in the treatment of type 2 diabetes complicated with metabolic syndrome. *Chinese Journal of Modern Drug Application* **2014**, *8*, 128-129.
134. Nie, F.R.; Xu, W.L.; Yang, Q.F. Clinical efficacy of metformin combined with sitagliptin in the treatment of early type 2 diabetes. *Practical Journal of Cardiac Cerebral Pneumal and Vascular Disease* **2013**, *21*, 81-82.
135. Li, W.M. Effectiveness and safety study of sitagliptin combined with metformin in the treatment of patients with type 2 diabetes. *Journal of Clinical and Experimental Medicine* **2013**, *12*, 922-923.
136. Zhang, L.L. Clinical efficacy of sitagliptin and metformin in treatment of type 2 diabetes mellitus. *China Medicine and Pharmacy* **2013**, *3*, 65-66.
137. Zhang, H.Y.; Dong, L. Clinical efficacy analysis of sitagliptin combined with metformin in the treatment of newly diagnosed type 2 diabetes. *China Health Care & Nutrition* **2013**, *23*, 3900-3901.
138. Jiao, X.M.; Xu, X.P.; Zhao, J.; Lv, X.F. Clinical observation of sitagliptin phosphate combined with metformin in the treatment of newly diagnosed type 2 diabetes. *Clinical Focus* **2013**, 44-45.
139. Li, J. Effect of saxagliptin combined with metformin in the treatment of elderly patients with type 2 diabetes. *Medical Journal of Chinese People's Health* **2020**, *32*, 21-23.
140. Deng, Y.Y.; Liao, T.J.; Lin, Y.S. Clinical effect of saxagliptin in patients with type 2 diabetes complicated by metabolic syndrome and its effect on microinflammatory state. *China Modern Medicine* **2020**, *27*, 98-101.
141. Zheng, H.Y. Effect of saxagliptin combined with metformin on glycemic control and islet β-cell function in patients newly diagnosed with type 2 diabetes. *Journal of North Pharmacy* **2020**, *17*, 57-58.
142. Zhao, P.Y. Analysis of the value of saxagliptin combined with metformin in the treatment of type 2 diabetes with abdominal obesity. *Electronic Journal of Clinical Medical Literature* **2019**, *6*, 55.
143. Mei, Y.J.; Gan, T. Clinical observation of the efficacy of saxagliptin combined with metformin in newly diagnosed type 2 diabetes complicated with nonalcoholic fatty liver disease. *Journal of Frontiers of Medicine* **2018**, *8*, 395-396.
144. Shi, F.M. To study the efficacy and characteristics of saxagliptin and metformin sustained-release tablets in the treatment of type 2 diabetes. *Smart Healthcare* **2018**, *4*, 99-100.
145. Shan, L. Effect analysis of saxagliptin combined with metformin sustained-release tablets in the treatment of type 2 diabetes. *China & Foreign Medical Treatment* **2018**, *37*, 8-10.
146. Tao, T.; Wu, P.; Wang, Y.; Liu, W. Comparison of glycemic control and β-cell function in new onset T2DM patients with PCOS of metformin and saxagliptin monotherapy or combination treatment. *BMC Endocr. Disord.* **2018**, *18*, 14.
147. Li, G.H. Efficacy of saxagliptin combined with metformin in the treatment of patients with newly diagnosed type 2 diabetes and its influence on related metabolic indices, China Medical University, 2017-01-01.
148. Dong, H.; Tian, J.; Zhang, Y.Y.; Shen, J.X. Clinical effect of saxagliptin combined with metformin in the treatment of type 2 diabetes complicated with nonalcoholic fatty liver disease. *Guangxi Medical Journal* **2017**, *39*, 316-318.
149. Zhang, W. Efficacy observation of saxagliptin combined with metformin in the treatment of newly diagnosed type 2 diabetes. *Journal of Medical Information* **2017**, *30*, 113-114.
150. Li, H.W. Effect of saxagliptin as add-on therapy on glycolipid metabolism and insulin sensitivity in patients with type 2 diabetes poorly controlled on metformin. *Heilongjiang Medical Journal* **2017**, *41*, 335-336.
151. Zhang, G.P. Efficacy and adverse reactions of saxagliptin combined with metformin in the treatment of newly diagnosed type 2 diabetes. *Psychological Doctor* **2017**, *23*, 110-111.
152. Ding, R.; Ding, G.C.; Cai, R.H. Efficacy and safety of dipeptidyl peptidase-4 inhibitor combined with metformin in the treatment of type 2 diabetes. *Chinese Journal of Biochemical and Pharmaceuticals* **2016**, 154-156.
153. Chen, Q.L. Efficacy observation of saxagliptin combined with metformin in the treatment of newly diagnosed type 2 diabetes. *Guide of China Medicine* **2015**, *13*, 133-134.
154. Liang, M. A randomized controlled study of saxagliptin combined with metformin in the treatment of patients newly diagnosed with type 2 diabetes. *Capital Food Medicine* **2015**, 75-76.
155. Feng, J.; Shi, P. Efficacy evaluation of saxagliptin combined with metformin in the treatment of 48 cases of elderly patients with type 2 diabetes. *China Pharmaceuticals* **2015**, 90-92.
156. Diao, Y.L.; Yu, J.H.; Wang, J. Clinical observation of saxagliptin combined with metformin in the treatment of type 2 diabetes. *China Pharmacy* **2015**, 3761-3763.

157. Wan, J.; Li, J.L.; Luo, Q.R.; Zeng, J.E. Efficacy analysis of metformin or saxagliptin alone and in combination in the treatment of type 2 diabetes. *Journal of Yangtze University(Natural Science Edition)* **2015**, 1-2.
158. Sun, Y.; Jin, J.; Wang, M.Z. Preliminary study on the efficacy of saxagliptin combined with metformin in the treatment of newly diagnosed type 2 diabetes. *Chinese Journal of Control of Endemic Diseases* **2014**, *29*, 232-233.
159. Lu, J.E.; Pang, L.R.; Gong, W.K. Efficacy observation of saxagliptin combined with metformin in the treatment of type 2 diabetes. *Modern Practical Medicine* **2014**, 191-192.
160. Sun, Y.; Li, Y.; Wang, M.Z. Efficacy of saxagliptin combined with metformin in the treatment of patients newly diagnosed with type 2 diabetes. *Chinese Journal of Gerontology* **2014**, 4172-4173.
161. Li, J.J.; Liu, C.M.; Dong, X.Y.; Zhang, L. Effect of saxagliptin and/or metformin on islet β-cell function in patients newly diagnosed with type 2 diabetes. *China Medicine and Pharmacy* **2013**, 16-18.
162. Chu, Y.N.; Zhang, X.K.; Li, M.X.; Zhao, Y.L. Application of metformin combined with vildagliptin in the treatment of type 2 diabetes. *Journal of Mathematical Medicine* **2020**, *33*, 1501-1503.
163. Wang, T.M. Clinical efficacy and safety of vildagliptin combined with metformin in the treatment of type 2 diabetes. *Chinese Journal of Clinical Rational Drug Use* **2020**, *13*, 62-64.
164. Wang, Q.L.; Huang, C.; Chen, X.H.; Fan, C.W.; Che, C.X.; Chen, S.S. Effect of vildagliptin on immune factors in patients with concurrent diabetes and coronary artery lesions. *Electronic Journal of Clinical Medical Literature* **2020**, *7*, 46, 48.
165. Feng, Y. Efficacy of vildagliptin combined with metformin in newly diagnosed type 2 diabetes and its effect on microinflammatory state. *Medical Innovation of China* **2019**, *16*, 110-113.
166. Liao, Q.H.; Chen, Y.H.; Guo, Z.Q.; Shi, Z.Z.; Lai, K.B.; Tan, Y.Q.; Wang, R. Efficacy of vildagliptin combined with metformin in the treatment of type 2 diabetes. *Journal of Tropical Medicine* **2018**, *18*, 685-688.
167. Fu, C.M.; Chu, J.Y.; Yan, L.T. Efficacy analysis of metformin combined with vildagliptin in type 2 diabetes mellitus. *China & Foreign Medical Treatment* **2018**, *37*, 112-113.
168. Peng, X.H.; Chen, H.; Fu, Y.; Qiu, Y.; Chen, X.Y.; Liu, H.D. Effect of vildagliptin combined with metformin on autoantibodies in patients with type 2 diabetes. *Lingnan Journal of Emergency Medicine* **2018**, *23*, 276-278.
169. Li, S.J.; Fang, M.L. Efficacy of metformin combined with vildagliptin in the treatment of type 2 diabetes and its effect on serum oxidation substance levels in patients. *Shanghai Medical & Pharmaceutical Journal* **2018**, *39*, 19-20.
170. Su, J.F. Clinical efficacy observation of vildagliptin combined with metformin in the treatment of type 2 diabetes. *Journal of Shanxi University of Chinese Medicine* **2018**, *19*, 47-49.
171. Zhao, Y.H.; Hu, Q.Y.; Lai, Y.N.; Peng, S.J. Effect of vildagliptin combined with metformin on inflammatory state and oxidative stress in patients with type 2 diabetes. *Journal of Ningxia Medical University* **2017**, *39*, 216-219.
172. Qu, J.C.; Xu, X.; Zhao, H.Y.; Li, L.; Zhao, L.; Liang, Y.L.; Wang, L.S.; Wang, T.; Zhu, K.S. Efficacy analysis of vildagliptin combined with metformin in the treatment of patients newly diagnosed with type 2 diabetes. *Chinese Journal of Diabetes* **2017**, *25*, 1093-1096.
173. Shi, C.L. A controlled study of vildagliptin combined with metformin in the treatment of type 2 diabetes complicated with cardio-cerebrovascular disease. *Clin. Med.* **2017**, *37*, 67-69.
174. Li, R.; Wang, H.C.; Liu, M.J. Effect of vildagliptin combined with metformin on improving islet β-cell function and insulin resistance of patients with newly diagnosed type 2 diabetes. *Modern Journal of Integrated Traditional Chinese and Western Medicine* **2016**, *25*, 2575-2577.
175. Zhao, X.W.; Tian, J.; Shen, J.X.; Yang, L.X.; Zhou, Y.H. Effect of vildagliptin on pulmonary function in obese type 2 diabetes patients treated with metformin. *Anhui Medical and Pharmaceutical Journal* **2016**, *20*, 369-373.
176. Jia, X.W.; Jia, H.T. Short-term efficacy and safety analysis of metformin combined with vildagliptin in the treatment of type 2 diabetes. *China Journal of Emergency Resuscitation and Disaster Medicine* **2015**, *10*, 460-463.
177. Zheng, Z.P.; Tian, F.; Zheng, Z.G.; Yu, M. Effect of vildagliptin combined with metformin on IMT, serum APN and Hcy levels in patients with type 2 diabetes complicated by cardio-cerebrovascular disease. *Journal of Nanchang University(Medical Sciences)* **2015**, *55*, 62-65.
178. Liang, C.Y.; Tan, S.Y.; Wang, Y.L. Clinical efficacy observation of vildagliptin (DPP-4 inhibitor) combined with oral hypoglycemic drugs in the treatment of 84 cases of type 2 diabetes. *Journal of Practical Diabetology* **2015**, 49-50.
179. Cheng, L.Y. DPP-4 inhibitor combined with metformin in the treatment of 75 cases of type 2 diabetes. *Journal of Yangtze University(Natural Science Edition)* **2014**, *11*, 66-68.
180. Chen, X.S.; Li, G.Y.; Wu, J.Y. Clinical efficacy study of vildagliptin combined with metformin in the treatment of elderly patients with type 2 diabetes. *Capital Medicine* **2014**, 73-74.
181. Yan, Z.D.; Hu, J.P.; Tao, D.Q.; Zhou, H.Y. Effect and safety of vildagliptin combined with metformin in the treatment of type 2 diabetes. *Chinese Journal of Difficult and Complicated Cases* **2014**, *13*, 156-158.
182. Lai, K.B.; Shi, Z.Z.; Huang, Z.C.; Guo, Z.Q.; Liao, Q.H.; Chen, Y.H. Effect of vildagliptin combined with metformin on IL-6, TNF-a, CRP in patients with type 2 diabetes. *Modern Diagnosis and Treatment* **2014**, *25*, 5544-5545.
183. Li, R.H. Effect analysis of metformin combined with DPP-4 inhibitor (linagliptin tablet) in the treatment of type 2 diabetes. *Journal of Frontiers of Medicine* **2020**, *10*, 116-117.
184. Wang, X.P.; Duan, B.H.; Du, F.M.; Liu, Y.; Bian, B.F. Clinical efficacy analysis of DDP-4 inhibitor in the treatment of elderly patients with diabetes. *Diabetes New World* **2020**, *23*, 61-63.
185. Zhou, D.Q. Effect of linagliptin combined with metformin on glycolipid metabolism in type 2 diabetes. *The Medical Forum* **2020**, *24*, 950-952.
186. Sun, T.T.; Hao, W.; Li, M.M.; Lu, L.; Fan, H.Q. Clinical study of linagliptin tablets combined with metformin tablets

in the treatment of newly diagnosed type 2 diabetes. *The Chinese Journal of Clinical Pharmacology* **2019**, *35*, 633-636.
187. Chen, K.F. Effect of linagliptin combined with metformin on blood glucose and islet β-cell function in patients with type 2 diabetes. *Journal of Practical Diabetology* **2019**, *15*, 25-26.
188. Guo, C.M. Efficacy study of linagliptin combined with metformin in the treatment of newly diagnosed type 2 diabetes. *Diabetes New World* **2019**, *22*, 73-74.
189. Bai, F.W.; Liu, X.H.; Liu, X.P. To observe the clinical efficacy and safety of linagliptin tablets combined with metformin tablets in the treatment of newly diagnosed type 2 diabetes. *Diabetes World* **2019**, *16*, 90-91.
190. Niu, S. Efficacy and safety of linagliptin combined with metformin in the treatment of type 2 diabetes. *China Rural Health* **2019**, *11*, 51-52.
191. Jin, J. A randomized controlled study of linagliptin in the treatment of newly diagnosed type 2 diabetes. *Chinese Practical Journal of Rural Doctor* **2018**, *25*, 40-42.
192. Wang, X.W. Effect analysis of adding linagliptin to patients with type 2 diabetes who were poorly controlled by metformin alone. *Contemporary Medicine* **2018**, *24*, 174-175.
193. Gao, F.D. Effect of linagliptin combined with metformin on insulin resistance and islet β-cells in patients with type 2 diabetes. *Diabetes New World* **2017**, *20*, 86-87.
194. Zhao, X.W.; Tian, J.; Shen, J.X.; Yang, L.X.; Zhou, Y.H. Clinical observation of the effect of linagliptin on cardiovascular risk factors in obese type 2 diabetes patients treated with metformin. *Guizhou Medical Journal* **2016**, *40*, 40-42.
195. Zheng, F.P. Effect of DPP-4 inhibitor alogliptin on blood lipids in patients newly diagnosed with type 2 diabetes, China Medical University, 2016-01-01.
196. Wu, S.Y.; Zhao, J.L.; Xiong, C.Y.; Sun, C.; Mao, X.F. Effect of drug combination therapy on serum insulin, glucose, white blood cell count and lymphocyte level in patients with diabetes. *Journal of Tropical Medicine* **2020**, *20*, 380-383.
197. Cai, Y.H. Effect analysis of alogliptin combined with metformin on islet β-cell function and inflammatory state in patients newly diagnosed with type 2 diabetes. *Journal of Practical Diabetology* **2020**, *16*, 85-86.
198. Zhang, X.T.; Zeng, W.W.; Hu, M.H.; Liu, L.J.; Mao, Q.D.; Zhou, M.Q. Effect study of alogliptin on carotid intimal thickness in patients with diabetes. *Practical Clinical Journal of Integrated Traditional Chinese and Western Medicine* **2020**, *20*, 27-28.
199. Wei, C.W.; Huang, Z.Y.; Li, J.E.; Dai, D.D. Effect of alogliptin combined with metformin in the treatment of patients with type 2 diabetes complicated by dyslipidemia. *Chinese Journal of Modern Drug Application* **2020**, *14*, 182-184.
200. Zheng, Z.G.; Yu, M.; Tang, P.; Chen, Q.H. Effect of alogliptin combined with metformin on islet β-cell function and inflammatory state in patients newly diagnosed with type 2 diabetes. *Evaluation and Analysis of Drug-Use in Hospitals of China* **2019**, *19*, 1089-1091.
201. Qiao, C.F.; Chen, Y.; Huang, Q.J. Efficacy and pharmacoeconomic analysis of alogliptin combined with metformin in the treatment of 47 cases of type 2 diabetes. *Chinese Journal of Rational Drug Use* **2019**, *16*, 34-36.
202. Weng, Y.; Wang, J.K.; Song, Y.L.; Tang, Y.S.; Li, Y.; Zheng, F.P.; Li, C.L.; Zhou, Y.J. Efficacy and safety analysis of alogliptin combined with metformin in the treatment of type 2 diabetes complicated with nonalcoholic fatty liver disease. *Practical Pharmacy and Clinical Remedies* **2016**, *19*, 713-715.
203. Xie, L.H. Clinical observation of metformin combined with pioglitazone in the treatment of incipient type 2 diabetes. *China Pharmacy* **2016**, *27*, 1629-1631.
204. Li, J.Y. Prognostic observation of rosiglitazone combined with metformin in the treatment of type 2 diabetes. *Diabetes New World* **2014**, *34*, 11-13.
205. Lei, C.Y.; Liang, J. Clinical observation of metformin combination therapy for treating type 2 diabetes. *Medical Information* **2014**, *27*, 80.
206. Yang, X.D.; Li, W.Q. Rosiglitazone hydrochloride combined with metformin in the treatment of 75 cases of type 2 diabetes. *Journal of Community Medicine* **2013**, *11*, 51-52.
207. Yu, C.Y. Clinical study of metformin combined with rosiglitazone in the treatment of type 2 diabetes. *Chinese and Foreign Medical Research* **2013**, *11*, 21-22.
208. Gao, Y. Clinical study of metformin combined with rosiglitazone in the treatment of type 2 diabetes. *Chinese Journal of Practical Medicine* **2013**, *40*, 68-69.
209. Liu, M. Efficacy analysis of rosiglitazone tartrate combined with metformin in the treatment of type 2 diabetes. *Guide of China Medicine* **2012**, 578-579.
210. Li, Y.G.; Zeng, J.E.; Ning, S.X. Clinical observation of rosiglitazone combined with metformin sustained-release tablets in the treatment of newly diagnosed type 2 diabetes. *Journal of Clinical and Experimental Medicine* **2011**, *10*, 128-129.
211. Li, G.H. Efficacy and safety evaluation of rosiglitazone combined with metformin in the treatment of newly diagnosed type 2 diabetes. *Hebei Medical Journal* **2010**, *32*, 1995-1997.
212. Xia, Z.X.; Guo, Y.X.; Zhou, M.; Yan, J. Efficacy observation of metformin sustained-release tablets combined with rosiglitazone maleate in the treatment of type 2 diabetes. *Journal of Clinical and Experimental Medicine* **2009**, *8*, 27-28.
213. Zhong, F.D.; Guo, X.Z.; Sha, Y.C.; Jiao, G.W.; Xi, Y.N.; Mo, L.L. Effect observation of rosiglitazone combined with metformin sustained-release tablets in the treatment of newly diagnosed type 2 diabetes. *Medical Journal of Communications* **2009**, *23*, 48-49.

214. Xie, X.M. Efficacy observation of pioglitazone combined with metformin in the treatment of type 2 diabetes. *Chinese Journal of Clinical Rational Drug Use* **2020**, *13*, 65-66.
215. Di, J.N.; Hu, J.R. Effect observation of pioglitazone combined with metformin in obese type 2 diabetes patients with poor treatment compliance. *Ningxia Medical Journal* **2020**, *42*, 506-508.
216. Cao, W.F. Pharmacological analysis of pioglitazone combined with metformin in the treatment of type 2 diabetes in outpatient pharmacy. *Healthmust-Readmagazine* **2020**, 24.
217. Shi, Y.Q. Clinical effect analysis of metformin combined with pioglitazone in the treatment of type 2 diabetes. *Health Guide* **2020**, 60.
218. Zhang, H. To explore clinical significance of metformin combined with pioglitazone in the treatment of type 2 diabetes. *Diabetes New World* **2019**, *22*, 67-68.
219. Hu, K.Y. Clinical efficacy and safety of pioglitazone hydrochloride in the treatment of type 2 diabetes. *Chinese Journal of Clinical Rational Drug Use* **2019**, *12*, 6-7.
220. Wang, H.J.; Pi, Y.Z.; Li, L. Clinical effect of pioglitazone combined with metformin in patients with type 2 diabetes complicated with metabolic syndrome. *Heilongjiang Medicine Journal* **2019**, *32*, 857-859.
221. Gao, S.W. Clinical efficacy observation of pioglitazone combined with metformin in the treatment of type 2 diabetes. *Chinese Baby* **2019**, 80.
222. Guo, H.; Shi, J.L. Clinical effect analysis of metformin combined with pioglitazone in the treatment of type 2 diabetes. *Henan Medical Research* **2018**, *27*, 1425-1426.
223. Chen, G.M. Efficacy observation of metformin combined with pioglitazone in the treatment of patients with type 2 diabetes in community. *Psychological Doctor* **2018**, *24*, 100-101.
224. Ren, B.X.; Tian, D.Z. Clinical effect analysis of metformin combined with pioglitazone in the treatment of type 2 diabetes. *Journal of Medical Forum* **2018**, *39*, 145-147.
225. Li, S.Q. Clinical efficacy evaluation of metformin hydrochloride combined with pioglitazone hydrochloride on hypoglycemic effect in patients with type 2 diabetes. *Anti-Infection Pharmacy* **2016**, *13*, 464-466.
226. Zhang, C.R. Effect observation of pioglitazone on blood lipid in type 2 diabetes. *Diabetes New World* **2016**, *19*, 39-40.
227. Zhu, X.X. Efficacy observation of metformin combined with pioglitazone in the treatment of type 2 diabetes. *Diabetes New World* **2015**, *35*, 28-30.
228. Wang, T. Effect analysis of metformin combined with pioglitazone in the treatment of type 2 diabetes. *Chinese Journal of Clinical Rational Drug Use* **2015**, *8*, 40-41.
229. Ma, Z.X. Effect analysis of pioglitazone hydrochloride combined with metformin sustained-release tablets in the treatment of type 2 diabetes. *Diabetes New World* **2015**, *35*, 52-53.
230. Zhou, Y.Q.; Jin, F.; Zhang, M.; Hu, J.S. Effect analysis of metformin combined with pioglitazone in the treatment of elderly patients with type 2 diabetes. *Journal of Frontiers of Medicine* **2015**, *5*, 60-61.
231. Zhang, C.H. Clinical efficacy of metformin combined with pioglitazone in the treatment of type 2 diabetes. *World Latest Medicine Information* **2015**, 111-112.
232. Ren, Y.L. Effect observation of pioglitazone and metformin tablets in the treatment of type 2 diabetes. *China Health Standard Management* **2015**, 103-104.
233. Yang, H.M.; Qin, Q.H. Efficacy observation of pioglitazone combined with metformin in the treatment of type 2 diabetes. *Journal of Frontiers of Medicine* **2015**, *5*, 146-148.
234. Shen, C.M. Value analysis of metformin combined with pioglitazone in the treatment of refractory type 2 diabetes. *Shaanxi Medical Journal* **2014**, *43*, 1077-1079.
235. Du, Y.J. Efficacy observation of metformin combined with pioglitazone in the treatment of type 2 diabetes. *Health Research* **2014**, *34*, 212-213.
236. Zhang, Y.; Yang, H.Y.; Tan, Z.M. Effect of pioglitazone combined with metformin on high sensitivity C-reactive protein and D-dimer in patients with type 2 diabetes. *Modern Journal of Integrated Traditional Chinese and Western Medicine* **2013**, *22*, 723-724.
237. Bi, X.S. Efficacy observation of metformin combined with pioglitazone in the treatment of type 2 diabetes. *Prevention and Treatment of Cardiovascular Disease* **2013**, 49-51.
238. Zhong, J. Efficacy observation of metformin hydrochloride combined with pioglitazone in the treatment of type 2 diabetes. *Seek Medical and Ask the Medicine* **2013**, 303-304.
239. Jiang, H. Efficacy observation of metformin combined with pioglitazone in the treatment of type 2 diabetes. *China Practical Medicine* **2012**, *7*, 153-155.
240. Zhu, Y.F.; Li, B. Clinical analysis of metformin combined with pioglitazone in the treatment of 134 cases of type 2 diabetes. *China Health Industry* **2012**, *9*, 57-58.
241. Teng, Y.P. A controlled study of pioglitazone combined with metformin in the treatment of type 2 diabetes. *China Health Industry* **2012**, *9*, 5-6.
242. Yang, Y.B. Clinical observation of pioglitazone hydrochloride combined with metformin in the treatment of type 2 diabetes. *Chinese and Foreign Medical Research* **2012**, 23-24.
243. Guo, H.F. Efficacy analysis of pioglitazone and metformin in the treatment of type 2 diabetes. *Chinese Journal of Misdiagnostics* **2011**, *11*, 3136-3137.
244. Hong, Y.J.; Guo, W.Y. Efficacy and safety analysis of pioglitazone combined with metformin in the treatment of type 2 diabetes. *China Pharmacy* **2010**, *21*, 2258-2259.
245. Deng, J.H. Efficacy observation of pioglitazone combined with metformin in the treatment of type 2 diabetes.

Proceeding of Clinical Medicine **2009**, *18*, 809-811.
246. Li, L. Clinical effect study of dapagliflozin combined with metformin in the treatment of type 2 diabetes. *Electronic Journal of Practical Gynecological Endocrinology* **2020**, *7*, 192-193.
247. Song, Q.Q.; Lao, B.C.; Huang, C.; Zhao, Y.N.; Chen, W.J. Effect of dapagliflozin on adiponectin and leptin levels in obese type 2 diabetes patients. *Modern Hospitals* **2020**, *20*, 1215-1217, 1220.
248. Yang, Y. Clinical observation of dapagliflozin combined with metformin in the treatment of type 2 diabetes complicated with metabolic syndrome, Xinxiang Medical University.
249. Li, J.S.; Zhang, Y.P.; Ren, M.M. Clinical efficacy study of dapagliflozin combined with metformin in the treatment of type 2 diabetic nephropathy. *Heilongjiang Medical Journal* **2020**, *44*, 948-950.
250. Ren, H.X. Clinical effect and safety observation of metformin combined with dapagliflozin in patients with type 2 diabetes and heart failure. *Living over 100* **2020**, 256.
251. Wang, Y.; Yuan, X. Effect of dapagliflozin combined with metformin on glycolipid metabolism in patients with type 2 diabetes. *Tibetan Medicine* **2020**, *41*, 73-74.
252. Liu, X.J. Effect observation of dapagliflozin combined with metformin in the treatment of type 2 diabetes. *Medical Journal of Chinese People's Health* **2019**, *31*, 44-46.
253. Xu, J.; Qiu, C.J.; Gong, W.W.; Li, X.; Zhang, Y.Y. Clinical observation of dapagliflozin combined with metformin in the treatment of adult patients newly diagnosed with type 2 diabetes. *Clinical Medication Journal* **2019**, *17*, 66-69.
254. Liu, X.Y.; Cao, H.W.; Lai, J.B.; Fang, Y.J.; Huang, X.X.; Li, X.M. Clinical observation of empagliflozin combined with metformin in the treatment of newly diagnosed overweight and obese type 2 diabetes. *Medical & Pharmaceutical Journal of Chinese People's Liberation Army* **2020**, *32*, 26-29.
255. Liu, S.X.; Zhang, Y. Efficacy and safety analysis of empagliflozin combined with metformin in the treatment of type 2 diabetes. *World Latest Medicine Information* **2020**, 195.
256. He, M.L.; Ding, Q.L.; Wang, J.J. Efficacy of empagliflozin combined with metformin in obese type 2 diabetes patients. *Electronic Journal of Clinical Medical Literature* **2020**, *7*, 172, 177.
257. Wei, Z.X. Effect observation of exenatide combined with metformin in obese type 2 diabetes patients. *Henan Medical Research* **2018**, *27*, 3785-3786.
258. Hou, X.L.; Wu, S.G.; Guo, Y.P. Efficacy observation of exenatide in the treatment of obese type 2 diabetes complicated with obstructive sleep apnea hypopnea syndrome. *Journal of Xinxiang Medical University* **2018**, *35*, 50-53.
259. Zhou, R.F.; Zhong, S.; Guo, Y.; Sun, Y.J.; Shao, Z.X. Effect of metformin combined with exenatide on islet function in patients with newly diagnosed type 2 diabetes. *Clinical Focus* **2017**, *32*, 965-968.
260. Li, B.W.; Yu, J.L.; Zhang, H.; Jiang, L.; Li, Y.L.; Li, N.; Zhang, P. Effect of GLP-1 receptor agonist on early insulin secretion and body weight in type 2 diabetes. *Medical Information* **2015**, *28*, 142-143.
261. Pan, N.N. Clinical observation of exenatide combined with metformin in the treatment of elderly patients with type 2 diabetes. *Chinese Journal of Clinical Rational Drug Use* **2014**, 76-77.
262. Li, J.S.; Zhang, Y.P.; Ren, M.M. Effect observation of liraglutide combined with metformin in the treatment of type 2 diabetes and its influence on blood glucose. *Heilongjiang Journal of Traditional Chinese Medicine* **2020**, *49*, 39-40.
263. Liu, Y.D. Effect of liraglutide combined with metformin in type 2 diabetes. *Journal of Shanxi Health Vocational College* **2020**, *30*, 26-28.
264. Lin, Y.; Feng, L. Effect of liraglutide combined with metformin on serum Hcy, CysC, hs-CRP levels and glycolipid metabolism in elderly patients with type 2 diabetes. *Chinese Journal of Gerontology* **2020**, *40*, 502-504.
265. Yang, M.; Cun, Y.K. To study the clinical efficacy of liraglutide combined with metformin in the treatment of type 2 diabetes. *Diabetes World* **2020**, 94.
266. Jia, C.; Zhang, T.; Wang, H.; Qi, F.J. Effect of liraglutide on blood lipid, vascular endothelial function and liver fibrosis in patients with concurrent nonalcoholic fatty liver disease and type 2 diabetes. *Journal of Practical Hepatology* **2020**, *23*, 203-206.
267. Liu, W.F. Effect of metformin combined with liraglutide in the treatment of obese type 2 diabetes. *Chinese Health Care* **2020**, *38*, 140-142.
268. Liu, H.Y.; Liu, J.Y.; He, G.X. Efficacy of liraglutide combined with metformin in the treatment of obese diabetes and its effect on microinflammation and oxidative stress indexes. *Chinese Journal of Coal Industry Medicine* **2020**, *23*, 186-191.
269. Guo, W.; Tian, W.; Lin, L.; Xu, X. Liraglutide or insulin glargine treatments improves hepatic fat in obese patients with type 2 diabetes and nonalcoholic fatty liver disease in twenty-six weeks: A randomized placebo-controlled trial. *Diabetes Res. Clin. Pr.* **2020**, *170*.
270. Lai, X.Y.; Shen, Y.F.; Yang, Y.; Zou, F.; Cai, X.; Xiong, Y.; Lei, S.H. Evaluation of clinical efficacy of liraglutide combined with metformin in the treatment of newly diagnosed type 2 diabetes with overweight/obesity. *Jiangxi Medical Journal* **2020**, *55*, 95-97.
271. Wang, Y.Q. Clinical efficacy and safety assessment of liraglutide combined with metformin in treatment of type 2 diabetes mellitus. *Hebei Medical Journal* **2019**, *41*, 392-395.
272. Li, L.B.; Weng, J.L.; Li, J.H. Clinical efficacy of liraglutide combined with metformin in obese type 2 diabetes patients and its effect on serum chemokine and lactone level. *Chongqing Medicine* **2019**, *48*, 33-36.
273. Chen, C.X. Effect of liraglutide combined with metformin on body weight, blood glucose and islet function in obese type 2 diabetes patients. *Journal of Practical Diabetology* **2019**, *15*, 24-25.
274. Wu, J. Clinical efficacy study of liraglutide in the treatment of obese type 2 diabetes. *Our Health* **2019**, 47.

275. Wang, L.Y. Efficacy of liraglutide in patients with type 2 diabetes and nonalcoholic fatty liver disease. *Clinical Journal of Chinese Medicine* **2019**, *11*, 52-53.
276. Shi, G.L.; Guo, H.Y. Efficacy analysis of liraglutide combined with metformin in T2MD patients with obesity. *Our Health* **2019**, 68.
277. Zu, Q. Effect of liraglutide combined with metformin on blood glucose and islet β-cell function in the treatment of obese type 2 diabetes. *Journal of Practical Diabetology* **2019**, *15*, 21-22.
278. Ye, X.X.; Yang, L. Clinical value of liraglutide combined with metformin in the treatment of patients newly diagnosed with obese T2DM and its effect on constitution and metabolism. *Sichuan Medical Journal* **2019**, *40*, 951-955.
279. Cheng, X.F. Clinical effect of liraglutide combined with metformin hydrochloride tablets in the treatment of type 2 diabetes and its influence on patients' compliance. *Clinical Research and Practice* **2019**, *4*, 34-36.
280. Jian, X.H.; Shen, J.; Xu, J.B.; Liu, Y.; Chen, Y.M. Clinical study of liraglutide injection in the treatment of overweight and obese type 2 diabetes patients with microalbuminuria. *The Chinese Journal of Clinical Pharmacology* **2018**, *34*, 2803-2806.
281. Wang, X.Y.; Lv, C.; Sun, Y.Y.; Liang, L. Effect of liraglutide combined with metformin in the treatment of type 2 diabetes. *Journal of Community Medicine* **2018**, *16*, 37-38.
282. Yang, X.Q. Efficacy of liraglutide combined with metformin in the treatment of type 2 diabetes. *China & Foreign Medical Treatment* **2018**, *37*, 78-80.
283. Tang, X.Y. Clinical effect observation of liraglutide combined with metformin in the treatment of type 2 diabetes. *Electronic Journal of Clinical Medical Literature* **2018**, *5*, 164-165.
284. Su, J.F. Clinical efficacy observation of liraglutide combined with metformin in the treatment of obese type 2 diabetes. *Journal of Practical Medical Techniques* **2018**, *25*, 816-819.
285. Ma, Y. Effect observation of liraglutide combined with metformin in the treatment of obese type 2 diabetes. *Henan Medical Research* **2018**, *27*, 3764-3765.
286. Zhao, P.Y. Efficacy analysis of liraglutide combined with metformin in type 2 diabetes. *Diabetes World* **2018**, *15*, 104.
287. He, X.Y. Effect observation of glucagon-like peptide-1 analogues combined with metformin in the treatment of obese type 2 diabetes. *Henan Medical Research* **2018**, *27*, 2979-2980.
288. Zhang, L. Effect analysis of liraglutide plus metformin on the microinflammatory state in obese type 2 diabetes. *Diabetes New World* **2017**, *20*, 77-78.
289. Tu, C.F.; Shen, F.X. Effect of liraglutide combined with metformin on inflammatory state and metabolic indexes in patients with type 2 diabetes and obesity. *Chinese Rural Health Service Administration* **2017**, *37*, 1409-1411.
290. Si, M.; Liao, Y.G.; Yang, H. Clinical efficacy of liraglutide combined with metformin in the treatment of newly diagnosed obese type 2 diabetes patients. *Chinese Journal of Geriatric Care* **2017**, *15*, 39-41.
291. Qu, J.C.; Li, L.; Zhao, L.; Wang, T.; Zhu, K.S. Efficacy of glucagon-like peptide-1 analogue in overweight or obese patients newly diagnosed with type 2 diabetes. *China Medicine* **2017**, *12*, 1012-1014.
292. Wang, C.X.; Gao, H.; Wang, Y.L. Effect of liraglutide combined with metformin on vascular endothelial function in Uygur and Han nationality patients with obese type 2 diabetes in Xinjiang. *Journal of Xinjiang Medical University* **2016**, *39*, 968-971.
293. Song, W.R. Clinical efficacy observation of liraglutide combined with metformin in the treatment of obese type 2 diabetes patients. *Drug Evaluation* **2016**, *13*, 23-26.
294. Xue, G.F.; Yang, X.R. Clinical efficacy of liraglutide combined with metformin in the treatment of type 2 diabetes. *Journal of Today Health* **2016**, *15*, 160.
295. Yao, Y.N.; Zhu, Z.L.; Wang, Y.L. Clinical efficacy of liraglutide combined with metformin hydrochloride in the treatment of Uygur and Han nationality patients with obese type 2 diabetes. *Journal of Xinjiang Medical University* **2015**, *38*, 1527-1530.
296. Liu, C.L. Effect of liraglutide on main physiological indices of type 2 diabetes patients. *China Pharmaceuticals* **2015**, *24*, 30-31.
297. Zhao, F.L.; Qi, M.; Liu, H. Efficacy of liraglutide combined with metformin in the treatment of type 2 diabetes patients with obesity and its effect on microinflammatory state. *China Journal of Modern Medicine* **2015**, 43-46.
298. Wang, Y.; Cui, Y.G. Effect of insulin glargine combined with metformin on blood glucose, SOD, MDA and GSH-PX in patients with type 2 diabetes. *Modern Medicine and Health Research* **2020**, *4*, 113-115.
299. Wang, F.; Song, S.Y. Clinical effect of insulin glargine combined with metformin in the treatment of 40 cases of type 2 diabetes. *Diabetes World* **2020**, *17*, 158-159.
300. Zeng, C.M.; Qu, H. Clinical effect of insulin glargine combined with metformin in the treatment of elderly patients with type 2 diabetes and its effect on FBG, 2hPBG and HbA1c levels. *Our Health* **2020**, 33.
301. Li, Z.Z. Clinical effect observation of insulin glargine combined with metformin in elderly patients with type 2 diabetes. *Journal of Practical Diabetology* **2019**, *15*, 24-25.
302. Chang, J. Effect and safety of insulin glargine combined with metformin in the treatment of type 2 diabetes. *Journal of Bethune Medical Science* **2019**, *17*, 244-246.
303. Zhang, W.H.; Hu, H.X. Efficacy and safety analysis of insulin glargine combined with metformin in intensive treatment of new-onset type 2 diabetes. *Journal of North Pharmacy* **2015**, *12*, 144-145.
304. Liu, S.J. Efficacy study of insulin glargine combined with metformin in the treatment of type 2 diabetes. *China Practical Medicine* **2015**, *10*, 157-158.
305. Ma, L. Efficacy analysis of insulin glargine combined with metformin in the treatment of type 2 diabetes. *Jilin Medical*

Journal **2015**, 714-715.
306. Li, M. Clinical study of insulin glargine combined with metformin in the treatment of type 2 diabetes. *Medicine and Health* **2015**, *0*, 149.
307. He, R.Y. Clinical efficacy and safety of insulin glargine injection combined with metformin in the treatment of type 2 diabetes. *Diabetes New World* **2015**, 36.
308. Ma, Y.M.; Huang, Y. Clinical efficacy of insulin glargine combined with metformin in the treatment of type 2 diabetes. *China Journal of Pharmaceutical Economics* **2015**, *10*, 83-84.
309. He, Y.F.; Zhao, Q. A randomized controlled study of metformin monotherapy and different combination regimens in the treatment of type 2 diabetes. *Chinese Manipulation and Rehabilitation Medicine* **2014**, *5*, 122-123.
310. Liu, G.H. Efficacy observation of recombinant insulin glargine combined with metformin in the treatment of type 2 diabetes. *New Medicine* **2018**, *28*, 118-119.
311. Lin, J.B.; Li, Y.M. Study of the value of insulin detemir combined with metformin on TC and TG in patients with newly diagnosed type 2 diabetes. *Home Medicine* **2019**, 48.
312. Li, R.L. Effect of Novolin N combined with metformin on blood glucose, C-peptide and CRP in patients with type 2 diabetes. *Modern Journal of Integrated Traditional Chinese and Western Medicine* **2014**, *23*, 1207-1209.
313. Ding, J.P.; Tang, H. Analysis of clinical effect of metformin sustained-release tablets combined with Novolin 30R in type 2 diabetes. *Diet Science* **2019**, *0*.
314. Liu, J.; Cui, X.H.; Wang, L.Y.; Xu, Y.K. Effect of premixed insulin combined with metformin on recovery of islet function in patients newly diagnosed with type 2 diabetes. *Hebei Medicine* **2019**, *25*, 18-22.
315. Cao, Y.Q.; Fan, H.B.; Lu, Q.H.; Dong, Y.; Gu, Y.P. Efficacy observation of metformin combined with humulin in the treatment of obese type 2 diabetes with newly diagnosed diabetic foot. *Drugs & Clinic* **2014**, *29*, 615-618.
